# Federal Funding and Citation Metrics of US Biomedical Researchers, 1996 to 2022

**DOI:** 10.1001/jamanetworkopen.2022.45590

**Published:** 2022-12-07

**Authors:** John P. A. Ioannidis, Iztok Hozo, Benjamin Djulbegovic

**Affiliations:** 1 Department of Medicine, Stanford University, Stanford, California; 2 Meta-Research Innovation Center at Stanford University, Stanford, California; 3 Department of Epidemiology and Population Health, Stanford University, Stanford, California; 4 Department of Biomedical Data Science, Stanford University, Stanford, California; 5 Department of Statistics, Stanford University, Stanford, California; 6 Department of Mathematics, Indiana University Northwest, Gary; 7 Beckman Research Institute, Department of Computational and Quantitative Medicine, City of Hope, Duarte, California

## Abstract

**Importance:**

Both citation and funding metrics converge in shaping current perceptions of academic success.

**Objective:**

To evaluate what proportion of the most-cited US-based scientists are funded by biomedical federal agencies and whether funded scientists are more cited than nonfunded ones.

**Design, Setting, and Participants:**

This survey study used linkage of a Scopus-based database on top-cited US researchers (according to a composite citation metric) and the National Institutes of Health RePORTER database of federal funding (33 biomedical federal agencies). Matching was based on name and institution. US-based top-cited scientists who were allocated to any of 69 scientific subfields highly related to biomedicine were considered in the main analysis. Data were downloaded on June 11, 2022.

**Main Outcomes and Measures:**

Proportion of US-based top-cited biomedical scientists who had any (1996-2022), recent (2015-2022), and current (2021-2022) funding. Comparisons of funded and nonfunded scientists assessed total citations and a composite citation index.

**Results:**

There were 204 603 records in RePORTER (1996-2022) and 75 316 US-based top-cited scientists in the career-long citation database; 40 887 scientists were included in the main analysis. The proportion of US-based top-cited biomedical scientists (according to career-long citation impact) who had received any federal funding from biomedical research agencies was 62.7% (25 650 of 40 887) for any funding (1996-2022), 23.1% (9427 of 40 887) for recent funding (2015-2022), and 14.1% (5778 of 40 887) for current funding (2021-2022). Respective proportions were 64.8%, 31.4%, and 20.9%, for top-cited scientists according to recent single-year citation impact. There was large variability across scientific subfields (eg, current funding: 31% of career-long impact top-cited scientists in geriatrics, 30% in bioinformatics and 29% in developmental biology, but 0% in legal and forensic medicine, general psychology and cognitive sciences, and gender studies). Funded top-cited researchers were overall more cited than nonfunded top-cited scientists (median [IQR], 9594 [5650-1703] vs 5352 [3057-9890] citations; *P* < .001) and substantial difference remained after adjusting for subfield and years since first publication. Differences were more prominent in some specific biomedical subfields.

**Conclusions and Relevance:**

In this survey study, biomedical federal funding had offered support to approximately two-thirds of the top-cited biomedical scientists at some point during the last quarter century, but only a small minority of top-cited scientists had current federal biomedical funding. The large unevenness across subfields needs to be addressed with ways that improve equity, efficiency, excellence, and translational potential.

## Key Points

**Question:**

What is the association between federal biomedical funding and high citation impact among US scientists?

**Findings:**

In this survey study, two-thirds of the top-cited biomedical scientists received some federal funding from biomedical agencies in the last quarter of a century, but only a small minority had such funding at the time of the survey, with large variability across research subfields. Funded top-cited scientists attracted more citations than nonfunded ones.

**Meaning:**

This large-scale mapping of the citation impact and funding association can offer insights for a rational reform of the funding landscape and the reward system in biomedicine.

## Teaser

This survey study evaluates the association between federal funding and citation metrics of US biomedical researchers from 1996 to 2022.

## Introduction

Citation metrics^1^ are key indicators of research productivity in the academic reward system, despite widely acknowledged limitations.^2^ Research funding is also considered indicative of scientific success, perhaps even more so in the US. Successful competition for research funding, particularly from federal agencies such as the National Institutes of Health (NIH), is used by most US academic institutions in promotion, tenure, and recruitment decisions.^3^ Institutions also incentivize their faculty to seek federal funding because it covers multiple expenses through grant-related indirect costs.

Despite the clear importance of funding on research output, to our knowledge, to date, there is no good large-scale empirical data on the association between funding and citation metrics across biomedical research. Earlier work^4^ suggested that NIH-funded studies tend to attract more citations. However, other work^5^ found that most scientists with extremely cited articles (>1000 citations) are not funded by the NIH. That analysis focused on extremely cited studies; however, it would be useful to obtain evidence on a large, comprehensive sample of highly cited scientists considering their entire career impact as well as their recent citation impact. To assess to what extent highly cited US biomedical scientists are funded by federal agencies, we merged a database of most-cited scientists^6^ with the NIH RePORTER repository of federally funded research projects of 33 US government agencies.^7^ We assessed how many biomedical scientists had received federal funding any time since 1996, since 2015, and since 2021. Secondarily, we compared whether key citation indices differed between funded and nonfunded top-cited scientists.

## Methods

### Citations Database

This survey study was an analysis of freely available public data sets and was thus exempt from institutional review board approval and the need for informed consent in accordance with 45 CFR §46. The report follows the Strengthening the Reporting of Observational Studies in Epidemiology (STROBE) reporting guideline. We used the August 2021 update of the databases of the top-cited scientists across science fields according to Scopus.^6^ Data were used separately for career-long and for single recent-year citation impact (citations received during 2020 only). Databases/code are freely available.^8^ Additional details have been published elsewhere.^9,10,11^ The database includes all the scientists who rank among the top 100 000 across all fields according to a validated composite citation index^9^ that considers 6 citation indicators (total citations, Hirsch h index, Schreiber co-authorship-adjusted hm index, citations to articles as single author, citations to articles as single or first author, and citations to articles as single, first, or last author), as previously described^9,10,11^ or they are within the top 2% of scientists of their main subfield discipline among authors who have published at least 5 articles. Classification into scientific fields and subfields follows the Science-Metrix classification with machine learning classification of multidisciplinary journals.^12^ All citation metrics are computed without excluding self-citations. Less than 5% of the top 2% scientists when self-citations are included are no longer in the top 2% of their subdiscipline when self-citations are excluded; only 0.01% fall below the top 10%.

Science-Metrix classified science in 174 subfields. We prespecified 69 subfields as being highly related to biomedicine (eAppendix in Supplement 1). For the main analysis, we considered as biomedical scientists those assigned primarily to any of these 69 subfields (ie, the most common subfield for their publications is 1 of these 69 subfields).

### NIH RePORTER Database

RePORTER^7^ is a repository of federal funded research projects from 33 US government agencies (27 NIH institutes, Agency for Healthcare Research and Quality, Centers for Disease Control and Prevention, Food and Drug Administration, Health Resources and Services Administration, Administration for Children and Family, and US Department of Veterans Affairs). We downloaded data on June 11, 2022. Funded projects cover the period 1985 to 2022, but we used only data for 1996 to 2022 for better alignment with the coverage of the citation database (Scopus data are more complete from 1996 forward).

### Matching Process

If the name in the citations and RePORTER databases is exactly the same, we considered the scientists matched. To improve on this brute force matching, we added 3 criteria for matching missing middle initials: whenever a name in RePORTER was given with full first and middle names, we tried to match it to citations database with the same last name and first name and middle name initial; whenever a name was given in RePORTER with last name and full first name but no middle name or initial, we tried to match it to the citations database with the same last name and first name (ignoring any middle name info in the citations database); and whenever a name was given in RePORTER with last name and full first name and a middle name initial, we tried to match it to the citations database with the same last name and first name (provided any middle name or initial in the citations database was not inconsistent with the RePORTER middle name initial).

### Manual Validation of Random Samples

We randomly selected 100 scientists from RePORTER who were matched to the career-long citation impact database using our algorithm and manually checked each of them for validity. Out of these 100 scientists, 85 were verified to be correct matches (same name and same institution or affiliated institutions), 3 had the same name but it was impossible to check institution match because RePORTER had missing institution data, and 12 were wrongly matched (they had the same name but were actually different scientists). Thus, the false matching rate was 12% to 15%.

We also randomly selected 100 scientists from RePORTER who were not matched to the career-long citation impact database using our algorithm. We manually checked each of them whether the citations database included them with different spelling or different country. Of 100 scientists, 4 were missed matching and for another 2 we could not be certain. Thus, the missed matching rate was 4% to 6%.

Finally, some scientists appear more than 1 time in RePORTER, because they may be entered with different spelling. Thus, the number of scientists who are not matched may be lower, if many duplicates exist. To assess the magnitude of this problem, we also randomly selected 100 scientists from RePORTER who were not matched using our algorithm and manually checked whether each of them appeared as a duplicate in RePORTER: of these 100 scientists, 94 were uniquely identified, 1 was a possible duplicate (RePORTER had another entry with the same initials and ambiguous institution data, so one cannot confirm uniqueness), and 5 were clearly repeated entries (same scientist but with a different spelling). Thus, the duplicate rate was 5% to 6%.

### Statistical Analysis

We considered all 6 possible combinations of matching different citations and funding data. These included the consideration of the career-long citation impact and, separately, the single recent year citation impact; and of funding at any time (having any active grants in years 1996-2022), recent (having any active grant in years 2015-2022), and current (having any active grant in years 2021-2022). In all 6 analyses, the main outcome of interest was the proportion of top-cited scientists who had funding. We evaluated these proportions overall in all biomedical scientists and in each of the 69 Science Metrix–defined subfields highly related to biomedicine.

We also evaluated whether in each analysis funded and nonfunded scientists differed in the total number of citations and in the composite citation indicator. Comparisons used the Mann-Whitney *U* test, and medians are presented (also occasionally means for comparison). Given that older scientists have more time to obtain grants and also to accrue citations, we also performed regressions adjusting for the publication age of the scientist (number of years since the year of the first publication). We performed linear regression, mixed-effect linear regression, and mixed-effect linear regression of log-transformed citation counts. The mixed model added random effect variation which accounts for variability of our measurements for scientists in each subfield. *P* values are 2-tailed with significance at *P* < .005.^13^ Analyses were completed in Stata statistical software version 17 (StataCorp).

## Results

### Overall Proportion of Top-Cited Scientists Funded

We started with 204 603 records in the RePORTER database for 1996 to 2022 and 186 177 records in the career-long citations database, of which 75 316 were US-based scientists (40 887 classified in the 69 subfields highly related to biomedicine). Among subfields highly related to biomedicine (Table 1), the proportion of top-cited career impact scientists who had received any federal funding from biomedical research agencies was 62.7% (25 650 of 40 887) for any funding in 1996 to 2022, 23.1% (9427 of 40 887) for recent funding, and 14.1% (5778 of 40 887) for current funding. Respective proportions were 64.8%, 31.4%, and 20.9%, for top-cited scientists according to recent single-year citation impact. For the other subfields, biomedical funding was overall very low and did not exceed 15% in any of the analyses (Table 1).

**Table 1.** Proportion Funded in 69 Subfields of Science That Are Highly Related to Biomedicine and 105 Other Subfields of Science

Top-cited US-based researchers, by career, year, and funding time^a^	Funded No./total No. (%)
Career	
Any funding time	
Total	30 417/75 316 (40)
Fields highly related to biomedicine^b^	25 650/40 887 (63)
Other fields	4821/34 429 (14)
Recent funding time	
Total	1120/75 316 (15)
Fields highly related to biomedicine^b^	9427/40 887 (23)
Other fields	1813/34 429 (5)
Current funding time	
Total	6854/75 316 (9)
Fields highly related to biomedicine^b^	5778/40 887 (14)
Other fields	1076/34 429 (3)
Recent year	
Any funding time	
Total	27 535/65 560 (42)
Fields highly related to biomedicine^b^	23 172/35 787 (65)
Other fields	4363/29 773 (15)
Recent funding time	
Total	13 195/65 560 (20)
Fields highly related to biomedicine^b^	11 252/35 787 (31)
Other fields	1943/29 773 (7)
Current funding time	
Total	8656/65 560 (13)
Fields highly related to biomedicine^b^	7465/35 787 (21)
Other fields	1191/29 773 (4)

^a^ Funding time is defined as any (ie, any grant entry in RePORTER in 1996 to 2022), recent (ie, any entry in the RePORTER that covers any year in the period 2015 until 2022), and current (ie, any entry in the RePORTER that covers 2021 and/or 2022).

^b^ Highly related fields (according to the Science-Metrix classification) are 69 subfields: that is, the 60 subfields within the larger fields of biomedical research, clinical medicine, public health and health services, and psychology and cognitive sciences, as well as 9 prespecified subfields from other large fields (ie, applied ethics, bioinformatics, biomedical engineering, biotechnology, demography, gender studies, medical informatics, medicinal and biomolecular chemistry, and veterinary sciences).

There was large variability across subfields. Table 2 shows for each analysis the 3 subfields with the highest proportions of funded top-cited scientists and the 3 subfields with the lowest proportions of funded top-cited scientists. For example, when career-long impact was considered, almost all top-cited scientists had received such funding at some point in 1996 to 2022 in the fields of developmental biology (a classification subfield in Science-Metrix that includes most -omics) (1566 of 1769 [89%]), substance abuse (240 of 277 [87%]) and immunology (1247 of 1474 [85%]), while low funding proportions were seen in several fields, with the lowest being in legal and forensic medicine (2 of 66 [3%]), psychoanalysis (4 of 50 [8%]), and general psychology and cognitive sciences (8 of 65 [12%]). For recent funding, the highest proportions were seen in developmental biology (744 of 1769 [42%]), bioinformatics (81 of 196 [41%]), and geriatrics (39 of 97 [40%]), and the same 3 subfields topped proportions funded for current funding (developmental biology, 510 of 1769 [29%]; bioinformatics, 58 of 196 [30%]; and geriatrics, 30 of 97 [31%]). Conversely, general psychology and cognitive sciences, legal and forensic medicine, and psychoanalysis subfields that are highly related to biomedicine had less than 5% of their top-cited scientists funded recently. In addition, dentistry, veterinary sciences, social psychology, anatomy and morphology, general clinical medicine, human factors, psychoanalysis, legal and forensic medicine, general psychology and cognitive sciences, and gender studies subfields that are highly related to biomedicine had less than 5% of their top-cited scientists currently funded. When single recent year impact was considered, the highest proportions of funding were dominated by geriatrics (30 of 97 [31%]), gerontology (97 of 112 [87%]), substance abuse (184 of 214 [86%]), developmental biology (510 of 1769 [29%]), and (for current funding) medical informatics (41 of 119 [34%]), while several subfields that are highly related to biomedicine had negligible proportions of their top-cited scientists being funded. Gender studies, legal and forensic medicine, and general psychology and cognitive sciences fared the worst, with 0% of top-cited scientists (Table 2).

**Table 2.** Subfields With the Highest Proportions of Funded Top-Cited Scientists and Subfields With the Lowest Proportions of Funded Top-Cited Scientists Among the 69 Subfields Prespecified as Highly Related to Biomedicine^a^

Analysis^b^	Three subfields with highest proportions of funded top-cited scientists		Three subfields with lowest proportions of funded top-cited scientists	
	Subfield	No./No. (%)	Subfield	No./No. (%)
Career-long impact, any funding	Developmental biology	1566/1769 (89)	General psychology and cognitive sciences	8/65 (12)
	Substance abuse	240/277 (87)	Psychoanalysis	4/50 (8)
	Immunology	1247/1474 (85)	Legal and forensic medicine	2/66 (3)
Career-long impact, recent funding	Developmental biology	744/1769 (42)	Psychoanalysis	1/50 (2)
	Bioinformatics	81/196 (41)	Legal and forensic medicine	1/66 (2)
	Geriatrics	39/97 (40)	General psychology and cognitive sciences	0/65
Career-long impact, current funding	Geriatrics	30/97 (31)	Legal and forensic medicine	0/66
	Bioinformatics	58/196 (30)	General psychology and cognitive sciences	0/65
	Developmental biology	510/1769 (29)	Gender studies	0/13
Recent year impact, any funding	Geriatrics	66/75 (88)	Anatomy and morphology	8/56 (14)
	Gerontology	97/112 (87)	Gender studies	2/15 (13)
	Substance abuse	184/214 (86)	Legal and forensic medicine	3/53 (6)
Recent year impact, recent funding	Geriatrics	41/75 (55)	General psychology and cognitive sciences	3/64 (5)
	Substance abuse	106/214 (50)	Legal and forensic medicine	2/53 (4)
	Developmental biology	799/1621 (49)	Anatomy and morphology	2/56 (4)
Recent year impact, current funding	Geriatrics	32/75 (43)	General psychology and cognitive sciences	2/64 (3)
	Developmental biology	590/1621 (36)	Human factors	4/177 (2)
	Medical informatics	41/119 (34)	Gender studies	0/15

^a^ Highly related fields (according to the Science-Metrix classification) are 69 subfields, ie, the 60 subfields within the larger fields of biomedical research, clinical medicine, public health and health services, and psychology and cognitive sciences as well as 9 prespecified subfields from other large fields, ie, applied ethics, bioinformatics, biomedical engineering, biotechnology, demography, gender studies, medical informatics, medicinal and biomolecular chemistry, and veterinary sciences.

^b^ Funding time is defined as 'any': any grant entry in RePORTER in 1996 to 2022; 'recent': any entry in the RePORTER that covers any year in the period 2015 until 2022; 'current': any entry in the RePORTER that covers 2021 and/or 2022.

Among the 105 other subfields that we had considered a priori as not highly related to biomedicine, most had very low or even zero proportions of their top-cited scientists funded. However, exceptions existed, in particular for family studies, organic chemistry, analytical chemistry, statistics and probability, and nanoscience and nanotechnology (eTable 1 in Supplement 1).

### Difference in Citation Metrics Between Funded and Nonfunded Top-Cited Scientists

Funded scientists had higher citation counts and composite citation indices than nonfunded scientists (Figure, Table 3). The difference in median career-long citations was more substantial when any funding was considered (more than 4222 citations for the 69 highly related fields) and it was smaller when recent funding was considered (more than 3068 citations) and even smaller when current funding was considered (more than 2833 citations). Most of the 69 subfields showed higher citations in funded vs nonfunded scientists, but exceptions occurred (eTable 2 in Supplement 1).

**Figure.**
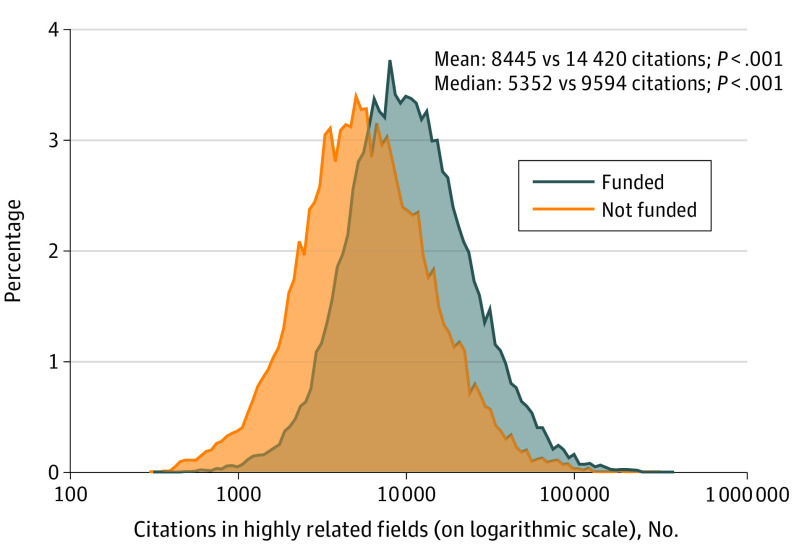
Distribution of Citations (Career-Long) for Top-Cited Scientists in Biomedical Subfields of Science According to Whether They Have Received Federal Biomedical Funding or Not

**Table 3.** Comparison of Citation Counts and of Composite Citation Index Metrics in Funded and Nonfunded Scientists in Scientific Subfields Highly Related to Biomedicine^a^

Top-cited US-based researchers	Funding time^b^	Median (IQR)		*P* value	Median (IQR)		*P* value
		Citations for funded	Citations for nonfunded		Composite index for funded	Composite index for nonfunded	
Career-long impact	Any funding	9594 (5650-17 031)	5352 (3057-9890)	<.001	3.69 (3.5-3.96)	3.51 (3.31-3.73)	<.001
Career-long impact	Recent funding	10 258 (6089-17 936)	7190 (3997-13 310)	<.001	3.69 (3.5-3.94)	3.6 (3.4-3.86)	<.001
Career-long impact	Current funding	10 312 (6183-17 936)	7479 (4165-13 793)	<.001	3.68 (3.5-3.94)	3.61 (3.41-3.87)	<.001
Recent year impact	Any funding	1265 (737-2290)	820 (451-1526)	<.001	3.01 (2.81-3.28)	2.85 (2.65-3.07)	<.001
Recent year impact	Recent funding	1346 (802-2422)	985 (546-1846)	<.001	3 (2.82-3.26)	2.93 (2.72-3.19)	<.001
Recent year impact	Current funding	1376 (817-2459)	1026 (572-1917)	<.001	3 (2.82-3.26)	2.94 (2.73-3.2)	<.001

^a^ Highly related fields (according to the Science-Metrix classification) are 69 subfields, ie, the 60 subfields within the larger fields of biomedical research, clinical medicine, public health and health services, and psychology and cognitive sciences as well as 9 prespecified subfields from other large fields, ie, applied ethics, bioinformatics, biomedical engineering, biotechnology, demography, gender studies, medical informatics, medicinal and biomolecular chemistry, and veterinary sciences.

^b^ Funding time is defined as 'any': any grant entry in RePORTER in 1996 to 2022; 'recent': any entry in the RePORTER that covers any year in the period 2015 until 2022; 'current': any entry in the RePORTER that covers 2021 and/or 2022.

After adjusting for subfield and the publication age of the scientist, the funded scientists still had higher citation metrics than the nonfunded ones. The differences tended to become smaller (eg, more than 2069 citations in career-impact for those with vs those without current funding, and similar pictures emerged also for subfield-specific analyses) (eTable 3 and eTable 4 in Supplement 1).

### Publication Age Subgroups

Percentages of top-cited US biomedical scientists (recent single-year citation impact) with current funding were 31.6% for scientists publishing 20 or fewer years (2191 of 6942). The respective percentages were 22.5% for those publishing 21 to 40 years (4313 of 19 141) and 9.9% for those publishing longer than 40 years (961 of 9704).

## Discussion

This survey study found that biomedical federal funding has offered support as principal investigators to approximately two-thirds of the top-cited biomedical scientists at some point during the last quarter century. However, the rates of support were smaller when only recent funding was considered, and only a small minority of top-cited scientists had current federal biomedical funding as principal investigators. The current funding rates were higher for scientists who were within 2 decades of publishing their first article, but even among them, the large majority did not have current funding. There is very large variability across scientific subfields, with some subfields having had almost ubiquitous funding of their top-cited scientists at least at some point during the last quarter century, and other subfields having negligible funding coverage for their top-cited scientists from biomedical federal agencies. Funded top-cited scientists attract substantially more citations than nonfunded ones, after adjusting for scientific subfield. Citation differences persist also when further adjustment is made for publication age of scientists.

These data highlight the great importance of federal funding for the most impactful researchers in the bibliometrics of the scientific literature. However, they also demonstrate that the large majority of top-cited biomedical scientists do not have current federal funding from biomedical federal agencies as principal investigators. This would suggest the need for further strengthening of the federal research budget. Investment in biomedical science is vital. Economic and improved health outcomes such as longer life expectancy are well-documented benefits of research investment.^14,15,16^ There is concern that many capable scientists do not receive federal funding or receive federal funding for the first time very late in their career, prompting suggestions for funding reform.^17,18,19,20^ Moreover, there is great unevenness across subfields and it is unclear whether this unevenness is justified. It is possible that some subfields are more able to attract funding from other federal (eg, National Science Foundation) and nonfederal sources, such as industry, foundations, institutions, philanthropy, and so forth. However, it is likely that there is genuine imbalance in funding that is associated with the funding agency leadership choices, the ability of some subfields to make stronger claims for the support of their work, the existence (or perceived existence) of major opportunities for discoveries, and the fact that study section members tend to prefer funding work that they feel more familiar with.^5^ The extremely low rates of funding of some subfields focused on psychology, cognitive sciences, gender studies, legal and forensic medicine, and human factors is problematic because these fields may also have difficulty to attract other funding (eg, from industry). What may emerge may be a strengthening of some disciplines, while others (with crucial potential contributions) are abandoned. Fields that receive the majority of funding may not necessarily be the best investments and inertia may delay refocusing of research priorities.^16^ Scientists in underfunded fields often make pleas for more attention to their discipline,^21^ but fragmented efforts may not allow rational, comprehensive reform.

The observed association of higher citation indices with federal funding is congruent with these considerations. Correlations between citation indices and federal funding have also been previously observed in more limited analyses focused on specific specialties (eg, radiology or neurosurgery).^22,23,24^ However, the direction of a causal relationship, if any, in this association in unclear. Bidirectional effects are plausible. More funding could lead to more productivity and citations. Concurrent greater productivity and citations offer ammunition to claim and secure more funding. We should caution that we focused only on the top-cited scientists and there is no guarantee that less-cited scientists show the same difference between funded and nonfunded ones. However, top-cited scientists are the ones whose work is more widely used in the scientific literature, so they represent a critically influential sample. Of course, neither citations nor funding guarantee quality and reproducibility of the work.

### Limitations

Some limitations should be discussed. First, matching of names across the 2 databases is imperfect. We performed in-depth evaluation of random samples of names for validation purposes. These validations suggest that matching errors are fairly small and the same applies for redundancy in the databases (duplicate entries of the same person are extremely uncommon^9^ in the citations database and fairly uncommon in RePORTER). If one were to adjust for the magnitude of the potential matching and duplication errors, the proportion funded would be similar to the crude estimates we have recorded or only slightly larger. Data for women may have the extra problem of changes in name upon change in marital status. However, the vast majority of marriage-related surname changes occur before the age of getting federal funding; therefore, funding and highly cited data sets would contain the same surname for these women. Second, some scientists in the citations database are currently well beyond their prime publishing years (and very few may also have died); therefore, they cannot be considered eligible for current or even recent funding, but the analysis of any funding at any time is not affected by this consideration. Third, recording of funding in RePORTER does not mean that the funding has covered a substantial portion of a researcher’s investigational agenda and effort. Many federal grants offer very limited money and we did not try to analyze amounts of funding as this would be precarious to do across fields with very different cost requirements and over decades. Fourth, RePORTER catalogs the principal investigators of extramural grants (including multiple principal investigators). Some researchers may have received funding through other roles (coinvestigators, subaward, intramural NIH, and contractors) and not be retrievable in RePORTER. Fifth, some top-cited biomedical researchers may have received other federal funding (eg, National Science Foundation for psychology disciplines or Department of Defense) not included in RePORTER. Sixth, the citations database has some errors in accuracy and precision, but overall these errors have been documented to be small in previous validation exercises; we refer to previous work^9,10,11^ for more details. Although other scientists who perform important work may not be among those included in the top-cited lists, the included scientists clearly have a large impact in the literature—even if the true deep scientific quality of this impact cannot be certain and is often even intangible long after scientific work is published.

## Conclusions

Both funding success and citation metrics have major limitations as measures of impact, novelty, or good scientific work. However, they are widely used in academia and beyond and can shape, support, or harm careers and shape science and allocation of precious resources. Our analysis offers insights about the possibly bidirectional association of funding and citation impact and provides a map of US science across 2 important dimensions. The findings that only a small minority of top-cited scientists had current federal biomedical funding calls for thoughtful inclusive redesign of future funding agendas.

## Notes

Supplement 1. **eAppendix. **Supplementary Text **eTable 1. **Percentage Funded by Field **eTable 2. **Citation Counts and Composite Citation Indices for Each Subfield (Ordered by Percentage Funded) **eTable 3. **Linear Regressions **eTable 4. **Mixed-Effects Linear Regressions

Supplement 2. Data Sharing Statement
