# Supplementary material for: Federal Funding and Citation Metrics of US Biomedical Researchers, 1996 to 2022
Source: JAMA Netw Open. 2022 Dec 7;5(12):e2245590. doi: 10.1001/jamanetworkopen.2022.45590 (PMC9856501; doi:10.1001/jamanetworkopen.2022.45590)
Supplement: Supplement 1. — eAppendix. Supplementary Text eTable 1. Percentage Funded by Field eTable 2. Citation Counts and Composite Citation Indices for Each Subfield (Ordered by Percentage Funded) eTable 3. Linear Regressions eTable 4. Mixed-Effects Linear Regressions [file jamanetwopen-e2245590-s001.pdf]

Supplemental Online Content

Ioannidis JPA, Hozo I, Djulbegovic B. Federal funding and citation metrics of US biomedical researchers, 1996 to 2022. *JAMA Netw Open*. 2022;5(12):e2245590. doi:10.1001/jamanetworkopen.2022.45590

eAppendix. Supplementary Text

eTable 1. Percentage Funded by Field

eTable 2. Citation Counts and Composite Citation Indices for Each Subfield (Ordered by Percentage Funded)

eTable 3. Linear Regressions

eTable 4. Mixed-Effects Linear Regressions

This supplemental material has been provided by the authors to give readers additional information about their work.

© 2022 Ioannidis JPA et al. *JAMA Network Open.*

eAppendix. SUPPLEMENTARY TEXT

General comments: Funding time is defined as 'any': any grant entry in Reporter; 'recent': any entry in the Reporter that covers any year in the period 2015 until 2022; 'current': any entry in the Reporter that covers 2021 and/or 2022. Highly related fields (according to the Science-Metrix classification) are 69 subfields, i.e. the 60 subfields within the larger fields of Biomedical Research, Clinical Medicine, Public Health and Health Services, and Psychology and Cognitive Sciences as well as 9 prespecified subfields from other large fields, i.e. Applied Ethics, Bioinformatics, Biomedical Engineering, Biotechnology, Demography, Gender Studies, Medical Informatics, Medicinal and Biomolecular Chemistry, and Veterinary Sciences. The 69 subfields are listed as follows (please note that the names of the subfields are those given by Science-Metrix and it is an effort to capture the core interest of each subfield, but this may not always be fully inclusive with such concrete terms, e.g. Developmental Biology includes a lot of genomics and other omics):

Developmental Biology

Substance Abuse

Immunology

Geriatrics

Biochemistry & Molecular Biology

Endocrinology & Metabolism

Gerontology

Virology

Neurology & Neurosurgery

Genetics & Heredity

Bioinformatics

Psychiatry

Oncology & Carcinogenesis

Public Health

Medical Informatics

Demography

Physiology

Epidemiology

Biomedical Engineering

Developmental & Child Psychology

© 2022 Ioannidis JPA et al. *JAMA Network Open.*

Arthritis & Rheumatology

Allergy

Biophysics

Respiratory System

Experimental Psychology

Gastroenterology & Hepatology

Cardiovascular System & Hematology

Health Policy & Services

Urology & Nephrology

Speech-Language Pathology & Audiology

Clinical Psychology

Pediatrics

Microbiology

Applied Ethics

Nutrition & Dietetics

Ophthalmology & Optometry

Environmental & Occupational Health

Toxicology

Nursing

Obstetrics & Reproductive Medicine

Emergency & Critical Care Medicine

Rehabilitation

Pharmacology & Pharmacy

Behavioral Science & Comparative Psychology

Nuclear Medicine & Medical Imaging

Otorhinolaryngology

Dentistry

Tropical Medicine

Mycology & Parasitology

© 2022 Ioannidis JPA et al. *JAMA Network Open.*

Medicinal & Biomolecular Chemistry

Anesthesiology

Dermatology & Venereal Diseases

Biotechnology

Social Psychology

Surgery

General & Internal Medicine

Complementary & Alternative Medicine

Sport Sciences

Microscopy

Pathology

Orthopedics

General Clinical Medicine

Veterinary Sciences

Human Factors

Anatomy & Morphology

Gender Studies

General Psychology & Cognitive Sciences

Psychoanalysis

Legal & Forensic Medicine

© 2022 Ioannidis JPA et al. *JAMA Network Open.*

SUPPLEMENTARY TABLES

eTable1. Percentage Funded by Field

eTable 1.1: Percentage funded by field for Career: Any funding time

Top-cited US-based researchers: Subfield	Funding time	Total	Funded (%)
Career: Developmental Biology	Any	1769	1566(89%)
Career: Substance Abuse	Any	277	240(87%)
Career: Immunology	Any	1474	1247(85%)
Career: Geriatrics	Any	97	82(85%)
Career: Biochemistry & Molecular Biology	Any	2173	1756(81%)
Career: Endocrinology & Metabolism	Any	993	800(81%)
Career: Gerontology	Any	126	101(80%)
Career: Virology	Any	726	578(80%)
Career: Neurology & Neurosurgery	Any	3337	2635(79%)
Career: Genetics & Heredity	Any	353	278(79%)
Career: Bioinformatics	Any	196	150(77%)
Career: Psychiatry	Any	927	707(76%)
Career: Oncology & Carcinogenesis	Any	2894	2144(74%)
Career: Public Health	Any	692	507(73%)
Career: Medical Informatics	Any	126	90(71%)
Career: Demography	Any	42	30(71%)
Career: Physiology	Any	247	176(71%)
Career: Epidemiology	Any	156	111(71%)
Career: Biomedical Engineering	Any	599	425(71%)
Career: Developmental & Child Psychology	Any	459	323(70%)
Career: Arthritis & Rheumatology	Any	229	160(70%)
Career: Allergy	Any	142	98(69%)
Career: Biophysics	Any	186	128(69%)
Career: Respiratory System	Any	591	389(66%)
Career: Experimental Psychology	Any	633	412(65%)
Career: Gastroenterology & Hepatology	Any	700	454(65%)
Career: Cardiovascular System & Hematology	Any	1876	1207(64%)
Career: Health Policy & Services	Any	214	135(63%)
Career: Urology & Nephrology	Any	814	510(63%)
Career: Speech-Language Pathology & Audiology	Any	111	68(61%)
Career: Clinical Psychology	Any	215	129(60%)
Career: Pediatrics	Any	752	448(60%)
Career: Microbiology	Any	1471	876(60%)
Career: Family Studies	Any	56	33(59%)
Career: Applied Ethics	Any	71	41(58%)
Career: Nutrition & Dietetics	Any	367	211(57%)
Career: Ophthalmology & Optometry	Any	771	443(57%)

© 2022 Ioannidis JPA et al. *JAMA Network Open.*

**Career: Organic Chemistry**	**Any**	725	413(57%)
**Career: Environmental & Occupational Health**	**Any**	123	70(57%)
**Career: Toxicology**	**Any**	541	301(56%)
**Career: Nursing**	**Any**	919	502(55%)
**Career: Obstetrics & Reproductive Medicine**	**Any**	734	400(54%)
**Career: Emergency & Critical Care Medicine**	**Any**	339	181(53%)
**Career: Rehabilitation**	**Any**	230	122(53%)
**Career: Pharmacology & Pharmacy**	**Any**	833	425(51%)
**Career: Behavioral Science & Comparative Psychology**	**Any**	207	103(50%)
**Career: Analytical Chemistry**	**Any**	527	256(49%)
**Career: Nuclear Medicine & Medical Imaging**	**Any**	1184	562(47%)
**Career: Otorhinolaryngology**	**Any**	522	232(44%)
**Career: Dentistry**	**Any**	564	250(44%)
**Career: Tropical Medicine**	**Any**	217	95(44%)
**Career: Mycology & Parasitology**	**Any**	146	63(43%)
**Career: Statistics & Probability**	**Any**	249	107(43%)
**Career: Medicinal & Biomolecular Chemistry**	**Any**	652	280(43%)
**Career: Anesthesiology**	**Any**	435	186(43%)
**Career: Dermatology & Venereal Diseases**	**Any**	381	160(42%)
**Career: Biotechnology**	**Any**	270	110(41%)
**Career: Social Psychology**	**Any**	482	196(41%)
**Career: Surgery**	**Any**	1261	504(40%)
**Career: General & Internal Medicine**	**Any**	1574	624(40%)
**Career: Complementary & Alternative Medicine**	**Any**	81	32(40%)
**Career: Sport Sciences**	**Any**	186	68(37%)
**Career: Acoustics**	**Any**	310	113(36%)
**Career: History of Social Sciences**	**Any**	23	8(35%)
**Career: Nanoscience & Nanotechnology**	**Any**	488	169(35%)
**Career: General Chemistry**	**Any**	490	167(34%)
**Career: Microscopy**	**Any**	30	10(33%)
**Career: Criminology**	**Any**	153	47(31%)
**Career: Sociology**	**Any**	157	48(31%)
**Career: Optics**	**Any**	484	146(30%)
**Career: Pathology**	**Any**	293	87(30%)
**Career: Social Work**	**Any**	88	26(30%)
**Career: Evolutionary Biology**	**Any**	398	117(29%)
**Career: Orthopedics**	**Any**	777	214(28%)
**Career: Social Sciences Methods**	**Any**	77	21(27%)
**Career: Chemical Physics**	**Any**	890	238(27%)
**Career: General Clinical Medicine**	**Any**	128	31(24%)
**Career: Plant Biology & Botany**	**Any**	812	193(24%)
**Career: Inorganic & Nuclear Chemistry**	**Any**	318	75(24%)
**Career: Veterinary Sciences**	**Any**	558	131(23%)
**Career: Environmental Sciences**	**Any**	422	88(21%)
**Career: Human Factors**	**Any**	166	31(19%)
**Career: Ornithology**	**Any**	33	6(18%)
**Career: Urban & Regional Planning**	**Any**	61	11(18%)
**Career: Economics**	**Any**	453	81(18%)

© 2022 Ioannidis JPA et al. *JAMA Network Open.*

**Career: Artificial Intelligence & Image Processing**	**Any**	1439	249(17%)
**Career: Polymers**	**Any**	531	89(17%)
**Career: Economic Theory**	**Any**	18	3(17%)
**Career: Development Studies**	**Any**	24	4(17%)
**Career: Anatomy & Morphology**	**Any**	54	9(17%)
**Career: Distributed Computing**	**Any**	139	23(17%)
**Career: Entomology**	**Any**	294	46(16%)
**Career: Gender Studies**	**Any**	13	2(15%)
**Career: History of Science, Technology & Medicine**	**Any**	20	3(15%)
**Career: Software Engineering**	**Any**	228	34(15%)
**Career: Education**	**Any**	732	108(15%)
**Career: Food Science**	**Any**	268	39(15%)
**Career: Industrial Engineering & Automation**	**Any**	628	90(14%)
**Career: Logistics & Transportation**	**Any**	183	26(14%)
**Career: Geography**	**Any**	92	13(14%)
**Career: Sport, Leisure & Tourism**	**Any**	46	6(13%)
**Career: Numerical & Computational Mathematics**	**Any**	120	15(13%)
**Career: Communication & Media Studies**	**Any**	154	19(12%)
**Career: General Psychology & Cognitive Sciences**	**Any**	65	8(12%)
**Career: Fluids & Plasmas**	**Any**	372	45(12%)
**Career: Information & Library Sciences**	**Any**	117	14(12%)
**Career: Dairy & Animal Science**	**Any**	454	54(12%)
**Career: Optoelectronics & Photonics**	**Any**	933	108(12%)
**Career: Anthropology**	**Any**	123	14(11%)
**Career: Religions & Theology**	**Any**	90	10(11%)
**Career: Drama & Theater**	**Any**	9	1(11%)
**Career: Design Practice & Management**	**Any**	100	11(11%)
**Career: Environmental Engineering**	**Any**	410	45(11%)
**Career: Chemical Engineering**	**Any**	330	36(11%)
**Career: Applied Mathematics**	**Any**	138	15(11%)
**Career: Marketing**	**Any**	129	14(11%)
**Career: Zoology**	**Any**	99	10(10%)
**Career: Architecture**	**Any**	10	1(10%)
**Career: Ecology**	**Any**	743	72(9.7%)
**Career: Marine Biology & Hydrobiology**	**Any**	331	32(9.7%)
**Career: Networking & Telecommunications**	**Any**	1476	140(9.5%)
**Career: Computer Hardware & Architecture**	**Any**	227	21(9.3%)
**Career: Electrical & Electronic Engineering**	**Any**	501	46(9.2%)
**Career: Geological & Geomatics Engineering**	**Any**	357	32(9%)
**Career: Information Systems**	**Any**	209	18(8.6%)
**Career: Law**	**Any**	106	9(8.5%)
**Career: Mathematical Physics**	**Any**	24	2(8.3%)
**Career: Strategic, Defence & Security Studies**	**Any**	182	15(8.2%)
**Career: Agricultural Economics & Policy**	**Any**	73	6(8.2%)
**Career: General Physics**	**Any**	488	40(8.2%)
**Career: Languages & Linguistics**	**Any**	110	9(8.2%)
**Career: Mining & Metallurgy**	**Any**	62	5(8.1%)
**Career: Psychoanalysis**	**Any**	50	4(8%)

© 2022 Ioannidis JPA et al. *JAMA Network Open.*

**Career: Political Science & Public Administration**	**Any**	228	18(7.9%)
**Career: Building & Construction**	**Any**	128	10(7.8%)
**Career: Oceanography**	**Any**	185	14(7.6%)
**Career: Literary Studies**	**Any**	159	12(7.5%)
**Career: Cultural Studies**	**Any**	54	4(7.4%)
**Career: Materials**	**Any**	1363	99(7.3%)
**Career: Applied Physics**	**Any**	1918	138(7.2%)
**Career: Econometrics**	**Any**	42	3(7.1%)
**Career: Mechanical Engineering & Transports**	**Any**	706	50(7.1%)
**Career: Operations Research**	**Any**	205	14(6.8%)
**Career: Finance**	**Any**	132	9(6.8%)
**Career: Business & Management**	**Any**	526	34(6.5%)
**Career: Meteorology & Atmospheric Sciences**	**Any**	923	59(6.4%)
**Career: General Mathematics**	**Any**	502	32(6.4%)
**Career: Computation Theory & Mathematics**	**Any**	177	11(6.2%)
**Career: Fisheries**	**Any**	227	14(6.2%)
**Career: Philosophy**	**Any**	130	8(6.2%)
**Career: Archaeology**	**Any**	114	7(6.1%)
**Career: Paleontology**	**Any**	168	10(6%)
**Career: Energy**	**Any**	1212	72(5.9%)
**Career: Agronomy & Agriculture**	**Any**	456	27(5.9%)
**Career: Aerospace & Aeronautics**	**Any**	754	44(5.8%)
**Career: Astronomy & Astrophysics**	**Any**	789	44(5.6%)
**Career: Classics**	**Any**	18	1(5.6%)
**Career: Geology**	**Any**	92	5(5.4%)
**Career: Forestry**	**Any**	209	11(5.3%)
**Career: Geochemistry & Geophysics**	**Any**	843	41(4.9%)
**Career: Nuclear & Particle Physics**	**Any**	1030	48(4.7%)
**Career: History**	**Any**	111	5(4.5%)
**Career: International Relations**	**Any**	68	3(4.4%)
**Career: Physical Chemistry**	**Any**	114	5(4.4%)
**Career: Science Studies**	**Any**	26	1(3.8%)
**Career: Civil Engineering**	**Any**	275	10(3.6%)
**Career: Legal & Forensic Medicine**	**Any**	66	2(3%)
**Career: Music**	**Any**	42	1(2.4%)
**Career: Accounting**	**Any**	42	1(2.4%)
**Career: Horticulture**	**Any**	75	1(1.3%)
**Career: Industrial Relations**	**Any**	8	0(0%)
**Career: Folklore**	**Any**	6	0(0%)
**Career: Automobile Design & Engineering**	**Any**	7	0(0%)
**Career: Art Practice, History & Theory**	**Any**	32	0(0%)

eTable 1.2: Percentage funded by field for Career: Recent funding time

**Top-cited US-based researchers: Subfield**	**Funding time**	**Total**	**Funded (%)**
**Career: Developmental Biology**	**Recent**	1769	744(42%)

© 2022 Ioannidis JPA et al. *JAMA Network Open.*

**Career: Bioinformatics**	**Recent**	196	81(41%)
**Career: Geriatrics**	**Recent**	97	39(40%)
**Career: Substance Abuse**	**Recent**	277	100(36%)
**Career: Biomedical Engineering**	**Recent**	599	205(34%)
**Career: Medical Informatics**	**Recent**	126	43(34%)
**Career: Immunology**	**Recent**	1474	502(34%)
**Career: Epidemiology**	**Recent**	156	53(34%)
**Career: Virology**	**Recent**	726	246(34%)
**Career: Neurology & Neurosurgery**	**Recent**	3337	1060(32%)
**Career: Public Health**	**Recent**	692	212(31%)
**Career: Gerontology**	**Recent**	126	38(30%)
**Career: Oncology & Carcinogenesis**	**Recent**	2894	861(30%)
**Career: Genetics & Heredity**	**Recent**	353	102(29%)
**Career: Demography**	**Recent**	42	11(26%)
**Career: Health Policy & Services**	**Recent**	214	56(26%)
**Career: Developmental & Child Psychology**	**Recent**	459	117(25%)
**Career: Applied Ethics**	**Recent**	71	18(25%)
**Career: Gastroenterology & Hepatology**	**Recent**	700	176(25%)
**Career: Endocrinology & Metabolism**	**Recent**	993	246(25%)
**Career: Biophysics**	**Recent**	186	46(25%)
**Career: Psychiatry**	**Recent**	927	229(25%)
**Career: Arthritis & Rheumatology**	**Recent**	229	55(24%)
**Career: Emergency & Critical Care Medicine**	**Recent**	339	81(24%)
**Career: Microbiology**	**Recent**	1471	343(23%)
**Career: Allergy**	**Recent**	142	33(23%)
**Career: Respiratory System**	**Recent**	591	136(23%)
**Career: Clinical Psychology**	**Recent**	215	49(23%)
**Career: Cardiovascular System & Hematology**	**Recent**	1876	423(23%)
**Career: Biochemistry & Molecular Biology**	**Recent**	2173	483(22%)
**Career: Physiology**	**Recent**	247	54(22%)
**Career: Ophthalmology & Optometry**	**Recent**	771	164(21%)
**Career: Analytical Chemistry**	**Recent**	527	112(21%)
**Career: Urology & Nephrology**	**Recent**	814	166(20%)
**Career: Rehabilitation**	**Recent**	230	46(20%)
**Career: Microscopy**	**Recent**	30	6(20%)
**Career: Tropical Medicine**	**Recent**	217	43(20%)
**Career: Statistics & Probability**	**Recent**	249	49(20%)
**Career: Family Studies**	**Recent**	56	11(20%)
**Career: Toxicology**	**Recent**	541	106(20%)
**Career: Nutrition & Dietetics**	**Recent**	367	71(19%)
**Career: Medicinal & Biomolecular Chemistry**	**Recent**	652	126(19%)
**Career: Nuclear Medicine & Medical Imaging**	**Recent**	1184	228(19%)
**Career: Nanoscience & Nanotechnology**	**Recent**	488	91(19%)
**Career: Pediatrics**	**Recent**	752	140(19%)
**Career: Pharmacology & Pharmacy**	**Recent**	833	151(18%)
**Career: Optics**	**Recent**	484	87(18%)
**Career: Anesthesiology**	**Recent**	435	78(18%)
**Career: Obstetrics & Reproductive Medicine**	**Recent**	734	130(18%)

© 2022 Ioannidis JPA et al. *JAMA Network Open.*

**Career: Experimental Psychology**	**Recent**	633	111(18%)
**Career: Speech-Language Pathology & Audiology**	**Recent**	111	19(17%)
**Career: Biotechnology**	**Recent**	270	45(17%)
**Career: Organic Chemistry**	**Recent**	725	116(16%)
**Career: Nursing**	**Recent**	919	147(16%)
**Career: Environmental & Occupational Health**	**Recent**	123	19(15%)
**Career: Behavioral Science & Comparative Psychology**	**Recent**	207	27(13%)
**Career: Mycology & Parasitology**	**Recent**	146	19(13%)
**Career: General & Internal Medicine**	**Recent**	1574	204(13%)
**Career: Otorhinolaryngology**	**Recent**	522	67(13%)
**Career: Sociology**	**Recent**	157	20(13%)
**Career: Surgery**	**Recent**	1261	159(13%)
**Career: Development Studies**	**Recent**	24	3(13%)
**Career: Drama & Theater**	**Recent**	9	1(11%)
**Career: General Chemistry**	**Recent**	490	54(11%)
**Career: Dermatology & Venereal Diseases**	**Recent**	381	41(11%)
**Career: Acoustics**	**Recent**	310	33(11%)
**Career: Criminology**	**Recent**	153	16(10%)
**Career: Evolutionary Biology**	**Recent**	398	41(10%)
**Career: Orthopedics**	**Recent**	777	79(10%)
**Career: Environmental Sciences**	**Recent**	422	41(9.7%)
**Career: Distributed Computing**	**Recent**	139	13(9.4%)
**Career: Social Work**	**Recent**	88	8(9.1%)
**Career: Social Sciences Methods**	**Recent**	77	7(9.1%)
**Career: Artificial Intelligence & Image Processing**	**Recent**	1439	128(8.9%)
**Career: Complementary & Alternative Medicine**	**Recent**	81	7(8.6%)
**Career: Dentistry**	**Recent**	564	45(8%)
**Career: Veterinary Sciences**	**Recent**	558	44(7.9%)
**Career: Pathology**	**Recent**	293	23(7.8%)
**Career: Plant Biology & Botany**	**Recent**	812	63(7.8%)
**Career: Gender Studies**	**Recent**	13	1(7.7%)
**Career: Social Psychology**	**Recent**	482	37(7.7%)
**Career: Geography**	**Recent**	92	7(7.6%)
**Career: Sport Sciences**	**Recent**	186	14(7.5%)
**Career: Industrial Engineering & Automation**	**Recent**	628	46(7.3%)
**Career: Chemical Physics**	**Recent**	890	65(7.3%)
**Career: Urban & Regional Planning**	**Recent**	61	4(6.6%)
**Career: Sport, Leisure & Tourism**	**Recent**	46	3(6.5%)
**Career: Entomology**	**Recent**	294	18(6.1%)
**Career: Human Factors**	**Recent**	166	10(6%)
**Career: Information & Library Sciences**	**Recent**	117	7(6%)
**Career: Numerical & Computational Mathematics**	**Recent**	120	7(5.8%)
**Career: Economics**	**Recent**	453	26(5.7%)
**Career: Economic Theory**	**Recent**	18	1(5.6%)
**Career: Classics**	**Recent**	18	1(5.6%)
**Career: Anatomy & Morphology**	**Recent**	54	3(5.6%)
**Career: General Clinical Medicine**	**Recent**	128	7(5.5%)
**Career: Logistics & Transportation**	**Recent**	183	10(5.5%)

© 2022 Ioannidis JPA et al. *JAMA Network Open.*

**Career: Computer Hardware & Architecture**	**Recent**	227	12(5.3%)
**Career: Polymers**	**Recent**	531	28(5.3%)
**Career: Communication & Media Studies**	**Recent**	154	8(5.2%)
**Career: Fluids & Plasmas**	**Recent**	372	19(5.1%)
**Career: Software Engineering**	**Recent**	228	11(4.8%)
**Career: Electrical & Electronic Engineering**	**Recent**	501	24(4.8%)
**Career: Optoelectronics & Photonics**	**Recent**	933	44(4.7%)
**Career: Marketing**	**Recent**	129	6(4.7%)
**Career: Languages & Linguistics**	**Recent**	110	5(4.5%)
**Career: Education**	**Recent**	732	33(4.5%)
**Career: Networking & Telecommunications**	**Recent**	1476	66(4.5%)
**Career: Religions & Theology**	**Recent**	90	4(4.4%)
**Career: Archaeology**	**Recent**	114	5(4.4%)
**Career: History of Social Sciences**	**Recent**	23	1(4.3%)
**Career: Applied Mathematics**	**Recent**	138	6(4.3%)
**Career: Mathematical Physics**	**Recent**	24	1(4.2%)
**Career: Agricultural Economics & Policy**	**Recent**	73	3(4.1%)
**Career: Food Science**	**Recent**	268	11(4.1%)
**Career: Anthropology**	**Recent**	123	5(4.1%)
**Career: Science Studies**	**Recent**	26	1(3.8%)
**Career: Information Systems**	**Recent**	209	8(3.8%)
**Career: Finance**	**Recent**	132	5(3.8%)
**Career: Cultural Studies**	**Recent**	54	2(3.7%)
**Career: General Physics**	**Recent**	488	18(3.7%)
**Career: Chemical Engineering**	**Recent**	330	12(3.6%)
**Career: Mechanical Engineering & Transports**	**Recent**	706	25(3.5%)
**Career: Ecology**	**Recent**	743	26(3.5%)
**Career: Operations Research**	**Recent**	205	7(3.4%)
**Career: Dairy & Animal Science**	**Recent**	454	15(3.3%)
**Career: Mining & Metallurgy**	**Recent**	62	2(3.2%)
**Career: Building & Construction**	**Recent**	128	4(3.1%)
**Career: Philosophy**	**Recent**	130	4(3.1%)
**Career: Political Science & Public Administration**	**Recent**	228	7(3.1%)
**Career: Zoology**	**Recent**	99	3(3%)
**Career: Ornithology**	**Recent**	33	1(3%)
**Career: Design Practice & Management**	**Recent**	100	3(3%)
**Career: Materials**	**Recent**	1363	40(2.9%)
**Career: Environmental Engineering**	**Recent**	410	12(2.9%)
**Career: Inorganic & Nuclear Chemistry**	**Recent**	318	9(2.8%)
**Career: Geological & Geomatics Engineering**	**Recent**	357	10(2.8%)
**Career: Oceanography**	**Recent**	185	5(2.7%)
**Career: Literary Studies**	**Recent**	159	4(2.5%)
**Career: Meteorology & Atmospheric Sciences**	**Recent**	923	23(2.5%)
**Career: Applied Physics**	**Recent**	1918	46(2.4%)
**Career: Econometrics**	**Recent**	42	1(2.4%)
**Career: Business & Management**	**Recent**	526	12(2.3%)
**Career: Computation Theory & Mathematics**	**Recent**	177	4(2.3%)
**Career: Aerospace & Aeronautics**	**Recent**	754	17(2.3%)

© 2022 Ioannidis JPA et al. *JAMA Network Open.*

**Career: Strategic, Defence & Security Studies**	**Recent**	182	4(2.2%)
**Career: General Mathematics**	**Recent**	502	11(2.2%)
**Career: Civil Engineering**	**Recent**	275	6(2.2%)
**Career: Geology**	**Recent**	92	2(2.2%)
**Career: Energy**	**Recent**	1212	26(2.1%)
**Career: Psychoanalysis**	**Recent**	50	1(2%)
**Career: Law**	**Recent**	106	2(1.9%)
**Career: Marine Biology & Hydrobiology**	**Recent**	331	6(1.8%)
**Career: Astronomy & Astrophysics**	**Recent**	789	14(1.8%)
**Career: Fisheries**	**Recent**	227	4(1.8%)
**Career: Legal & Forensic Medicine**	**Recent**	66	1(1.5%)
**Career: International Relations**	**Recent**	68	1(1.5%)
**Career: Nuclear & Particle Physics**	**Recent**	1030	13(1.3%)
**Career: Geochemistry & Geophysics**	**Recent**	843	10(1.2%)
**Career: Forestry**	**Recent**	209	2(.96%)
**Career: Physical Chemistry**	**Recent**	114	1(.88%)
**Career: Agronomy & Agriculture**	**Recent**	456	4(.88%)
**Career: Paleontology**	**Recent**	168	0(0%)
**Career: Music**	**Recent**	42	0(0%)
**Career: Industrial Relations**	**Recent**	8	0(0%)
**Career: Horticulture**	**Recent**	75	0(0%)
**Career: History of Science, Technology & Medicine**	**Recent**	20	0(0%)
**Career: History**	**Recent**	111	0(0%)
**Career: General Psychology & Cognitive Sciences**	**Recent**	65	0(0%)
**Career: Folklore**	**Recent**	6	0(0%)
**Career: Automobile Design & Engineering**	**Recent**	7	0(0%)
**Career: Art Practice, History & Theory**	**Recent**	32	0(0%)
**Career: Architecture**	**Recent**	10	0(0%)
**Career: Accounting**	**Recent**	42	0(0%)

© 2022 Ioannidis JPA et al. *JAMA Network Open.*

eTable 1.3: Percentage funded by field for Career: Current funding time

Top-cited US-based researchers: Subfield	Funding time	Total	Funded (%)
Career: Geriatrics	Current	97	30(31%)
Career: Bioinformatics	Current	196	58(30%)
Career: Developmental Biology	Current	1769	510(29%)
Career: Substance Abuse	Current	277	64(23%)
Career: Medical Informatics	Current	126	29(23%)
Career: Virology	Current	726	159(22%)
Career: Biomedical Engineering	Current	599	131(22%)
Career: Neurology & Neurosurgery	Current	3337	692(21%)
Career: Immunology	Current	1474	300(20%)
Career: Gerontology	Current	126	25(20%)
Career: Applied Ethics	Current	71	14(20%)
Career: Public Health	Current	692	134(19%)
Career: Demography	Current	42	8(19%)
Career: Oncology & Carcinogenesis	Current	2894	517(18%)
Career: Gastroenterology & Hepatology	Current	700	116(17%)
Career: Allergy	Current	142	23(16%)
Career: Biophysics	Current	186	30(16%)
Career: Developmental & Child Psychology	Current	459	73(16%)
Career: Health Policy & Services	Current	214	34(16%)
Career: Physiology	Current	247	39(16%)
Career: Arthritis & Rheumatology	Current	229	36(16%)
Career: Epidemiology	Current	156	24(15%)
Career: Psychiatry	Current	927	142(15%)
Career: Genetics & Heredity	Current	353	54(15%)
Career: Emergency & Critical Care Medicine	Current	339	51(15%)
Career: Endocrinology & Metabolism	Current	993	148(15%)
Career: Statistics & Probability	Current	249	36(14%)
Career: Ophthalmology & Optometry	Current	771	110(14%)
Career: Microbiology	Current	1471	209(14%)
Career: Cardiovascular System & Hematology	Current	1876	260(14%)
Career: Respiratory System	Current	591	79(13%)
Career: Clinical Psychology	Current	215	28(13%)
Career: Nuclear Medicine & Medical Imaging	Current	1184	153(13%)
Career: Analytical Chemistry	Current	527	68(13%)
Career: Medicinal & Biomolecular Chemistry	Current	652	81(12%)
Career: Nanoscience & Nanotechnology	Current	488	60(12%)
Career: Urology & Nephrology	Current	814	100(12%)
Career: Optics	Current	484	58(12%)
Career: Biochemistry & Molecular Biology	Current	2173	250(12%)
Career: Nutrition & Dietetics	Current	367	42(11%)
Career: Experimental Psychology	Current	633	72(11%)
Career: Rehabilitation	Current	230	26(11%)
Career: Toxicology	Current	541	61(11%)

© 2022 Ioannidis JPA et al. *JAMA Network Open.*

**Career: Drama & Theater**	**Current**	9	1(11%)
**Career: Obstetrics & Reproductive Medicine**	**Current**	734	80(11%)
**Career: Pediatrics**	**Current**	752	80(11%)
**Career: Anesthesiology**	**Current**	435	46(11%)
**Career: Pharmacology & Pharmacy**	**Current**	833	83(10%)
**Career: Speech-Language Pathology & Audiology**	**Current**	111	11(9.9%)
**Career: Organic Chemistry**	**Current**	725	71(9.8%)
**Career: Environmental & Occupational Health**	**Current**	123	12(9.8%)
**Career: Family Studies**	**Current**	56	5(8.9%)
**Career: Development Studies**	**Current**	24	2(8.3%)
**Career: Mycology & Parasitology**	**Current**	146	12(8.2%)
**Career: Nursing**	**Current**	919	73(7.9%)
**Career: Surgery**	**Current**	1261	99(7.9%)
**Career: Tropical Medicine**	**Current**	217	17(7.8%)
**Career: General Chemistry**	**Current**	490	38(7.8%)
**Career: Complementary & Alternative Medicine**	**Current**	81	6(7.4%)
**Career: General & Internal Medicine**	**Current**	1574	112(7.1%)
**Career: Biotechnology**	**Current**	270	19(7%)
**Career: Microscopy**	**Current**	30	2(6.7%)
**Career: Acoustics**	**Current**	310	20(6.5%)
**Career: Sociology**	**Current**	157	10(6.4%)
**Career: Otorhinolaryngology**	**Current**	522	33(6.3%)
**Career: Behavioral Science & Comparative Psychology**	**Current**	207	13(6.3%)
**Career: Orthopedics**	**Current**	777	46(5.9%)
**Career: Sport Sciences**	**Current**	186	11(5.9%)
**Career: Social Work**	**Current**	88	5(5.7%)
**Career: Economic Theory**	**Current**	18	1(5.6%)
**Career: Evolutionary Biology**	**Current**	398	22(5.5%)
**Career: Dermatology & Venereal Diseases**	**Current**	381	21(5.5%)
**Career: Artificial Intelligence & Image Processing**	**Current**	1439	79(5.5%)
**Career: Environmental Sciences**	**Current**	422	23(5.5%)
**Career: Plant Biology & Botany**	**Current**	812	42(5.2%)
**Career: Pathology**	**Current**	293	15(5.1%)
**Career: Chemical Physics**	**Current**	890	43(4.8%)
**Career: Industrial Engineering & Automation**	**Current**	628	30(4.8%)
**Career: Entomology**	**Current**	294	14(4.8%)
**Career: Criminology**	**Current**	153	7(4.6%)
**Career: Religions & Theology**	**Current**	90	4(4.4%)
**Career: Sport, Leisure & Tourism**	**Current**	46	2(4.3%)
**Career: Distributed Computing**	**Current**	139	6(4.3%)
**Career: Dentistry**	**Current**	564	24(4.3%)
**Career: Social Sciences Methods**	**Current**	77	3(3.9%)
**Career: Science Studies**	**Current**	26	1(3.8%)
**Career: Logistics & Transportation**	**Current**	183	7(3.8%)
**Career: Veterinary Sciences**	**Current**	558	21(3.8%)
**Career: Social Psychology**	**Current**	482	18(3.7%)
**Career: Anatomy & Morphology**	**Current**	54	2(3.7%)
**Career: Applied Mathematics**	**Current**	138	5(3.6%)

**Career: Polymers**	**Current**	531	19(3.6%)
**Career: Information & Library Sciences**	**Current**	117	4(3.4%)
**Career: Communication & Media Studies**	**Current**	154	5(3.2%)
**Career: Mining & Metallurgy**	**Current**	62	2(3.2%)
**Career: General Clinical Medicine**	**Current**	128	4(3.1%)
**Career: Marketing**	**Current**	129	4(3.1%)
**Career: Economics**	**Current**	453	14(3.1%)
**Career: Software Engineering**	**Current**	228	7(3.1%)
**Career: Ornithology**	**Current**	33	1(3%)
**Career: Finance**	**Current**	132	4(3%)
**Career: Human Factors**	**Current**	166	5(3%)
**Career: Agricultural Economics & Policy**	**Current**	73	2(2.7%)
**Career: Computer Hardware & Architecture**	**Current**	227	6(2.6%)
**Career: Networking & Telecommunications**	**Current**	1476	39(2.6%)
**Career: Political Science & Public Administration**	**Current**	228	6(2.6%)
**Career: Food Science**	**Current**	268	7(2.6%)
**Career: Education**	**Current**	732	19(2.6%)
**Career: Dairy & Animal Science**	**Current**	454	11(2.4%)
**Career: Econometrics**	**Current**	42	1(2.4%)
**Career: Mechanical Engineering & Transports**	**Current**	706	16(2.3%)
**Career: Strategic, Defence & Security Studies**	**Current**	182	4(2.2%)
**Career: Electrical & Electronic Engineering**	**Current**	501	11(2.2%)
**Career: Geography**	**Current**	92	2(2.2%)
**Career: Chemical Engineering**	**Current**	330	7(2.1%)
**Career: General Physics**	**Current**	488	10(2%)
**Career: Optoelectronics & Photonics**	**Current**	933	19(2%)
**Career: Psychoanalysis**	**Current**	50	1(2%)
**Career: Design Practice & Management**	**Current**	100	2(2%)
**Career: Operations Research**	**Current**	205	4(2%)
**Career: Environmental Engineering**	**Current**	410	8(2%)
**Career: Information Systems**	**Current**	209	4(1.9%)
**Career: Literary Studies**	**Current**	159	3(1.9%)
**Career: Cultural Studies**	**Current**	54	1(1.9%)
**Career: Languages & Linguistics**	**Current**	110	2(1.8%)
**Career: Fisheries**	**Current**	227	4(1.8%)
**Career: Archaeology**	**Current**	114	2(1.8%)
**Career: Computation Theory & Mathematics**	**Current**	177	3(1.7%)
**Career: Numerical & Computational Mathematics**	**Current**	120	2(1.7%)
**Career: Urban & Regional Planning**	**Current**	61	1(1.6%)
**Career: Anthropology**	**Current**	123	2(1.6%)
**Career: Materials**	**Current**	1363	22(1.6%)
**Career: Fluids & Plasmas**	**Current**	372	6(1.6%)
**Career: General Mathematics**	**Current**	502	8(1.6%)
**Career: Building & Construction**	**Current**	128	2(1.6%)
**Career: Philosophy**	**Current**	130	2(1.5%)
**Career: Meteorology & Atmospheric Sciences**	**Current**	923	14(1.5%)
**Career: Aerospace & Aeronautics**	**Current**	754	11(1.5%)
**Career: Energy**	**Current**	1212	17(1.4%)

© 2022 Ioannidis JPA et al. *JAMA Network Open.*

**Career: Geological & Geomatics Engineering**	**Current**	357	5(1.4%)
**Career: Ecology**	**Current**	743	10(1.3%)
**Career: Oceanography**	**Current**	185	2(1.1%)
**Career: Business & Management**	**Current**	526	5(.95%)
**Career: Law**	**Current**	106	1(.94%)
**Career: Applied Physics**	**Current**	1918	18(.94%)
**Career: Marine Biology & Hydrobiology**	**Current**	331	3(.91%)
**Career: Physical Chemistry**	**Current**	114	1(.88%)
**Career: Nuclear & Particle Physics**	**Current**	1030	8(.78%)
**Career: Astronomy & Astrophysics**	**Current**	789	6(.76%)
**Career: Civil Engineering**	**Current**	275	2(.73%)
**Career: Geochemistry & Geophysics**	**Current**	843	6(.71%)
**Career: Agronomy & Agriculture**	**Current**	456	3(.66%)
**Career: Inorganic & Nuclear Chemistry**	**Current**	318	2(.63%)
**Career: Forestry**	**Current**	209	1(.48%)
**Career: Zoology**	**Current**	99	0(0%)
**Career: Paleontology**	**Current**	168	0(0%)
**Career: Music**	**Current**	42	0(0%)
**Career: Mathematical Physics**	**Current**	24	0(0%)
**Career: Legal & Forensic Medicine**	**Current**	66	0(0%)
**Career: International Relations**	**Current**	68	0(0%)
**Career: Industrial Relations**	**Current**	8	0(0%)
**Career: Horticulture**	**Current**	75	0(0%)
**Career: History of Social Sciences**	**Current**	23	0(0%)
**Career: History of Science, Technology & Medicine**	**Current**	20	0(0%)
**Career: History**	**Current**	111	0(0%)
**Career: Geology**	**Current**	92	0(0%)
**Career: General Psychology & Cognitive Sciences**	**Current**	65	0(0%)
**Career: Gender Studies**	**Current**	13	0(0%)
**Career: Folklore**	**Current**	6	0(0%)
**Career: Classics**	**Current**	18	0(0%)
**Career: Automobile Design & Engineering**	**Current**	7	0(0%)
**Career: Art Practice, History & Theory**	**Current**	32	0(0%)
**Career: Architecture**	**Current**	10	0(0%)
**Career: Accounting**	**Current**	42	0(0%)

© 2022 Ioannidis JPA et al. *JAMA Network Open.*

eTable 1.4: Percentage funded by field for Recent year: Any funding time

Top-cited US-based researchers: Subfield	Funding time	Total	Funded (%)
Recent year: Geriatrics	Any	75	66(88%)
Recent year: Gerontology	Any	112	97(87%)
Recent year: Substance Abuse	Any	214	184(86%)
Recent year: Developmental Biology	Any	1621	1388(86%)
Recent year: Endocrinology & Metabolism	Any	729	607(83%)
Recent year: Immunology	Any	1329	1103(83%)
Recent year: Neurology & Neurosurgery	Any	2793	2283(82%)
Recent year: Biochemistry & Molecular Biology	Any	1718	1395(81%)
Recent year: Virology	Any	685	552(81%)
Recent year: Psychiatry	Any	757	610(81%)
Recent year: Genetics & Heredity	Any	335	263(79%)
Recent year: Allergy	Any	122	91(75%)
Recent year: Biophysics	Any	169	125(74%)
Recent year: Epidemiology	Any	129	95(74%)
Recent year: Biomedical Engineering	Any	526	387(74%)
Recent year: Arthritis & Rheumatology	Any	206	151(73%)
Recent year: Oncology & Carcinogenesis	Any	2746	1990(72%)
Recent year: Developmental & Child Psychology	Any	513	368(72%)
Recent year: Medical Informatics	Any	119	85(71%)
Recent year: Physiology	Any	228	162(71%)
Recent year: Public Health	Any	637	452(71%)
Recent year: Gastroenterology & Hepatology	Any	631	436(69%)
Recent year: Urology & Nephrology	Any	737	494(67%)
Recent year: Respiratory System	Any	546	365(67%)
Recent year: Pediatrics	Any	714	476(67%)
Recent year: Bioinformatics	Any	180	120(67%)
Recent year: Cardiovascular System & Hematology	Any	1694	1119(66%)
Recent year: Health Policy & Services	Any	191	123(64%)
Recent year: Experimental Psychology	Any	610	388(64%)
Recent year: Emergency & Critical Care Medicine	Any	261	165(63%)
Recent year: Clinical Psychology	Any	234	147(63%)
Recent year: Ophthalmology & Optometry	Any	695	432(62%)
Recent year: Family Studies	Any	55	34(62%)
Recent year: Speech-Language Pathology & Audiology	Any	114	70(61%)
Recent year: Rehabilitation	Any	199	122(61%)
Recent year: Obstetrics & Reproductive Medicine	Any	637	385(60%)
Recent year: Toxicology	Any	433	259(60%)
Recent year: Environmental & Occupational Health	Any	98	58(59%)
Recent year: Nutrition & Dietetics	Any	320	189(59%)
Recent year: Microbiology	Any	1319	779(59%)
Recent year: Nursing	Any	764	447(59%)
Recent year: Demography	Any	36	21(58%)
Recent year: Analytical Chemistry	Any	333	193(58%)

© 2022 Ioannidis JPA et al. *JAMA Network Open.*

**Recent year: Nuclear Medicine & Medical Imaging**	**Any**	1007	576(57%)
**Recent year: Organic Chemistry**	**Any**	574	323(56%)
**Recent year: Pharmacology & Pharmacy**	**Any**	624	333(53%)
**Recent year: Medicinal & Biomolecular Chemistry**	**Any**	384	196(51%)
**Recent year: Complementary & Alternative Medicine**	**Any**	50	25(50%)
**Recent year: Behavioral Science & Comparative Psychology**	**Any**	127	62(49%)
**Recent year: Anesthesiology**	**Any**	403	195(48%)
**Recent year: Dentistry**	**Any**	441	210(48%)
**Recent year: Otorhinolaryngology**	**Any**	506	239(47%)
**Recent year: Tropical Medicine**	**Any**	213	99(46%)
**Recent year: Applied Ethics**	**Any**	54	25(46%)
**Recent year: General & Internal Medicine**	**Any**	1293	592(46%)
**Recent year: Statistics & Probability**	**Any**	270	121(45%)
**Recent year: Surgery**	**Any**	1125	499(44%)
**Recent year: Mycology & Parasitology**	**Any**	105	46(44%)
**Recent year: Dermatology & Venereal Diseases**	**Any**	341	144(42%)
**Recent year: History of Social Sciences**	**Any**	17	7(41%)
**Recent year: Biotechnology**	**Any**	185	76(41%)
**Recent year: General Chemistry**	**Any**	415	167(40%)
**Recent year: Sport Sciences**	**Any**	159	61(38%)
**Recent year: Social Psychology**	**Any**	562	214(38%)
**Recent year: Acoustics**	**Any**	233	86(37%)
**Recent year: Optics**	**Any**	437	138(32%)
**Recent year: Orthopedics**	**Any**	716	224(31%)
**Recent year: Chemical Physics**	**Any**	697	208(30%)
**Recent year: Evolutionary Biology**	**Any**	366	109(30%)
**Recent year: Nanoscience & Nanotechnology**	**Any**	595	173(29%)
**Recent year: Sociology**	**Any**	245	69(28%)
**Recent year: Pathology**	**Any**	299	84(28%)
**Recent year: Plant Biology & Botany**	**Any**	683	186(27%)
**Recent year: General Clinical Medicine**	**Any**	103	28(27%)
**Recent year: Veterinary Sciences**	**Any**	483	130(27%)
**Recent year: Environmental Sciences**	**Any**	234	62(26%)
**Recent year: Social Sciences Methods**	**Any**	100	25(25%)
**Recent year: Criminology**	**Any**	188	47(25%)
**Recent year: Microscopy**	**Any**	33	8(24%)
**Recent year: Inorganic & Nuclear Chemistry**	**Any**	205	47(23%)
**Recent year: Social Work**	**Any**	83	19(23%)
**Recent year: Polymers**	**Any**	375	79(21%)
**Recent year: Drama & Theater**	**Any**	10	2(20%)
**Recent year: Economics**	**Any**	586	112(19%)
**Recent year: Food Science**	**Any**	169	32(19%)
**Recent year: Artificial Intelligence & Image Processing**	**Any**	1230	228(19%)
**Recent year: Distributed Computing**	**Any**	103	19(18%)
**Recent year: Industrial Engineering & Automation**	**Any**	492	83(17%)
**Recent year: Entomology**	**Any**	259	43(17%)
**Recent year: Design Practice & Management**	**Any**	67	11(16%)
**Recent year: Human Factors**	**Any**	177	29(16%)

© 2022 Ioannidis JPA et al. *JAMA Network Open.*

**Recent year: Information & Library Sciences**	**Any**	95	15(16%)
**Recent year: Fluids & Plasmas**	**Any**	325	51(16%)
**Recent year: General Psychology & Cognitive Sciences**	**Any**	64	10(16%)
**Recent year: Urban & Regional Planning**	**Any**	71	11(15%)
**Recent year: Education**	**Any**	699	108(15%)
**Recent year: Psychoanalysis**	**Any**	33	5(15%)
**Recent year: Development Studies**	**Any**	27	4(15%)
**Recent year: Anatomy & Morphology**	**Any**	56	8(14%)
**Recent year: Geography**	**Any**	120	17(14%)
**Recent year: Ornithology**	**Any**	29	4(14%)
**Recent year: History of Science, Technology & Medicine**	**Any**	15	2(13%)
**Recent year: Gender Studies**	**Any**	15	2(13%)
**Recent year: Dairy & Animal Science**	**Any**	336	43(13%)
**Recent year: Logistics & Transportation**	**Any**	151	19(13%)
**Recent year: Economic Theory**	**Any**	16	2(13%)
**Recent year: Optoelectronics & Photonics**	**Any**	697	87(12%)
**Recent year: Software Engineering**	**Any**	178	22(12%)
**Recent year: Marine Biology & Hydrobiology**	**Any**	287	35(12%)
**Recent year: Anthropology**	**Any**	116	14(12%)
**Recent year: Numerical & Computational Mathematics**	**Any**	101	12(12%)
**Recent year: Geological & Geomatics Engineering**	**Any**	282	31(11%)
**Recent year: Electrical & Electronic Engineering**	**Any**	364	40(11%)
**Recent year: Physical Chemistry**	**Any**	107	11(10%)
**Recent year: Econometrics**	**Any**	49	5(10%)
**Recent year: Computer Hardware & Architecture**	**Any**	226	23(10%)
**Recent year: Communication & Media Studies**	**Any**	187	19(10%)
**Recent year: Environmental Engineering**	**Any**	316	32(10%)
**Recent year: Networking & Telecommunications**	**Any**	1008	102(10%)
**Recent year: Archaeology**	**Any**	101	10(9.9%)
**Recent year: Zoology**	**Any**	92	9(9.8%)
**Recent year: Religions & Theology**	**Any**	82	8(9.8%)
**Recent year: Chemical Engineering**	**Any**	188	18(9.6%)
**Recent year: Ecology**	**Any**	811	76(9.4%)
**Recent year: Materials**	**Any**	980	91(9.3%)
**Recent year: General Physics**	**Any**	449	41(9.1%)
**Recent year: Oceanography**	**Any**	168	15(8.9%)
**Recent year: Building & Construction**	**Any**	102	9(8.8%)
**Recent year: Sport, Leisure & Tourism**	**Any**	104	9(8.7%)
**Recent year: Applied Mathematics**	**Any**	104	9(8.7%)
**Recent year: Mechanical Engineering & Transports**	**Any**	487	42(8.6%)
**Recent year: Strategic, Defence & Security Studies**	**Any**	130	11(8.5%)
**Recent year: Languages & Linguistics**	**Any**	99	8(8.1%)
**Recent year: Computation Theory & Mathematics**	**Any**	176	14(8%)
**Recent year: Marketing**	**Any**	254	20(7.9%)
**Recent year: Law**	**Any**	92	7(7.6%)
**Recent year: Meteorology & Atmospheric Sciences**	**Any**	1047	77(7.4%)
**Recent year: Applied Physics**	**Any**	1547	113(7.3%)
**Recent year: Operations Research**	**Any**	167	12(7.2%)

© 2022 Ioannidis JPA et al. *JAMA Network Open.*

**Recent year: Finance**	**Any**	181	13(7.2%)
**Recent year: Political Science & Public Administration**	**Any**	345	24(7%)
**Recent year: Forestry**	**Any**	175	12(6.9%)
**Recent year: Business & Management**	**Any**	818	54(6.6%)
**Recent year: Literary Studies**	**Any**	138	9(6.5%)
**Recent year: General Mathematics**	**Any**	423	26(6.1%)
**Recent year: Aerospace & Aeronautics**	**Any**	638	39(6.1%)
**Recent year: Geology**	**Any**	82	5(6.1%)
**Recent year: History**	**Any**	101	6(5.9%)
**Recent year: Information Systems**	**Any**	169	10(5.9%)
**Recent year: Energy**	**Any**	850	50(5.9%)
**Recent year: Science Studies**	**Any**	35	2(5.7%)
**Recent year: Legal & Forensic Medicine**	**Any**	53	3(5.7%)
**Recent year: Cultural Studies**	**Any**	36	2(5.6%)
**Recent year: Astronomy & Astrophysics**	**Any**	638	35(5.5%)
**Recent year: Accounting**	**Any**	73	4(5.5%)
**Recent year: Music**	**Any**	37	2(5.4%)
**Recent year: Geochemistry & Geophysics**	**Any**	741	40(5.4%)
**Recent year: Agronomy & Agriculture**	**Any**	361	19(5.3%)
**Recent year: Nuclear & Particle Physics**	**Any**	945	46(4.9%)
**Recent year: Fisheries**	**Any**	187	9(4.8%)
**Recent year: Classics**	**Any**	21	1(4.8%)
**Recent year: Mining & Metallurgy**	**Any**	43	2(4.7%)
**Recent year: Paleontology**	**Any**	136	6(4.4%)
**Recent year: Agricultural Economics & Policy**	**Any**	69	3(4.3%)
**Recent year: Philosophy**	**Any**	127	5(3.9%)
**Recent year: International Relations**	**Any**	56	2(3.6%)
**Recent year: Civil Engineering**	**Any**	209	6(2.9%)
**Recent year: Mathematical Physics**	**Any**	21	0(0%)
**Recent year: Industrial Relations**	**Any**	7	0(0%)
**Recent year: Horticulture**	**Any**	60	0(0%)
**Recent year: Folklore**	**Any**	7	0(0%)
**Recent year: Automobile Design & Engineering**	**Any**	7	0(0%)
**Recent year: Art Practice, History & Theory**	**Any**	24	0(0%)
**Recent year: Architecture**	**Any**	10	0(0%)

© 2022 Ioannidis JPA et al. *JAMA Network Open.*

eTable 1.5: Percentage funded by field for Recent year: Recent funding time

Top-cited US-based researchers: Subfield	Funding time	Total	Funded (%)
Recent year: Geriatrics	Recent	75	41(55%)
Recent year: Substance Abuse	Recent	214	106(50%)
Recent year: Developmental Biology	Recent	1621	799(49%)
Recent year: Medical Informatics	Recent	119	57(48%)
Recent year: Bioinformatics	Recent	180	83(46%)
Recent year: Virology	Recent	685	311(45%)
Recent year: Immunology	Recent	1329	592(45%)
Recent year: Gerontology	Recent	112	49(44%)
Recent year: Biomedical Engineering	Recent	526	230(44%)
Recent year: Neurology & Neurosurgery	Recent	2793	1160(42%)
Recent year: Oncology & Carcinogenesis	Recent	2746	1088(40%)
Recent year: Public Health	Recent	637	245(38%)
Recent year: Genetics & Heredity	Recent	335	128(38%)
Recent year: Emergency & Critical Care Medicine	Recent	261	99(38%)
Recent year: Epidemiology	Recent	129	47(36%)
Recent year: Psychiatry	Recent	757	269(36%)
Recent year: Gastroenterology & Hepatology	Recent	631	222(35%)
Recent year: Analytical Chemistry	Recent	333	117(35%)
Recent year: Arthritis & Rheumatology	Recent	206	70(34%)
Recent year: Allergy	Recent	122	41(34%)
Recent year: Developmental & Child Psychology	Recent	513	171(33%)
Recent year: Endocrinology & Metabolism	Recent	729	241(33%)
Recent year: Respiratory System	Recent	546	179(33%)
Recent year: Nuclear Medicine & Medical Imaging	Recent	1007	318(32%)
Recent year: Cardiovascular System & Hematology	Recent	1694	534(32%)
Recent year: Health Policy & Services	Recent	191	60(31%)
Recent year: Microbiology	Recent	1319	413(31%)
Recent year: Toxicology	Recent	433	135(31%)
Recent year: Pediatrics	Recent	714	221(31%)
Recent year: Biophysics	Recent	169	52(31%)
Recent year: Rehabilitation	Recent	199	60(30%)
Recent year: Urology & Nephrology	Recent	737	221(30%)
Recent year: Ophthalmology & Optometry	Recent	695	208(30%)
Recent year: Physiology	Recent	228	67(29%)
Recent year: Biochemistry & Molecular Biology	Recent	1718	503(29%)
Recent year: Clinical Psychology	Recent	234	66(28%)
Recent year: Medicinal & Biomolecular Chemistry	Recent	384	108(28%)
Recent year: Obstetrics & Reproductive Medicine	Recent	637	176(28%)
Recent year: Anesthesiology	Recent	403	110(27%)
Recent year: Tropical Medicine	Recent	213	57(27%)
Recent year: Pharmacology & Pharmacy	Recent	624	158(25%)
Recent year: Nutrition & Dietetics	Recent	320	80(25%)
Recent year: Nursing	Recent	764	191(25%)

© 2022 Ioannidis JPA et al. *JAMA Network Open.*

**Recent year: Demography**	**Recent**	36	9(25%)
**Recent year: Speech-Language Pathology & Audiology**	**Recent**	114	28(25%)
**Recent year: Statistics & Probability**	**Recent**	270	65(24%)
**Recent year: Family Studies**	**Recent**	55	13(24%)
**Recent year: Environmental & Occupational Health**	**Recent**	98	23(23%)
**Recent year: Experimental Psychology**	**Recent**	610	137(22%)
**Recent year: Applied Ethics**	**Recent**	54	12(22%)
**Recent year: Organic Chemistry**	**Recent**	574	126(22%)
**Recent year: Optics**	**Recent**	437	95(22%)
**Recent year: Microscopy**	**Recent**	33	7(21%)
**Recent year: General & Internal Medicine**	**Recent**	1293	269(21%)
**Recent year: Biotechnology**	**Recent**	185	38(21%)
**Recent year: Otorhinolaryngology**	**Recent**	506	103(20%)
**Recent year: Surgery**	**Recent**	1125	227(20%)
**Recent year: General Chemistry**	**Recent**	415	77(19%)
**Recent year: Mycology & Parasitology**	**Recent**	105	19(18%)
**Recent year: Nanoscience & Nanotechnology**	**Recent**	595	106(18%)
**Recent year: Behavioral Science & Comparative Psychology**	**Recent**	127	22(17%)
**Recent year: Dermatology & Venereal Diseases**	**Recent**	341	55(16%)
**Recent year: Acoustics**	**Recent**	233	37(16%)
**Recent year: Orthopedics**	**Recent**	716	108(15%)
**Recent year: Environmental Sciences**	**Recent**	234	35(15%)
**Recent year: Sociology**	**Recent**	245	33(13%)
**Recent year: Complementary & Alternative Medicine**	**Recent**	50	6(12%)
**Recent year: Dentistry**	**Recent**	441	52(12%)
**Recent year: Distributed Computing**	**Recent**	103	12(12%)
**Recent year: Pathology**	**Recent**	299	34(11%)
**Recent year: Evolutionary Biology**	**Recent**	366	41(11%)
**Recent year: Artificial Intelligence & Image Processing**	**Recent**	1230	134(11%)
**Recent year: Veterinary Sciences**	**Recent**	483	51(11%)
**Recent year: Plant Biology & Botany**	**Recent**	683	71(10%)
**Recent year: Chemical Physics**	**Recent**	697	71(10%)
**Recent year: Industrial Engineering & Automation**	**Recent**	492	50(10%)
**Recent year: Sport Sciences**	**Recent**	159	16(10%)
**Recent year: Drama & Theater**	**Recent**	10	1(10%)
**Recent year: Social Work**	**Recent**	83	8(9.6%)
**Recent year: Criminology**	**Recent**	188	18(9.6%)
**Recent year: Design Practice & Management**	**Recent**	67	6(9%)
**Recent year: Polymers**	**Recent**	375	32(8.5%)
**Recent year: General Clinical Medicine**	**Recent**	103	8(7.8%)
**Recent year: Geography**	**Recent**	120	9(7.5%)
**Recent year: Social Psychology**	**Recent**	562	42(7.5%)
**Recent year: Development Studies**	**Recent**	27	2(7.4%)
**Recent year: Economics**	**Recent**	586	42(7.2%)
**Recent year: Fluids & Plasmas**	**Recent**	325	23(7.1%)
**Recent year: Social Sciences Methods**	**Recent**	100	7(7%)
**Recent year: Entomology**	**Recent**	259	18(6.9%)
**Recent year: Numerical & Computational Mathematics**	**Recent**	101	7(6.9%)

© 2022 Ioannidis JPA et al. *JAMA Network Open.*

**Recent year: Electrical & Electronic Engineering**	**Recent**	364	25(6.9%)
**Recent year: Gender Studies**	**Recent**	15	1(6.7%)
**Recent year: Computer Hardware & Architecture**	**Recent**	226	15(6.6%)
**Recent year: Logistics & Transportation**	**Recent**	151	10(6.6%)
**Recent year: Food Science**	**Recent**	169	11(6.5%)
**Recent year: Information & Library Sciences**	**Recent**	95	6(6.3%)
**Recent year: Psychoanalysis**	**Recent**	33	2(6.1%)
**Recent year: Education**	**Recent**	699	42(6%)
**Recent year: Archaeology**	**Recent**	101	6(5.9%)
**Recent year: Optoelectronics & Photonics**	**Recent**	697	41(5.9%)
**Recent year: History of Social Sciences**	**Recent**	17	1(5.9%)
**Recent year: Science Studies**	**Recent**	35	2(5.7%)
**Recent year: Human Factors**	**Recent**	177	10(5.6%)
**Recent year: Urban & Regional Planning**	**Recent**	71	4(5.6%)
**Recent year: Networking & Telecommunications**	**Recent**	1008	52(5.2%)
**Recent year: Dairy & Animal Science**	**Recent**	336	17(5.1%)
**Recent year: Languages & Linguistics**	**Recent**	99	5(5.1%)
**Recent year: Building & Construction**	**Recent**	102	5(4.9%)
**Recent year: Religions & Theology**	**Recent**	82	4(4.9%)
**Recent year: Inorganic & Nuclear Chemistry**	**Recent**	205	10(4.9%)
**Recent year: Applied Mathematics**	**Recent**	104	5(4.8%)
**Recent year: Mechanical Engineering & Transports**	**Recent**	487	23(4.7%)
**Recent year: General Psychology & Cognitive Sciences**	**Recent**	64	3(4.7%)
**Recent year: Mining & Metallurgy**	**Recent**	43	2(4.7%)
**Recent year: Software Engineering**	**Recent**	178	8(4.5%)
**Recent year: Anthropology**	**Recent**	116	5(4.3%)
**Recent year: Materials**	**Recent**	980	42(4.3%)
**Recent year: Chemical Engineering**	**Recent**	188	8(4.3%)
**Recent year: Accounting**	**Recent**	73	3(4.1%)
**Recent year: Econometrics**	**Recent**	49	2(4.1%)
**Recent year: Ecology**	**Recent**	811	32(3.9%)
**Recent year: Sport, Leisure & Tourism**	**Recent**	104	4(3.8%)
**Recent year: Legal & Forensic Medicine**	**Recent**	53	2(3.8%)
**Recent year: Communication & Media Studies**	**Recent**	187	7(3.7%)
**Recent year: Physical Chemistry**	**Recent**	107	4(3.7%)
**Recent year: Operations Research**	**Recent**	167	6(3.6%)
**Recent year: Anatomy & Morphology**	**Recent**	56	2(3.6%)
**Recent year: Information Systems**	**Recent**	169	6(3.6%)
**Recent year: Marketing**	**Recent**	254	9(3.5%)
**Recent year: Ornithology**	**Recent**	29	1(3.4%)
**Recent year: General Physics**	**Recent**	449	15(3.3%)
**Recent year: Finance**	**Recent**	181	6(3.3%)
**Recent year: Zoology**	**Recent**	92	3(3.3%)
**Recent year: Oceanography**	**Recent**	168	5(3%)
**Recent year: Meteorology & Atmospheric Sciences**	**Recent**	1047	31(3%)
**Recent year: Applied Physics**	**Recent**	1547	44(2.8%)
**Recent year: Geological & Geomatics Engineering**	**Recent**	282	8(2.8%)
**Recent year: Aerospace & Aeronautics**	**Recent**	638	18(2.8%)

© 2022 Ioannidis JPA et al. *JAMA Network Open.*

**Recent year: Marine Biology & Hydrobiology**	**Recent**	287	8(2.8%)
**Recent year: Cultural Studies**	**Recent**	36	1(2.8%)
**Recent year: Energy**	**Recent**	850	22(2.6%)
**Recent year: Environmental Engineering**	**Recent**	316	8(2.5%)
**Recent year: Geology**	**Recent**	82	2(2.4%)
**Recent year: Business & Management**	**Recent**	818	19(2.3%)
**Recent year: Strategic, Defence & Security Studies**	**Recent**	130	3(2.3%)
**Recent year: Computation Theory & Mathematics**	**Recent**	176	4(2.3%)
**Recent year: Law**	**Recent**	92	2(2.2%)
**Recent year: Political Science & Public Administration**	**Recent**	345	7(2%)
**Recent year: History**	**Recent**	101	2(2%)
**Recent year: International Relations**	**Recent**	56	1(1.8%)
**Recent year: Astronomy & Astrophysics**	**Recent**	638	11(1.7%)
**Recent year: General Mathematics**	**Recent**	423	7(1.7%)
**Recent year: Fisheries**	**Recent**	187	3(1.6%)
**Recent year: Philosophy**	**Recent**	127	2(1.6%)
**Recent year: Literary Studies**	**Recent**	138	2(1.4%)
**Recent year: Agricultural Economics & Policy**	**Recent**	69	1(1.4%)
**Recent year: Civil Engineering**	**Recent**	209	3(1.4%)
**Recent year: Geochemistry & Geophysics**	**Recent**	741	10(1.3%)
**Recent year: Forestry**	**Recent**	175	2(1.1%)
**Recent year: Nuclear & Particle Physics**	**Recent**	945	10(1.1%)
**Recent year: Agronomy & Agriculture**	**Recent**	361	3(.83%)
**Recent year: Paleontology**	**Recent**	136	1(.74%)
**Recent year: Music**	**Recent**	37	0(0%)
**Recent year: Mathematical Physics**	**Recent**	21	0(0%)
**Recent year: Industrial Relations**	**Recent**	7	0(0%)
**Recent year: Horticulture**	**Recent**	60	0(0%)
**Recent year: History of Science, Technology & Medicine**	**Recent**	15	0(0%)
**Recent year: Folklore**	**Recent**	7	0(0%)
**Recent year: Economic Theory**	**Recent**	16	0(0%)
**Recent year: Classics**	**Recent**	21	0(0%)
**Recent year: Automobile Design & Engineering**	**Recent**	7	0(0%)
**Recent year: Art Practice, History & Theory**	**Recent**	24	0(0%)
**Recent year: Architecture**	**Recent**	10	0(0%)

© 2022 Ioannidis JPA et al. *JAMA Network Open.*

eTable 1.6: Percentage funded by field for Recent year: Current funding time

Top-cited US-based researchers: Subfield	Funding time	Total	Funded (%)
Recent year: Geriatrics	Current	75	32(43%)
Recent year: Developmental Biology	Current	1621	590(36%)
Recent year: Medical Informatics	Current	119	41(34%)
Recent year: Bioinformatics	Current	180	61(34%)
Recent year: Substance Abuse	Current	214	67(31%)
Recent year: Gerontology	Current	112	35(31%)
Recent year: Biomedical Engineering	Current	526	160(30%)
Recent year: Virology	Current	685	206(30%)
Recent year: Immunology	Current	1329	394(30%)
Recent year: Neurology & Neurosurgery	Current	2793	801(29%)
Recent year: Allergy	Current	122	33(27%)
Recent year: Emergency & Critical Care Medicine	Current	261	70(27%)
Recent year: Oncology & Carcinogenesis	Current	2746	725(26%)
Recent year: Public Health	Current	637	163(26%)
Recent year: Gastroenterology & Hepatology	Current	631	154(24%)
Recent year: Psychiatry	Current	757	177(23%)
Recent year: Arthritis & Rheumatology	Current	206	48(23%)
Recent year: Epidemiology	Current	129	29(22%)
Recent year: Genetics & Heredity	Current	335	75(22%)
Recent year: Analytical Chemistry	Current	333	74(22%)
Recent year: Ophthalmology & Optometry	Current	695	153(22%)
Recent year: Developmental & Child Psychology	Current	513	112(22%)
Recent year: Nuclear Medicine & Medical Imaging	Current	1007	219(22%)
Recent year: Endocrinology & Metabolism	Current	729	156(21%)
Recent year: Biophysics	Current	169	36(21%)
Recent year: Toxicology	Current	433	91(21%)
Recent year: Cardiovascular System & Hematology	Current	1694	354(21%)
Recent year: Respiratory System	Current	546	114(21%)
Recent year: Physiology	Current	228	47(21%)
Recent year: Rehabilitation	Current	199	41(21%)
Recent year: Microbiology	Current	1319	269(20%)
Recent year: Urology & Nephrology	Current	737	148(20%)
Recent year: Medicinal & Biomolecular Chemistry	Current	384	77(20%)
Recent year: Health Policy & Services	Current	191	38(20%)
Recent year: Pediatrics	Current	714	136(19%)
Recent year: Obstetrics & Reproductive Medicine	Current	637	119(19%)
Recent year: Anesthesiology	Current	403	72(18%)
Recent year: Statistics & Probability	Current	270	47(17%)
Recent year: Biochemistry & Molecular Biology	Current	1718	290(17%)
Recent year: Speech-Language Pathology & Audiology	Current	114	19(17%)
Recent year: Demography	Current	36	6(17%)
Recent year: Experimental Psychology	Current	610	97(16%)
Recent year: Pharmacology & Pharmacy	Current	624	96(15%)

© 2022 Ioannidis JPA et al. *JAMA Network Open.*

**Recent year: Clinical Psychology**	**Current**	234	35(15%)
**Recent year: Applied Ethics**	**Current**	54	8(15%)
**Recent year: Organic Chemistry**	**Current**	574	85(15%)
**Recent year: Nutrition & Dietetics**	**Current**	320	46(14%)
**Recent year: Mycology & Parasitology**	**Current**	105	15(14%)
**Recent year: Nursing**	**Current**	764	109(14%)
**Recent year: Optics**	**Current**	437	61(14%)
**Recent year: General Chemistry**	**Current**	415	56(13%)
**Recent year: General & Internal Medicine**	**Current**	1293	173(13%)
**Recent year: Surgery**	**Current**	1125	150(13%)
**Recent year: Nanoscience & Nanotechnology**	**Current**	595	75(13%)
**Recent year: Otorhinolaryngology**	**Current**	506	62(12%)
**Recent year: Environmental & Occupational Health**	**Current**	98	12(12%)
**Recent year: Tropical Medicine**	**Current**	213	26(12%)
**Recent year: Microscopy**	**Current**	33	4(12%)
**Recent year: Biotechnology**	**Current**	185	22(12%)
**Recent year: Drama & Theater**	**Current**	10	1(10%)
**Recent year: Complementary & Alternative Medicine**	**Current**	50	5(10%)
**Recent year: Acoustics**	**Current**	233	23(9.9%)
**Recent year: Dermatology & Venereal Diseases**	**Current**	341	33(9.7%)
**Recent year: Orthopedics**	**Current**	716	68(9.5%)
**Recent year: Family Studies**	**Current**	55	5(9.1%)
**Recent year: Environmental Sciences**	**Current**	234	21(9%)
**Recent year: Behavioral Science & Comparative Psychology**	**Current**	127	11(8.7%)
**Recent year: Sociology**	**Current**	245	19(7.8%)
**Recent year: Pathology**	**Current**	299	23(7.7%)
**Recent year: Design Practice & Management**	**Current**	67	5(7.5%)
**Recent year: Dentistry**	**Current**	441	32(7.3%)
**Recent year: Plant Biology & Botany**	**Current**	683	49(7.2%)
**Recent year: Evolutionary Biology**	**Current**	366	26(7.1%)
**Recent year: Artificial Intelligence & Image Processing**	**Current**	1230	83(6.7%)
**Recent year: Chemical Physics**	**Current**	697	47(6.7%)
**Recent year: Industrial Engineering & Automation**	**Current**	492	33(6.7%)
**Recent year: Criminology**	**Current**	188	12(6.4%)
**Recent year: Sport Sciences**	**Current**	159	10(6.3%)
**Recent year: Veterinary Sciences**	**Current**	483	30(6.2%)
**Recent year: Psychoanalysis**	**Current**	33	2(6.1%)
**Recent year: Social Work**	**Current**	83	5(6%)
**Recent year: Polymers**	**Current**	375	22(5.9%)
**Recent year: General Clinical Medicine**	**Current**	103	6(5.8%)
**Recent year: Entomology**	**Current**	259	15(5.8%)
**Recent year: Religions & Theology**	**Current**	82	4(4.9%)
**Recent year: Distributed Computing**	**Current**	103	5(4.9%)
**Recent year: Computer Hardware & Architecture**	**Current**	226	10(4.4%)
**Recent year: Information & Library Sciences**	**Current**	95	4(4.2%)
**Recent year: Education**	**Current**	699	28(4%)
**Recent year: Logistics & Transportation**	**Current**	151	6(4%)
**Recent year: Social Psychology**	**Current**	562	22(3.9%)

© 2022 Ioannidis JPA et al. *JAMA Network Open.*

**Recent year: Applied Mathematics**	**Current**	104	4(3.8%)
**Recent year: Legal & Forensic Medicine**	**Current**	53	2(3.8%)
**Recent year: Physical Chemistry**	**Current**	107	4(3.7%)
**Recent year: Development Studies**	**Current**	27	1(3.7%)
**Recent year: Economics**	**Current**	586	21(3.6%)
**Recent year: Anatomy & Morphology**	**Current**	56	2(3.6%)
**Recent year: Food Science**	**Current**	169	6(3.6%)
**Recent year: Ornithology**	**Current**	29	1(3.4%)
**Recent year: Geography**	**Current**	120	4(3.3%)
**Recent year: Electrical & Electronic Engineering**	**Current**	364	12(3.3%)
**Recent year: Networking & Telecommunications**	**Current**	1008	33(3.3%)
**Recent year: Dairy & Animal Science**	**Current**	336	11(3.3%)
**Recent year: General Psychology & Cognitive Sciences**	**Current**	64	2(3.1%)
**Recent year: Fluids & Plasmas**	**Current**	325	10(3.1%)
**Recent year: Social Sciences Methods**	**Current**	100	3(3%)
**Recent year: Numerical & Computational Mathematics**	**Current**	101	3(3%)
**Recent year: Building & Construction**	**Current**	102	3(2.9%)
**Recent year: Mechanical Engineering & Transports**	**Current**	487	14(2.9%)
**Recent year: Science Studies**	**Current**	35	1(2.9%)
**Recent year: Cultural Studies**	**Current**	36	1(2.8%)
**Recent year: Marketing**	**Current**	254	7(2.8%)
**Recent year: Anthropology**	**Current**	116	3(2.6%)
**Recent year: Materials**	**Current**	980	24(2.4%)
**Recent year: Operations Research**	**Current**	167	4(2.4%)
**Recent year: Mining & Metallurgy**	**Current**	43	1(2.3%)
**Recent year: Optoelectronics & Photonics**	**Current**	697	16(2.3%)
**Recent year: Human Factors**	**Current**	177	4(2.3%)
**Recent year: Software Engineering**	**Current**	178	4(2.2%)
**Recent year: Finance**	**Current**	181	4(2.2%)
**Recent year: Meteorology & Atmospheric Sciences**	**Current**	1047	23(2.2%)
**Recent year: Communication & Media Studies**	**Current**	187	4(2.1%)
**Recent year: Econometrics**	**Current**	49	1(2%)
**Recent year: Political Science & Public Administration**	**Current**	345	7(2%)
**Recent year: Languages & Linguistics**	**Current**	99	2(2%)
**Recent year: General Physics**	**Current**	449	9(2%)
**Recent year: Archaeology**	**Current**	101	2(2%)
**Recent year: Sport, Leisure & Tourism**	**Current**	104	2(1.9%)
**Recent year: Environmental Engineering**	**Current**	316	6(1.9%)
**Recent year: Energy**	**Current**	850	16(1.9%)
**Recent year: Marine Biology & Hydrobiology**	**Current**	287	5(1.7%)
**Recent year: Ecology**	**Current**	811	13(1.6%)
**Recent year: Chemical Engineering**	**Current**	188	3(1.6%)
**Recent year: Philosophy**	**Current**	127	2(1.6%)
**Recent year: Aerospace & Aeronautics**	**Current**	638	10(1.6%)
**Recent year: Inorganic & Nuclear Chemistry**	**Current**	205	3(1.5%)
**Recent year: Agricultural Economics & Policy**	**Current**	69	1(1.4%)
**Recent year: Geological & Geomatics Engineering**	**Current**	282	4(1.4%)
**Recent year: Accounting**	**Current**	73	1(1.4%)

© 2022 Ioannidis JPA et al. *JAMA Network Open.*

**Recent year: Applied Physics**	**Current**	1547	19(1.2%)
**Recent year: Business & Management**	**Current**	818	10(1.2%)
**Recent year: Information Systems**	**Current**	169	2(1.2%)
**Recent year: Computation Theory & Mathematics**	**Current**	176	2(1.1%)
**Recent year: Astronomy & Astrophysics**	**Current**	638	6(.94%)
**Recent year: Geochemistry & Geophysics**	**Current**	741	6(.81%)
**Recent year: Strategic, Defence & Security Studies**	**Current**	130	1(.77%)
**Recent year: Paleontology**	**Current**	136	1(.74%)
**Recent year: Literary Studies**	**Current**	138	1(.72%)
**Recent year: General Mathematics**	**Current**	423	3(.71%)
**Recent year: Nuclear & Particle Physics**	**Current**	945	6(.63%)
**Recent year: Oceanography**	**Current**	168	1(.6%)
**Recent year: Agronomy & Agriculture**	**Current**	361	2(.55%)
**Recent year: Fisheries**	**Current**	187	1(.53%)
**Recent year: Zoology**	**Current**	92	0(0%)
**Recent year: Urban & Regional Planning**	**Current**	71	0(0%)
**Recent year: Music**	**Current**	37	0(0%)
**Recent year: Mathematical Physics**	**Current**	21	0(0%)
**Recent year: Law**	**Current**	92	0(0%)
**Recent year: International Relations**	**Current**	56	0(0%)
**Recent year: Industrial Relations**	**Current**	7	0(0%)
**Recent year: Horticulture**	**Current**	60	0(0%)
**Recent year: History of Social Sciences**	**Current**	17	0(0%)
**Recent year: History of Science, Technology & Medicine**	**Current**	15	0(0%)
**Recent year: History**	**Current**	101	0(0%)
**Recent year: Geology**	**Current**	82	0(0%)
**Recent year: Gender Studies**	**Current**	15	0(0%)
**Recent year: Forestry**	**Current**	175	0(0%)
**Recent year: Folklore**	**Current**	7	0(0%)
**Recent year: Economic Theory**	**Current**	16	0(0%)
**Recent year: Classics**	**Current**	21	0(0%)
**Recent year: Civil Engineering**	**Current**	209	0(0%)
**Recent year: Automobile Design & Engineering**	**Current**	7	0(0%)
**Recent year: Art Practice, History & Theory**	**Current**	24	0(0%)
**Recent year: Architecture**	**Current**	10	0(0%)

© 2022 Ioannidis JPA et al. *JAMA Network Open.*

eTable 2. Citation Counts and Composite Citation Indices for Each Subfield (Ordered by Percentage Funded)

eTable 2.1 : Career-long impact, Funding time any funding citation counts and composite citation indices for each subfield (ordered by percentage funded)

Top-cited US-based researchers: Subfield (perc. funded)	Classification	Citations for funded, median	Citations for non-funded, median	p-value	Composite index for funded, median	Composite index for non-funded, median	p-value
**Developmental Biology (89%)**	Highly related fields	12842	10942	0.003	3.82	3.76	0.014
**Substance Abuse (87%)**	Highly related fields	7834	5392	0.005	3.73	3.64	0.063
**Immunology (85%)**	Highly related fields	13888	12865	0.012	3.81	3.68	<0.001
**Geriatrics (85%)**	Highly related fields	9847	4994	0.003	3.64	3.53	0.142
**Biochemistry & Molecular Biology (81%)**	Highly related fields	10284	7192	<0.001	3.79	3.7	<0.001
**Endocrinology & Metabolism (81%)**	Highly related fields	12233	9650	<0.001	3.79	3.72	<0.001
**Gerontology (80%)**	Highly related fields	7152	3317	0.001	3.74	3.5	0.004
**Virology (80%)**	Highly related fields	10352	7624	<0.001	3.63	3.55	<0.001
**Neurology & Neurosurgery (79%)**	Highly related fields	11476	8561	<0.001	3.81	3.7	<0.001
**Genetics & Heredity (79%)**	Highly related fields	13806	8860	<0.001	3.68	3.62	0.063
**Bioinformatics (77%)**	Highly related fields	12262	7761	0.012	3.63	3.52	0.019
**Psychiatry (76%)**	Highly related fields	12995	8047	<0.001	3.86	3.72	<0.001
**Oncology & Carcinogenesis (74%)**	Highly related fields	14252	11172	<0.001	3.7	3.59	<0.001
**Public Health (73%)**	Highly related fields	7667	6619	0.001	3.68	3.61	0.004
**Medical Informatics (71%)**	Highly related fields	4352	3404	0.041	3.34	3.17	0.009
**Demography (71%)**	Highly related fields	4286	2260	0.058	3.6	3.56	0.911
**Physiology (71%)**	Highly related fields	6838	4617	<0.001	3.75	3.66	0.013
**Epidemiology (71%)**	Highly related fields	16480	10546	0.005	3.89	3.73	0.237

© 2022 Ioannidis JPA et al. *JAMA Network Open.*

**Biomedical Engineering (71%)**	Highly related fields	6113	4070	<0.001	3.39	3.29	0.001
**Developmental & Child Psychology (70%)**	Highly related fields	8495	5279	<0.001	3.81	3.65	<0.001
**Arthritis & Rheumatology (70%)**	Highly related fields	13517	12504	0.049	3.79	3.73	0.021
**Allergy (69%)**	Highly related fields	9587	7563	0.003	3.77	3.66	0.030
**Biophysics (69%)**	Highly related fields	7676	2939	<0.001	3.66	3.35	<0.001
**Respiratory System (66%)**	Highly related fields	11186	9769	0.013	3.69	3.67	0.489
**Experimental Psychology (65%)**	Highly related fields	7137	5142	<0.001	3.8	3.73	0.004
**Gastroenterology & Hepatology (65%)**	Highly related fields	11005	8016	<0.001	3.74	3.66	<0.001
**Cardiovascular System & Hematology (64%)**	Highly related fields	14741	12270	<0.001	3.79	3.69	<0.001
**Health Policy & Services (63%)**	Highly related fields	7915	4993	0.002	3.65	3.54	0.013
**Urology & Nephrology (63%)**	Highly related fields	10090	7520	<0.001	3.71	3.58	<0.001
**Speech-Language Pathology & Audiology (61%)**	Highly related fields	3170	3904	0.347	3.48	3.5	0.417
**Clinical Psychology (60%)**	Highly related fields	10172	5683	<0.001	3.82	3.7	0.004
**Pediatrics (60%)**	Highly related fields	6112	4322	<0.001	3.47	3.36	<0.001
**Microbiology (60%)**	Highly related fields	8973	7541	<0.001	3.68	3.62	<0.001
**Family Studies (59%)**	Other fields	3575	2278	0.007	3.59	3.39	0.048
**Applied Ethics (58%)**	Highly related fields	4638	2669	0.003	3.64	3.47	0.056
**Nutrition & Dietetics (57%)**	Highly related fields	7903	5857	<0.001	3.73	3.6	0.001
**Ophthalmology & Optometry (57%)**	Highly related fields	7416	4649	<0.001	3.58	3.47	<0.001
**Organic Chemistry (57%)**	Other fields	7187	5401	<0.001	3.67	3.55	<0.001
**Environmental & Occupational Health (57%)**	Highly related fields	4184	3353	0.012	3.38	3.26	0.005
**Toxicology (56%)**	Highly related fields	6442	3944	<0.001	3.49	3.39	<0.001
**Nursing (55%)**	Highly related fields	2018	1258	<0.001	3.12	3.03	<0.001

© 2022 Ioannidis JPA et al. *JAMA Network Open.*

**Obstetrics & Reproductive Medicine (54%)**	Highly related fields	7616	5005	<0.001	3.61	3.46	<0.001
**Emergency & Critical Care Medicine (53%)**	Highly related fields	8576	5130	<0.001	3.52	3.43	0.001
**Rehabilitation (53%)**	Highly related fields	4662	3686	0.001	3.47	3.35	0.003
**Pharmacology & Pharmacy (51%)**	Highly related fields	5538	3620	<0.001	3.45	3.31	<0.001
**Behavioral Science & Comparative Psychology (50%)**	Highly related fields	5072	3916	<0.001	3.78	3.69	0.048
**Analytical Chemistry (49%)**	Other fields	6609	3696	<0.001	3.48	3.36	<0.001
**Nuclear Medicine & Medical Imaging (47%)**	Highly related fields	7837	5258	<0.001	3.55	3.39	<0.001
**Otorhinolaryngology (44%)**	Highly related fields	3940	3319	0.001	3.38	3.33	0.004
**Dentistry (44%)**	Highly related fields	4572	2814	<0.001	3.46	3.34	<0.001
**Tropical Medicine (44%)**	Highly related fields	6738	4014	<0.001	3.52	3.32	<0.001
**Mycology & Parasitology (43%)**	Highly related fields	4813	4646	0.539	3.41	3.49	0.998
**Statistics & Probability (43%)**	Other fields	11431	5598	<0.001	3.78	3.66	0.004
**Medicinal & Biomolecular Chemistry (43%)**	Highly related fields	4644	3581	<0.001	3.28	3.14	<0.001
**Anesthesiology (43%)**	Highly related fields	6281	4140	<0.001	3.47	3.37	<0.001
**Dermatology & Venereal Diseases (42%)**	Highly related fields	8008	4697	<0.001	3.68	3.54	<0.001
**Biotechnology (41%)**	Highly related fields	6671	4219	<0.001	3.44	3.27	0.004
**Social Psychology (41%)**	Highly related fields	10567	6215	<0.001	3.93	3.75	<0.001
**Surgery (40%)**	Highly related fields	8570	5061	<0.001	3.47	3.38	<0.001
**General & Internal Medicine (40%)**	Highly related fields	5786	2779	<0.001	3.35	3.14	<0.001
**Complementary & Alternative Medicine (40%)**	Highly related fields	2674	981	<0.001	3.01	2.86	0.042
**Sport Sciences (37%)**	Highly related fields	6500	4860	0.003	3.56	3.52	0.045
**Acoustics (36%)**	Other fields	3620	2680	0.001	3.44	3.4	0.215
**History of Social Sciences (35%)**	Other fields	1745	1740	0.651	3.46	3.25	0.796

© 2022 Ioannidis JPA et al. *JAMA Network Open.*

**Nanoscience & Nanotechnology (35%)**	Other fields	14211	12246	0.032	3.63	3.53	0.079
**General Chemistry (34%)**	Other fields	7990	3518	<0.001	3.63	3.3	<0.001
**Microscopy (33%)**	Highly related fields	9950	4837	0.035	3.57	3.45	0.202
**Criminology (31%)**	Other fields	4848	3566	0.001	3.62	3.6	0.255
**Sociology (31%)**	Other fields	4018	3670	0.193	3.66	3.7	0.291
**Optics (30%)**	Other fields	6704	5176	<0.001	3.38	3.3	0.088
**Pathology (30%)**	Highly related fields	10145	8933	0.039	3.69	3.61	0.321
**Social Work (30%)**	Other fields	2179	1526	0.058	3.26	3.23	0.100
**Evolutionary Biology (29%)**	Other fields	8799	6563	<0.001	3.9	3.78	0.002
**Orthopedics (28%)**	Highly related fields	8903	4973	<0.001	3.64	3.45	<0.001
**Social Sciences Methods (27%)**	Other fields	9838	4916	0.001	4.02	3.71	0.002
**Chemical Physics (27%)**	Other fields	11822	7682	<0.001	3.88	3.77	<0.001
**General Clinical Medicine (24%)**	Highly related fields	3716	2040	<0.001	3.44	3.03	<0.001
**Plant Biology & Botany (24%)**	Other fields	8824	5160	<0.001	3.69	3.47	<0.001
**Inorganic & Nuclear Chemistry (24%)**	Other fields	7773	5586	0.023	3.65	3.52	0.005
**Veterinary Sciences (23%)**	Highly related fields	4095	2952	<0.001	3.37	3.3	0.041
**Environmental Sciences (21%)**	Other fields	7461	4139	<0.001	3.49	3.36	0.001
**Human Factors (19%)**	Highly related fields	5312	4990	0.411	3.62	3.58	0.702
**Ornithology (18%)**	Other fields	2028	3711	0.148	3.4	3.44	0.852
**Urban & Regional Planning (18%)**	Other fields	3257	2278	0.268	3.5	3.54	0.388
**Economics (18%)**	Other fields	5788	4649	0.021	3.74	3.72	0.247
**Artificial Intelligence & Image Processing (17%)**	Other fields	6266	4047	<0.001	3.47	3.33	<0.001
**Polymers (17%)**	Other fields	7916	5343	<0.001	3.57	3.48	0.164
**Economic Theory (17%)**	Other fields	3669	2701	0.859	3.56	3.61	0.953
**Development Studies (17%)**	Other fields	3826	3565	0.757	3.56	3.57	0.535
**Anatomy & Morphology (17%)**	Highly related fields	2612	1419	0.088	3.02	3.07	0.198
**Distributed Computing (17%)**	Other fields	4890	3099	0.001	3.24	3.13	0.072
**Entomology (16%)**	Other fields	4967	3189	<0.001	3.59	3.46	0.036
**Gender Studies (15%)**	Highly related fields	2960	2284	0.324	3.61	3.5	0.236

© 2022 Ioannidis JPA et al. *JAMA Network Open.*

**History of Science, Technology & Medicine (15%)**	Other fields	957	761	0.874	3.15	3.12	0.958
**Software Engineering (15%)**	Other fields	4660	4504	0.191	3.48	3.46	0.437
**Education (15%)**	Other fields	3374	2290	<0.001	3.44	3.35	<0.001
**Food Science (15%)**	Other fields	6002	3877	<0.001	3.57	3.36	0.005
**Industrial Engineering & Automation (14%)**	Other fields	5380	4123	0.002	3.52	3.43	0.165
**Logistics & Transportation (14%)**	Other fields	2504	2848	0.353	3.19	3.29	0.055
**Geography (14%)**	Other fields	4866	3227	0.002	3.83	3.66	0.005
**Sport, Leisure & Tourism (13%)**	Other fields	3071	3757	0.267	3.59	3.56	0.453
**Numerical & Computational Mathematics (13%)**	Other fields	4001	3507	0.163	3.59	3.54	0.821
**Communication & Media Studies (12%)**	Other fields	3009	2781	0.369	3.54	3.48	0.357
**General Psychology & Cognitive Sciences (12%)**	Highly related fields	3202	3365	0.811	3.6	3.47	0.348
**Fluids & Plasmas (12%)**	Other fields	8612	6618	0.014	3.8	3.72	0.114
**Information & Library Sciences (12%)**	Other fields	2294	1245	0.012	3.44	3.14	0.051
**Dairy & Animal Science (12%)**	Other fields	4046	3011	0.005	3.34	3.24	0.158
**Optoelectronics & Photonics (12%)**	Other fields	3848	2806	<0.001	3.15	3.11	0.137
**Anthropology (11%)**	Other fields	4001	3204	0.327	3.73	3.6	0.588
**Religions & Theology (11%)**	Other fields	784	475	0.508	2.89	2.85	0.729
**Drama & Theater (11%)**	Other fields	606	357	0.439	2.8	2.67	0.439
**Design Practice & Management (11%)**	Other fields	4323	2892	0.563	3.24	3.28	0.882
**Environmental Engineering (11%)**	Other fields	5009	4072	0.042	3.42	3.44	0.723
**Chemical Engineering (11%)**	Other fields	4285	4074	0.295	3.44	3.43	0.811
**Applied Mathematics (11%)**	Other fields	4476	4595	0.835	3.52	3.61	0.926
**Marketing (11%)**	Other fields	6545	5722	0.293	3.77	3.64	0.368

© 2022 Ioannidis JPA et al. *JAMA Network Open.*

**Zoology (10%)**	Other fields	2070	2115	0.862	3.24	3.19	0.312
**Architecture (10%)**	Other fields	465	238	0.384	2.45	2.48	0.602
**Ecology (9.7%)**	Other fields	7557	7548	0.412	3.83	3.74	0.019
**Marine Biology & Hydrobiology (9.7%)**	Other fields	6058	6136	0.768	3.66	3.7	0.726
**Networking & Telecommunications (9.5%)**	Other fields	3715	3691	0.192	3.29	3.25	0.497
**Computer Hardware & Architecture (9.3%)**	Other fields	4918	4403	0.823	3.25	3.33	0.505
**Electrical & Electronic Engineering (9.2%)**	Other fields	3502	2503	0.009	3.04	3.04	0.740
**Geological & Geomatics Engineering (9%)**	Other fields	6690	4526	0.060	3.71	3.44	0.034
**Information Systems (8.6%)**	Other fields	6086	5645	0.197	3.61	3.58	0.990
**Law (8.5%)**	Other fields	1025	1252	0.764	3.23	3.22	0.764
**Mathematical Physics (8.3%)**	Other fields	2324	3075	0.465	3.37	3.53	0.144
**Strategic, Defence & Security Studies (8.2%)**	Other fields	2495	2076	0.183	3.49	3.26	0.009
**Agricultural Economics & Policy (8.2%)**	Other fields	3642	4084	0.888	3.58	3.58	0.534
**General Physics (8.2%)**	Other fields	6484	5568	0.295	3.6	3.57	0.903
**Languages & Linguistics (8.2%)**	Other fields	2937	1820	0.082	3.59	3.42	0.061
**Mining & Metallurgy (8.1%)**	Other fields	1218	954	0.544	2.74	2.76	0.826
**Psychoanalysis (8%)**	Highly related fields	1245	1338	0.943	3.23	3.31	0.543
**Political Science & Public Administration (7.9%)**	Other fields	5258	3489	0.023	3.76	3.63	0.231
**Building & Construction (7.8%)**	Other fields	3477	2960	0.135	3.32	3.32	0.887
**Oceanography (7.6%)**	Other fields	6151	4641	0.239	3.68	3.58	0.247
**Literary Studies (7.5%)**	Other fields	382	351	0.686	2.76	2.7	0.620
**Cultural Studies (7.4%)**	Other fields	874	795	0.597	2.99	3.11	0.092
**Materials (7.3%)**	Other fields	4898	3408	<0.001	3.45	3.27	0.002
**Applied Physics (7.2%)**	Other fields	7915	6332	0.007	3.64	3.53	0.002

© 2022 Ioannidis JPA et al. *JAMA Network Open.*

**Econometrics (7.1%)**	Other fields	17980	6910	0.213	3.88	3.97	0.574
**Mechanical Engineering & Transports (7.1%)**	Other fields	3309	2869	0.343	3.4	3.36	0.747
**Operations Research (6.8%)**	Other fields	3871	4231	0.201	3.55	3.6	0.229
**Finance (6.8%)**	Other fields	3236	4958	0.314	3.53	3.61	0.530
**Business & Management (6.5%)**	Other fields	6365	6058	0.535	3.75	3.69	0.244
**Meteorology & Atmospheric Sciences (6.4%)**	Other fields	8805	8746	0.863	3.76	3.74	0.918
**General Mathematics (6.4%)**	Other fields	1961	2247	0.781	3.42	3.43	0.577
**Computation Theory & Mathematics (6.2%)**	Other fields	5982	4894	0.316	3.68	3.62	0.535
**Fisheries (6.2%)**	Other fields	3816	3591	0.876	3.34	3.41	0.950
**Philosophy (6.2%)**	Other fields	1662	1353	0.492	3.43	3.38	0.608
**Archaeology (6.1%)**	Other fields	1927	1923	0.986	3.18	3.31	0.090
**Paleontology (6%)**	Other fields	6741	5589	0.573	3.84	3.71	0.410
**Energy (5.9%)**	Other fields	4465	3323	0.056	3.28	3.26	0.768
**Agronomy & Agriculture (5.9%)**	Other fields	5820	3930	0.248	3.46	3.39	0.508
**Aerospace & Aeronautics (5.8%)**	Other fields	2062	1839	0.170	3.13	3.13	0.903
**Astronomy & Astrophysics (5.6%)**	Other fields	10241	10762	0.789	3.77	3.74	0.185
**Classics (5.6%)**	Other fields	549	534	0.772	2.86	2.92	0.289
**Geology (5.4%)**	Other fields	3832	3385	0.938	3.52	3.48	0.636
**Forestry (5.3%)**	Other fields	2454	2711	0.935	3.29	3.22	0.598
**Geochemistry & Geophysics (4.9%)**	Other fields	6027	6442	0.993	3.8	3.73	0.851
**Nuclear & Particle Physics (4.7%)**	Other fields	6028	6744	0.215	3.61	3.62	0.668
**History (4.5%)**	Other fields	543	530	0.733	2.92	2.88	0.798
**International Relations (4.4%)**	Other fields	2949	2338	0.362	3.54	3.5	0.378
**Physical Chemistry (4.4%)**	Other fields	15148	6278	0.107	3.87	3.51	0.210
**Science Studies (3.8%)**	Other fields	4961	4004	0.549	3.59	3.69	0.257
**Civil Engineering (3.6%)**	Other fields	1610	2205	0.242	3.04	3.17	0.139
**Legal & Forensic Medicine (3%)**	Highly related fields	1132	1332	0.654	2.85	2.98	0.190
**Music (2.4%)**	Other fields	575	437	0.302	3.03	2.78	0.127
**Accounting (2.4%)**	Other fields	10654	5375	0.173	3.9	3.54	0.173
**Horticulture (1.3%)**	Other fields	1584	2110	0.644	3.12	3.14	0.853

© 2022 Ioannidis JPA et al. *JAMA Network Open.*

**Industrial Relations (0%)**	Other fields	.	1524		.	3.34	
**Folklore (0%)**	Other fields	.	310		.	2.61	
**Automobile Design & Engineering (0%)**	Other fields	.	860		.	2.69	
**Art Practice, History & Theory (0%)**	Other fields	.	210		.	2.47	

© 2022 Ioannidis JPA et al. *JAMA Network Open.*

eTable 2.2 : Career-long impact, Funding time recent funding citation counts and composite citation indices for each subfield (ordered by percentage funded)

Top-cited US-based researchers: Subfield (perc. funded)	Classification	Citations for funded, median	Citations for non-funded, median	p-value	Composite index for funded, median	Composite index for non-funded, median	p-value
Developmental Biology (42%)	Highly related fields	12967	12512	0.048	3.8	3.82	0.433
Bioinformatics (41%)	Highly related fields	15297	8222	<0.001	3.69	3.54	0.056
Geriatrics (40%)	Highly related fields	12950	8067	0.010	3.71	3.62	0.306
Substance Abuse (36%)	Highly related fields	8516	7193	0.016	3.68	3.73	0.422
Biomedical Engineering (34%)	Highly related fields	6113	5039	0.011	3.37	3.36	0.958
Medical Informatics (34%)	Highly related fields	4194	3967	0.275	3.35	3.3	0.155
Immunology (34%)	Highly related fields	14470	13093	0.003	3.83	3.77	0.024
Epidemiology (34%)	Highly related fields	15811	14374	0.671	3.74	3.86	0.519
Virology (34%)	Highly related fields	1.0e+04	9705	0.109	3.6	3.61	0.753
Neurology & Neurosurgery (32%)	Highly related fields	11562	10342	<0.001	3.78	3.78	0.728
Public Health (31%)	Highly related fields	7869	7198	0.005	3.71	3.63	0.015
Gerontology (30%)	Highly related fields	7551	6134	0.017	3.74	3.62	0.122
Oncology & Carcinogenesis (30%)	Highly related fields	15064	12526	<0.001	3.66	3.68	0.319
Genetics & Heredity (29%)	Highly related fields	15385	11562	0.001	3.7	3.67	0.548
Demography (26%)	Highly related fields	2837	3432	0.830	3.51	3.61	0.466
Health Policy & Services (26%)	Highly related fields	7301	7555	0.177	3.62	3.59	0.209
Developmental & Child Psychology (25%)	Highly related fields	9051	6643	<0.001	3.86	3.73	<0.001
Applied Ethics (25%)	Highly related fields	6159	3077	<0.001	3.67	3.55	0.110
Gastroenterology & Hepatology (25%)	Highly related fields	12311	9249	0.001	3.73	3.7	0.076

© 2022 Ioannidis JPA et al. *JAMA Network Open.*

**Endocrinology & Metabolism (25%)**	Highly related fields	11786	11565	0.417	3.78	3.77	0.685
**Biophysics (25%)**	Highly related fields	6072	5727	0.170	3.56	3.5	0.752
**Psychiatry (25%)**	Highly related fields	14867	10684	<0.001	3.9	3.81	0.031
**Arthritis & Rheumatology (24%)**	Highly related fields	13201	13569	0.867	3.78	3.78	0.484
**Emergency & Critical Care Medicine (24%)**	Highly related fields	10183	6102	<0.001	3.54	3.46	0.043
**Microbiology (23%)**	Highly related fields	9152	8088	0.010	3.66	3.65	0.419
**Allergy (23%)**	Highly related fields	9374	8456	0.298	3.65	3.75	0.418
**Respiratory System (23%)**	Highly related fields	11174	10570	0.577	3.65	3.69	0.583
**Clinical Psychology (23%)**	Highly related fields	10384	7198	0.002	3.88	3.74	0.029
**Cardiovascular System & Hematology (23%)**	Highly related fields	15696	13298	<0.001	3.77	3.75	0.165
**Biochemistry & Molecular Biology (22%)**	Highly related fields	10813	9176	<0.001	3.77	3.76	0.697
**Physiology (22%)**	Highly related fields	6498	6188	0.112	3.74	3.71	0.457
**Ophthalmology & Optometry (21%)**	Highly related fields	7658	5548	<0.001	3.6	3.52	<0.001
**Analytical Chemistry (21%)**	Other fields	7039	4381	<0.001	3.47	3.4	0.014
**Urology & Nephrology (20%)**	Highly related fields	10675	8539	0.010	3.68	3.66	0.186
**Rehabilitation (20%)**	Highly related fields	4524	4084	0.209	3.43	3.42	0.368
**Microscopy (20%)**	Highly related fields	10562	4837	0.017	3.67	3.45	0.108
**Tropical Medicine (20%)**	Highly related fields	7007	4532	0.003	3.53	3.37	0.104
**Statistics & Probability (20%)**	Other fields	13714	6898	<0.001	3.86	3.7	0.053
**Family Studies (20%)**	Other fields	4770	2920	0.056	3.65	3.48	0.051
**Toxicology (20%)**	Highly related fields	7044	4733	<0.001	3.51	3.44	0.016
**Nutrition & Dietetics (19%)**	Highly related fields	9204	6728	0.014	3.71	3.67	0.720
**Medicinal & Biomolecular Chemistry (19%)**	Highly related fields	4633	3930	0.007	3.24	3.18	0.040

© 2022 Ioannidis JPA et al. *JAMA Network Open.*

**Nuclear Medicine & Medical Imaging (19%)**	Highly related fields	8202	5983	<0.001	3.52	3.44	<0.001
**Nanoscience & Nanotechnology (19%)**	Other fields	14139	12558	0.216	3.61	3.55	0.564
**Pediatrics (19%)**	Highly related fields	6967	4993	<0.001	3.47	3.4	0.003
**Pharmacology & Pharmacy (18%)**	Highly related fields	6252	4316	<0.001	3.45	3.37	0.001
**Optics (18%)**	Other fields	6752	5290	0.001	3.36	3.32	0.502
**Anesthesiology (18%)**	Highly related fields	6464	4597	<0.001	3.45	3.41	0.057
**Obstetrics & Reproductive Medicine (18%)**	Highly related fields	7686	5819	<0.001	3.59	3.51	0.113
**Experimental Psychology (18%)**	Highly related fields	7891	6229	<0.001	3.81	3.77	0.029
**Speech-Language Pathology & Audiology (17%)**	Highly related fields	2806	3457	0.469	3.45	3.5	0.536
**Biotechnology (17%)**	Highly related fields	6095	5049	0.043	3.42	3.31	0.201
**Organic Chemistry (16%)**	Other fields	8620	6297	<0.001	3.67	3.61	0.040
**Nursing (16%)**	Highly related fields	2337	1503	<0.001	3.13	3.06	0.001
**Environmental & Occupational Health (15%)**	Highly related fields	3855	3745	0.758	3.33	3.3	0.758
**Behavioral Science & Comparative Psychology (13%)**	Highly related fields	5918	4397	0.024	3.79	3.71	0.649
**Mycology & Parasitology (13%)**	Highly related fields	4781	4675	0.621	3.42	3.47	0.970
**General & Internal Medicine (13%)**	Highly related fields	6007	3456	<0.001	3.28	3.2	0.001
**Otorhinolaryngology (13%)**	Highly related fields	4290	3479	0.006	3.4	3.35	0.042
**Sociology (13%)**	Other fields	4492	3783	0.414	3.69	3.68	0.461
**Surgery (13%)**	Highly related fields	8499	5842	<0.001	3.51	3.4	0.001
**Development Studies (13%)**	Other fields	2051	4223	0.631	3.56	3.57	0.315
**Drama & Theater (11%)**	Other fields	606	357	0.439	2.8	2.67	0.439
**General Chemistry (11%)**	Other fields	8799	4255	<0.001	3.66	3.34	0.001
**Dermatology & Venereal Diseases (11%)**	Highly related fields	10273	5810	<0.001	3.76	3.6	0.007
**Acoustics (11%)**	Other fields	3241	2923	0.090	3.36	3.42	0.510
**Criminology (10%)**	Other fields	5521	3764	0.016	3.63	3.6	0.414

© 2022 Ioannidis JPA et al. *JAMA Network Open.*

**Evolutionary Biology (10%)**	Other fields	8705	6844	0.018	3.86	3.8	0.497
**Orthopedics (10%)**	Highly related fields	9151	5472	<0.001	3.63	3.5	<0.001
**Environmental Sciences (9.7%)**	Other fields	7291	4399	0.001	3.45	3.37	0.153
**Distributed Computing (9.4%)**	Other fields	4890	3192	0.012	3.13	3.13	0.510
**Social Work (9.1%)**	Other fields	2259	1587	0.087	3.26	3.24	0.159
**Social Sciences Methods (9.1%)**	Other fields	10839	5163	0.040	4.1	3.72	0.018
**Artificial Intelligence & Image Processing (8.9%)**	Other fields	5809	4172	<0.001	3.45	3.34	0.034
**Complementary & Alternative Medicine (8.6%)**	Highly related fields	3306	1372	0.010	3.09	2.86	0.064
**Dentistry (8%)**	Highly related fields	4670	3403	0.005	3.46	3.39	0.319
**Veterinary Sciences (7.9%)**	Highly related fields	4201	3062	0.002	3.35	3.31	0.599
**Pathology (7.8%)**	Highly related fields	12052	9349	0.138	3.67	3.62	0.564
**Plant Biology & Botany (7.8%)**	Other fields	9493	5688	<0.001	3.66	3.5	<0.001
**Gender Studies (7.7%)**	Highly related fields	3202	2400	0.285	3.66	3.5	0.181
**Social Psychology (7.7%)**	Highly related fields	10753	7178	0.085	3.94	3.8	0.211
**Geography (7.6%)**	Other fields	4576	3281	0.055	3.74	3.68	0.188
**Sport Sciences (7.5%)**	Highly related fields	6253	5240	0.706	3.47	3.53	0.718
**Industrial Engineering & Automation (7.3%)**	Other fields	5599	4187	0.047	3.5	3.44	0.379
**Chemical Physics (7.3%)**	Other fields	13599	8323	<0.001	3.93	3.79	0.001
**Urban & Regional Planning (6.6%)**	Other fields	5110	2308	0.308	3.69	3.53	0.662
**Sport, Leisure & Tourism (6.5%)**	Other fields	2884	3753	0.519	3.54	3.57	0.982
**Entomology (6.1%)**	Other fields	7467	3331	0.001	3.71	3.46	0.036
**Human Factors (6%)**	Highly related fields	5070	5089	0.489	3.64	3.58	0.207
**Information & Library Sciences (6%)**	Other fields	2095	1293	0.317	3.19	3.16	0.863
**Numerical & Computational Mathematics (5.8%)**	Other fields	9289	3452	0.064	3.63	3.54	0.542
**Economics (5.7%)**	Other fields	8352	4727	0.005	3.92	3.71	0.033

© 2022 Ioannidis JPA et al. *JAMA Network Open.*

**Economic Theory (5.6%)**	Other fields	3859	2701	0.772	3.51	3.61	0.386
**Classics (5.6%)**	Other fields	361	538	0.101	2.85	2.92	0.101
**Anatomy & Morphology (5.6%)**	Highly related fields	1231	1583	0.985	2.81	3.07	0.015
**General Clinical Medicine (5.5%)**	Highly related fields	6849	2662	0.021	3.61	3.08	0.013
**Logistics & Transportation (5.5%)**	Other fields	2506	2803	0.589	3.19	3.27	0.428
**Computer Hardware & Architecture (5.3%)**	Other fields	5481	4368	0.545	3.23	3.33	0.201
**Polymers (5.3%)**	Other fields	7412	5524	0.017	3.6	3.49	0.506
**Communication & Media Studies (5.2%)**	Other fields	3023	2784	0.430	3.57	3.48	0.536
**Fluids & Plasmas (5.1%)**	Other fields	11761	6618	0.001	3.88	3.73	0.074
**Software Engineering (4.8%)**	Other fields	6139	4522	0.275	3.48	3.46	0.516
**Electrical & Electronic Engineering (4.8%)**	Other fields	3410	2535	0.081	3.03	3.04	0.857
**Optoelectronics & Photonics (4.7%)**	Other fields	4096	2880	0.017	3.11	3.11	0.496
**Marketing (4.7%)**	Other fields	5556	6018	0.884	3.63	3.64	0.561
**Languages & Linguistics (4.5%)**	Other fields	2937	1832	0.184	3.58	3.42	0.426
**Education (4.5%)**	Other fields	4463	2366	<0.001	3.58	3.36	<0.001
**Networking & Telecommunications (4.5%)**	Other fields	4059	3680	0.530	3.17	3.26	0.196
**Religions & Theology (4.4%)**	Other fields	911	463	0.106	3.06	2.85	0.210
**Archaeology (4.4%)**	Other fields	1927	1923	0.906	3.35	3.3	0.443
**History of Social Sciences (4.3%)**	Other fields	1613	1743	0.880	3.31	3.31	1.000
**Applied Mathematics (4.3%)**	Other fields	7299	4499	0.321	3.73	3.61	0.638
**Mathematical Physics (4.2%)**	Other fields	2455	2830	0.718	3.38	3.49	0.427
**Agricultural Economics & Policy (4.1%)**	Other fields	3582	4088	0.221	3.58	3.58	0.453
**Food Science (4.1%)**	Other fields	5487	3996	0.067	3.46	3.39	0.681
**Anthropology (4.1%)**	Other fields	3487	3228	0.828	3.52	3.61	0.391
**Science Studies (3.8%)**	Other fields	4961	4004	0.549	3.59	3.69	0.257

© 2022 Ioannidis JPA et al. *JAMA Network Open.*

**Information Systems (3.8%)**	Other fields	5605	5798	0.501	3.61	3.58	0.563
**Finance (3.8%)**	Other fields	5679	4919	0.882	3.67	3.59	0.571
**Cultural Studies (3.7%)**	Other fields	814	795	0.410	3.13	3.1	0.680
**General Physics (3.7%)**	Other fields	6681	5581	0.465	3.62	3.57	0.819
**Chemical Engineering (3.6%)**	Other fields	5097	4079	0.476	3.55	3.43	0.504
**Mechanical Engineering & Transports (3.5%)**	Other fields	3801	2884	0.291	3.24	3.36	0.789
**Ecology (3.5%)**	Other fields	7263	7548	0.860	3.9	3.75	0.172
**Operations Research (3.4%)**	Other fields	4391	4226	0.645	3.5	3.6	0.371
**Dairy & Animal Science (3.3%)**	Other fields	4212	3062	0.035	3.4	3.25	0.452
**Mining & Metallurgy (3.2%)**	Other fields	5030	1020	0.605	3.25	2.76	0.661
**Building & Construction (3.1%)**	Other fields	5319	2994	0.087	3.21	3.32	0.603
**Philosophy (3.1%)**	Other fields	2367	1358	0.686	3.51	3.38	1.000
**Political Science & Public Administration (3.1%)**	Other fields	4718	3499	0.225	3.57	3.64	0.910
**Zoology (3%)**	Other fields	4056	2076	0.090	3.34	3.2	0.221
**Ornithology (3%)**	Other fields	2139	3168	0.462	3.37	3.43	0.674
**Design Practice & Management (3%)**	Other fields	5320	3001	0.498	3.24	3.28	0.992
**Materials (2.9%)**	Other fields	5960	3443	0.001	3.52	3.28	0.004
**Environmental Engineering (2.9%)**	Other fields	3537	4225	0.629	3.4	3.44	0.665
**Inorganic & Nuclear Chemistry (2.8%)**	Other fields	4382	5908	0.470	3.36	3.55	0.084
**Geological & Geomatics Engineering (2.8%)**	Other fields	10138	4568	0.023	3.76	3.45	0.064
**Oceanography (2.7%)**	Other fields	8622	4618	0.141	3.75	3.58	0.168
**Literary Studies (2.5%)**	Other fields	469	351	0.428	2.81	2.7	0.409
**Meteorology & Atmospheric Sciences (2.5%)**	Other fields	7792	8800	0.145	3.71	3.74	0.675
**Applied Physics (2.4%)**	Other fields	8254	6372	0.051	3.65	3.53	0.065
**Econometrics (2.4%)**	Other fields	17980	6910	0.302	3.88	3.97	0.902
**Business & Management (2.3%)**	Other fields	5650	6071	0.941	3.71	3.7	0.943

© 2022 Ioannidis JPA et al. *JAMA Network Open.*

**Computation Theory & Mathematics (2.3%)**	Other fields	8243	4906	0.203	3.71	3.62	0.483
**Aerospace & Aeronautics (2.3%)**	Other fields	2463	1884	0.301	3.13	3.12	0.927
**Strategic, Defence & Security Studies (2.2%)**	Other fields	3126	2117	0.833	3.59	3.27	0.074
**General Mathematics (2.2%)**	Other fields	1878	2240	0.636	3.37	3.43	0.371
**Civil Engineering (2.2%)**	Other fields	3434	2152	0.424	3.06	3.17	0.674
**Geology (2.2%)**	Other fields	2839	3402	0.407	3.53	3.48	0.728
**Energy (2.1%)**	Other fields	4554	3374	0.377	3.23	3.26	0.979
**Psychoanalysis (2%)**	Highly related fields	4061	1330	0.136	3.67	3.29	0.136
**Law (1.9%)**	Other fields	1289	1221	1.000	3.19	3.22	0.577
**Marine Biology & Hydrobiology (1.8%)**	Other fields	5379	6136	0.766	3.61	3.7	0.718
**Astronomy & Astrophysics (1.8%)**	Other fields	8979	10830	0.202	3.79	3.74	0.522
**Fisheries (1.8%)**	Other fields	4535	3591	0.504	3.52	3.4	0.307
**Legal & Forensic Medicine (1.5%)**	Highly related fields	1275	1327	0.937	2.93	2.98	0.854
**International Relations (1.5%)**	Other fields	3418	2448	0.558	3.67	3.52	0.460
**Nuclear & Particle Physics (1.3%)**	Other fields	6051	6735	0.529	3.54	3.62	0.318
**Geochemistry & Geophysics (1.2%)**	Other fields	6012	6385	0.538	3.63	3.73	0.087
**Forestry (.96%)**	Other fields	2201	2708	0.488	3.21	3.23	0.833
**Physical Chemistry (.88%)**	Other fields	3764	6576	0.338	3.48	3.51	0.891
**Agronomy & Agriculture (.88%)**	Other fields	5300	3948	0.885	3.45	3.39	0.970
**Paleontology (0%)**	Other fields	.	5643		.	3.72	
**Music (0%)**	Other fields	.	446		.	2.79	
**Industrial Relations (0%)**	Other fields	.	1524		.	3.34	
**Horticulture (0%)**	Other fields	.	2081		.	3.14	
**History of Science, Technology & Medicine (0%)**	Other fields	.	782		.	3.13	
**History (0%)**	Other fields	.	532		.	2.88	
**General Psychology & Cognitive Sciences (0%)**	Highly related fields	.	3365		.	3.48	
**Folklore (0%)**	Other fields	.	310		.	2.61	

© 2022 Ioannidis JPA et al. *JAMA Network Open.*

**Automobile Design & Engineering (0%)**	Other fields	.	860		.	2.69	
**Art Practice, History & Theory (0%)**	Other fields	.	210		.	2.47	
**Architecture (0%)**	Other fields	.	259		.	2.47	
**Accounting (0%)**	Other fields	.	5445		.	3.54	

© 2022 Ioannidis JPA et al. *JAMA Network Open.*

eTable 2.3 : Career-long impact, Funding time current funding citation counts and composite citation indices for each subfield (ordered by percentage funded)

Top-cited US-based researchers: Subfield (perc. funded)	Classification	Citations for funded, median	Citations for non-funded, median	p-value	Composite index for funded, median	Composite index for non-funded, median	p-value
Geriatrics (31%)	Highly related fields	10839	9133	0.217	3.57	3.64	0.845
Bioinformatics (30%)	Highly related fields	14836	9462	0.007	3.69	3.54	0.118
Developmental Biology (29%)	Highly related fields	13168	12541	0.037	3.79	3.82	0.104
Substance Abuse (23%)	Highly related fields	8603	7416	0.121	3.68	3.73	0.211
Medical Informatics (23%)	Highly related fields	4194	4026	0.202	3.5	3.3	0.156
Virology (22%)	Highly related fields	10016	9720	0.304	3.6	3.6	0.955
Biomedical Engineering (22%)	Highly related fields	6113	5174	0.060	3.31	3.37	0.539
Neurology & Neurosurgery (21%)	Highly related fields	11459	10579	0.007	3.77	3.78	0.638
Immunology (20%)	Highly related fields	15484	13119	0.001	3.88	3.77	0.005
Gerontology (20%)	Highly related fields	7573	6230	0.143	3.75	3.62	0.140
Applied Ethics (20%)	Highly related fields	6906	3197	0.002	3.62	3.56	0.418
Public Health (19%)	Highly related fields	8145	7301	0.009	3.71	3.65	0.066
Demography (19%)	Highly related fields	3532	3282	0.949	3.53	3.59	0.423
Oncology & Carcinogenesis (18%)	Highly related fields	14737	13030	<0.001	3.65	3.68	0.053
Gastroenterology & Hepatology (17%)	Highly related fields	12537	9605	0.049	3.72	3.7	0.327
Allergy (16%)	Highly related fields	9374	8456	0.491	3.75	3.75	0.589
Biophysics (16%)	Highly related fields	5430	6020	0.717	3.48	3.52	0.559
Developmental & Child Psychology (16%)	Highly related fields	9033	6918	0.008	3.86	3.74	0.002
Health Policy & Services (16%)	Highly related fields	6978	7567	0.811	3.58	3.61	0.966

© 2022 Ioannidis JPA et al. *JAMA Network Open.*

**Physiology (16%)**	Highly related fields	5972	6492	0.657	3.73	3.72	0.930
**Arthritis & Rheumatology (16%)**	Highly related fields	12811	13497	0.640	3.76	3.79	0.402
**Epidemiology (15%)**	Highly related fields	13964	15301	0.926	3.76	3.84	0.666
**Psychiatry (15%)**	Highly related fields	15417	11068	<0.001	3.9	3.82	0.053
**Genetics & Heredity (15%)**	Highly related fields	15832	11983	0.011	3.64	3.67	0.431
**Emergency & Critical Care Medicine (15%)**	Highly related fields	9522	6216	0.001	3.55	3.47	0.151
**Endocrinology & Metabolism (15%)**	Highly related fields	11919	11565	0.390	3.77	3.78	0.536
**Statistics & Probability (14%)**	Other fields	10685	7455	0.033	3.8	3.7	0.347
**Ophthalmology & Optometry (14%)**	Highly related fields	7668	5621	<0.001	3.6	3.53	0.007
**Microbiology (14%)**	Highly related fields	8839	8261	0.038	3.66	3.65	0.351
**Cardiovascular System & Hematology (14%)**	Highly related fields	15934	13421	0.006	3.76	3.76	0.847
**Respiratory System (13%)**	Highly related fields	9910	10639	0.687	3.61	3.69	0.339
**Clinical Psychology (13%)**	Highly related fields	10228	7700	0.204	3.82	3.74	0.311
**Nuclear Medicine & Medical Imaging (13%)**	Highly related fields	8077	6067	<0.001	3.51	3.45	0.032
**Analytical Chemistry (13%)**	Other fields	7187	4504	<0.001	3.39	3.42	0.754
**Medicinal & Biomolecular Chemistry (12%)**	Highly related fields	4694	3986	0.027	3.19	3.19	0.264
**Nanoscience & Nanotechnology (12%)**	Other fields	12566	12694	0.607	3.65	3.55	0.572
**Urology & Nephrology (12%)**	Highly related fields	11108	8675	0.026	3.69	3.66	0.321
**Optics (12%)**	Other fields	6211	5527	0.024	3.3	3.34	0.862
**Biochemistry & Molecular Biology (12%)**	Highly related fields	11189	9351	<0.001	3.79	3.76	0.143
**Nutrition & Dietetics (11%)**	Highly related fields	9888	6824	0.044	3.7	3.67	0.847
**Experimental Psychology (11%)**	Highly related fields	9469	6183	<0.001	3.91	3.76	0.002
**Rehabilitation (11%)**	Highly related fields	5546	4032	0.028	3.45	3.42	0.206

© 2022 Ioannidis JPA et al. *JAMA Network Open.*

**Toxicology (11%)**	Highly related fields	7828	5089	<0.001	3.47	3.45	0.113
**Drama & Theater (11%)**	Other fields	606	357	0.439	2.8	2.67	0.439
**Obstetrics & Reproductive Medicine (11%)**	Highly related fields	7559	5916	0.022	3.59	3.52	0.237
**Pediatrics (11%)**	Highly related fields	6146	5109	<0.001	3.47	3.41	0.098
**Anesthesiology (11%)**	Highly related fields	6764	4696	0.001	3.48	3.41	0.120
**Pharmacology & Pharmacy (10%)**	Highly related fields	5538	4508	0.002	3.39	3.38	0.287
**Speech-Language Pathology & Audiology (9.9%)**	Highly related fields	2857	3380	0.459	3.38	3.49	0.547
**Organic Chemistry (9.8%)**	Other fields	8436	6344	<0.001	3.7	3.61	0.067
**Environmental & Occupational Health (9.8%)**	Highly related fields	3363	3847	0.545	3.31	3.3	0.772
**Family Studies (8.9%)**	Other fields	4688	2976	0.381	3.54	3.5	0.943
**Development Studies (8.3%)**	Other fields	3741	3565	0.917	3.48	3.57	0.296
**Mycology & Parasitology (8.2%)**	Highly related fields	5086	4661	0.593	3.45	3.46	0.960
**Nursing (7.9%)**	Highly related fields	2865	1561	<0.001	3.17	3.07	0.002
**Surgery (7.9%)**	Highly related fields	8494	6014	<0.001	3.46	3.41	0.232
**Tropical Medicine (7.8%)**	Highly related fields	7167	4979	0.046	3.55	3.39	0.173
**General Chemistry (7.8%)**	Other fields	8763	4364	<0.001	3.73	3.34	0.005
**Complementary & Alternative Medicine (7.4%)**	Highly related fields	3645	1421	0.010	3.13	2.86	0.043
**General & Internal Medicine (7.1%)**	Highly related fields	6242	3586	<0.001	3.32	3.2	0.015
**Biotechnology (7%)**	Highly related fields	6265	5049	0.166	3.5	3.31	0.171
**Microscopy (6.7%)**	Highly related fields	10312	5644	0.280	3.78	3.46	0.135
**Acoustics (6.5%)**	Other fields	3213	2976	0.284	3.34	3.42	0.441
**Sociology (6.4%)**	Other fields	4332	3815	0.527	3.7	3.68	0.790
**Otorhinolaryngology (6.3%)**	Highly related fields	4490	3530	0.024	3.37	3.36	0.213
**Behavioral Science & Comparative Psychology (6.3%)**	Highly related fields	6282	4557	0.137	3.79	3.71	0.867

**Orthopedics (5.9%)**	Highly related fields	9267	5580	<0.001	3.65	3.5	0.003
**Sport Sciences (5.9%)**	Highly related fields	7114	5198	0.404	3.45	3.53	0.788
**Social Work (5.7%)**	Other fields	2382	1638	0.308	3.28	3.24	0.245
**Economic Theory (5.6%)**	Other fields	3859	2701	0.772	3.51	3.61	0.386
**Evolutionary Biology (5.5%)**	Other fields	9058	6924	0.017	3.89	3.8	0.151
**Dermatology & Venereal Diseases (5.5%)**	Highly related fields	8804	5984	0.008	3.67	3.61	0.211
**Artificial Intelligence & Image Processing (5.5%)**	Other fields	4867	4293	0.102	3.44	3.35	0.224
**Environmental Sciences (5.5%)**	Other fields	7291	4504	0.015	3.44	3.37	0.340
**Plant Biology & Botany (5.2%)**	Other fields	9646	5794	<0.001	3.72	3.51	0.001
**Pathology (5.1%)**	Highly related fields	10145	9393	0.891	3.55	3.63	0.584
**Chemical Physics (4.8%)**	Other fields	13002	8433	0.011	3.93	3.79	0.066
**Industrial Engineering & Automation (4.8%)**	Other fields	5400	4234	0.149	3.52	3.44	0.337
**Entomology (4.8%)**	Other fields	7706	3312	<0.001	3.76	3.46	0.006
**Criminology (4.6%)**	Other fields	4788	3799	0.089	3.61	3.6	0.582
**Religions & Theology (4.4%)**	Other fields	911	463	0.106	3.06	2.85	0.210
**Sport, Leisure & Tourism (4.3%)**	Other fields	3629	3732	0.829	3.61	3.57	0.829
**Distributed Computing (4.3%)**	Other fields	6213	3356	0.012	3.23	3.13	0.836
**Dentistry (4.3%)**	Highly related fields	4451	3419	0.087	3.45	3.39	0.614
**Social Sciences Methods (3.9%)**	Other fields	30480	5254	0.073	4.39	3.72	0.040
**Science Studies (3.8%)**	Other fields	4961	4004	0.549	3.59	3.69	0.257
**Logistics & Transportation (3.8%)**	Other fields	2116	2775	0.319	3.16	3.28	0.024
**Veterinary Sciences (3.8%)**	Highly related fields	5021	3105	0.003	3.4	3.31	0.149
**Social Psychology (3.7%)**	Highly related fields	11577	7178	0.086	3.95	3.8	0.083
**Anatomy & Morphology (3.7%)**	Highly related fields	3295	1505	0.156	2.95	3.06	0.272

© 2022 Ioannidis JPA et al. *JAMA Network Open.*

**Applied Mathematics (3.6%)**	Other fields	6279	4521	0.745	3.46	3.61	0.789
**Polymers (3.6%)**	Other fields	7233	5559	0.102	3.57	3.49	0.762
**Information & Library Sciences (3.4%)**	Other fields	1095	1299	0.631	3.12	3.16	0.893
**Communication & Media Studies (3.2%)**	Other fields	2875	2784	0.325	3.54	3.49	0.479
**Mining & Metallurgy (3.2%)**	Other fields	5030	1020	0.605	3.25	2.76	0.661
**General Clinical Medicine (3.1%)**	Highly related fields	10192	2712	0.018	3.63	3.08	0.013
**Marketing (3.1%)**	Other fields	6267	5797	0.606	3.72	3.64	0.714
**Economics (3.1%)**	Other fields	9915	4739	0.001	3.96	3.71	0.009
**Software Engineering (3.1%)**	Other fields	6139	4522	0.308	3.59	3.46	0.275
**Ornithology (3%)**	Other fields	2139	3168	0.462	3.37	3.43	0.674
**Finance (3%)**	Other fields	6458	4901	0.700	3.69	3.59	0.418
**Human Factors (3%)**	Highly related fields	4766	5142	0.441	3.67	3.58	0.155
**Agricultural Economics & Policy (2.7%)**	Other fields	3180	4084	0.265	3.58	3.58	1.000
**Computer Hardware & Architecture (2.6%)**	Other fields	4513	4437	0.696	3.26	3.32	0.990
**Networking & Telecommunications (2.6%)**	Other fields	4203	3679	0.306	3.16	3.25	0.629
**Political Science & Public Administration (2.6%)**	Other fields	5295	3497	0.089	3.67	3.64	0.661
**Food Science (2.6%)**	Other fields	4023	4018	0.558	3.23	3.41	0.532
**Education (2.6%)**	Other fields	4463	2375	<0.001	3.64	3.36	0.001
**Dairy & Animal Science (2.4%)**	Other fields	3802	3067	0.299	3.35	3.25	0.988
**Econometrics (2.4%)**	Other fields	17980	6910	0.302	3.88	3.97	0.902
**Mechanical Engineering & Transports (2.3%)**	Other fields	3934	2911	0.739	3.15	3.36	0.127

© 2022 Ioannidis JPA et al. *JAMA Network Open.*

**Strategic, Defence & Security Studies (2.2%)**	Other fields	3126	2117	0.773	3.57	3.27	0.103
**Electrical & Electronic Engineering (2.2%)**	Other fields	3080	2546	0.350	2.89	3.04	0.279
**Geography (2.2%)**	Other fields	3516	3345	0.872	3.6	3.68	0.453
**Chemical Engineering (2.1%)**	Other fields	3832	4079	0.802	3.37	3.43	0.951
**General Physics (2%)**	Other fields	7034	5581	0.232	3.62	3.57	0.744
**Optoelectronics & Photonics (2%)**	Other fields	3883	2901	0.278	3.15	3.11	0.506
**Psychoanalysis (2%)**	Highly related fields	4061	1330	0.136	3.67	3.29	0.136
**Design Practice & Management (2%)**	Other fields	5309	3034	0.787	3.37	3.27	0.588
**Operations Research (2%)**	Other fields	4628	4221	0.708	3.68	3.6	0.785
**Environmental Engineering (2%)**	Other fields	4128	4209	0.916	3.4	3.44	0.986
**Information Systems (1.9%)**	Other fields	9535	5645	0.137	3.69	3.58	0.321
**Literary Studies (1.9%)**	Other fields	591	349	0.355	2.92	2.7	0.349
**Cultural Studies (1.9%)**	Other fields	1102	794	0.585	3.29	3.1	0.352
**Languages & Linguistics (1.8%)**	Other fields	2577	1838	0.408	3.62	3.43	0.194
**Fisheries (1.8%)**	Other fields	6517	3591	0.265	3.59	3.4	0.174
**Archaeology (1.8%)**	Other fields	2125	1922	0.796	3.29	3.31	0.666
**Computation Theory & Mathematics (1.7%)**	Other fields	3448	4911	0.699	3.68	3.63	0.991
**Numerical & Computational Mathematics (1.7%)**	Other fields	9914	3498	0.062	3.95	3.54	0.085
**Urban & Regional Planning (1.6%)**	Other fields	3463	2360	0.570	3.37	3.54	0.281
**Anthropology (1.6%)**	Other fields	4446	3247	0.368	3.61	3.61	0.704
**Materials (1.6%)**	Other fields	7834	3448	0.001	3.67	3.28	0.004
**Fluids & Plasmas (1.6%)**	Other fields	13640	6643	0.013	3.91	3.73	0.080
**General Mathematics (1.6%)**	Other fields	1729	2247	0.161	3.37	3.43	0.084

© 2022 Ioannidis JPA et al. *JAMA Network Open.*

**Building & Construction (1.6%)**	Other fields	5601	3016	0.120	3.46	3.32	0.939
**Philosophy (1.5%)**	Other fields	5532	1353	0.020	4.02	3.38	0.023
**Meteorology & Atmospheric Sciences (1.5%)**	Other fields	7974	8765	0.550	3.74	3.74	0.956
**Aerospace & Aeronautics (1.5%)**	Other fields	2674	1884	0.500	3.07	3.13	0.681
**Energy (1.4%)**	Other fields	4706	3378	0.410	3.13	3.26	0.586
**Geological & Geomatics Engineering (1.4%)**	Other fields	12243	4575	0.028	3.84	3.45	0.176
**Ecology (1.3%)**	Other fields	6631	7570	0.697	4.02	3.75	0.195
**Oceanography (1.1%)**	Other fields	10377	4641	0.058	3.78	3.58	0.319
**Business & Management (.95%)**	Other fields	7503	6052	0.388	3.86	3.7	0.414
**Law (.94%)**	Other fields	1025	1223	0.524	3.23	3.22	0.961
**Applied Physics (.94%)**	Other fields	8020	6408	0.484	3.64	3.53	0.151
**Marine Biology & Hydrobiology (.91%)**	Other fields	11873	6074	0.219	4.02	3.69	0.221
**Physical Chemistry (.88%)**	Other fields	3764	6576	0.338	3.48	3.51	0.891
**Nuclear & Particle Physics (.78%)**	Other fields	7802	6724	0.538	3.63	3.62	0.871
**Astronomy & Astrophysics (.76%)**	Other fields	8250	10762	0.465	3.88	3.74	0.217
**Civil Engineering (.73%)**	Other fields	3434	2152	0.445	3.39	3.16	0.865
**Geochemistry & Geophysics (.71%)**	Other fields	5389	6405	0.404	3.54	3.73	0.031
**Agronomy & Agriculture (.66%)**	Other fields	7813	3940	0.540	3.69	3.39	0.475
**Inorganic & Nuclear Chemistry (.63%)**	Other fields	4341	5884	0.418	3.4	3.55	0.388
**Forestry (.48%)**	Other fields	1063	2711	0.136	3.08	3.23	0.353
**Zoology (0%)**	Other fields	.	2100		.	3.21	
**Paleontology (0%)**	Other fields	.	5643		.	3.72	
**Music (0%)**	Other fields	.	446		.	2.79	
**Mathematical Physics (0%)**	Other fields	.	2774		.	3.49	
**Legal & Forensic Medicine (0%)**	Highly related fields	.	1326		.	2.97	

© 2022 Ioannidis JPA et al. *JAMA Network Open.*

**International Relations (0%)**	Other fields	.	2518			.	3.52	
**Industrial Relations (0%)**	Other fields	.	1524			.	3.34	
**Horticulture (0%)**	Other fields	.	2081			.	3.14	
**History of Social Sciences (0%)**	Other fields	.	1740			.	3.31	
**History of Science, Technology & Medicine (0%)**	Other fields	.	782			.	3.13	
**History (0%)**	Other fields	.	532			.	2.88	
**Geology (0%)**	Other fields	.	3402			.	3.48	
**General Psychology & Cognitive Sciences (0%)**	Highly related fields	.	3365			.	3.48	
**Gender Studies (0%)**	Highly related fields	.	2515			.	3.51	
**Folklore (0%)**	Other fields	.	310			.	2.61	
**Classics (0%)**	Other fields	.	536			.	2.92	
**Automobile Design & Engineering (0%)**	Other fields	.	860			.	2.69	
**Art Practice, History & Theory (0%)**	Other fields	.	210			.	2.47	
**Architecture (0%)**	Other fields	.	259			.	2.47	
**Accounting (0%)**	Other fields	.	5445			.	3.54	

© 2022 Ioannidis JPA et al. *JAMA Network Open.*

eTable 2.4 : Recent year impact, Funding time any funding citation counts and composite citation indices for each subfield (ordered by percentage funded)

Top-cited US-based researchers: Subfield (perc. funded)	Classification	Citations for funded, median	Citations for non-funded, median	p-value	Composite index for funded, median	Composite index for non-funded, median	p-value
**Geriatrics (88%)**	Highly related fields	1567	1003	0.015	3.11	2.97	0.033
**Gerontology (87%)**	Highly related fields	1210	536	0.002	3.16	2.98	0.023
**Substance Abuse (86%)**	Highly related fields	1155	919	0.048	3.09	3.06	0.472
**Developmental Biology (86%)**	Highly related fields	1826	2005	0.102	3.15	3.07	<0.001
**Endocrinology & Metabolism (83%)**	Highly related fields	1579	1290	0.003	3.16	3.05	<0.001
**Immunology (83%)**	Highly related fields	1825	2049	0.271	3.12	2.99	<0.001
**Neurology & Neurosurgery (82%)**	Highly related fields	1561	1311	<0.001	3.14	3.03	<0.001
**Biochemistry & Molecular Biology (81%)**	Highly related fields	985	756	<0.001	2.98	2.84	<0.001
**Virology (81%)**	Highly related fields	1204	1158	0.486	2.86	2.82	0.018
**Psychiatry (81%)**	Highly related fields	1724	1174	<0.001	3.2	3.1	0.023
**Genetics & Heredity (79%)**	Highly related fields	1477	1277	0.097	2.85	2.82	0.103
**Allergy (75%)**	Highly related fields	1704	1215	0.019	3.19	3.02	0.059
**Biophysics (74%)**	Highly related fields	715	479	0.001	2.83	2.68	<0.001
**Epidemiology (74%)**	Highly related fields	2056	1906	0.477	3.14	3.18	0.688
**Biomedical Engineering (74%)**	Highly related fields	999	713	<0.001	2.86	2.73	<0.001
**Arthritis & Rheumatology (73%)**	Highly related fields	2153	1718	0.004	3.18	3.08	0.026
**Oncology & Carcinogenesis (72%)**	Highly related fields	2165	1827	<0.001	3.03	2.93	<0.001
**Developmental & Child Psychology (72%)**	Highly related fields	1132	761	<0.001	3.2	3.1	0.007

© 2022 Ioannidis JPA et al. *JAMA Network Open.*

**Medical Informatics (71%)**	Highly related fields	703	586	0.088	2.71	2.7	0.625
**Physiology (71%)**	Highly related fields	763	517	<0.001	2.96	2.94	0.404
**Public Health (71%)**	Highly related fields	1243	1004	0.001	3.13	3.08	0.046
**Gastroenterology & Hepatology (69%)**	Highly related fields	1415	1151	<0.001	3	2.91	<0.001
**Urology & Nephrology (67%)**	Highly related fields	1222	1043	0.001	2.95	2.81	<0.001
**Respiratory System (67%)**	Highly related fields	1550	1363	0.001	2.97	2.93	0.031
**Pediatrics (67%)**	Highly related fields	831	598	<0.001	2.72	2.64	<0.001
**Bioinformatics (67%)**	Highly related fields	2402	1840	0.089	3.03	2.98	0.237
**Cardiovascular System & Hematology (66%)**	Highly related fields	1799	1762	0.037	3.06	2.98	<0.001
**Health Policy & Services (64%)**	Highly related fields	1131	853	0.021	3.01	2.92	0.005
**Experimental Psychology (64%)**	Highly related fields	981	724	<0.001	3.21	3.15	0.025
**Emergency & Critical Care Medicine (63%)**	Highly related fields	1313	794	<0.001	2.98	2.76	<0.001
**Clinical Psychology (63%)**	Highly related fields	1270	895	<0.001	3.17	3.14	0.093
**Ophthalmology & Optometry (62%)**	Highly related fields	978	688	<0.001	2.87	2.73	<0.001
**Family Studies (62%)**	Other fields	529	310	0.007	3.04	2.94	0.323
**Speech-Language Pathology & Audiology (61%)**	Highly related fields	465	597	0.273	2.87	2.9	0.834
**Rehabilitation (61%)**	Highly related fields	784	677	0.144	2.84	2.84	0.706
**Obstetrics & Reproductive Medicine (60%)**	Highly related fields	874	689	<0.001	2.84	2.74	<0.001
**Toxicology (60%)**	Highly related fields	1051	674	<0.001	2.86	2.76	<0.001
**Environmental & Occupational Health (59%)**	Highly related fields	565	491	0.216	2.65	2.58	0.107
**Nutrition & Dietetics (59%)**	Highly related fields	1268	921	<0.001	3.12	3.01	0.006
**Microbiology (59%)**	Highly related fields	1339	1275	0.169	3.02	2.96	<0.001
**Nursing (59%)**	Highly related fields	345	211	<0.001	2.49	2.47	0.085
**Demography (58%)**	Highly related fields	533	339	0.033	3.06	2.99	0.642

© 2022 Ioannidis JPA et al. *JAMA Network Open.*

**Analytical Chemistry (58%)**	Other fields	881	626	<0.001	2.81	2.77	0.085
**Nuclear Medicine & Medical Imaging (57%)**	Highly related fields	960	727	<0.001	2.75	2.65	<0.001
**Organic Chemistry (56%)**	Other fields	1023	848	0.005	2.98	2.83	<0.001
**Pharmacology & Pharmacy (53%)**	Highly related fields	718	519	<0.001	2.75	2.62	<0.001
**Medicinal & Biomolecular Chemistry (51%)**	Highly related fields	801	591	<0.001	2.68	2.58	<0.001
**Complementary & Alternative Medicine (50%)**	Highly related fields	520	242	<0.001	2.65	2.53	0.273
**Behavioral Science & Comparative Psychology (49%)**	Highly related fields	727	614	0.085	3.11	3.07	0.169
**Anesthesiology (48%)**	Highly related fields	743	602	<0.001	2.77	2.64	<0.001
**Dentistry (48%)**	Highly related fields	663	423	<0.001	2.73	2.67	0.002
**Otorhinolaryngology (47%)**	Highly related fields	528	507	0.442	2.66	2.57	0.005
**Tropical Medicine (46%)**	Highly related fields	908	634	0.001	2.77	2.67	0.001
**Applied Ethics (46%)**	Highly related fields	686	489	0.081	3.04	2.92	0.461
**General & Internal Medicine (46%)**	Highly related fields	776	536	<0.001	2.73	2.49	<0.001
**Statistics & Probability (45%)**	Other fields	1447	758	<0.001	3.18	3.09	0.093
**Surgery (44%)**	Highly related fields	1034	686	<0.001	2.75	2.61	<0.001
**Mycology & Parasitology (44%)**	Highly related fields	695	778	0.321	2.79	2.82	0.181
**Dermatology & Venereal Diseases (42%)**	Highly related fields	1012	673	<0.001	2.87	2.73	<0.001
**History of Social Sciences (41%)**	Other fields	184	262	0.526	2.75	2.76	0.696
**Biotechnology (41%)**	Highly related fields	1046	945	0.343	2.94	2.82	0.112
**General Chemistry (40%)**	Other fields	806	728	0.052	2.83	2.64	<0.001
**Sport Sciences (38%)**	Highly related fields	1102	898	0.020	3.01	3.02	0.710
**Social Psychology (38%)**	Highly related fields	1363	820	<0.001	3.39	3.21	<0.001
**Acoustics (37%)**	Other fields	505	436	0.072	2.77	2.77	0.990
**Optics (32%)**	Other fields	987	907	0.100	2.77	2.76	0.130
**Orthopedics (31%)**	Highly related fields	1159	810	<0.001	2.95	2.83	<0.001

© 2022 Ioannidis JPA et al. *JAMA Network Open.*

**Chemical Physics (30%)**	Other fields	1629	1075	<0.001	3.18	3.08	0.002
**Evolutionary Biology (30%)**	Other fields	1011	916	0.003	3.24	3.13	0.005
**Nanoscience & Nanotechnology (29%)**	Other fields	2493	2400	0.121	3.22	3.12	0.001
**Sociology (28%)**	Other fields	467	415	0.132	3.05	3.11	0.471
**Pathology (28%)**	Highly related fields	1232	1032	0.030	2.89	2.8	0.105
**Plant Biology & Botany (27%)**	Other fields	1183	863	<0.001	3.02	2.94	0.001
**General Clinical Medicine (27%)**	Highly related fields	571	276	<0.001	2.57	2.3	0.008
**Veterinary Sciences (27%)**	Highly related fields	532	377	<0.001	2.59	2.55	0.027
**Environmental Sciences (26%)**	Other fields	1648	1154	0.002	3.16	3.08	0.089
**Social Sciences Methods (25%)**	Other fields	1099	719	0.003	3.47	3.15	0.007
**Criminology (25%)**	Other fields	805	474	<0.001	3.14	3.04	0.026
**Microscopy (24%)**	Highly related fields	1146	603	0.027	2.9	2.74	0.141
**Inorganic & Nuclear Chemistry (23%)**	Other fields	923	690	0.142	2.89	2.76	0.011
**Social Work (23%)**	Other fields	468	271	0.001	2.88	2.71	0.004
**Polymers (21%)**	Other fields	1353	791	<0.001	2.99	2.89	0.074
**Drama & Theater (20%)**	Other fields	52.5	50	1.000	2.12	2.1	0.602
**Economics (19%)**	Other fields	746	597	0.001	3.2	3.13	0.033
**Food Science (19%)**	Other fields	1271	828	0.026	3.07	2.96	0.052
**Artificial Intelligence & Image Processing (19%)**	Other fields	1028	867	0.001	2.87	2.86	0.224
**Distributed Computing (18%)**	Other fields	665	450	0.010	2.52	2.39	0.332
**Industrial Engineering & Automation (17%)**	Other fields	860	714	0.017	2.96	2.86	0.099
**Entomology (17%)**	Other fields	918	503	<0.001	3.04	2.8	<0.001
**Design Practice & Management (16%)**	Other fields	577	515	0.839	2.83	2.77	0.973
**Human Factors (16%)**	Highly related fields	994	712	0.024	3.01	3.02	0.167
**Information & Library Sciences (16%)**	Other fields	257	173	0.117	2.73	2.54	0.098
**Fluids & Plasmas (16%)**	Other fields	943	802	0.198	3.11	3.08	0.434

© 2022 Ioannidis JPA et al. *JAMA Network Open.*

**General Psychology & Cognitive Sciences (16%)**	Highly related fields	402	589	0.631	2.94	2.99	0.882
**Urban & Regional Planning (15%)**	Other fields	499	422	0.348	3.3	3.13	0.162
**Education (15%)**	Other fields	616	407	<0.001	2.95	2.93	0.299
**Psychoanalysis (15%)**	Highly related fields	151	121	0.269	2.33	2.4	0.547
**Development Studies (15%)**	Other fields	720	605	0.633	3.13	3.14	0.891
**Anatomy & Morphology (14%)**	Highly related fields	260	180	0.374	2.44	2.4	0.656
**Geography (14%)**	Other fields	465	401	0.128	3.29	3.05	0.071
**Ornithology (14%)**	Other fields	287	361	0.569	2.75	2.68	0.752
**History of Science, Technology & Medicine (13%)**	Other fields	91.5	68	0.932	2.32	2.27	1.000
**Gender Studies (13%)**	Highly related fields	345	360	0.497	3.05	3.02	0.734
**Dairy & Animal Science (13%)**	Other fields	576	495	0.140	2.67	2.64	0.715
**Logistics & Transportation (13%)**	Other fields	481	604	0.265	2.89	2.96	0.130
**Economic Theory (13%)**	Other fields	308	358	0.427	3	2.86	0.525
**Optoelectronics & Photonics (12%)**	Other fields	469	368	0.040	2.44	2.41	0.524
**Software Engineering (12%)**	Other fields	485	630	0.183	2.7	2.8	0.209
**Marine Biology & Hydrobiology (12%)**	Other fields	920	943	0.963	3.01	3.08	0.258
**Anthropology (12%)**	Other fields	525	400	0.218	3.01	3	0.564
**Numerical & Computational Mathematics (12%)**	Other fields	995	515	0.061	2.93	3.01	0.950
**Geological & Geomatics Engineering (11%)**	Other fields	1035	977	0.332	3.16	3.07	0.255
**Electrical & Electronic Engineering (11%)**	Other fields	557	450	0.067	2.55	2.53	0.464
**Physical Chemistry (10%)**	Other fields	2102	1328	0.043	2.94	2.98	0.813
**Econometrics (10%)**	Other fields	898	1069	0.668	3.3	3.5	0.355
**Computer Hardware & Architecture (10%)**	Other fields	726	496	0.028	2.62	2.51	0.035

© 2022 Ioannidis JPA et al. *JAMA Network Open.*

**Communication & Media Studies (10%)**	Other fields	520	446	0.388	3.13	3.07	0.452
**Environmental Engineering (10%)**	Other fields	1230	754	<0.001	3.01	2.97	0.215
**Networking & Telecommunications (10%)**	Other fields	549	564	0.759	2.61	2.63	0.635
**Archaeology (9.9%)**	Other fields	388	350	0.645	2.75	2.79	0.351
**Zoology (9.8%)**	Other fields	284	345	0.703	2.61	2.57	0.901
**Religions & Theology (9.8%)**	Other fields	94.5	70	0.340	2.28	2.26	0.220
**Chemical Engineering (9.6%)**	Other fields	1385	854	0.079	3.08	2.96	0.278
**Ecology (9.4%)**	Other fields	1160	1222	0.989	3.24	3.15	0.131
**Materials (9.3%)**	Other fields	831	716	0.036	2.89	2.82	0.307
**General Physics (9.1%)**	Other fields	751	661	0.149	2.86	2.82	0.842
**Oceanography (8.9%)**	Other fields	796	641	0.104	3.15	2.92	0.313
**Building & Construction (8.8%)**	Other fields	1431	843	0.044	3.08	3.05	0.536
**Sport, Leisure & Tourism (8.7%)**	Other fields	550	675	0.275	3.1	3.09	0.959
**Applied Mathematics (8.7%)**	Other fields	1594	930	0.028	3.45	3.13	0.088
**Mechanical Engineering & Transports (8.6%)**	Other fields	562	556	0.776	2.81	2.86	0.224
**Strategic, Defence & Security Studies (8.5%)**	Other fields	602	373	0.157	2.84	2.86	0.661
**Languages & Linguistics (8.1%)**	Other fields	503	360	0.186	3.33	3.05	0.106
**Computation Theory & Mathematics (8%)**	Other fields	598	517	0.577	2.97	2.94	0.678
**Marketing (7.9%)**	Other fields	947	678	0.571	3.25	3.09	0.161
**Law (7.6%)**	Other fields	128	142	0.918	2.57	2.47	0.591
**Meteorology & Atmospheric Sciences (7.4%)**	Other fields	1419	1357	0.371	3.12	3.13	0.431
**Applied Physics (7.3%)**	Other fields	948	876	0.148	2.89	2.82	0.106
**Operations Research (7.2%)**	Other fields	530	707	0.492	3.12	3.15	0.780
**Finance (7.2%)**	Other fields	634	773	0.231	3.01	3.12	0.099
**Political Science & Public Administration (7%)**	Other fields	601	442	0.122	3.18	3.06	0.174
**Forestry (6.9%)**	Other fields	466	492	0.654	2.81	2.64	0.354

© 2022 Ioannidis JPA et al. *JAMA Network Open.*

**Business & Management (6.6%)**	Other fields	965	776	0.162	3.19	3.17	0.607
**Literary Studies (6.5%)**	Other fields	45	44	0.836	2.12	2	0.921
**General Mathematics (6.1%)**	Other fields	229	302	0.110	2.84	2.85	0.511
**Aerospace & Aeronautics (6.1%)**	Other fields	345	289	0.533	2.49	2.5	0.320
**Geology (6.1%)**	Other fields	381	505	0.388	2.73	2.89	0.218
**History (5.9%)**	Other fields	57	47	0.278	2.17	2.07	0.239
**Information Systems (5.9%)**	Other fields	880	900	0.615	3.07	3.06	0.699
**Energy (5.9%)**	Other fields	953	780	0.032	2.91	2.87	0.655
**Science Studies (5.7%)**	Other fields	415	552	0.477	2.99	3.16	0.155
**Legal & Forensic Medicine (5.7%)**	Highly related fields	245	209	0.847	2.31	2.29	0.729
**Cultural Studies (5.6%)**	Other fields	84	128	0.189	2.46	2.54	0.407
**Astronomy & Astrophysics (5.5%)**	Other fields	1406	1769	0.427	3.11	3.14	0.995
**Accounting (5.5%)**	Other fields	809	687	0.396	3.04	3.1	0.827
**Music (5.4%)**	Other fields	49	52	0.788	2.15	2.06	0.893
**Geochemistry & Geophysics (5.4%)**	Other fields	729	874	0.329	3.16	3.15	0.608
**Agronomy & Agriculture (5.3%)**	Other fields	1018	765	0.154	3.01	2.89	0.110
**Nuclear & Particle Physics (4.9%)**	Other fields	830	757	0.865	2.93	2.9	0.391
**Fisheries (4.8%)**	Other fields	491	618	0.399	2.69	2.81	0.334
**Classics (4.8%)**	Other fields	67	40.5	0.186	2.32	2.03	0.186
**Mining & Metallurgy (4.7%)**	Other fields	241	252	0.908	2.54	2.36	0.686
**Paleontology (4.4%)**	Other fields	829	803	0.832	3.08	3.1	0.560
**Agricultural Economics & Policy (4.3%)**	Other fields	911	732	0.617	3.23	3.08	0.814
**Philosophy (3.9%)**	Other fields	415	201	0.246	3.16	2.85	0.118
**International Relations (3.6%)**	Other fields	389	343	0.791	2.92	3.01	0.402
**Civil Engineering (2.9%)**	Other fields	642	561	0.681	2.82	2.84	0.763
**Mathematical Physics (0%)**	Other fields	.	358		.	2.88	
**Industrial Relations (0%)**	Other fields	.	218		.	2.87	
**Horticulture (0%)**	Other fields	.	396		.	2.64	
**Folklore (0%)**	Other fields	.	45		.	2.1	

© 2022 Ioannidis JPA et al. *JAMA Network Open.*

**Automobile Design & Engineering (0%)**	Other fields	.	122		.	2.18	
**Art Practice, History & Theory (0%)**	Other fields	.	36		.	1.88	
**Architecture (0%)**	Other fields	.	74		.	2.12	

© 2022 Ioannidis JPA et al. *JAMA Network Open.*

eTable 2.5 : Recent year impact, Funding time recent funding citation counts and composite citation indices for each subfield (ordered by percentage funded)

Top-cited US-based researchers: Subfield (perc. funded)	Classification	Citations for funded, median	Citations for non-funded, median	p-value	Composite index for funded, median	Composite index for non-funded, median	p-value
**Geriatrics (55%)**	Highly related fields	1681	1190	0.154	3.09	3.07	0.655
**Substance Abuse (50%)**	Highly related fields	1208	1014	0.353	3.06	3.12	0.113
**Developmental Biology (49%)**	Highly related fields	1905	1789	0.057	3.14	3.14	0.710
**Medical Informatics (48%)**	Highly related fields	712	624	0.171	2.73	2.7	0.690
**Bioinformatics (46%)**	Highly related fields	3039	1714	<0.001	3.04	2.98	0.128
**Virology (45%)**	Highly related fields	1171	1229	0.577	2.86	2.84	0.695
**Immunology (45%)**	Highly related fields	1917	1827	0.358	3.11	3.09	0.272
**Gerontology (44%)**	Highly related fields	1240	1005	0.044	3.16	3.12	0.723
**Biomedical Engineering (44%)**	Highly related fields	990	806	0.001	2.84	2.83	0.194
**Neurology & Neurosurgery (42%)**	Highly related fields	1581	1462	0.004	3.13	3.12	0.879
**Oncology & Carcinogenesis (40%)**	Highly related fields	2297	1905	<0.001	3.02	2.98	0.057
**Public Health (38%)**	Highly related fields	1260	1095	0.100	3.13	3.1	0.724
**Genetics & Heredity (38%)**	Highly related fields	1953	1279	<0.001	2.86	2.84	0.137
**Emergency & Critical Care Medicine (38%)**	Highly related fields	1438	975	<0.001	2.98	2.82	0.001
**Epidemiology (36%)**	Highly related fields	2172	1906	0.198	3.14	3.15	0.938
**Psychiatry (36%)**	Highly related fields	1746	1523	0.002	3.18	3.18	0.647
**Gastroenterology & Hepatology (35%)**	Highly related fields	1440	1259	0.019	3.01	2.95	0.080
**Analytical Chemistry (35%)**	Other fields	867	707	<0.001	2.8	2.79	0.404
**Arthritis & Rheumatology (34%)**	Highly related fields	1818	2052	0.637	3.11	3.13	0.754

© 2022 Ioannidis JPA et al. *JAMA Network Open.*

**Allergy (34%)**	Highly related fields	1704	1344	0.114	3.15	3.17	0.822
**Developmental & Child Psychology (33%)**	Highly related fields	1244	892	<0.001	3.18	3.16	0.213
**Endocrinology & Metabolism (33%)**	Highly related fields	1458	1542	0.667	3.13	3.15	0.394
**Respiratory System (33%)**	Highly related fields	1531	1438	0.190	2.95	2.95	0.535
**Nuclear Medicine & Medical Imaging (32%)**	Highly related fields	1057	786	<0.001	2.78	2.68	<0.001
**Cardiovascular System & Hematology (32%)**	Highly related fields	1990	1723	<0.001	3.07	3.01	0.003
**Health Policy & Services (31%)**	Highly related fields	1183	1013	0.041	3.03	2.95	0.051
**Microbiology (31%)**	Highly related fields	1377	1280	0.151	2.99	2.98	0.211
**Toxicology (31%)**	Highly related fields	1129	777	<0.001	2.85	2.79	0.021
**Pediatrics (31%)**	Highly related fields	873	698	<0.001	2.73	2.67	0.007
**Biophysics (31%)**	Highly related fields	707	587	0.352	2.81	2.79	0.553
**Rehabilitation (30%)**	Highly related fields	786	700	0.277	2.82	2.86	0.360
**Urology & Nephrology (30%)**	Highly related fields	1279	1108	0.016	2.97	2.85	<0.001
**Ophthalmology & Optometry (30%)**	Highly related fields	1062	754	<0.001	2.91	2.77	<0.001
**Physiology (29%)**	Highly related fields	765	628	0.036	2.91	2.96	0.765
**Biochemistry & Molecular Biology (29%)**	Highly related fields	1064	888	<0.001	2.97	2.93	0.504
**Clinical Psychology (28%)**	Highly related fields	1307	1078	0.007	3.17	3.15	0.392
**Medicinal & Biomolecular Chemistry (28%)**	Highly related fields	780	672	0.010	2.64	2.62	0.353
**Obstetrics & Reproductive Medicine (28%)**	Highly related fields	878	747	0.031	2.81	2.79	0.338
**Anesthesiology (27%)**	Highly related fields	798	627	<0.001	2.76	2.68	0.059
**Tropical Medicine (27%)**	Highly related fields	897	697	0.022	2.75	2.69	0.292
**Pharmacology & Pharmacy (25%)**	Highly related fields	772	588	<0.001	2.75	2.66	0.002
**Nutrition & Dietetics (25%)**	Highly related fields	1386	995	<0.001	3.16	3.04	0.179

© 2022 Ioannidis JPA et al. *JAMA Network Open.*

**Nursing (25%)**	Highly related fields	376	250	<0.001	2.52	2.46	0.007
**Demography (25%)**	Highly related fields	533	446	0.149	3.07	2.97	0.454
**Speech-Language Pathology & Audiology (25%)**	Highly related fields	485	490	0.707	2.86	2.88	0.449
**Statistics & Probability (24%)**	Other fields	1532	835	<0.001	3.19	3.12	0.226
**Family Studies (24%)**	Other fields	609	434	0.005	3.14	2.94	0.003
**Environmental & Occupational Health (23%)**	Highly related fields	626	508	0.335	2.65	2.62	0.753
**Experimental Psychology (22%)**	Highly related fields	1078	825	<0.001	3.17	3.2	0.425
**Applied Ethics (22%)**	Highly related fields	847	493	0.010	3.06	2.94	0.204
**Organic Chemistry (22%)**	Other fields	1158	856	<0.001	2.97	2.9	0.038
**Optics (22%)**	Other fields	985	918	0.146	2.76	2.77	0.756
**Microscopy (21%)**	Highly related fields	1129	608	0.045	2.8	2.74	0.291
**General & Internal Medicine (21%)**	Highly related fields	777	613	<0.001	2.71	2.55	<0.001
**Biotechnology (21%)**	Highly related fields	1022	959	0.997	2.91	2.85	0.742
**Otorhinolaryngology (20%)**	Highly related fields	605	497	0.042	2.67	2.6	0.156
**Surgery (20%)**	Highly related fields	1074	752	<0.001	2.75	2.65	0.001
**General Chemistry (19%)**	Other fields	903	728	0.046	2.82	2.68	0.122
**Mycology & Parasitology (18%)**	Highly related fields	734	755	0.825	2.88	2.79	0.641
**Nanoscience & Nanotechnology (18%)**	Other fields	2394	2441	0.351	3.2	3.14	0.052
**Behavioral Science & Comparative Psychology (17%)**	Highly related fields	796	668	0.240	3.04	3.1	0.863
**Dermatology & Venereal Diseases (16%)**	Highly related fields	976	751	0.001	2.81	2.78	0.181
**Acoustics (16%)**	Other fields	566	436	0.014	2.74	2.78	0.460
**Orthopedics (15%)**	Highly related fields	1083	871	0.001	2.95	2.85	0.031
**Environmental Sciences (15%)**	Other fields	1987	1277	<0.001	3.21	3.08	0.082
**Sociology (13%)**	Other fields	489	438	0.499	3.14	3.09	0.833
**Complementary & Alternative Medicine (12%)**	Highly related fields	653	290	0.008	2.79	2.55	0.073

© 2022 Ioannidis JPA et al. *JAMA Network Open.*

**Dentistry (12%)**	Highly related fields	793	490	<0.001	2.77	2.7	0.014
**Distributed Computing (12%)**	Other fields	605	454	0.080	2.37	2.41	0.773
**Pathology (11%)**	Highly related fields	1579	1032	0.020	2.89	2.83	0.208
**Evolutionary Biology (11%)**	Other fields	1194	931	0.014	3.26	3.15	0.109
**Artificial Intelligence & Image Processing (11%)**	Other fields	987	895	0.134	2.85	2.86	0.677
**Veterinary Sciences (11%)**	Highly related fields	534	398	<0.001	2.63	2.55	0.012
**Plant Biology & Botany (10%)**	Other fields	1190	895	<0.001	3.01	2.96	0.085
**Chemical Physics (10%)**	Other fields	1648	1200	0.003	3.16	3.1	0.068
**Industrial Engineering & Automation (10%)**	Other fields	810	721	0.344	2.93	2.87	0.993
**Sport Sciences (10%)**	Highly related fields	1264	941	0.109	3.02	3.01	0.663
**Drama & Theater (10%)**	Other fields	73	48	0.384	2.2	2.08	0.223
**Social Work (9.6%)**	Other fields	527	275	0.004	2.97	2.72	0.014
**Criminology (9.6%)**	Other fields	910	514	0.001	3.14	3.07	0.107
**Design Practice & Management (9%)**	Other fields	622	508	0.442	2.81	2.78	0.878
**Polymers (8.5%)**	Other fields	1668	852	<0.001	3.03	2.89	0.211
**General Clinical Medicine (7.8%)**	Highly related fields	577	295	0.059	2.57	2.36	0.423
**Geography (7.5%)**	Other fields	465	401	0.206	3.29	3.05	0.405
**Social Psychology (7.5%)**	Highly related fields	1276	936	0.090	3.48	3.26	0.061
**Development Studies (7.4%)**	Other fields	553	605	0.711	3.02	3.18	0.267
**Economics (7.2%)**	Other fields	680	610	0.008	3.26	3.14	0.059
**Fluids & Plasmas (7.1%)**	Other fields	1286	796	0.003	3.3	3.07	0.040
**Social Sciences Methods (7%)**	Other fields	2253	740	0.013	3.63	3.16	0.006
**Entomology (6.9%)**	Other fields	1083	524	<0.001	3.22	2.82	<0.001
**Numerical & Computational Mathematics (6.9%)**	Other fields	1112	512	0.033	2.92	3	0.957
**Electrical & Electronic Engineering (6.9%)**	Other fields	489	452	0.277	2.5	2.54	0.706
**Gender Studies (6.7%)**	Highly related fields	326	362	0.247	3.14	2.99	0.355

© 2022 Ioannidis JPA et al. *JAMA Network Open.*

**Computer Hardware & Architecture (6.6%)**	Other fields	772	496	0.040	2.62	2.52	0.218
**Logistics & Transportation (6.6%)**	Other fields	635	596	0.994	2.93	2.96	0.334
**Food Science (6.5%)**	Other fields	1381	845	0.066	3.05	2.97	0.422
**Information & Library Sciences (6.3%)**	Other fields	188	181	0.467	2.79	2.55	0.058
**Psychoanalysis (6.1%)**	Highly related fields	334	121	0.070	2.77	2.38	0.258
**Education (6%)**	Other fields	724	413	<0.001	2.96	2.94	0.294
**Archaeology (5.9%)**	Other fields	382	354	0.513	2.76	2.79	0.698
**Optoelectronics & Photonics (5.9%)**	Other fields	501	369	0.020	2.48	2.41	0.286
**History of Social Sciences (5.9%)**	Other fields	184	215	0.838	2.58	2.77	0.414
**Science Studies (5.7%)**	Other fields	415	552	0.477	2.99	3.16	0.155
**Human Factors (5.6%)**	Highly related fields	991	748	0.284	3.06	3.01	0.457
**Urban & Regional Planning (5.6%)**	Other fields	799	426	0.307	3.43	3.16	0.178
**Networking & Telecommunications (5.2%)**	Other fields	537	564	0.840	2.6	2.63	0.237
**Dairy & Animal Science (5.1%)**	Other fields	701	492	0.026	2.57	2.64	0.938
**Languages & Linguistics (5.1%)**	Other fields	365	362	0.936	3.2	3.06	0.987
**Building & Construction (4.9%)**	Other fields	1504	843	0.036	3.08	3.05	0.846
**Religions & Theology (4.9%)**	Other fields	98.5	70	0.185	2.24	2.28	0.532
**Inorganic & Nuclear Chemistry (4.9%)**	Other fields	922	739	0.550	2.8	2.78	0.904
**Applied Mathematics (4.8%)**	Other fields	1594	930	0.027	3.55	3.13	0.164
**Mechanical Engineering & Transports (4.7%)**	Other fields	538	559	0.812	2.81	2.86	0.246
**General Psychology & Cognitive Sciences (4.7%)**	Highly related fields	501	578	0.886	2.79	2.98	0.260
**Mining & Metallurgy (4.7%)**	Other fields	836	252	0.453	2.88	2.36	0.299
**Software Engineering (4.5%)**	Other fields	719	622	0.744	2.76	2.79	0.589
**Anthropology (4.3%)**	Other fields	592	400	0.146	2.88	3.01	0.426

© 2022 Ioannidis JPA et al. *JAMA Network Open.*

**Materials (4.3%)**	Other fields	1249	716	0.003	2.95	2.82	0.251
**Chemical Engineering (4.3%)**	Other fields	1394	870	0.403	3.02	2.96	0.652
**Accounting (4.1%)**	Other fields	805	700	0.911	2.97	3.11	0.266
**Econometrics (4.1%)**	Other fields	1326	1065	0.649	3.1	3.48	0.157
**Ecology (3.9%)**	Other fields	1006	1226	0.459	3.16	3.16	0.514
**Sport, Leisure & Tourism (3.8%)**	Other fields	584	658	0.761	3.09	3.1	0.866
**Legal & Forensic Medicine (3.8%)**	Highly related fields	172	211	0.608	2.3	2.3	0.963
**Communication & Media Studies (3.7%)**	Other fields	718	446	0.126	3.12	3.08	0.639
**Physical Chemistry (3.7%)**	Other fields	1724	1358	0.490	2.96	2.98	0.743
**Operations Research (3.6%)**	Other fields	680	693	0.686	3.16	3.14	0.618
**Anatomy & Morphology (3.6%)**	Highly related fields	373	184	1.000	2.39	2.4	0.895
**Information Systems (3.6%)**	Other fields	837	900	0.822	3.17	3.06	0.865
**Marketing (3.5%)**	Other fields	724	686	0.859	3.23	3.09	0.683
**Ornithology (3.4%)**	Other fields	401	353	0.811	2.95	2.68	0.189
**General Physics (3.3%)**	Other fields	760	679	0.288	2.86	2.82	0.471
**Finance (3.3%)**	Other fields	698	738	0.449	3.1	3.11	0.623
**Zoology (3.3%)**	Other fields	590	327	0.156	2.67	2.57	0.517
**Oceanography (3%)**	Other fields	1059	653	0.100	3.16	2.92	0.127
**Meteorology & Atmospheric Sciences (3%)**	Other fields	1305	1360	0.448	3.27	3.12	0.080
**Applied Physics (2.8%)**	Other fields	1189	879	0.019	2.81	2.82	0.569
**Geological & Geomatics Engineering (2.8%)**	Other fields	1880	935	0.064	3.46	3.07	0.037
**Aerospace & Aeronautics (2.8%)**	Other fields	306	291	0.840	2.48	2.5	0.533
**Marine Biology & Hydrobiology (2.8%)**	Other fields	919	940	0.576	2.97	3.07	0.296
**Cultural Studies (2.8%)**	Other fields	100	124	0.810	2.52	2.54	0.962
**Energy (2.6%)**	Other fields	1018	788	0.053	2.94	2.87	0.548
**Environmental Engineering (2.5%)**	Other fields	971	787	0.178	2.9	2.97	0.715
**Geology (2.4%)**	Other fields	378	490	0.321	2.83	2.89	0.400
**Business & Management (2.3%)**	Other fields	932	790	0.260	3.31	3.17	0.288

© 2022 Ioannidis JPA et al. *JAMA Network Open.*

**Strategic, Defence & Security Studies (2.3%)**	Other fields	225	408	0.587	2.84	2.86	0.480
**Computation Theory & Mathematics (2.3%)**	Other fields	862	507	0.082	3.25	2.93	0.104
**Law (2.2%)**	Other fields	219	138	0.470	2.55	2.48	0.851
**Political Science & Public Administration (2%)**	Other fields	1694	446	0.044	3.43	3.07	0.059
**History (2%)**	Other fields	48.5	50	0.678	2.08	2.08	0.715
**International Relations (1.8%)**	Other fields	519	333	0.439	2.98	3.01	0.734
**Astronomy & Astrophysics (1.7%)**	Other fields	1280	1769	0.297	3.11	3.14	0.906
**General Mathematics (1.7%)**	Other fields	251	299	0.648	2.95	2.85	0.847
**Fisheries (1.6%)**	Other fields	1170	593	0.169	2.99	2.8	0.263
**Philosophy (1.6%)**	Other fields	736	200	0.023	3.65	2.86	0.020
**Literary Studies (1.4%)**	Other fields	95.5	44	0.285	2.44	1.99	0.165
**Agricultural Economics & Policy (1.4%)**	Other fields	911	732	0.651	3.23	3.08	0.547
**Civil Engineering (1.4%)**	Other fields	1074	563	0.215	2.83	2.84	0.855
**Geochemistry & Geophysics (1.3%)**	Other fields	733	869	0.420	3.14	3.15	0.499
**Forestry (1.1%)**	Other fields	489	492	0.720	2.73	2.65	0.888
**Nuclear & Particle Physics (1.1%)**	Other fields	890	759	0.708	2.9	2.9	0.969
**Agronomy & Agriculture (.83%)**	Other fields	761	772	0.383	3.23	2.89	0.129
**Paleontology (.74%)**	Other fields	496	803	0.279	2.88	3.1	0.143
**Music (0%)**	Other fields	.	52		.	2.06	
**Mathematical Physics (0%)**	Other fields	.	358		.	2.88	
**Industrial Relations (0%)**	Other fields	.	218		.	2.87	
**Horticulture (0%)**	Other fields	.	396		.	2.64	
**History of Science, Technology & Medicine (0%)**	Other fields	.	68		.	2.27	
**Folklore (0%)**	Other fields	.	45		.	2.1	
**Economic Theory (0%)**	Other fields	.	323		.	2.93	
**Classics (0%)**	Other fields	.	41		.	2.04	

© 2022 Ioannidis JPA et al. *JAMA Network Open.*

**Automobile Design & Engineering (0%)**	Other fields	.	122		.	2.18	
**Art Practice, History & Theory (0%)**	Other fields	.	36		.	1.88	
**Architecture (0%)**	Other fields	.	74		.	2.12	

© 2022 Ioannidis JPA et al. *JAMA Network Open.*

eTable 2.6 : Recent year impact, Funding time current funding citation counts and composite citation indices for each subfield (ordered by percentage funded)

Top-cited US-based researchers: Subfield (perc. funded)	Classification	Citations for funded, median	Citations for non-funded, median	p-value	Composite index for funded, median	Composite index for non-funded, median	p-value
**Geriatrics (43%)**	Highly related fields	1755	1287	0.380	3.07	3.08	0.881
**Developmental Biology (36%)**	Highly related fields	1956	1802	0.054	3.13	3.15	0.390
**Medical Informatics (34%)**	Highly related fields	708	637	0.238	2.74	2.69	0.195
**Bioinformatics (34%)**	Highly related fields	2949	1739	0.004	3.06	2.98	0.079
**Substance Abuse (31%)**	Highly related fields	1356	1028	0.093	3.06	3.1	0.280
**Gerontology (31%)**	Highly related fields	1240	1066	0.442	3.07	3.14	0.600
**Biomedical Engineering (30%)**	Highly related fields	988	831	0.016	2.83	2.83	0.682
**Virology (30%)**	Highly related fields	1198	1202	0.335	2.88	2.84	0.224
**Immunology (30%)**	Highly related fields	1925	1841	0.561	3.1	3.09	0.706
**Neurology & Neurosurgery (29%)**	Highly related fields	1577	1479	0.005	3.13	3.11	0.423
**Allergy (27%)**	Highly related fields	1600	1362	0.851	3.15	3.17	0.753
**Emergency & Critical Care Medicine (27%)**	Highly related fields	1381	1013	0.001	2.97	2.84	0.023
**Oncology & Carcinogenesis (26%)**	Highly related fields	2330	1978	<0.001	3.02	2.99	0.625
**Public Health (26%)**	Highly related fields	1298	1132	0.051	3.12	3.11	0.759
**Gastroenterology & Hepatology (24%)**	Highly related fields	1433	1281	0.092	2.99	2.97	0.319
**Psychiatry (23%)**	Highly related fields	1729	1573	0.005	3.16	3.19	0.631
**Arthritis & Rheumatology (23%)**	Highly related fields	2088	1964	0.951	3.07	3.14	0.447
**Epidemiology (22%)**	Highly related fields	1990	2028	0.977	3.06	3.17	0.157
**Genetics & Heredity (22%)**	Highly related fields	1770	1357	0.006	2.84	2.84	0.870

© 2022 Ioannidis JPA et al. *JAMA Network Open.*

**Analytical Chemistry (22%)**	Other fields	973	729	<0.001	2.8	2.79	0.751
**Ophthalmology & Optometry (22%)**	Highly related fields	1123	783	<0.001	2.88	2.79	0.002
**Developmental & Child Psychology (22%)**	Highly related fields	1221	950	0.005	3.18	3.16	0.953
**Nuclear Medicine & Medical Imaging (22%)**	Highly related fields	1108	810	<0.001	2.76	2.7	<0.001
**Endocrinology & Metabolism (21%)**	Highly related fields	1444	1541	0.529	3.13	3.15	0.985
**Biophysics (21%)**	Highly related fields	707	601	0.608	2.78	2.8	0.839
**Toxicology (21%)**	Highly related fields	1129	818	<0.001	2.83	2.8	0.246
**Cardiovascular System & Hematology (21%)**	Highly related fields	1984	1754	0.001	3.06	3.02	0.043
**Respiratory System (21%)**	Highly related fields	1446	1449	0.427	2.92	2.96	0.184
**Physiology (21%)**	Highly related fields	705	663	0.590	2.91	2.96	0.784
**Rehabilitation (21%)**	Highly related fields	815	715	0.137	2.8	2.85	0.209
**Microbiology (20%)**	Highly related fields	1396	1277	0.021	3	2.98	0.066
**Urology & Nephrology (20%)**	Highly related fields	1324	1109	0.070	2.97	2.87	0.003
**Medicinal & Biomolecular Chemistry (20%)**	Highly related fields	806	679	0.023	2.63	2.62	0.686
**Health Policy & Services (20%)**	Highly related fields	1144	1020	0.192	3.01	2.98	0.553
**Pediatrics (19%)**	Highly related fields	845	714	0.004	2.73	2.68	0.078
**Obstetrics & Reproductive Medicine (19%)**	Highly related fields	791	766	0.634	2.8	2.79	0.776
**Anesthesiology (18%)**	Highly related fields	781	644	0.004	2.73	2.7	0.298
**Statistics & Probability (17%)**	Other fields	1491	873	0.001	3.19	3.12	0.360
**Biochemistry & Molecular Biology (17%)**	Highly related fields	1141	901	<0.001	2.99	2.93	0.085
**Speech-Language Pathology & Audiology (17%)**	Highly related fields	607	469	0.574	2.89	2.87	0.799
**Demography (17%)**	Highly related fields	625	445	0.149	3.09	2.96	0.373
**Experimental Psychology (16%)**	Highly related fields	1154	818	<0.001	3.23	3.19	0.060

© 2022 Ioannidis JPA et al. *JAMA Network Open.*

**Pharmacology & Pharmacy (15%)**	Highly related fields	775	608	<0.001	2.74	2.67	0.213
**Clinical Psychology (15%)**	Highly related fields	1232	1126	0.207	3.16	3.15	0.919
**Applied Ethics (15%)**	Highly related fields	988	549	0.021	3.07	2.96	0.252
**Organic Chemistry (15%)**	Other fields	1175	873	0.005	2.92	2.9	0.262
**Nutrition & Dietetics (14%)**	Highly related fields	1378	1018	0.019	3.14	3.06	0.397
**Mycology & Parasitology (14%)**	Highly related fields	734	755	0.996	2.81	2.8	0.660
**Nursing (14%)**	Highly related fields	409	266	<0.001	2.5	2.47	0.119
**Optics (14%)**	Other fields	931	927	0.322	2.73	2.77	0.760
**General Chemistry (13%)**	Other fields	911	730	0.064	2.84	2.69	0.064
**General & Internal Medicine (13%)**	Highly related fields	804	632	<0.001	2.71	2.56	<0.001
**Surgery (13%)**	Highly related fields	1067	770	<0.001	2.75	2.66	0.011
**Nanoscience & Nanotechnology (13%)**	Other fields	2256	2447	0.903	3.15	3.15	0.530
**Otorhinolaryngology (12%)**	Highly related fields	708	499	0.007	2.63	2.61	0.490
**Environmental & Occupational Health (12%)**	Highly related fields	594	538	0.749	2.66	2.62	0.991
**Tropical Medicine (12%)**	Highly related fields	873	736	0.190	2.76	2.69	0.354
**Microscopy (12%)**	Highly related fields	1046	664	0.408	2.9	2.75	0.544
**Biotechnology (12%)**	Highly related fields	966	971	0.885	2.87	2.86	0.932
**Drama & Theater (10%)**	Other fields	73	48	0.384	2.2	2.08	0.223
**Complementary & Alternative Medicine (10%)**	Highly related fields	669	294	0.017	2.8	2.53	0.025
**Acoustics (9.9%)**	Other fields	558	448	0.161	2.74	2.77	0.586
**Dermatology & Venereal Diseases (9.7%)**	Highly related fields	976	768	0.034	2.81	2.79	0.646
**Orthopedics (9.5%)**	Highly related fields	1121	880	0.004	2.97	2.85	0.025
**Family Studies (9.1%)**	Other fields	643	442	0.114	3.05	2.97	0.482
**Environmental Sciences (9%)**	Other fields	2081	1297	0.001	3.25	3.08	0.083
**Behavioral Science & Comparative Psychology (8.7%)**	Highly related fields	1129	671	0.278	3.06	3.08	0.751

© 2022 Ioannidis JPA et al. *JAMA Network Open.*

**Sociology (7.8%)**	Other fields	418	445	0.472	3.03	3.1	0.219
**Pathology (7.7%)**	Highly related fields	1656	1079	0.182	2.89	2.83	0.479
**Design Practice & Management (7.5%)**	Other fields	577	515	0.519	2.94	2.77	0.633
**Dentistry (7.3%)**	Highly related fields	713	499	0.002	2.71	2.7	0.220
**Plant Biology & Botany (7.2%)**	Other fields	1262	909	<0.001	3	2.96	0.251
**Evolutionary Biology (7.1%)**	Other fields	1247	934	0.025	3.3	3.15	0.039
**Artificial Intelligence & Image Processing (6.7%)**	Other fields	908	904	0.630	2.8	2.86	0.410
**Chemical Physics (6.7%)**	Other fields	1648	1222	0.059	3.2	3.1	0.124
**Industrial Engineering & Automation (6.7%)**	Other fields	794	728	0.537	2.89	2.88	0.828
**Criminology (6.4%)**	Other fields	733	523	0.031	3.13	3.07	0.456
**Sport Sciences (6.3%)**	Highly related fields	1591	941	0.018	3.08	3.01	0.395
**Veterinary Sciences (6.2%)**	Highly related fields	615	411	0.011	2.67	2.55	0.065
**Psychoanalysis (6.1%)**	Highly related fields	334	121	0.070	2.77	2.38	0.258
**Social Work (6%)**	Other fields	697	295	0.009	2.98	2.72	0.034
**Polymers (5.9%)**	Other fields	1743	861	0.004	3	2.9	0.902
**General Clinical Medicine (5.8%)**	Highly related fields	697	295	0.017	2.57	2.36	0.499
**Entomology (5.8%)**	Other fields	1093	526	<0.001	3.24	2.82	<0.001
**Religions & Theology (4.9%)**	Other fields	98.5	70	0.185	2.24	2.28	0.532
**Distributed Computing (4.9%)**	Other fields	602	461	0.565	2.43	2.4	0.365
**Computer Hardware & Architecture (4.4%)**	Other fields	707	497	0.197	2.69	2.52	0.213
**Information & Library Sciences (4.2%)**	Other fields	147	181	0.875	2.71	2.57	0.266
**Education (4%)**	Other fields	704	417	0.001	2.99	2.93	0.674
**Logistics & Transportation (4%)**	Other fields	839	595	0.399	2.93	2.96	0.647
**Social Psychology (3.9%)**	Highly related fields	1303	942	0.210	3.49	3.27	0.138
**Applied Mathematics (3.8%)**	Other fields	1331	942	0.112	3.3	3.15	0.499
**Legal & Forensic Medicine (3.8%)**	Highly related fields	172	211	0.608	2.3	2.3	0.963

© 2022 Ioannidis JPA et al. *JAMA Network Open.*

**Physical Chemistry (3.7%)**	Other fields	1724	1358	0.490	2.96	2.98	0.743
**Development Studies (3.7%)**	Other fields	843	601	0.521	3.08	3.16	0.608
**Economics (3.6%)**	Other fields	1404	609	0.001	3.6	3.14	0.006
**Anatomy & Morphology (3.6%)**	Highly related fields	373	184	1.000	2.39	2.4	0.895
**Food Science (3.6%)**	Other fields	1605	853	0.238	2.92	2.99	0.912
**Ornithology (3.4%)**	Other fields	401	353	0.811	2.95	2.68	0.189
**Geography (3.3%)**	Other fields	336	406	0.310	2.95	3.06	0.225
**Electrical & Electronic Engineering (3.3%)**	Other fields	470	460	0.717	2.44	2.54	0.387
**Networking & Telecommunications (3.3%)**	Other fields	559	561	0.742	2.61	2.63	0.436
**Dairy & Animal Science (3.3%)**	Other fields	665	493	0.054	2.49	2.65	0.164
**General Psychology & Cognitive Sciences (3.1%)**	Highly related fields	359	583	0.203	2.74	2.99	0.083
**Fluids & Plasmas (3.1%)**	Other fields	1146	802	0.116	3.28	3.08	0.245
**Social Sciences Methods (3%)**	Other fields	3951	760	0.059	4.25	3.23	0.033
**Numerical & Computational Mathematics (3%)**	Other fields	1112	512	0.036	3.57	2.98	0.222
**Building & Construction (2.9%)**	Other fields	1504	879	0.140	3.08	3.05	0.976
**Mechanical Engineering & Transports (2.9%)**	Other fields	759	554	0.803	2.78	2.86	0.298
**Science Studies (2.9%)**	Other fields	583	546	0.621	3.03	3.16	0.488
**Cultural Studies (2.8%)**	Other fields	100	124	0.810	2.52	2.54	0.962
**Marketing (2.8%)**	Other fields	724	686	0.721	3.23	3.09	0.442
**Anthropology (2.6%)**	Other fields	630	411	0.113	2.88	3.01	0.334
**Materials (2.4%)**	Other fields	1308	721	0.004	2.98	2.82	0.142
**Operations Research (2.4%)**	Other fields	819	693	0.601	3.29	3.14	0.565
**Mining & Metallurgy (2.3%)**	Other fields	202	253	0.573	2.31	2.36	0.809
**Optoelectronics & Photonics (2.3%)**	Other fields	639	373	0.104	2.57	2.41	0.164
**Human Factors (2.3%)**	Highly related fields	1129	739	0.056	3.37	3.01	0.136
**Software Engineering (2.2%)**	Other fields	719	622	0.746	2.65	2.8	0.149
**Finance (2.2%)**	Other fields	698	738	0.550	3.19	3.11	0.556

© 2022 Ioannidis JPA et al. *JAMA Network Open.*

**Meteorology & Atmospheric Sciences (2.2%)**	Other fields	1558	1357	0.404	3.27	3.12	0.128
**Communication & Media Studies (2.1%)**	Other fields	826	450	0.062	3.22	3.07	0.406
**Econometrics (2%)**	Other fields	2443	1044	0.289	3.3	3.47	0.621
**Political Science & Public Administration (2%)**	Other fields	1694	446	0.044	3.43	3.07	0.059
**Languages & Linguistics (2%)**	Other fields	484	360	0.412	3.32	3.05	0.196
**General Physics (2%)**	Other fields	629	688	0.648	2.83	2.83	0.520
**Archaeology (2%)**	Other fields	447	360	0.567	2.91	2.78	0.609
**Sport, Leisure & Tourism (1.9%)**	Other fields	790	653	0.776	3.25	3.09	0.478
**Environmental Engineering (1.9%)**	Other fields	1266	787	0.089	2.9	2.97	0.825
**Energy (1.9%)**	Other fields	1030	785	0.023	2.85	2.87	0.974
**Marine Biology & Hydrobiology (1.7%)**	Other fields	1053	929	0.158	3.12	3.07	0.832
**Ecology (1.6%)**	Other fields	951	1224	0.703	3.18	3.16	0.482
**Chemical Engineering (1.6%)**	Other fields	1314	878	0.802	3.35	2.96	0.173
**Philosophy (1.6%)**	Other fields	736	200	0.023	3.65	2.86	0.020
**Aerospace & Aeronautics (1.6%)**	Other fields	371	290	0.660	2.52	2.5	0.732
**Inorganic & Nuclear Chemistry (1.5%)**	Other fields	920	757	0.769	2.81	2.78	0.784
**Agricultural Economics & Policy (1.4%)**	Other fields	911	732	0.651	3.23	3.08	0.547
**Geological & Geomatics Engineering (1.4%)**	Other fields	1457	963	0.562	3.3	3.07	0.248
**Accounting (1.4%)**	Other fields	494	713	0.537	3.1	3.09	1.000
**Applied Physics (1.2%)**	Other fields	945	886	0.262	2.71	2.82	0.686
**Business & Management (1.2%)**	Other fields	1052	791	0.223	3.15	3.17	0.682
**Information Systems (1.2%)**	Other fields	1793	898	0.269	3.49	3.06	0.110
**Computation Theory & Mathematics (1.1%)**	Other fields	607	517	0.665	3.11	2.94	0.485
**Astronomy & Astrophysics (.94%)**	Other fields	2954	1751	0.397	3.11	3.14	0.820
**Geochemistry & Geophysics (.81%)**	Other fields	755	868	0.694	3.14	3.15	0.653

© 2022 Ioannidis JPA et al. *JAMA Network Open.*

**Strategic, Defence & Security Studies (.77%)**	Other fields	159	408	0.139	2.84	2.86	0.926
**Paleontology (.74%)**	Other fields	496	803	0.279	2.88	3.1	0.143
**Literary Studies (.72%)**	Other fields	143	44	0.163	2.75	2	0.129
**General Mathematics (.71%)**	Other fields	267	298	0.703	2.95	2.85	0.295
**Nuclear & Particle Physics (.63%)**	Other fields	3204	759	0.085	3.2	2.9	0.062
**Oceanography (.6%)**	Other fields	1077	654	0.307	2.71	2.93	0.184
**Agronomy & Agriculture (.55%)**	Other fields	1898	771	0.268	3.34	2.89	0.078
**Fisheries (.53%)**	Other fields	1170	593	0.201	2.99	2.8	0.481
**Zoology (0%)**	Other fields	.	337		.	2.58	
**Urban & Regional Planning (0%)**	Other fields	.	428		.	3.16	
**Music (0%)**	Other fields	.	52		.	2.06	
**Mathematical Physics (0%)**	Other fields	.	358		.	2.88	
**Law (0%)**	Other fields	.	138		.	2.48	
**International Relations (0%)**	Other fields	.	343		.	3.01	
**Industrial Relations (0%)**	Other fields	.	218		.	2.87	
**Horticulture (0%)**	Other fields	.	396		.	2.64	
**History of Social Sciences (0%)**	Other fields	.	197		.	2.75	
**History of Science, Technology & Medicine (0%)**	Other fields	.	68		.	2.27	
**History (0%)**	Other fields	.	50		.	2.08	
**Geology (0%)**	Other fields	.	490		.	2.89	
**Gender Studies (0%)**	Highly related fields	.	360		.	3.02	
**Forestry (0%)**	Other fields	.	492		.	2.65	
**Folklore (0%)**	Other fields	.	45		.	2.1	
**Economic Theory (0%)**	Other fields	.	323		.	2.93	
**Classics (0%)**	Other fields	.	41		.	2.04	
**Civil Engineering (0%)**	Other fields	.	564		.	2.84	
**Automobile Design & Engineering (0%)**	Other fields	.	122		.	2.18	
**Art Practice, History & Theory (0%)**	Other fields	.	36		.	1.88	
**Architecture (0%)**	Other fields	.	74		.	2.12	

© 2022 Ioannidis JPA et al. *JAMA Network Open.*

eTable 3. Linear Regressions

eTable 3.1 : Career-long impact, Funding time any funding Linear Regressions for each subfield (ordered by percentage funded)

Top-cited US-based researchers: Subfield (perc. funded)	Classification	Dependent Variable	Constant (p-val)	Funded (p-val)	Years since first pub (p-val)
**Developmental Biology (89%)**	**Highly related fields**	Raw citations	16567 (<0.001)	4342 (0.026)	17.1 (0.773)
		Composite	3.53 (<0.001)	.0816 (0.001)	.00752 (<0.001)
**Substance Abuse (87%)**	**Highly related fields**	Raw citations	2560 (0.207)	2396 (0.055)	134 (0.001)
		Composite	3.52 (<0.001)	.0959 (0.035)	.00466 (0.001)
**Immunology (85%)**	**Highly related fields**	Raw citations	14839 (<0.001)	3606 (0.004)	32.7 (0.412)
		Composite	3.6 (<0.001)	.129 (<0.001)	.00406 (<0.001)
**Geriatrics (85%)**	**Highly related fields**	Raw citations	5149 (0.361)	6804 (0.052)	50.5 (0.711)
		Composite	3.33 (<0.001)	.125 (0.179)	.0075 (0.041)
**Biochemistry & Molecular Biology (81%)**	**Highly related fields**	Raw citations	9394 (<0.001)	3582 (<0.001)	25.5 (0.287)
		Composite	3.57 (<0.001)	.118 (<0.001)	.00382 (<0.001)
**Endocrinology & Metabolism (81%)**	**Highly related fields**	Raw citations	10291 (<0.001)	4340 (<0.001)	50 (0.226)
		Composite	3.6 (<0.001)	.11 (<0.001)	.00405 (<0.001)
**Gerontology (80%)**	**Highly related fields**	Raw citations	6911 (0.466)	8101 (0.087)	-39.8 (0.837)
		Composite	3.34 (<0.001)	.205 (0.003)	.00533 (0.060)
**Virology (80%)**	**Highly related fields**	Raw citations	6774 (<0.001)	3764 (<0.001)	78.1 (0.027)
		Composite	3.39 (<0.001)	.1 (<0.001)	.00534 (<0.001)
**Neurology & Neurosurgery (79%)**	**Highly related fields**	Raw citations	9504 (<0.001)	5083 (<0.001)	46.2 (0.043)
		Composite	3.55 (<0.001)	.131 (<0.001)	.00482 (<0.001)
**Genetics & Heredity (79%)**	**Highly related fields**	Raw citations	17597 (<0.001)	4701 (0.019)	-97.5 (0.216)
		Composite	3.51 (<0.001)	.0698 (0.047)	.00414 (0.003)

© 2022 Ioannidis JPA et al. *JAMA Network Open.*

**Bioinformatics (77%)**	**Highly related fields**	Raw citations	15469 (0.024)	6025 (0.149)	-27.7 (0.871)
		Composite	3.37 (<0.001)	.127 (0.016)	.00574 (0.008)
**Psychiatry (76%)**	**Highly related fields**	Raw citations	4549 (0.067)	7887 (<0.001)	149 (0.003)
		Composite	3.56 (<0.001)	.15 (<0.001)	.00527 (<0.001)
**Oncology & Carcinogenesis (74%)**	**Highly related fields**	Raw citations	14805 (<0.001)	4412 (<0.001)	18.4 (0.558)
		Composite	3.41 (<0.001)	.119 (<0.001)	.00628 (<0.001)
**Public Health (73%)**	**Highly related fields**	Raw citations	4933 (0.004)	872 (0.355)	142 (<0.001)
		Composite	3.45 (<0.001)	.0795 (0.001)	.00587 (<0.001)
**Medical Informatics (71%)**	**Highly related fields**	Raw citations	3995 (0.098)	2189 (0.151)	5.3 (0.926)
		Composite	3.18 (<0.001)	.142 (0.025)	.00323 (0.170)
**Demography (71%)**	**Highly related fields**	Raw citations	-107 (0.968)	467 (0.750)	103 (0.061)
		Composite	3.32 (<0.001)	.0289 (0.739)	.00681 (0.037)
**Physiology (71%)**	**Highly related fields**	Raw citations	2908 (0.028)	3168 (<0.001)	58.6 (0.015)
		Composite	3.63 (<0.001)	.0918 (0.007)	.00195 (0.095)
**Epidemiology (71%)**	**Highly related fields**	Raw citations	-4950 (0.668)	11292 (0.058)	483 (0.055)
		Composite	3.5 (<0.001)	.0814 (0.173)	.00859 (0.001)
**Biomedical Engineering (71%)**	**Highly related fields**	Raw citations	1657 (0.373)	5018 (<0.001)	84.3 (0.034)
		Composite	3.07 (<0.001)	.167 (<0.001)	.00764 (<0.001)
**Developmental & Child Psychology (70%)**	**Highly related fields**	Raw citations	1724 (0.326)	5275 (<0.001)	119 (0.002)
		Composite	3.48 (<0.001)	.164 (<0.001)	.00591 (<0.001)
**Arthritis & Rheumatology (70%)**	**Highly related fields**	Raw citations	5392 (0.189)	5538 (0.006)	199 (0.021)
		Composite	3.49 (<0.001)	.139 (0.003)	.00647 (0.001)
**Allergy (69%)**	**Highly related fields**	Raw citations	4591 (0.205)	5965 (0.001)	86.9 (0.238)
		Composite	3.48 (<0.001)	.162 (0.004)	.00481 (0.032)
**Biophysics (69%)**	**Highly related fields**	Raw citations	654 (0.840)	6523 (<0.001)	91.3 (0.161)
		Composite	3.1 (<0.001)	.297 (<0.001)	.007 (<0.001)

© 2022 Ioannidis JPA et al. *JAMA Network Open.*

**Respiratory System (66%)**	**Highly related fields**	Raw citations	8992 (<0.001)	2276 (0.022)	89.3 (0.047)
		Composite	3.53 (<0.001)	.0332 (0.180)	.00476 (<0.001)
**Experimental Psychology (65%)**	**Highly related fields**	Raw citations	4684 (<0.001)	3123 (<0.001)	53.5 (0.023)
		Composite	3.62 (<0.001)	.0967 (<0.001)	.00366 (<0.001)
**Gastroenterology & Hepatology (65%)**	**Highly related fields**	Raw citations	9502 (<0.001)	3471 (<0.001)	42.2 (0.300)
		Composite	3.55 (<0.001)	.107 (<0.001)	.00381 (<0.001)
**Cardiovascular System & Hematology (64%)**	**Highly related fields**	Raw citations	18376 (<0.001)	3143 (0.002)	6.02 (0.886)
		Composite	3.55 (<0.001)	.102 (<0.001)	.00485 (<0.001)
**Health Policy & Services (63%)**	**Highly related fields**	Raw citations	-284 (0.914)	3052 (0.026)	222 (0.001)
		Composite	3.23 (<0.001)	.117 (0.003)	.0104 (<0.001)
**Urology & Nephrology (63%)**	**Highly related fields**	Raw citations	10978 (<0.001)	4202 (<0.001)	-29 (0.431)
		Composite	3.49 (<0.001)	.138 (<0.001)	.00345 (<0.001)
**Speech-Language Pathology & Audiology (61%)**	**Highly related fields**	Raw citations	6842 (<0.001)	-278 (0.724)	-49.7 (0.243)
		Composite	3.38 (<0.001)	-.0379 (0.429)	.00447 (0.085)
**Clinical Psychology (60%)**	**Highly related fields**	Raw citations	1024 (0.705)	6703 (<0.001)	154 (0.004)
		Composite	3.57 (<0.001)	.167 (<0.001)	.00476 (0.006)
**Pediatrics (60%)**	**Highly related fields**	Raw citations	4647 (<0.001)	2414 (<0.001)	27 (0.140)
		Composite	3.21 (<0.001)	.13 (<0.001)	.00486 (<0.001)
**Microbiology (60%)**	**Highly related fields**	Raw citations	11977 (<0.001)	2368 (<0.001)	-39.4 (0.119)
		Composite	3.5 (<0.001)	.0787 (<0.001)	.00453 (<0.001)
**Family Studies (59%)**	**Other fields**	Raw citations	-751 (0.596)	1672 (0.027)	98.9 (0.004)
		Composite	3.11 (<0.001)	.111 (0.052)	.0101 (<0.001)
**Applied Ethics (58%)**	**Highly related fields**	Raw citations	5693 (<0.001)	1671 (0.042)	-44.2 (0.245)
		Composite	3.47 (<0.001)	.0812 (0.170)	.00286 (0.298)
**Nutrition & Dietetics (57%)**	**Highly related fields**	Raw citations	6617 (0.002)	2723 (0.013)	48.5 (0.282)
		Composite	3.42 (<0.001)	.137 (<0.001)	.00607 (<0.001)

© 2022 Ioannidis JPA et al. *JAMA Network Open.*

**Ophthalmology & Optometry (57%)**	**Highly related fields**	Raw citations	8300 (<0.001)	3543 (<0.001)	-41.7 (0.098)
		Composite	3.43 (<0.001)	.122 (<0.001)	.00222 (0.012)
**Organic Chemistry (57%)**	**Other fields**	Raw citations	7114 (<0.001)	3134 (<0.001)	23.3 (0.409)
		Composite	3.41 (<0.001)	.165 (<0.001)	.00458 (<0.001)
**Environmental & Occupational Health (57%)**	**Highly related fields**	Raw citations	3908 (0.081)	750 (0.389)	21.3 (0.674)
		Composite	3.1 (<0.001)	.122 (0.004)	.00503 (0.039)
**Toxicology (56%)**	**Highly related fields**	Raw citations	8041 (<0.001)	2954 (<0.001)	-49 (0.110)
		Composite	3.32 (<0.001)	.115 (<0.001)	.00365 (0.003)
**Nursing (55%)**	**Highly related fields**	Raw citations	770 (0.045)	1412 (<0.001)	29.4 (0.001)
		Composite	2.97 (<0.001)	.0966 (<0.001)	.00355 (<0.001)
**Obstetrics & Reproductive Medicine (54%)**	**Highly related fields**	Raw citations	7477 (<0.001)	3282 (<0.001)	-28.4 (0.199)
		Composite	3.41 (<0.001)	.141 (<0.001)	.0026 (0.004)
**Emergency & Critical Care Medicine (53%)**	**Highly related fields**	Raw citations	2569 (0.322)	6546 (<0.001)	124 (0.051)
		Composite	3.22 (<0.001)	.149 (<0.001)	.00724 (<0.001)
**Rehabilitation (53%)**	**Highly related fields**	Raw citations	3170 (0.012)	1469 (0.009)	39.9 (0.173)
		Composite	3.22 (<0.001)	.0989 (0.004)	.00559 (0.002)
**Pharmacology & Pharmacy (51%)**	**Highly related fields**	Raw citations	3751 (<0.001)	3041 (<0.001)	30 (0.097)
		Composite	3.24 (<0.001)	.148 (<0.001)	.00362 (<0.001)
**Behavioral Science & Comparative Psychology (50%)**	**Highly related fields**	Raw citations	3640 (0.004)	2043 (0.001)	29.3 (0.276)
		Composite	3.55 (<0.001)	.0796 (0.021)	.0042 (0.008)
**Analytical Chemistry (49%)**	**Other fields**	Raw citations	3742 (0.001)	4361 (<0.001)	30.1 (0.213)
		Composite	3.18 (<0.001)	.169 (<0.001)	.00555 (<0.001)
**Nuclear Medicine & Medical Imaging (47%)**	**Highly related fields**	Raw citations	5043 (<0.001)	4177 (<0.001)	37.7 (0.078)
		Composite	3.32 (<0.001)	.163 (<0.001)	.00282 (<0.001)
**Otorhinolaryngology (44%)**	**Highly related fields**	Raw citations	5626 (<0.001)	759 (0.022)	-31.3 (0.023)
		Composite	3.28 (<0.001)	.0652 (0.002)	.00245 (0.005)

© 2022 Ioannidis JPA et al. *JAMA Network Open.*

**Dentistry (44%)**	**Highly related fields**	Raw citations	4145 (<0.001)	2233 (<0.001)	-4.44 (0.786)
		Composite	3.3 (<0.001)	.101 (<0.001)	.00277 (0.006)
**Tropical Medicine (44%)**	**Highly related fields**	Raw citations	6474 (<0.001)	3029 (0.001)	-22.9 (0.478)
		Composite	3.31 (<0.001)	.128 (0.001)	.00207 (0.153)
**Mycology & Parasitology (43%)**	**Highly related fields**	Raw citations	8260 (<0.001)	-105 (0.922)	-41.5 (0.324)
		Composite	3.4 (<0.001)	.00709 (0.876)	.00229 (0.201)
**Statistics & Probability (43%)**	**Other fields**	Raw citations	9377 (0.004)	4827 (0.009)	13.3 (0.840)
		Composite	3.65 (<0.001)	.122 (0.002)	.00221 (0.114)
**Medicinal & Biomolecular Chemistry (43%)**	**Highly related fields**	Raw citations	814 (0.356)	2946 (<0.001)	108 (<0.001)
		Composite	2.87 (<0.001)	.186 (<0.001)	.00879 (<0.001)
**Anesthesiology (43%)**	**Highly related fields**	Raw citations	4603 (<0.001)	3137 (<0.001)	20.7 (0.425)
		Composite	3.32 (<0.001)	.148 (<0.001)	.00319 (0.017)
**Dermatology & Venereal Diseases (42%)**	**Highly related fields**	Raw citations	7447 (<0.001)	4780 (<0.001)	-19.5 (0.513)
		Composite	3.56 (<0.001)	.132 (<0.001)	.000994 (0.383)
**Biotechnology (41%)**	**Highly related fields**	Raw citations	2604 (0.117)	3341 (0.001)	104 (0.016)
		Composite	3.06 (<0.001)	.151 (<0.001)	.00882 (<0.001)
**Social Psychology (41%)**	**Highly related fields**	Raw citations	4642 (0.005)	5428 (<0.001)	97.2 (0.004)
		Composite	3.63 (<0.001)	.158 (<0.001)	.00462 (<0.001)
**Surgery (40%)**	**Highly related fields**	Raw citations	6990 (<0.001)	4518 (<0.001)	-1.68 (0.920)
		Composite	3.37 (<0.001)	.129 (<0.001)	.00171 (0.006)
**General & Internal Medicine (40%)**	**Highly related fields**	Raw citations	10778 (<0.001)	4012 (<0.001)	-87.6 (<0.001)
		Composite	3.16 (<0.001)	.224 (<0.001)	.00147 (0.010)
**Complementary & Alternative Medicine (40%)**	**Highly related fields**	Raw citations	1654 (0.048)	2040 (<0.001)	-3.94 (0.869)
		Composite	2.96 (<0.001)	.163 (0.037)	-.000779 (0.833)
**Sport Sciences (37%)**	**Highly related fields**	Raw citations	2289 (0.288)	3834 (0.003)	111 (0.046)
		Composite	3.41 (<0.001)	.0891 (0.041)	.00419 (0.026)

© 2022 Ioannidis JPA et al. *JAMA Network Open.*

**Acoustics (36%)**	**Other fields**	Raw citations	5495 (<0.001)	885 (0.035)	-35.8 (0.013)
		Composite	3.51 (<0.001)	.0277 (0.371)	-.000713 (0.499)
**History of Social Sciences (35%)**	**Other fields**	Raw citations	194 (0.941)	-1365 (0.290)	67.3 (0.232)
		Composite	2.87 (<0.001)	.0155 (0.901)	.012 (0.035)
**Nanoscience & Nanotechnology (35%)**	**Other fields**	Raw citations	6113 (0.039)	4857 (0.031)	493 (<0.001)
		Composite	3.28 (<0.001)	.0836 (0.021)	.0142 (<0.001)
**General Chemistry (34%)**	**Other fields**	Raw citations	9904 (<0.001)	7159 (<0.001)	-43.9 (0.222)
		Composite	3.18 (<0.001)	.358 (<0.001)	.00393 (<0.001)
**Microscopy (33%)**	**Highly related fields**	Raw citations	5132 (0.173)	3678 (0.057)	21 (0.803)
		Composite	3.26 (<0.001)	.149 (0.220)	.00483 (0.376)
**Criminology (31%)**	**Other fields**	Raw citations	2645 (0.017)	1543 (0.022)	51.3 (0.068)
		Composite	3.49 (<0.001)	.0521 (0.206)	.00419 (0.016)
**Sociology (31%)**	**Other fields**	Raw citations	2403 (0.014)	778 (0.228)	60.8 (0.007)
		Composite	3.59 (<0.001)	-.0261 (0.487)	.00395 (0.003)
**Optics (30%)**	**Other fields**	Raw citations	7771 (<0.001)	2447 (0.016)	.845 (0.983)
		Composite	3.23 (<0.001)	.0743 (0.043)	.00524 (<0.001)
**Pathology (30%)**	**Highly related fields**	Raw citations	14703 (<0.001)	910 (0.494)	-68.1 (0.196)
		Composite	3.57 (<0.001)	.0283 (0.477)	.00272 (0.084)
**Social Work (30%)**	**Other fields**	Raw citations	907 (0.155)	856 (0.018)	27.3 (0.124)
		Composite	3.2 (<0.001)	.0656 (0.161)	.00199 (0.389)
**Evolutionary Biology (29%)**	**Other fields**	Raw citations	3332 (0.154)	6642 (<0.001)	136 (0.018)
		Composite	3.59 (<0.001)	.116 (<0.001)	.0064 (<0.001)
**Orthopedics (28%)**	**Highly related fields**	Raw citations	6404 (<0.001)	4295 (<0.001)	2.44 (0.896)
		Composite	3.34 (<0.001)	.193 (<0.001)	.00449 (<0.001)
**Social Sciences Methods (27%)**	**Other fields**	Raw citations	1935 (0.652)	13613 (<0.001)	89.3 (0.285)
		Composite	3.53 (<0.001)	.317 (<0.001)	.00454 (0.015)

© 2022 Ioannidis JPA et al. *JAMA Network Open.*

**Chemical Physics (27%)**	**Other fields**	Raw citations	9130 (<0.001)	7215 (<0.001)	63.1 (0.069)
		Composite	3.73 (<0.001)	.123 (<0.001)	.00235 (0.001)
**General Clinical Medicine (24%)**	**Highly related fields**	Raw citations	3799 (0.019)	4497 (<0.001)	-11.6 (0.734)
		Composite	2.91 (<0.001)	.334 (<0.001)	.00431 (0.060)
**Plant Biology & Botany (24%)**	**Other fields**	Raw citations	7253 (<0.001)	4304 (<0.001)	-8.13 (0.695)
		Composite	3.41 (<0.001)	.181 (<0.001)	.00327 (<0.001)
**Inorganic & Nuclear Chemistry (24%)**	**Other fields**	Raw citations	4970 (0.013)	2307 (0.071)	72.1 (0.070)
		Composite	3.33 (<0.001)	.151 (<0.001)	.00539 (<0.001)
**Veterinary Sciences (23%)**	**Highly related fields**	Raw citations	3034 (<0.001)	1192 (<0.001)	17.2 (0.175)
		Composite	3.23 (<0.001)	.0519 (0.014)	.00282 (0.002)
**Environmental Sciences (21%)**	**Other fields**	Raw citations	5054 (<0.001)	3869 (<0.001)	26.2 (0.423)
		Composite	3.25 (<0.001)	.166 (<0.001)	.00519 (<0.001)
**Human Factors (19%)**	**Highly related fields**	Raw citations	4463 (<0.001)	226 (0.807)	61.7 (0.080)
		Composite	3.41 (<0.001)	.00886 (0.871)	.00705 (0.001)
**Ornithology (18%)**	**Other fields**	Raw citations	3738 (0.001)	-922 (0.243)	-1.63 (0.941)
		Composite	3.27 (<0.001)	.00966 (0.902)	.00468 (0.041)
**Urban & Regional Planning (18%)**	**Other fields**	Raw citations	3631 (0.007)	758 (0.438)	-3.28 (0.920)
		Composite	3.56 (<0.001)	.101 (0.278)	.000996 (0.747)
**Economics (18%)**	**Other fields**	Raw citations	6258 (<0.001)	898 (0.283)	13.1 (0.591)
		Composite	3.61 (<0.001)	.0616 (0.080)	.00437 (<0.001)
**Artificial Intelligence & Image Processing (17%)**	**Other fields**	Raw citations	5478 (<0.001)	2503 (0.001)	59.9 (0.019)
		Composite	3.18 (<0.001)	.0877 (0.001)	.0081 (<0.001)
**Polymers (17%)**	**Other fields**	Raw citations	8319 (<0.001)	3998 (0.002)	-5.84 (0.855)
		Composite	3.49 (<0.001)	.0735 (0.080)	.0019 (0.073)
**Economic Theory (17%)**	**Other fields**	Raw citations	2779 (0.139)	-472 (0.692)	21.1 (0.588)
		Composite	3.52 (<0.001)	-.0314 (0.705)	.00233 (0.392)

© 2022 Ioannidis JPA et al. *JAMA Network Open.*

**Development Studies (17%)**	**Other fields**	Raw citations	5171 (0.023)	-32.7 (0.977)	-36.8 (0.498)
		Composite	3.9 (<0.001)	-.114 (0.390)	-.00629 (0.323)
**Anatomy & Morphology (17%)**	**Highly related fields**	Raw citations	2427 (0.015)	871 (0.373)	-7.35 (0.737)
		Composite	2.97 (<0.001)	-.0567 (0.556)	.00418 (0.057)
**Distributed Computing (17%)**	**Other fields**	Raw citations	2065 (0.205)	5045 (<0.001)	61.3 (0.206)
		Composite	3.13 (<0.001)	.148 (0.019)	.00228 (0.413)
**Entomology (16%)**	**Other fields**	Raw citations	4528 (<0.001)	2884 (<0.001)	-13.2 (0.357)
		Composite	3.49 (<0.001)	.126 (0.001)	.000296 (0.791)
**Gender Studies (15%)**	**Highly related fields**	Raw citations	3224 (0.175)	149 (0.898)	-10.6 (0.851)
		Composite	3.35 (<0.001)	.0864 (0.506)	.00425 (0.503)
**History of Science, Technology & Medicine (15%)**	**Other fields**	Raw citations	350 (0.673)	-115 (0.852)	15.6 (0.325)
		Composite	3 (<0.001)	-.00475 (0.974)	.00379 (0.310)
**Software Engineering (15%)**	**Other fields**	Raw citations	7404 (<0.001)	907 (0.164)	-62.1 (0.012)
		Composite	3.51 (<0.001)	.00865 (0.853)	.000547 (0.757)
**Education (15%)**	**Other fields**	Raw citations	1643 (<0.001)	1753 (<0.001)	42 (<0.001)
		Composite	3.24 (<0.001)	.0881 (0.001)	.00489 (<0.001)
**Food Science (15%)**	**Other fields**	Raw citations	4993 (<0.001)	2336 (0.041)	11.4 (0.708)
		Composite	3.31 (<0.001)	.166 (0.002)	.0033 (0.020)
**Industrial Engineering & Automation (14%)**	**Other fields**	Raw citations	5629 (<0.001)	1686 (0.013)	6.56 (0.752)
		Composite	3.38 (<0.001)	.0786 (0.046)	.00349 (0.004)
**Logistics & Transportation (14%)**	**Other fields**	Raw citations	3226 (<0.001)	-771 (0.226)	14.3 (0.469)
		Composite	3.23 (<0.001)	-.117 (0.065)	.00419 (0.033)
**Geography (14%)**	**Other fields**	Raw citations	1986 (0.073)	2635 (0.004)	52.1 (0.062)
		Composite	3.51 (<0.001)	.228 (0.001)	.00512 (0.014)
**Sport, Leisure & Tourism (13%)**	**Other fields**	Raw citations	3113 (0.010)	-1394 (0.197)	52.1 (0.184)
		Composite	3.44 (<0.001)	-.0131 (0.882)	.00616 (0.058)

© 2022 Ioannidis JPA et al. *JAMA Network Open.*

**Numerical & Computational Mathematics (13%)**	**Other fields**	Raw citations	1444 (0.357)	2179 (0.066)	79 (0.025)
		Composite	3.58 (<0.001)	-.0119 (0.864)	.00121 (0.562)
**Communication & Media Studies (12%)**	**Other fields**	Raw citations	1049 (0.258)	-254 (0.807)	81.3 (0.002)
		Composite	3.45 (<0.001)	.0128 (0.821)	.00336 (0.016)
**General Psychology & Cognitive Sciences (12%)**	**Highly related fields**	Raw citations	2790 (0.007)	547 (0.553)	28.1 (0.254)
		Composite	3.36 (<0.001)	.0861 (0.349)	.0043 (0.082)
**Fluids & Plasmas (12%)**	**Other fields**	Raw citations	13332 (<0.001)	954 (0.625)	-79.2 (0.131)
		Composite	3.73 (<0.001)	.0502 (0.271)	.00145 (0.236)
**Information & Library Sciences (12%)**	**Other fields**	Raw citations	422 (0.471)	1575 (0.011)	46.9 (0.005)
		Composite	2.99 (<0.001)	.187 (0.021)	.00712 (0.001)
**Dairy & Animal Science (12%)**	**Other fields**	Raw citations	4387 (<0.001)	1320 (0.004)	-11 (0.357)
		Composite	3.26 (<0.001)	.0617 (0.123)	.00129 (0.223)
**Optoelectronics & Photonics (12%)**	**Other fields**	Raw citations	3186 (<0.001)	1654 (0.001)	27.5 (0.064)
		Composite	2.99 (<0.001)	.0624 (0.068)	.00574 (<0.001)
**Anthropology (11%)**	**Other fields**	Raw citations	2312 (0.012)	299 (0.648)	39 (0.058)
		Composite	3.48 (<0.001)	.0231 (0.691)	.00405 (0.027)
**Religions & Theology (11%)**	**Other fields**	Raw citations	694 (0.094)	1209 (0.017)	1.88 (0.856)
		Composite	2.9 (<0.001)	.135 (0.189)	.000388 (0.855)
**Drama & Theater (11%)**	**Other fields**	Raw citations	-188 (0.513)	379 (0.116)	21.8 (0.051)
		Composite	2.24 (<0.001)	.246 (0.204)	.0169 (0.065)
**Design Practice & Management (11%)**	**Other fields**	Raw citations	3428 (<0.001)	984 (0.267)	8.29 (0.679)
		Composite	3.26 (<0.001)	.0088 (0.920)	.00201 (0.311)
**Environmental Engineering (11%)**	**Other fields**	Raw citations	5204 (<0.001)	2518 (0.001)	.271 (0.989)
		Composite	3.39 (<0.001)	.0792 (0.066)	.00295 (0.015)
**Chemical Engineering (11%)**	**Other fields**	Raw citations	7541 (<0.001)	1228 (0.213)	-40.3 (0.055)
		Composite	3.45 (<0.001)	.0426 (0.454)	.000511 (0.672)

© 2022 Ioannidis JPA et al. *JAMA Network Open.*

**Applied Mathematics (11%)**	**Other fields**	Raw citations	3721 (0.224)	2946 (0.293)	99.7 (0.149)
		Composite	3.51 (<0.001)	.0474 (0.637)	.0051 (0.040)
**Marketing (11%)**	**Other fields**	Raw citations	2260 (0.276)	760 (0.654)	157 (0.008)
		Composite	3.55 (<0.001)	.083 (0.218)	.0048 (0.040)
**Zoology (10%)**	**Other fields**	Raw citations	2160 (0.012)	547 (0.489)	14.3 (0.523)
		Composite	3.17 (<0.001)	.0279 (0.706)	.00178 (0.396)
**Architecture (10%)**	**Other fields**	Raw citations	-1503 (0.446)	-1246 (0.608)	82.4 (0.166)
		Composite	2.63 (<0.001)	-.112 (0.706)	-.00186 (0.784)
**Ecology (9.7%)**	**Other fields**	Raw citations	6330 (<0.001)	1125 (0.370)	116 (0.003)
		Composite	3.55 (<0.001)	.114 (0.001)	.00723 (<0.001)
**Marine Biology & Hydrobiology (9.7%)**	**Other fields**	Raw citations	7932 (<0.001)	882 (0.410)	-4.67 (0.889)
		Composite	3.67 (<0.001)	.0126 (0.776)	.00217 (0.117)
**Networking & Telecommunications (9.5%)**	**Other fields**	Raw citations	5869 (<0.001)	-49 (0.935)	-7.6 (0.548)
		Composite	3.18 (<0.001)	.0276 (0.383)	.00459 (<0.001)
**Computer Hardware & Architecture (9.3%)**	**Other fields**	Raw citations	5123 (<0.001)	308 (0.749)	11.5 (0.674)
		Composite	3.17 (<0.001)	-.0273 (0.685)	.00665 (0.001)
**Electrical & Electronic Engineering (9.2%)**	**Other fields**	Raw citations	3198 (<0.001)	1177 (0.158)	22.7 (0.192)
		Composite	3.02 (<0.001)	.0495 (0.412)	.00391 (0.002)
**Geological & Geomatics Engineering (9%)**	**Other fields**	Raw citations	5987 (<0.001)	1388 (0.166)	5.72 (0.833)
		Composite	3.3 (<0.001)	.12 (0.029)	.00521 (0.001)
**Information Systems (8.6%)**	**Other fields**	Raw citations	5740 (0.003)	632 (0.731)	64.4 (0.244)
		Composite	3.49 (<0.001)	-.0374 (0.585)	.00482 (0.020)
**Law (8.5%)**	**Other fields**	Raw citations	728 (0.455)	-198 (0.804)	33.4 (0.310)
		Composite	3.11 (<0.001)	.0375 (0.633)	.00577 (0.077)
**Mathematical Physics (8.3%)**	**Other fields**	Raw citations	4734 (0.442)	-2710 (0.608)	8.12 (0.939)
		Composite	3.98 (<0.001)	-.388 (0.163)	-.00584 (0.289)

© 2022 Ioannidis JPA et al. *JAMA Network Open.*

**Strategic, Defence & Security Studies (8.2%)**	**Other fields**	Raw citations	1156 (0.209)	1403 (0.181)	53.4 (0.030)
		Composite	3.13 (<0.001)	.189 (0.021)	.00552 (0.004)
**Agricultural Economics & Policy (8.2%)**	**Other fields**	Raw citations	6112 (<0.001)	2081 (0.156)	-26.5 (0.494)
		Composite	3.68 (<0.001)	-.0732 (0.474)	-.00042 (0.877)
**General Physics (8.2%)**	**Other fields**	Raw citations	9805 (<0.001)	2778 (0.071)	-31.9 (0.247)
		Composite	3.51 (<0.001)	.0254 (0.653)	.00247 (0.015)
**Languages & Linguistics (8.2%)**	**Other fields**	Raw citations	1228 (0.026)	1095 (0.050)	30.6 (0.034)
		Composite	3.34 (<0.001)	.165 (0.036)	.00355 (0.079)
**Mining & Metallurgy (8.1%)**	**Other fields**	Raw citations	-646 (0.497)	1779 (0.087)	60 (0.010)
		Composite	2.34 (<0.001)	.151 (0.384)	.014 (0.001)
**Psychoanalysis (8%)**	**Highly related fields**	Raw citations	1159 (0.431)	-16.9 (0.990)	13.1 (0.590)
		Composite	3.44 (<0.001)	-.0642 (0.641)	-.000979 (0.685)
**Political Science & Public Administration (7.9%)**	**Other fields**	Raw citations	3338 (0.001)	2392 (0.043)	34.6 (0.172)
		Composite	3.62 (<0.001)	.0806 (0.174)	.00205 (0.108)
**Building & Construction (7.8%)**	**Other fields**	Raw citations	2249 (0.019)	925 (0.417)	52.5 (0.058)
		Composite	3.12 (<0.001)	.0654 (0.496)	.00747 (0.002)
**Oceanography (7.6%)**	**Other fields**	Raw citations	5640 (<0.001)	1001 (0.391)	2.28 (0.938)
		Composite	3.39 (<0.001)	.0701 (0.260)	.00529 (0.001)
**Literary Studies (7.5%)**	**Other fields**	Raw citations	271 (0.242)	-82.3 (0.768)	10.4 (0.160)
		Composite	2.63 (<0.001)	.0347 (0.669)	.00521 (0.016)
**Cultural Studies (7.4%)**	**Other fields**	Raw citations	1086 (0.052)	-164 (0.778)	2.14 (0.863)
		Composite	3.25 (<0.001)	-.143 (0.228)	-.0012 (0.632)
**Materials (7.3%)**	**Other fields**	Raw citations	6710 (<0.001)	2386 (0.003)	-25.5 (0.059)
		Composite	3.27 (<0.001)	.13 (0.001)	.00237 (0.001)
**Applied Physics (7.2%)**	**Other fields**	Raw citations	10274 (<0.001)	2334 (0.030)	-13.5 (0.460)
		Composite	3.52 (<0.001)	.095 (0.001)	.00198 (<0.001)

© 2022 Ioannidis JPA et al. *JAMA Network Open.*

**Econometrics (7.1%)**	**Other fields**	Raw citations	3666 (0.469)	6447 (0.215)	169 (0.199)
		Composite	3.57 (<0.001)	.0774 (0.692)	.0101 (0.045)
**Mechanical Engineering & Transports (7.1%)**	**Other fields**	Raw citations	4650 (<0.001)	2431 (0.008)	-3.61 (0.839)
		Composite	3.29 (<0.001)	.0471 (0.382)	.00319 (0.003)
**Operations Research (6.8%)**	**Other fields**	Raw citations	974 (0.479)	-1618 (0.231)	123 (<0.001)
		Composite	3.45 (<0.001)	-.0941 (0.205)	.00547 (0.003)
**Finance (6.8%)**	**Other fields**	Raw citations	3630 (0.041)	-1836 (0.343)	87.8 (0.057)
		Composite	3.48 (<0.001)	-.09 (0.340)	.00547 (0.015)
**Business & Management (6.5%)**	**Other fields**	Raw citations	5584 (<0.001)	-398 (0.737)	67.8 (0.011)
		Composite	3.63 (<0.001)	.0236 (0.611)	.00359 (0.001)
**Meteorology & Atmospheric Sciences (6.4%)**	**Other fields**	Raw citations	10819 (<0.001)	-539 (0.652)	17.7 (0.528)
		Composite	3.68 (<0.001)	-.00165 (0.961)	.00293 (<0.001)
**General Mathematics (6.4%)**	**Other fields**	Raw citations	2958 (<0.001)	1782 (0.001)	-.659 (0.936)
		Composite	3.47 (<0.001)	.0336 (0.448)	.000721 (0.271)
**Computation Theory & Mathematics (6.2%)**	**Other fields**	Raw citations	5038 (<0.001)	805 (0.595)	34.3 (0.285)
		Composite	3.53 (<0.001)	.035 (0.675)	.00466 (0.009)
**Fisheries (6.2%)**	**Other fields**	Raw citations	4249 (<0.001)	542 (0.544)	5.02 (0.824)
		Composite	3.42 (<0.001)	.0137 (0.845)	.00128 (0.471)
**Philosophy (6.2%)**	**Other fields**	Raw citations	1544 (<0.001)	1032 (0.007)	3.66 (0.588)
		Composite	3.4 (<0.001)	.141 (0.065)	.000488 (0.718)
**Archaeology (6.1%)**	**Other fields**	Raw citations	2115 (0.003)	-748 (0.429)	15.8 (0.366)
		Composite	3.23 (<0.001)	-.154 (0.102)	.00427 (0.015)
**Paleontology (6%)**	**Other fields**	Raw citations	7104 (<0.001)	-108 (0.957)	4.64 (0.915)
		Composite	3.64 (<0.001)	.0608 (0.365)	.00263 (0.071)
**Energy (5.9%)**	**Other fields**	Raw citations	4517 (<0.001)	1099 (0.079)	7.36 (0.497)
		Composite	3.2 (<0.001)	.031 (0.436)	.00373 (<0.001)

© 2022 Ioannidis JPA et al. *JAMA Network Open.*

**Agronomy & Agriculture (5.9%)**	**Other fields**	Raw citations	5482 (<0.001)	825 (0.405)	-1.73 (0.927)
		Composite	3.36 (<0.001)	.0335 (0.549)	.0027 (0.012)
**Aerospace & Aeronautics (5.8%)**	**Other fields**	Raw citations	2312 (<0.001)	249 (0.513)	8.15 (0.289)
		Composite	3.08 (<0.001)	-.0169 (0.728)	.0029 (0.003)
**Astronomy & Astrophysics (5.6%)**	**Other fields**	Raw citations	18634 (<0.001)	-353 (0.876)	-85.6 (0.064)
		Composite	3.75 (<0.001)	.0537 (0.140)	.00118 (0.113)
**Classics (5.6%)**	**Other fields**	Raw citations	1694 (0.058)	-197 (0.851)	-23.7 (0.301)
		Composite	2.94 (<0.001)	-.143 (0.505)	.00159 (0.729)
**Geology (5.4%)**	**Other fields**	Raw citations	6016 (<0.001)	-128 (0.910)	-40 (0.061)
		Composite	3.64 (<0.001)	.0227 (0.830)	-.00215 (0.276)
**Forestry (5.3%)**	**Other fields**	Raw citations	2705 (<0.001)	389 (0.608)	18.2 (0.333)
		Composite	3.14 (<0.001)	.0618 (0.442)	.00393 (0.049)
**Geochemistry & Geophysics (4.9%)**	**Other fields**	Raw citations	7287 (<0.001)	72.3 (0.931)	15.7 (0.336)
		Composite	3.66 (<0.001)	.00353 (0.929)	.00313 (<0.001)
**Nuclear & Particle Physics (4.7%)**	**Other fields**	Raw citations	18813 (<0.001)	-5775 (0.033)	-102 (0.002)
		Composite	3.64 (<0.001)	-.0187 (0.689)	.0012 (0.037)
**History (4.5%)**	**Other fields**	Raw citations	833 (0.001)	-82.9 (0.826)	-.847 (0.877)
		Composite	2.91 (<0.001)	.00468 (0.961)	.000996 (0.470)
**International Relations (4.4%)**	**Other fields**	Raw citations	2196 (0.017)	340 (0.801)	27.3 (0.234)
		Composite	3.42 (<0.001)	.0887 (0.515)	.00402 (0.084)
**Physical Chemistry (4.4%)**	**Other fields**	Raw citations	11444 (<0.001)	16389 (<0.001)	-51.5 (0.323)
		Composite	3.49 (<0.001)	.277 (0.106)	.00337 (0.137)
**Science Studies (3.8%)**	**Other fields**	Raw citations	4042 (0.329)	270 (0.950)	27 (0.772)
		Composite	3.7 (<0.001)	-.141 (0.648)	.00151 (0.821)
**Civil Engineering (3.6%)**	**Other fields**	Raw citations	1981 (0.011)	-789 (0.511)	34.5 (0.072)
		Composite	3.1 (<0.001)	-.104 (0.330)	.00447 (0.009)

© 2022 Ioannidis JPA et al. *JAMA Network Open.*

**Legal & Forensic Medicine (3%)**	**Highly related fields**	Raw citations	1687 (0.120)	-1261 (0.493)	13.4 (0.628)
		Composite	2.92 (<0.001)	-.228 (0.240)	.00299 (0.307)
**Music (2.4%)**	**Other fields**	Raw citations	658 (0.029)	41.2 (0.939)	-3.65 (0.654)
		Composite	2.76 (<0.001)	.227 (0.150)	.00133 (0.569)
**Accounting (2.4%)**	**Other fields**	Raw citations	5000 (0.014)	4808 (0.124)	24.2 (0.655)
		Composite	3.44 (<0.001)	.286 (0.165)	.00512 (0.156)
**Horticulture (1.3%)**	**Other fields**	Raw citations	1138 (0.267)	-827 (0.650)	38.6 (0.170)
		Composite	2.98 (<0.001)	-.0732 (0.764)	.00666 (0.078)
**Industrial Relations (0%)**	**Other fields**	Raw citations			
		Composite			
**Folklore (0%)**	**Other fields**	Raw citations			
		Composite			
**Automobile Design & Engineering (0%)**	**Other fields**	Raw citations			
		Composite			
**Art Practice, History & Theory (0%)**	**Other fields**	Raw citations			
		Composite			

© 2022 Ioannidis JPA et al. *JAMA Network Open.*

eTable 3.2 : Career-long impact, Funding time recent funding Linear Regressions for each subfield (ordered by percentage funded)

Top-cited US-based researchers: Subfield (perc. funded)	Classification	Dependent Variable	Constant (p-val)	Funded (p-val)	Years since first pub (p-val)
Developmental Biology (42%)	Highly related fields	Raw citations	18380 (<0.001)	2703 (0.037)	40.4 (0.505)
		Composite	3.58 (<0.001)	.0292 (0.069)	.00773 (<0.001)
Bioinformatics (41%)	Highly related fields	Raw citations	13708 (0.037)	8132 (0.030)	66.5 (0.707)
		Composite	3.38 (<0.001)	.119 (0.012)	.00696 (0.002)
Geriatrics (40%)	Highly related fields	Raw citations	5149 (0.386)	4288 (0.119)	161 (0.271)
		Composite	3.26 (<0.001)	.146 (0.043)	.0108 (0.006)
Substance Abuse (36%)	Highly related fields	Raw citations	2670 (0.129)	2710 (0.003)	159 (<0.001)
		Composite	3.59 (<0.001)	.0219 (0.513)	.00461 (0.002)
Biomedical Engineering (34%)	Highly related fields	Raw citations	4129 (0.017)	3388 (0.002)	82.3 (0.046)
		Composite	3.18 (<0.001)	.0682 (0.034)	.00698 (<0.001)
Medical Informatics (34%)	Highly related fields	Raw citations	4045 (0.082)	2430 (0.107)	25.5 (0.666)
		Composite	3.2 (<0.001)	.133 (0.034)	.00425 (0.083)
Immunology (34%)	Highly related fields	Raw citations	16270 (<0.001)	2368 (0.015)	52.6 (0.201)
		Composite	3.66 (<0.001)	.064 (<0.001)	.00456 (<0.001)
Epidemiology (34%)	Highly related fields	Raw citations	2782 (0.801)	3730 (0.521)	458 (0.075)
		Composite	3.58 (<0.001)	-.00789 (0.892)	.00811 (0.002)
Virology (34%)	Highly related fields	Raw citations	8988 (<0.001)	1275 (0.126)	87 (0.017)
		Composite	3.45 (<0.001)	.0338 (0.132)	.00558 (<0.001)
Neurology & Neurosurgery (32%)	Highly related fields	Raw citations	13756 (<0.001)	1062 (0.067)	32.3 (0.174)
		Composite	3.66 (<0.001)	.0268 (0.026)	.00446 (<0.001)
Public Health (31%)	Highly related fields	Raw citations	4510 (0.007)	1653 (0.075)	157 (<0.001)
		Composite	3.46 (<0.001)	.0781 (0.001)	.00649 (<0.001)

© 2022 Ioannidis JPA et al. *JAMA Network Open.*

**Gerontology (30%)**	**Highly related fields**	Raw citations	6012 (0.469)	11949 (0.004)	56.4 (0.771)
		Composite	3.49 (<0.001)	.114 (0.068)	.00496 (0.093)
**Oncology & Carcinogenesis (30%)**	**Highly related fields**	Raw citations	16904 (<0.001)	2264 (0.006)	31.5 (0.344)
		Composite	3.49 (<0.001)	.0333 (0.014)	.00627 (<0.001)
**Genetics & Heredity (29%)**	**Highly related fields**	Raw citations	18062 (<0.001)	4592 (0.016)	-51.7 (0.533)
		Composite	3.52 (<0.001)	.064 (0.055)	.00476 (0.001)
**Demography (26%)**	**Highly related fields**	Raw citations	-2967 (0.289)	3338 (0.042)	156 (0.009)
		Composite	3.25 (<0.001)	.104 (0.295)	.00844 (0.020)
**Health Policy & Services (26%)**	**Highly related fields**	Raw citations	925 (0.720)	2001 (0.193)	228 (0.001)
		Composite	3.26 (<0.001)	.104 (0.019)	.0108 (<0.001)
**Developmental & Child Psychology (25%)**	**Highly related fields**	Raw citations	3284 (0.051)	4979 (<0.001)	141 (<0.001)
		Composite	3.53 (<0.001)	.152 (<0.001)	.00658 (<0.001)
**Applied Ethics (25%)**	**Highly related fields**	Raw citations	4954 (0.002)	2486 (0.008)	-16.5 (0.660)
		Composite	3.45 (<0.001)	.0944 (0.166)	.00399 (0.155)
**Gastroenterology & Hepatology (25%)**	**Highly related fields**	Raw citations	11100 (<0.001)	2220 (0.046)	44.6 (0.286)
		Composite	3.6 (<0.001)	.0773 (0.008)	.00397 (<0.001)
**Endocrinology & Metabolism (25%)**	**Highly related fields**	Raw citations	14061 (<0.001)	625 (0.584)	39.9 (0.355)
		Composite	3.7 (<0.001)	.0014 (0.954)	.00364 (<0.001)
**Biophysics (25%)**	**Highly related fields**	Raw citations	5070 (0.143)	1652 (0.427)	83.4 (0.255)
		Composite	3.3 (<0.001)	.0786 (0.197)	.00669 (0.002)
**Psychiatry (25%)**	**Highly related fields**	Raw citations	10288 (<0.001)	3928 (0.003)	133 (0.010)
		Composite	3.67 (<0.001)	.0758 (0.004)	.00497 (<0.001)
**Arthritis & Rheumatology (24%)**	**Highly related fields**	Raw citations	11215 (0.004)	700 (0.756)	146 (0.105)
		Composite	3.64 (<0.001)	.011 (0.831)	.00503 (0.015)
**Emergency & Critical Care Medicine (24%)**	**Highly related fields**	Raw citations	3871 (0.129)	7100 (<0.001)	138 (0.035)
		Composite	3.27 (<0.001)	.13 (0.002)	.00711 (<0.001)

© 2022 Ioannidis JPA et al. *JAMA Network Open.*

**Microbiology (23%)**	**Highly related fields**	Raw citations	12835 (<0.001)	1461 (0.042)	-34.2 (0.191)
		Composite	3.53 (<0.001)	.0507 (0.008)	.00472 (<0.001)
**Allergy (23%)**	**Highly related fields**	Raw citations	7706 (0.035)	3713 (0.092)	90.3 (0.262)
		Composite	3.6 (<0.001)	.0464 (0.486)	.00412 (0.093)
**Respiratory System (23%)**	**Highly related fields**	Raw citations	10474 (<0.001)	1032 (0.369)	83.8 (0.070)
		Composite	3.55 (<0.001)	.0191 (0.504)	.00472 (<0.001)
**Clinical Psychology (23%)**	**Highly related fields**	Raw citations	6159 (0.010)	4980 (0.003)	100 (0.059)
		Composite	3.69 (<0.001)	.139 (0.009)	.00347 (0.039)
**Cardiovascular System & Hematology (23%)**	**Highly related fields**	Raw citations	17864 (<0.001)	4871 (<0.001)	41.2 (0.342)
		Composite	3.59 (<0.001)	.0717 (<0.001)	.00509 (<0.001)
**Biochemistry & Molecular Biology (22%)**	**Highly related fields**	Raw citations	13024 (<0.001)	757 (0.281)	6.57 (0.787)
		Composite	3.69 (<0.001)	.0155 (0.342)	.00312 (<0.001)
**Physiology (22%)**	**Highly related fields**	Raw citations	5511 (<0.001)	1054 (0.186)	46 (0.065)
		Composite	3.7 (<0.001)	.0309 (0.413)	.00158 (0.180)
**Ophthalmology & Optometry (21%)**	**Highly related fields**	Raw citations	9174 (<0.001)	3663 (<0.001)	-32.9 (0.206)
		Composite	3.46 (<0.001)	.127 (<0.001)	.00253 (0.006)
**Analytical Chemistry (21%)**	**Other fields**	Raw citations	5155 (<0.001)	3633 (<0.001)	28.4 (0.260)
		Composite	3.24 (<0.001)	.14 (<0.001)	.00548 (<0.001)
**Urology & Nephrology (20%)**	**Highly related fields**	Raw citations	14037 (<0.001)	1128 (0.290)	-45.5 (0.237)
		Composite	3.57 (<0.001)	.0734 (0.007)	.00328 (0.001)
**Rehabilitation (20%)**	**Highly related fields**	Raw citations	4311 (<0.001)	915 (0.183)	25.6 (0.377)
		Composite	3.31 (<0.001)	.038 (0.369)	.00441 (0.014)
**Microscopy (20%)**	**Highly related fields**	Raw citations	4673 (0.196)	5392 (0.016)	35.6 (0.662)
		Composite	3.23 (<0.001)	.238 (0.094)	.00552 (0.304)
**Tropical Medicine (20%)**	**Highly related fields**	Raw citations	7901 (<0.001)	1618 (0.144)	-33 (0.320)
		Composite	3.37 (<0.001)	.0669 (0.175)	.00164 (0.269)

© 2022 Ioannidis JPA et al. *JAMA Network Open.*

**Statistics & Probability (20%)**	**Other fields**	Raw citations	10637 (0.001)	5233 (0.024)	8.11 (0.902)
		Composite	3.69 (<0.001)	.11 (0.027)	.00189 (0.181)
**Family Studies (20%)**	**Other fields**	Raw citations	-123 (0.927)	2036 (0.030)	97.8 (0.005)
		Composite	3.15 (<0.001)	.13 (0.065)	.0101 (<0.001)
**Toxicology (20%)**	**Highly related fields**	Raw citations	8420 (<0.001)	3264 (<0.001)	-33.9 (0.285)
		Composite	3.34 (<0.001)	.113 (0.001)	.0041 (0.001)
**Nutrition & Dietetics (19%)**	**Highly related fields**	Raw citations	7523 (<0.001)	2664 (0.058)	52 (0.262)
		Composite	3.5 (<0.001)	.072 (0.088)	.00566 (<0.001)
**Medicinal & Biomolecular Chemistry (19%)**	**Highly related fields**	Raw citations	1298 (0.149)	2568 (<0.001)	115 (<0.001)
		Composite	2.91 (<0.001)	.132 (<0.001)	.00907 (<0.001)
**Nuclear Medicine & Medical Imaging (19%)**	**Highly related fields**	Raw citations	6170 (<0.001)	3041 (<0.001)	44.7 (0.050)
		Composite	3.38 (<0.001)	.106 (<0.001)	.00295 (<0.001)
**Nanoscience & Nanotechnology (19%)**	**Other fields**	Raw citations	6951 (0.019)	3115 (0.258)	504 (<0.001)
		Composite	3.29 (<0.001)	.044 (0.320)	.0144 (<0.001)
**Pediatrics (19%)**	**Highly related fields**	Raw citations	5342 (<0.001)	2847 (<0.001)	32 (0.086)
		Composite	3.27 (<0.001)	.117 (<0.001)	.00485 (<0.001)
**Pharmacology & Pharmacy (18%)**	**Highly related fields**	Raw citations	4335 (<0.001)	3030 (<0.001)	40.1 (0.031)
		Composite	3.28 (<0.001)	.117 (<0.001)	.00393 (<0.001)
**Optics (18%)**	**Other fields**	Raw citations	7761 (<0.001)	3540 (0.004)	4.19 (0.913)
		Composite	3.25 (<0.001)	.0607 (0.166)	.00502 (<0.001)
**Anesthesiology (18%)**	**Highly related fields**	Raw citations	5756 (<0.001)	2786 (<0.001)	12.5 (0.638)
		Composite	3.39 (<0.001)	.0998 (0.010)	.00254 (0.063)
**Obstetrics & Reproductive Medicine (18%)**	**Highly related fields**	Raw citations	9194 (<0.001)	1693 (0.015)	-33.9 (0.143)
		Composite	3.49 (<0.001)	.0569 (0.044)	.00223 (0.018)
**Experimental Psychology (18%)**	**Highly related fields**	Raw citations	6479 (<0.001)	2860 (0.001)	47.3 (0.045)
		Composite	3.68 (<0.001)	.0928 (0.004)	.00349 (<0.001)

© 2022 Ioannidis JPA et al. *JAMA Network Open.*

**Speech-Language Pathology & Audiology (17%)**	**Highly related fields**	Raw citations	6410 (<0.001)	662 (0.521)	-45.9 (0.286)
		Composite	3.36 (<0.001)	.00525 (0.934)	.00439 (0.096)
**Biotechnology (17%)**	**Highly related fields**	Raw citations	3864 (0.021)	1784 (0.168)	98.4 (0.027)
		Composite	3.1 (<0.001)	.119 (0.037)	.00877 (<0.001)
**Organic Chemistry (16%)**	**Other fields**	Raw citations	8186 (<0.001)	4348 (<0.001)	23.7 (0.400)
		Composite	3.51 (<0.001)	.126 (0.001)	.00406 (<0.001)
**Nursing (16%)**	**Highly related fields**	Raw citations	1148 (0.001)	2076 (<0.001)	31.1 (0.001)
		Composite	3 (<0.001)	.126 (<0.001)	.00359 (<0.001)
**Environmental & Occupational Health (15%)**	**Highly related fields**	Raw citations	4784 (0.026)	-324 (0.786)	11.4 (0.821)
		Composite	3.21 (<0.001)	.0191 (0.744)	.0039 (0.119)
**Behavioral Science & Comparative Psychology (13%)**	**Highly related fields**	Raw citations	3705 (0.004)	1873 (0.035)	45.1 (0.099)
		Composite	3.56 (<0.001)	.0386 (0.450)	.00472 (0.003)
**Mycology & Parasitology (13%)**	**Highly related fields**	Raw citations	8399 (<0.001)	-564 (0.724)	-44 (0.303)
		Composite	3.41 (<0.001)	-.000403 (0.995)	.00227 (0.212)
**General & Internal Medicine (13%)**	**Highly related fields**	Raw citations	12625 (<0.001)	3604 (0.003)	-104 (<0.001)
		Composite	3.29 (<0.001)	.136 (<0.001)	.000181 (0.753)
**Otorhinolaryngology (13%)**	**Highly related fields**	Raw citations	5666 (<0.001)	963 (0.055)	-27.1 (0.054)
		Composite	3.28 (<0.001)	.0898 (0.005)	.00284 (0.002)
**Sociology (13%)**	**Other fields**	Raw citations	2549 (0.007)	1086 (0.221)	59.6 (0.008)
		Composite	3.58 (<0.001)	-.0241 (0.641)	.00402 (0.002)
**Surgery (13%)**	**Highly related fields**	Raw citations	8909 (<0.001)	3918 (<0.001)	-16.3 (0.340)
		Composite	3.43 (<0.001)	.0975 (<0.001)	.00122 (0.052)
**Development Studies (13%)**	**Other fields**	Raw citations	5361 (0.015)	-764 (0.540)	-39.5 (0.458)
		Composite	3.88 (<0.001)	-.167 (0.253)	-.00588 (0.342)
**Drama & Theater (11%)**	**Other fields**	Raw citations	-188 (0.513)	379 (0.116)	21.8 (0.051)
		Composite	2.24 (<0.001)	.246 (0.204)	.0169 (0.065)

© 2022 Ioannidis JPA et al. *JAMA Network Open.*

**General Chemistry (11%)**	**Other fields**	Raw citations	13581 (<0.001)	3801 (0.148)	-74.8 (0.038)
		Composite	3.34 (<0.001)	.285 (<0.001)	.00272 (0.005)
**Dermatology & Venereal Diseases (11%)**	**Highly related fields**	Raw citations	8923 (<0.001)	5520 (<0.001)	-20.9 (0.494)
		Composite	3.61 (<0.001)	.117 (0.018)	.000838 (0.471)
**Acoustics (11%)**	**Other fields**	Raw citations	5868 (<0.001)	299 (0.657)	-37.6 (0.011)
		Composite	3.54 (<0.001)	-.0497 (0.315)	-.00109 (0.315)
**Criminology (10%)**	**Other fields**	Raw citations	2832 (0.012)	1150 (0.263)	55.8 (0.051)
		Composite	3.49 (<0.001)	.0599 (0.338)	.00439 (0.012)
**Evolutionary Biology (10%)**	**Other fields**	Raw citations	4856 (0.041)	3072 (0.196)	139 (0.019)
		Composite	3.62 (<0.001)	.0489 (0.307)	.00645 (<0.001)
**Orthopedics (10%)**	**Highly related fields**	Raw citations	7241 (<0.001)	3837 (<0.001)	1.31 (0.946)
		Composite	3.38 (<0.001)	.171 (<0.001)	.00443 (<0.001)
**Environmental Sciences (9.7%)**	**Other fields**	Raw citations	5136 (<0.001)	4222 (0.001)	34.9 (0.294)
		Composite	3.26 (<0.001)	.136 (0.008)	.00541 (<0.001)
**Distributed Computing (9.4%)**	**Other fields**	Raw citations	1607 (0.331)	5988 (<0.001)	83.7 (0.087)
		Composite	3.13 (<0.001)	.106 (0.188)	.00286 (0.309)
**Social Work (9.1%)**	**Other fields**	Raw citations	945 (0.120)	1935 (0.001)	28.5 (0.097)
		Composite	3.2 (<0.001)	.163 (0.027)	.0021 (0.355)
**Social Sciences Methods (9.1%)**	**Other fields**	Raw citations	5776 (0.216)	8448 (0.141)	69.6 (0.451)
		Composite	3.57 (<0.001)	.38 (0.002)	.00476 (0.017)
**Artificial Intelligence & Image Processing (8.9%)**	**Other fields**	Raw citations	5549 (<0.001)	1884 (0.055)	66.1 (0.010)
		Composite	3.18 (<0.001)	.0864 (0.011)	.00835 (<0.001)
**Complementary & Alternative Medicine (8.6%)**	**Highly related fields**	Raw citations	2381 (0.006)	2062 (0.029)	-7.06 (0.782)
		Composite	3.01 (<0.001)	.205 (0.134)	-.00102 (0.785)
**Dentistry (8%)**	**Highly related fields**	Raw citations	5242 (<0.001)	1039 (0.167)	-8.8 (0.605)
		Composite	3.34 (<0.001)	.0697 (0.124)	.00266 (0.010)

© 2022 Ioannidis JPA et al. *JAMA Network Open.*

**Veterinary Sciences (7.9%)**	**Highly related fields**	Raw citations	3394 (<0.001)	876 (0.062)	13.5 (0.291)
		Composite	3.25 (<0.001)	.0165 (0.618)	.00259 (0.004)
**Pathology (7.8%)**	**Highly related fields**	Raw citations	14924 (<0.001)	573 (0.804)	-68 (0.207)
		Composite	3.56 (<0.001)	.065 (0.346)	.00296 (0.066)
**Plant Biology & Botany (7.8%)**	**Other fields**	Raw citations	7649 (<0.001)	6088 (<0.001)	-4.17 (0.843)
		Composite	3.44 (<0.001)	.203 (<0.001)	.00333 (<0.001)
**Gender Studies (7.7%)**	**Highly related fields**	Raw citations	3443 (0.159)	542 (0.741)	-16.7 (0.778)
		Composite	3.41 (<0.001)	.106 (0.566)	.00303 (0.649)
**Social Psychology (7.7%)**	**Highly related fields**	Raw citations	7636 (<0.001)	2379 (0.206)	74.5 (0.031)
		Composite	3.71 (<0.001)	.101 (0.080)	.004 (<0.001)
**Geography (7.6%)**	**Other fields**	Raw citations	1761 (0.127)	2708 (0.027)	62.5 (0.031)
		Composite	3.5 (<0.001)	.167 (0.071)	.00574 (0.010)
**Sport Sciences (7.5%)**	**Highly related fields**	Raw citations	3908 (0.073)	759 (0.753)	103 (0.072)
		Composite	3.45 (<0.001)	-.032 (0.692)	.00384 (0.045)
**Industrial Engineering & Automation (7.3%)**	**Other fields**	Raw citations	5873 (<0.001)	1191 (0.197)	4.07 (0.846)
		Composite	3.39 (<0.001)	.0566 (0.291)	.00338 (0.006)
**Chemical Physics (7.3%)**	**Other fields**	Raw citations	11369 (<0.001)	6593 (0.002)	46.4 (0.186)
		Composite	3.76 (<0.001)	.173 (<0.001)	.00223 (0.002)
**Urban & Regional Planning (6.6%)**	**Other fields**	Raw citations	3722 (0.006)	1254 (0.409)	-4.24 (0.897)
		Composite	3.58 (<0.001)	.0714 (0.622)	.000918 (0.768)
**Sport, Leisure & Tourism (6.5%)**	**Other fields**	Raw citations	3171 (0.010)	-1116 (0.449)	46.2 (0.240)
		Composite	3.44 (<0.001)	-.0313 (0.793)	.00611 (0.058)
**Entomology (6.1%)**	**Other fields**	Raw citations	4771 (<0.001)	3014 (<0.001)	-12.6 (0.394)
		Composite	3.49 (<0.001)	.172 (0.004)	.000408 (0.717)
**Human Factors (6%)**	**Highly related fields**	Raw citations	4439 (<0.001)	540 (0.716)	62.8 (0.070)
		Composite	3.41 (<0.001)	.0738 (0.399)	.00702 (0.001)

© 2022 Ioannidis JPA et al. *JAMA Network Open.*

**Information & Library Sciences (6%)**	**Other fields**	Raw citations	635 (0.285)	792 (0.355)	44.8 (0.008)
		Composite	3.02 (<0.001)	.0433 (0.699)	.00684 (0.002)
**Numerical & Computational Mathematics (5.8%)**	**Other fields**	Raw citations	1557 (0.312)	4150 (0.012)	77.1 (0.027)
		Composite	3.57 (<0.001)	.0519 (0.598)	.00122 (0.557)
**Economics (5.7%)**	**Other fields**	Raw citations	6202 (<0.001)	2505 (0.069)	14.9 (0.537)
		Composite	3.61 (<0.001)	.162 (0.005)	.00448 (<0.001)
**Economic Theory (5.6%)**	**Other fields**	Raw citations	2749 (0.150)	-9.76 (0.996)	20 (0.616)
		Composite	3.49 (<0.001)	-.145 (0.287)	.0029 (0.285)
**Classics (5.6%)**	**Other fields**	Raw citations	1821 (0.045)	-692 (0.508)	-26.5 (0.248)
		Composite	2.97 (<0.001)	-.138 (0.522)	.000834 (0.857)
**Anatomy & Morphology (5.6%)**	**Highly related fields**	Raw citations	2839 (0.003)	-414 (0.790)	-13.7 (0.524)
		Composite	2.98 (<0.001)	-.262 (0.082)	.00394 (0.058)
**General Clinical Medicine (5.5%)**	**Highly related fields**	Raw citations	4671 (0.006)	4784 (0.020)	-12.7 (0.725)
		Composite	2.98 (<0.001)	.317 (0.023)	.00422 (0.084)
**Logistics & Transportation (5.5%)**	**Other fields**	Raw citations	3164 (<0.001)	-717 (0.465)	14.1 (0.480)
		Composite	3.22 (<0.001)	-.0589 (0.546)	.00424 (0.033)
**Computer Hardware & Architecture (5.3%)**	**Other fields**	Raw citations	5136 (<0.001)	264 (0.833)	11.5 (0.674)
		Composite	3.17 (<0.001)	-.0908 (0.297)	.00655 (0.001)
**Polymers (5.3%)**	**Other fields**	Raw citations	9249 (<0.001)	3006 (0.159)	-15.4 (0.632)
		Composite	3.51 (<0.001)	.0498 (0.477)	.00171 (0.106)
**Communication & Media Studies (5.2%)**	**Other fields**	Raw citations	998 (0.281)	336 (0.827)	81.3 (0.002)
		Composite	3.45 (<0.001)	.0191 (0.820)	.00337 (0.015)
**Fluids & Plasmas (5.1%)**	**Other fields**	Raw citations	12731 (<0.001)	4258 (0.143)	-68.1 (0.197)
		Composite	3.71 (<0.001)	.131 (0.053)	.00171 (0.165)
**Software Engineering (4.8%)**	**Other fields**	Raw citations	7307 (<0.001)	2275 (0.033)	-58.5 (0.016)
		Composite	3.51 (<0.001)	.0251 (0.744)	.000579 (0.740)

© 2022 Ioannidis JPA et al. *JAMA Network Open.*

**Electrical & Electronic Engineering (4.8%)**	**Other fields**	Raw citations	3331 (<0.001)	634 (0.574)	21.1 (0.226)
		Composite	3.03 (<0.001)	.0114 (0.889)	.00382 (0.003)
**Optoelectronics & Photonics (4.7%)**	**Other fields**	Raw citations	3129 (<0.001)	2733 (0.001)	30.6 (0.040)
		Composite	2.99 (<0.001)	.105 (0.043)	.00586 (<0.001)
**Marketing (4.7%)**	**Other fields**	Raw citations	2400 (0.242)	-1934 (0.440)	158 (0.008)
		Composite	3.56 (<0.001)	-.0956 (0.337)	.00476 (0.042)
**Languages & Linguistics (4.5%)**	**Other fields**	Raw citations	1366 (0.015)	242 (0.744)	29 (0.048)
		Composite	3.36 (<0.001)	.0355 (0.734)	.00331 (0.108)
**Education (4.5%)**	**Other fields**	Raw citations	1702 (<0.001)	2779 (<0.001)	44.1 (<0.001)
		Composite	3.24 (<0.001)	.179 (<0.001)	.00502 (<0.001)
**Networking & Telecommunications (4.5%)**	**Other fields**	Raw citations	5917 (<0.001)	-593 (0.489)	-8.36 (0.510)
		Composite	3.18 (<0.001)	-.0431 (0.339)	.0045 (<0.001)
**Religions & Theology (4.4%)**	**Other fields**	Raw citations	1003 (0.019)	-52.6 (0.946)	-2.99 (0.780)
		Composite	2.93 (<0.001)	.0854 (0.587)	.0000947 (0.965)
**Archaeology (4.4%)**	**Other fields**	Raw citations	2107 (0.003)	-679 (0.540)	15.6 (0.373)
		Composite	3.23 (<0.001)	-.102 (0.356)	.00425 (0.016)
**History of Social Sciences (4.3%)**	**Other fields**	Raw citations	-424 (0.872)	-814 (0.790)	71.3 (0.219)
		Composite	2.89 (<0.001)	-.0518 (0.859)	.0119 (0.038)
**Applied Mathematics (4.3%)**	**Other fields**	Raw citations	2859 (0.345)	10207 (0.017)	117 (0.087)
		Composite	3.49 (<0.001)	.229 (0.136)	.00551 (0.027)
**Mathematical Physics (4.2%)**	**Other fields**	Raw citations	4008 (0.501)	-2176 (0.761)	18.9 (0.855)
		Composite	3.89 (<0.001)	-.358 (0.346)	-.00453 (0.409)
**Agricultural Economics & Policy (4.1%)**	**Other fields**	Raw citations	6524 (<0.001)	-2108 (0.301)	-30.6 (0.434)
		Composite	3.68 (<0.001)	-.13 (0.357)	-.000426 (0.875)
**Food Science (4.1%)**	**Other fields**	Raw citations	5243 (<0.001)	3202 (0.119)	10.4 (0.736)
		Composite	3.36 (<0.001)	.0863 (0.367)	.00278 (0.054)

**Anthropology (4.1%)**	**Other fields**	Raw citations	2374 (0.011)	-19.9 (0.985)	38.4 (0.066)
		Composite	3.5 (<0.001)	-.0445 (0.639)	.00387 (0.037)
**Science Studies (3.8%)**	**Other fields**	Raw citations	4042 (0.329)	270 (0.950)	27 (0.772)
		Composite	3.7 (<0.001)	-.141 (0.648)	.00151 (0.821)
**Information Systems (3.8%)**	**Other fields**	Raw citations	5783 (0.003)	46.2 (0.986)	64.6 (0.243)
		Composite	3.48 (<0.001)	.00696 (0.945)	.00482 (0.021)
**Finance (3.8%)**	**Other fields**	Raw citations	3543 (0.046)	-1360 (0.597)	88.2 (0.058)
		Composite	3.47 (<0.001)	.00115 (0.993)	.00535 (0.019)
**Cultural Studies (3.7%)**	**Other fields**	Raw citations	1081 (0.052)	-366 (0.650)	2.31 (0.852)
		Composite	3.24 (<0.001)	-.0615 (0.710)	-.0011 (0.664)
**General Physics (3.7%)**	**Other fields**	Raw citations	10053 (<0.001)	1077 (0.632)	-33.2 (0.231)
		Composite	3.5 (<0.001)	.0829 (0.314)	.00254 (0.013)
**Chemical Engineering (3.6%)**	**Other fields**	Raw citations	7632 (<0.001)	1851 (0.260)	-40.8 (0.052)
		Composite	3.45 (<0.001)	.0862 (0.362)	.000512 (0.672)
**Mechanical Engineering & Transports (3.5%)**	**Other fields**	Raw citations	4647 (<0.001)	3195 (0.012)	-2.2 (0.902)
		Composite	3.29 (<0.001)	.0219 (0.771)	.00312 (0.003)
**Ecology (3.5%)**	**Other fields**	Raw citations	6411 (<0.001)	505 (0.803)	116 (0.003)
		Composite	3.55 (<0.001)	.107 (0.054)	.00732 (<0.001)
**Operations Research (3.4%)**	**Other fields**	Raw citations	820 (0.549)	-1333 (0.477)	125 (<0.001)
		Composite	3.45 (<0.001)	-.0873 (0.397)	.00559 (0.003)
**Dairy & Animal Science (3.3%)**	**Other fields**	Raw citations	4591 (<0.001)	1254 (0.128)	-13.1 (0.278)
		Composite	3.26 (<0.001)	.0906 (0.211)	.00125 (0.237)
**Mining & Metallurgy (3.2%)**	**Other fields**	Raw citations	-841 (0.365)	4010 (0.012)	65.3 (0.005)
		Composite	2.31 (<0.001)	.515 (0.054)	.0149 (<0.001)
**Building & Construction (3.1%)**	**Other fields**	Raw citations	2248 (0.020)	1421 (0.421)	53.4 (0.055)
		Composite	3.13 (<0.001)	.0225 (0.880)	.00739 (0.002)

**Philosophy (3.1%)**	**Other fields**	Raw citations	1531 (<0.001)	1526 (0.004)	4.39 (0.514)
		Composite	3.4 (<0.001)	.181 (0.088)	.000582 (0.667)
**Political Science & Public Administration (3.1%)**	**Other fields**	Raw citations	3454 (0.001)	2617 (0.158)	34.4 (0.178)
		Composite	3.63 (<0.001)	.0339 (0.715)	.00198 (0.124)
**Zoology (3%)**	**Other fields**	Raw citations	1876 (0.023)	3975 (0.004)	20.4 (0.345)
		Composite	3.17 (<0.001)	.102 (0.435)	.00193 (0.358)
**Ornithology (3%)**	**Other fields**	Raw citations	3524 (0.001)	-1396 (0.434)	.408 (0.985)
		Composite	3.28 (<0.001)	-.0295 (0.867)	.00454 (0.047)
**Design Practice & Management (3%)**	**Other fields**	Raw citations	3386 (<0.001)	1663 (0.313)	11 (0.591)
		Composite	3.27 (<0.001)	-.0199 (0.902)	.00196 (0.330)
**Materials (2.9%)**	**Other fields**	Raw citations	6797 (<0.001)	2686 (0.029)	-25.3 (0.062)
		Composite	3.27 (<0.001)	.193 (0.002)	.00243 (<0.001)
**Environmental Engineering (2.9%)**	**Other fields**	Raw citations	5644 (<0.001)	1163 (0.395)	-4.68 (0.822)
		Composite	3.4 (<0.001)	.0259 (0.747)	.00278 (0.023)
**Inorganic & Nuclear Chemistry (2.8%)**	**Other fields**	Raw citations	5716 (0.005)	206 (0.951)	67.7 (0.096)
		Composite	3.4 (<0.001)	-.122 (0.283)	.0048 (0.001)
**Geological & Geomatics Engineering (2.8%)**	**Other fields**	Raw citations	5789 (<0.001)	4426 (0.011)	11 (0.684)
		Composite	3.3 (<0.001)	.194 (0.043)	.00536 (<0.001)
**Oceanography (2.7%)**	**Other fields**	Raw citations	5601 (<0.001)	2050 (0.281)	3.64 (0.901)
		Composite	3.38 (<0.001)	.16 (0.114)	.00539 (0.001)
**Literary Studies (2.5%)**	**Other fields**	Raw citations	260 (0.257)	-17.2 (0.971)	10.6 (0.151)
		Composite	2.63 (<0.001)	.0703 (0.606)	.00518 (0.016)
**Meteorology & Atmospheric Sciences (2.5%)**	**Other fields**	Raw citations	10959 (<0.001)	-2892 (0.123)	15.1 (0.589)
		Composite	3.68 (<0.001)	-.0241 (0.648)	.0029 (<0.001)
**Applied Physics (2.4%)**	**Other fields**	Raw citations	10388 (<0.001)	2418 (0.183)	-13.5 (0.458)
		Composite	3.52 (<0.001)	.104 (0.037)	.00198 (<0.001)

© 2022 Ioannidis JPA et al. *JAMA Network Open.*

**Econometrics (2.4%)**	**Other fields**	Raw citations	3434 (0.505)	5372 (0.544)	183 (0.169)
		Composite	3.54 (<0.001)	-.211 (0.521)	.0111 (0.029)
**Business & Management (2.3%)**	**Other fields**	Raw citations	5600 (<0.001)	-1132 (0.562)	67.4 (0.011)
		Composite	3.63 (<0.001)	-.0147 (0.848)	.0036 (0.001)
**Computation Theory & Mathematics (2.3%)**	**Other fields**	Raw citations	4982 (<0.001)	1821 (0.459)	36 (0.263)
		Composite	3.53 (<0.001)	.082 (0.546)	.00473 (0.008)
**Aerospace & Aeronautics (2.3%)**	**Other fields**	Raw citations	2314 (<0.001)	379 (0.528)	8.24 (0.284)
		Composite	3.08 (<0.001)	-.0131 (0.865)	.0029 (0.003)
**Strategic, Defence & Security Studies (2.2%)**	**Other fields**	Raw citations	1219 (0.187)	-102 (0.959)	55 (0.026)
		Composite	3.14 (<0.001)	.227 (0.141)	.00571 (0.003)
**General Mathematics (2.2%)**	**Other fields**	Raw citations	3090 (<0.001)	-413 (0.660)	-.84 (0.920)
		Composite	3.47 (<0.001)	-.0642 (0.385)	.000699 (0.286)
**Civil Engineering (2.2%)**	**Other fields**	Raw citations	1891 (0.014)	198 (0.897)	36 (0.059)
		Composite	3.09 (<0.001)	-.00369 (0.978)	.00467 (0.006)
**Geology (2.2%)**	**Other fields**	Raw citations	5985 (<0.001)	-1123 (0.524)	-38.9 (0.068)
		Composite	3.64 (<0.001)	-.00126 (0.994)	-.00216 (0.276)
**Energy (2.1%)**	**Other fields**	Raw citations	4520 (<0.001)	1691 (0.099)	8.05 (0.458)
		Composite	3.2 (<0.001)	.0679 (0.296)	.00376 (<0.001)
**Psychoanalysis (2%)**	**Highly related fields**	Raw citations	988 (0.497)	2357 (0.380)	15.2 (0.529)
		Composite	3.41 (<0.001)	.29 (0.276)	-.000634 (0.791)
**Law (1.9%)**	**Other fields**	Raw citations	742 (0.443)	-514 (0.749)	32.6 (0.314)
		Composite	3.11 (<0.001)	-.119 (0.453)	.0062 (0.054)
**Marine Biology & Hydrobiology (1.8%)**	**Other fields**	Raw citations	7982 (<0.001)	325 (0.891)	-4.01 (0.905)
		Composite	3.67 (<0.001)	.029 (0.767)	.0022 (0.113)
**Astronomy & Astrophysics (1.8%)**	**Other fields**	Raw citations	18941 (<0.001)	-6369 (0.107)	-91.4 (0.048)
		Composite	3.75 (<0.001)	.0463 (0.466)	.00118 (0.113)

© 2022 Ioannidis JPA et al. *JAMA Network Open.*

**Fisheries (1.8%)**	**Other fields**	Raw citations	4259 (<0.001)	519 (0.751)	5.37 (0.812)
		Composite	3.42 (<0.001)	.0749 (0.559)	.00126 (0.478)
**Legal & Forensic Medicine (1.5%)**	**Highly related fields**	Raw citations	1711 (0.119)	-1184 (0.649)	12.3 (0.661)
		Composite	2.93 (<0.001)	-.158 (0.565)	.00263 (0.376)
**International Relations (1.5%)**	**Other fields**	Raw citations	2208 (0.016)	-216 (0.926)	27.4 (0.237)
		Composite	3.43 (<0.001)	.0318 (0.892)	.00393 (0.096)
**Nuclear & Particle Physics (1.3%)**	**Other fields**	Raw citations	18699 (<0.001)	-7166 (0.161)	-103 (0.002)
		Composite	3.65 (<0.001)	-.0646 (0.464)	.00118 (0.042)
**Geochemistry & Geophysics (1.2%)**	**Other fields**	Raw citations	7325 (<0.001)	-1353 (0.415)	15.3 (0.348)
		Composite	3.66 (<0.001)	-.12 (0.125)	.00309 (<0.001)
**Forestry (.96%)**	**Other fields**	Raw citations	2792 (<0.001)	-1115 (0.520)	16.6 (0.374)
		Composite	3.15 (<0.001)	-.0613 (0.739)	.00375 (0.060)
**Physical Chemistry (.88%)**	**Other fields**	Raw citations	11640 (<0.001)	-5354 (0.568)	-38.2 (0.499)
		Composite	3.49 (<0.001)	-.247 (0.517)	.00373 (0.107)
**Agronomy & Agriculture (.88%)**	**Other fields**	Raw citations	5470 (<0.001)	1660 (0.508)	-.603 (0.975)
		Composite	3.36 (<0.001)	.106 (0.453)	.00276 (0.010)
**Paleontology (0%)**	**Other fields**	Raw citations			
		Composite			
**Music (0%)**	**Other fields**	Raw citations			
		Composite			
**Industrial Relations (0%)**	**Other fields**	Raw citations			
		Composite			
**Horticulture (0%)**	**Other fields**	Raw citations			
		Composite			
**History of Science, Technology & Medicine (0%)**	**Other fields**	Raw citations			
		Composite			
**History (0%)**	**Other fields**	Raw citations			
		Composite			
**General Psychology & Cognitive Sciences (0%)**	**Highly related fields**	Raw citations			

© 2022 Ioannidis JPA et al. *JAMA Network Open.*

		Composite			
**Folklore (0%)**	**Other fields**	Raw citations			
		Composite			
**Automobile Design & Engineering (0%)**	**Other fields**	Raw citations			
		Composite			
**Art Practice, History & Theory (0%)**	**Other fields**	Raw citations			
		Composite			
**Architecture (0%)**	**Other fields**	Raw citations			
		Composite			
**Accounting (0%)**	**Other fields**	Raw citations			
		Composite			

© 2022 Ioannidis JPA et al. *JAMA Network Open.*

eTable 3.3 : Career-long impact, Funding time current funding Linear Regressions for each subfield (ordered by percentage funded)

Top-cited US-based researchers: Subfield (perc. funded)	Classification	Dependent Variable	Constant (p-val)	Funded (p-val)	Years since first pub (p-val)
**Geriatrics (31%)**	**Highly related fields**	Raw citations	7240 (0.221)	2666 (0.364)	128 (0.384)
		Composite	3.33 (<0.001)	.0879 (0.257)	.00962 (0.015)
**Bioinformatics (30%)**	**Highly related fields**	Raw citations	18202 (0.005)	3599 (0.376)	-2.28 (0.990)
		Composite	3.41 (<0.001)	.102 (0.045)	.00664 (0.003)
**Developmental Biology (29%)**	**Highly related fields**	Raw citations	19299 (<0.001)	1961 (0.165)	31.3 (0.607)
		Composite	3.6 (<0.001)	.0117 (0.505)	.00753 (<0.001)
**Substance Abuse (23%)**	**Highly related fields**	Raw citations	3955 (0.023)	1569 (0.131)	142 (<0.001)
		Composite	3.63 (<0.001)	-.0224 (0.555)	.00412 (0.005)
**Medical Informatics (23%)**	**Highly related fields**	Raw citations	3976 (0.073)	3468 (0.039)	28.4 (0.625)
		Composite	3.21 (<0.001)	.18 (0.010)	.00432 (0.073)
**Virology (22%)**	**Highly related fields**	Raw citations	9012 (<0.001)	1496 (0.119)	89 (0.015)
		Composite	3.45 (<0.001)	.0466 (0.072)	.00569 (<0.001)
**Biomedical Engineering (22%)**	**Highly related fields**	Raw citations	4808 (0.003)	3856 (0.002)	72.1 (0.073)
		Composite	3.21 (<0.001)	.0523 (0.144)	.00655 (<0.001)
**Neurology & Neurosurgery (21%)**	**Highly related fields**	Raw citations	14167 (<0.001)	729 (0.272)	26.9 (0.255)
		Composite	3.67 (<0.001)	.0196 (0.154)	.00433 (<0.001)
**Immunology (20%)**	**Highly related fields**	Raw citations	16782 (<0.001)	2421 (0.033)	47.7 (0.244)
		Composite	3.67 (<0.001)	.0824 (<0.001)	.00456 (<0.001)
**Gerontology (20%)**	**Highly related fields**	Raw citations	15511 (0.061)	1837 (0.700)	-102 (0.601)
		Composite	3.53 (<0.001)	.09 (0.203)	.00419 (0.150)
**Applied Ethics (20%)**	**Highly related fields**	Raw citations	5381 (0.001)	2541 (0.013)	-24.2 (0.518)
		Composite	3.49 (<0.001)	.0413 (0.578)	.00342 (0.222)

© 2022 Ioannidis JPA et al. *JAMA Network Open.*

**Public Health (19%)**	**Highly related fields**	Raw citations	4564 (0.005)	2088 (0.054)	158 (<0.001)
		Composite	3.47 (<0.001)	.0841 (0.002)	.00642 (<0.001)
**Demography (19%)**	**Highly related fields**	Raw citations	-619 (0.831)	1038 (0.584)	117 (0.057)
		Composite	3.36 (<0.001)	-.0231 (0.837)	.00644 (0.078)
**Oncology & Carcinogenesis (18%)**	**Highly related fields**	Raw citations	17848 (<0.001)	1549 (0.114)	17.1 (0.603)
		Composite	3.5 (<0.001)	.0143 (0.370)	.00598 (<0.001)
**Gastroenterology & Hepatology (17%)**	**Highly related fields**	Raw citations	11847 (<0.001)	1338 (0.301)	34 (0.414)
		Composite	3.61 (<0.001)	.0665 (0.050)	.00373 (0.001)
**Allergy (16%)**	**Highly related fields**	Raw citations	10372 (0.003)	681 (0.782)	43.7 (0.579)
		Composite	3.63 (<0.001)	.0272 (0.714)	.00373 (0.118)
**Biophysics (16%)**	**Highly related fields**	Raw citations	5031 (0.147)	2001 (0.421)	86.4 (0.247)
		Composite	3.33 (<0.001)	.054 (0.458)	.00629 (0.004)
**Developmental & Child Psychology (16%)**	**Highly related fields**	Raw citations	4485 (0.006)	4997 (<0.001)	123 (0.002)
		Composite	3.57 (<0.001)	.148 (<0.001)	.006 (<0.001)
**Health Policy & Services (16%)**	**Highly related fields**	Raw citations	1789 (0.491)	577 (0.758)	216 (0.001)
		Composite	3.29 (<0.001)	.0579 (0.285)	.0104 (<0.001)
**Physiology (16%)**	**Highly related fields**	Raw citations	6256 (<0.001)	-514 (0.575)	36.5 (0.149)
		Composite	3.72 (<0.001)	-.00535 (0.902)	.00137 (0.252)
**Arthritis & Rheumatology (16%)**	**Highly related fields**	Raw citations	10770 (0.004)	1837 (0.478)	154 (0.080)
		Composite	3.64 (<0.001)	.0118 (0.842)	.005 (0.014)
**Epidemiology (15%)**	**Highly related fields**	Raw citations	1001 (0.926)	10141 (0.183)	496 (0.053)
		Composite	3.55 (<0.001)	.0412 (0.589)	.00847 (0.001)
**Psychiatry (15%)**	**Highly related fields**	Raw citations	10580 (<0.001)	4869 (0.002)	131 (0.011)
		Composite	3.68 (<0.001)	.0821 (0.009)	.00484 (<0.001)
**Genetics & Heredity (15%)**	**Highly related fields**	Raw citations	19705 (<0.001)	4478 (0.057)	-75.7 (0.353)
		Composite	3.57 (<0.001)	.00232 (0.955)	.00386 (0.008)

© 2022 Ioannidis JPA et al. *JAMA Network Open.*

**Emergency & Critical Care Medicine (15%)**	**Highly related fields**	Raw citations	6790 (0.007)	4966 (0.005)	82.9 (0.205)
		Composite	3.32 (<0.001)	.106 (0.030)	.00623 (0.001)
**Endocrinology & Metabolism (15%)**	**Highly related fields**	Raw citations	13853 (<0.001)	1278 (0.354)	44 (0.306)
		Composite	3.7 (<0.001)	.0075 (0.798)	.00369 (<0.001)
**Statistics & Probability (14%)**	**Other fields**	Raw citations	11748 (<0.001)	4219 (0.106)	-8.42 (0.898)
		Composite	3.72 (<0.001)	.0768 (0.169)	.00147 (0.298)
**Ophthalmology & Optometry (14%)**	**Highly related fields**	Raw citations	10708 (<0.001)	1751 (0.043)	-55.9 (0.033)
		Composite	3.5 (<0.001)	.0825 (0.007)	.00191 (0.038)
**Microbiology (14%)**	**Highly related fields**	Raw citations	12953 (<0.001)	1918 (0.026)	-35.4 (0.171)
		Composite	3.54 (<0.001)	.0542 (0.017)	.0046 (<0.001)
**Cardiovascular System & Hematology (14%)**	**Highly related fields**	Raw citations	19446 (<0.001)	3580 (0.014)	17.2 (0.689)
		Composite	3.62 (<0.001)	.0499 (0.027)	.00472 (<0.001)
**Respiratory System (13%)**	**Highly related fields**	Raw citations	11193 (<0.001)	-24.4 (0.986)	72.1 (0.114)
		Composite	3.56 (<0.001)	.00577 (0.869)	.00455 (<0.001)
**Clinical Psychology (13%)**	**Highly related fields**	Raw citations	7741 (0.001)	1524 (0.473)	84.7 (0.116)
		Composite	3.73 (<0.001)	.0574 (0.389)	.00307 (0.070)
**Nuclear Medicine & Medical Imaging (13%)**	**Highly related fields**	Raw citations	6940 (<0.001)	2480 (<0.001)	31.7 (0.161)
		Composite	3.41 (<0.001)	.0724 (0.004)	.00238 (0.003)
**Analytical Chemistry (13%)**	**Other fields**	Raw citations	5901 (<0.001)	3309 (0.001)	18.4 (0.467)
		Composite	3.29 (<0.001)	.0761 (0.071)	.00475 (<0.001)
**Medicinal & Biomolecular Chemistry (12%)**	**Highly related fields**	Raw citations	1538 (0.083)	2902 (<0.001)	113 (<0.001)
		Composite	2.93 (<0.001)	.12 (0.002)	.00877 (<0.001)
**Nanoscience & Nanotechnology (12%)**	**Other fields**	Raw citations	7634 (0.009)	449 (0.891)	497 (<0.001)
		Composite	3.3 (<0.001)	.0263 (0.617)	.0143 (<0.001)
**Urology & Nephrology (12%)**	**Highly related fields**	Raw citations	14065 (<0.001)	1537 (0.237)	-45.1 (0.238)
		Composite	3.58 (<0.001)	.0714 (0.031)	.00308 (0.002)

© 2022 Ioannidis JPA et al. *JAMA Network Open.*

**Optics (12%)**	**Other fields**	Raw citations	7949 (<0.001)	4544 (0.001)	1.31 (0.973)
		Composite	3.26 (<0.001)	.0423 (0.410)	.00481 (0.001)
**Biochemistry & Molecular Biology (12%)**	**Highly related fields**	Raw citations	12813 (<0.001)	1695 (0.062)	10.4 (0.666)
		Composite	3.68 (<0.001)	.0445 (0.035)	.00326 (<0.001)
**Nutrition & Dietetics (11%)**	**Highly related fields**	Raw citations	8095 (<0.001)	2690 (0.118)	43.3 (0.344)
		Composite	3.52 (<0.001)	.047 (0.364)	.00527 (<0.001)
**Experimental Psychology (11%)**	**Highly related fields**	Raw citations	6337 (<0.001)	4191 (<0.001)	51.2 (0.030)
		Composite	3.67 (<0.001)	.157 (<0.001)	.0037 (<0.001)
**Rehabilitation (11%)**	**Highly related fields**	Raw citations	3920 (0.001)	2184 (0.012)	34.2 (0.235)
		Composite	3.3 (<0.001)	.0716 (0.182)	.00462 (0.010)
**Toxicology (11%)**	**Highly related fields**	Raw citations	8361 (<0.001)	4376 (<0.001)	-29 (0.363)
		Composite	3.35 (<0.001)	.114 (0.009)	.00394 (0.002)
**Drama & Theater (11%)**	**Other fields**	Raw citations	-188 (0.513)	379 (0.116)	21.8 (0.051)
		Composite	2.24 (<0.001)	.246 (0.204)	.0169 (0.065)
**Obstetrics & Reproductive Medicine (11%)**	**Highly related fields**	Raw citations	9867 (<0.001)	592 (0.485)	-44.4 (0.054)
		Composite	3.51 (<0.001)	.0377 (0.273)	.00197 (0.034)
**Pediatrics (11%)**	**Highly related fields**	Raw citations	6378 (<0.001)	1513 (0.037)	16.2 (0.382)
		Composite	3.3 (<0.001)	.0813 (0.016)	.00428 (<0.001)
**Anesthesiology (11%)**	**Highly related fields**	Raw citations	6214 (<0.001)	3010 (0.001)	5.13 (0.845)
		Composite	3.41 (<0.001)	.0984 (0.041)	.00223 (0.099)
**Pharmacology & Pharmacy (10%)**	**Highly related fields**	Raw citations	5187 (<0.001)	1667 (0.031)	28.8 (0.125)
		Composite	3.32 (<0.001)	.0433 (0.245)	.00339 (<0.001)
**Speech-Language Pathology & Audiology (9.9%)**	**Highly related fields**	Raw citations	6996 (<0.001)	-1163 (0.368)	-55.1 (0.197)
		Composite	3.36 (<0.001)	-.0243 (0.758)	.00426 (0.103)
**Organic Chemistry (9.8%)**	**Other fields**	Raw citations	8871 (<0.001)	3838 (0.007)	15.3 (0.587)
		Composite	3.52 (<0.001)	.121 (0.007)	.00385 (<0.001)

© 2022 Ioannidis JPA et al. *JAMA Network Open.*

**Environmental & Occupational Health (9.8%)**	**Highly related fields**	Raw citations	5081 (0.019)	-1098 (0.453)	5.49 (0.914)
		Composite	3.22 (<0.001)	-.000389 (0.996)	.00377 (0.136)
**Family Studies (8.9%)**	**Other fields**	Raw citations	180 (0.895)	1669 (0.209)	96.4 (0.007)
		Composite	3.18 (<0.001)	.0128 (0.898)	.00977 (<0.001)
**Development Studies (8.3%)**	**Other fields**	Raw citations	5159 (0.019)	-48.8 (0.974)	-36.5 (0.494)
		Composite	3.86 (<0.001)	-.177 (0.310)	-.00532 (0.389)
**Mycology & Parasitology (8.2%)**	**Highly related fields**	Raw citations	8329 (<0.001)	-479 (0.807)	-43.2 (0.313)
		Composite	3.4 (<0.001)	.0122 (0.883)	.00232 (0.202)
**Nursing (7.9%)**	**Highly related fields**	Raw citations	1304 (<0.001)	2989 (<0.001)	29.4 (0.001)
		Composite	3.01 (<0.001)	.173 (<0.001)	.00347 (<0.001)
**Surgery (7.9%)**	**Highly related fields**	Raw citations	9513 (<0.001)	3110 (0.001)	-24.9 (0.147)
		Composite	3.45 (<0.001)	.0473 (0.152)	.000912 (0.147)
**Tropical Medicine (7.8%)**	**Highly related fields**	Raw citations	8018 (<0.001)	2713 (0.097)	-33.2 (0.314)
		Composite	3.38 (<0.001)	.0795 (0.276)	.00152 (0.304)
**General Chemistry (7.8%)**	**Other fields**	Raw citations	13531 (<0.001)	5432 (0.075)	-74 (0.039)
		Composite	3.36 (<0.001)	.302 (<0.001)	.00251 (0.009)
**Complementary & Alternative Medicine (7.4%)**	**Highly related fields**	Raw citations	2244 (0.010)	2381 (0.019)	-2.69 (0.916)
		Composite	3 (<0.001)	.259 (0.078)	-.00054 (0.885)
**General & Internal Medicine (7.1%)**	**Highly related fields**	Raw citations	13326 (<0.001)	2641 (0.097)	-114 (<0.001)
		Composite	3.31 (<0.001)	.113 (0.006)	-.000135 (0.813)
**Biotechnology (7%)**	**Highly related fields**	Raw citations	4261 (0.009)	1527 (0.415)	92.5 (0.036)
		Composite	3.12 (<0.001)	.159 (0.053)	.00851 (<0.001)
**Microscopy (6.7%)**	**Highly related fields**	Raw citations	6035 (0.129)	3451 (0.362)	23.3 (0.796)
		Composite	3.26 (<0.001)	.322 (0.163)	.0056 (0.307)
**Acoustics (6.5%)**	**Other fields**	Raw citations	5840 (<0.001)	570 (0.497)	-37.1 (0.012)
		Composite	3.53 (<0.001)	-.0568 (0.357)	-.00103 (0.336)

© 2022 Ioannidis JPA et al. *JAMA Network Open.*

**Sociology (6.4%)**	**Other fields**	Raw citations	2704 (0.004)	609 (0.617)	58.2 (0.010)
		Composite	3.57 (<0.001)	.00735 (0.917)	.0041 (0.002)
**Otorhinolaryngology (6.3%)**	**Highly related fields**	Raw citations	5652 (<0.001)	1622 (0.018)	-26.2 (0.061)
		Composite	3.29 (<0.001)	.0981 (0.026)	.00272 (0.002)
**Behavioral Science & Comparative Psychology (6.3%)**	**Highly related fields**	Raw citations	3888 (0.003)	1817 (0.142)	43.9 (0.110)
		Composite	3.57 (<0.001)	.0329 (0.644)	.00469 (0.003)
**Orthopedics (5.9%)**	**Highly related fields**	Raw citations	7766 (<0.001)	3284 (0.001)	-6.91 (0.723)
		Composite	3.4 (<0.001)	.157 (<0.001)	.0041 (<0.001)
**Sport Sciences (5.9%)**	**Highly related fields**	Raw citations	3800 (0.079)	1673 (0.534)	105 (0.066)
		Composite	3.45 (<0.001)	-.0187 (0.836)	.0039 (0.041)
**Social Work (5.7%)**	**Other fields**	Raw citations	1135 (0.074)	1393 (0.052)	25.7 (0.151)
		Composite	3.21 (<0.001)	.117 (0.205)	.00187 (0.419)
**Economic Theory (5.6%)**	**Other fields**	Raw citations	2749 (0.150)	-9.76 (0.996)	20 (0.616)
		Composite	3.49 (<0.001)	-.145 (0.287)	.0029 (0.285)
**Evolutionary Biology (5.5%)**	**Other fields**	Raw citations	4994 (0.035)	3554 (0.261)	138 (0.019)
		Composite	3.62 (<0.001)	.0811 (0.203)	.00648 (<0.001)
**Dermatology & Venereal Diseases (5.5%)**	**Highly related fields**	Raw citations	9162 (<0.001)	6568 (<0.001)	-21.1 (0.493)
		Composite	3.62 (<0.001)	.0743 (0.268)	.000661 (0.573)
**Artificial Intelligence & Image Processing (5.5%)**	**Other fields**	Raw citations	5687 (<0.001)	1298 (0.290)	64.8 (0.011)
		Composite	3.19 (<0.001)	.0637 (0.136)	.00829 (<0.001)
**Environmental Sciences (5.5%)**	**Other fields**	Raw citations	5263 (<0.001)	4227 (0.012)	36.3 (0.282)
		Composite	3.27 (<0.001)	.152 (0.024)	.00552 (<0.001)
**Plant Biology & Botany (5.2%)**	**Other fields**	Raw citations	7870 (<0.001)	6208 (<0.001)	-5.94 (0.779)
		Composite	3.44 (<0.001)	.198 (<0.001)	.00326 (0.001)
**Pathology (5.1%)**	**Highly related fields**	Raw citations	15555 (<0.001)	-2297 (0.413)	-79.6 (0.138)
		Composite	3.59 (<0.001)	-.0478 (0.569)	.00245 (0.126)

© 2022 Ioannidis JPA et al. *JAMA Network Open.*

**Chemical Physics (4.8%)**	**Other fields**	Raw citations	12058 (<0.001)	4285 (0.101)	37.7 (0.284)
		Composite	3.77 (<0.001)	.133 (0.011)	.00204 (0.004)
**Industrial Engineering & Automation (4.8%)**	**Other fields**	Raw citations	5880 (<0.001)	1965 (0.078)	3.68 (0.859)
		Composite	3.39 (<0.001)	.0694 (0.284)	.0033 (0.006)
**Entomology (4.8%)**	**Other fields**	Raw citations	4855 (<0.001)	3779 (<0.001)	-14.5 (0.322)
		Composite	3.49 (<0.001)	.231 (<0.001)	.000319 (0.774)
**Criminology (4.6%)**	**Other fields**	Raw citations	2985 (0.008)	756 (0.616)	54 (0.060)
		Composite	3.5 (<0.001)	.0849 (0.353)	.00435 (0.013)
**Religions & Theology (4.4%)**	**Other fields**	Raw citations	1003 (0.019)	-52.6 (0.946)	-2.99 (0.780)
		Composite	2.93 (<0.001)	.0854 (0.587)	.0000947 (0.965)
**Sport, Leisure & Tourism (4.3%)**	**Other fields**	Raw citations	3099 (0.012)	-972 (0.587)	47.7 (0.228)
		Composite	3.44 (<0.001)	-.0256 (0.859)	.00615 (0.057)
**Distributed Computing (4.3%)**	**Other fields**	Raw citations	2342 (0.154)	8703 (<0.001)	66.8 (0.171)
		Composite	3.14 (<0.001)	.217 (0.060)	.00249 (0.374)
**Dentistry (4.3%)**	**Highly related fields**	Raw citations	5429 (<0.001)	548 (0.585)	-11.7 (0.488)
		Composite	3.35 (<0.001)	.043 (0.476)	.00248 (0.015)
**Social Sciences Methods (3.9%)**	**Other fields**	Raw citations	6813 (0.127)	14216 (0.087)	51.6 (0.565)
		Composite	3.63 (<0.001)	.514 (0.005)	.00383 (0.049)
**Science Studies (3.8%)**	**Other fields**	Raw citations	4042 (0.329)	270 (0.950)	27 (0.772)
		Composite	3.7 (<0.001)	-.141 (0.648)	.00151 (0.821)
**Logistics & Transportation (3.8%)**	**Other fields**	Raw citations	3206 (<0.001)	-955 (0.416)	12.7 (0.525)
		Composite	3.24 (<0.001)	-.195 (0.094)	.00382 (0.055)
**Veterinary Sciences (3.8%)**	**Highly related fields**	Raw citations	3398 (<0.001)	1524 (0.022)	13.7 (0.283)
		Composite	3.24 (<0.001)	.0639 (0.173)	.00267 (0.003)
**Social Psychology (3.7%)**	**Highly related fields**	Raw citations	7827 (<0.001)	1501 (0.570)	73 (0.036)
		Composite	3.71 (<0.001)	.125 (0.124)	.004 (<0.001)

© 2022 Ioannidis JPA et al. *JAMA Network Open.*

**Anatomy & Morphology (3.7%)**	**Highly related fields**	Raw citations	2676 (0.005)	858 (0.651)	-10.9 (0.614)
		Composite	2.96 (<0.001)	-.105 (0.571)	.0043 (0.046)
**Applied Mathematics (3.6%)**	**Other fields**	Raw citations	3765 (0.226)	2805 (0.554)	104 (0.139)
		Composite	3.51 (<0.001)	.0465 (0.784)	.00517 (0.040)
**Polymers (3.6%)**	**Other fields**	Raw citations	9496 (<0.001)	2070 (0.418)	-19.1 (0.551)
		Composite	3.52 (<0.001)	-.00218 (0.979)	.0016 (0.128)
**Information & Library Sciences (3.4%)**	**Other fields**	Raw citations	682 (0.257)	172 (0.879)	44.6 (0.009)
		Composite	3.02 (<0.001)	.0424 (0.773)	.00689 (0.002)
**Communication & Media Studies (3.2%)**	**Other fields**	Raw citations	1000 (0.278)	585 (0.762)	81.2 (0.002)
		Composite	3.45 (<0.001)	.0306 (0.771)	.00336 (0.016)
**Mining & Metallurgy (3.2%)**	**Other fields**	Raw citations	-841 (0.365)	4010 (0.012)	65.3 (0.005)
		Composite	2.31 (<0.001)	.515 (0.054)	.0149 (<0.001)
**General Clinical Medicine (3.1%)**	**Highly related fields**	Raw citations	4741 (0.004)	7767 (0.004)	-13.8 (0.698)
		Composite	2.99 (<0.001)	.441 (0.015)	.00414 (0.089)
**Marketing (3.1%)**	**Other fields**	Raw citations	2302 (0.264)	-1698 (0.580)	159 (0.008)
		Composite	3.56 (<0.001)	-.0515 (0.673)	.00476 (0.044)
**Economics (3.1%)**	**Other fields**	Raw citations	6137 (<0.001)	4725 (0.010)	16.6 (0.492)
		Composite	3.61 (<0.001)	.255 (0.001)	.00451 (<0.001)
**Software Engineering (3.1%)**	**Other fields**	Raw citations	7314 (<0.001)	1442 (0.279)	-56.8 (0.020)
		Composite	3.5 (<0.001)	.058 (0.543)	.000601 (0.731)
**Ornithology (3%)**	**Other fields**	Raw citations	3524 (0.001)	-1396 (0.434)	.408 (0.985)
		Composite	3.28 (<0.001)	-.0295 (0.867)	.00454 (0.047)
**Finance (3%)**	**Other fields**	Raw citations	3568 (0.045)	-637 (0.824)	86.6 (0.062)
		Composite	3.48 (<0.001)	.0402 (0.774)	.00527 (0.020)
**Human Factors (3%)**	**Highly related fields**	Raw citations	4424 (<0.001)	451 (0.827)	63.8 (0.066)
		Composite	3.4 (<0.001)	.18 (0.139)	.00723 (<0.001)

© 2022 Ioannidis JPA et al. *JAMA Network Open.*

**Agricultural Economics & Policy (2.7%)**	**Other fields**	Raw citations	6469 (<0.001)	-2200 (0.375)	-29.9 (0.446)
		Composite	3.67 (<0.001)	-.077 (0.654)	-.000359 (0.895)
**Computer Hardware & Architecture (2.6%)**	**Other fields**	Raw citations	5135 (<0.001)	358 (0.838)	11.7 (0.670)
		Composite	3.16 (<0.001)	.0267 (0.827)	.00673 (0.001)
**Networking & Telecommunications (2.6%)**	**Other fields**	Raw citations	5889 (<0.001)	-456 (0.679)	-7.97 (0.529)
		Composite	3.18 (<0.001)	-.00216 (0.970)	.00456 (<0.001)
**Political Science & Public Administration (2.6%)**	**Other fields**	Raw citations	3341 (0.001)	3546 (0.077)	37.1 (0.147)
		Composite	3.62 (<0.001)	.0847 (0.400)	.00208 (0.107)
**Food Science (2.6%)**	**Other fields**	Raw citations	5514 (<0.001)	3799 (0.130)	4.56 (0.880)
		Composite	3.37 (<0.001)	.0365 (0.755)	.00255 (0.072)
**Education (2.6%)**	**Other fields**	Raw citations	1755 (<0.001)	3119 (<0.001)	43.9 (<0.001)
		Composite	3.25 (<0.001)	.199 (0.001)	.005 (<0.001)
**Dairy & Animal Science (2.4%)**	**Other fields**	Raw citations	4689 (<0.001)	522 (0.585)	-14.7 (0.224)
		Composite	3.27 (<0.001)	.0237 (0.778)	.00112 (0.290)
**Econometrics (2.4%)**	**Other fields**	Raw citations	3434 (0.505)	5372 (0.544)	183 (0.169)
		Composite	3.54 (<0.001)	-.211 (0.521)	.0111 (0.029)
**Mechanical Engineering & Transports (2.3%)**	**Other fields**	Raw citations	5149 (<0.001)	-537 (0.734)	-10.7 (0.551)
		Composite	3.31 (<0.001)	-.0951 (0.310)	.00288 (0.007)
**Strategic, Defence & Security Studies (2.2%)**	**Other fields**	Raw citations	1213 (0.190)	205 (0.917)	55 (0.026)
		Composite	3.14 (<0.001)	.175 (0.257)	.00572 (0.003)
**Electrical & Electronic Engineering (2.2%)**	**Other fields**	Raw citations	3374 (<0.001)	547 (0.739)	20.3 (0.241)
		Composite	3.03 (<0.001)	-.0614 (0.604)	.00375 (0.003)
**Geography (2.2%)**	**Other fields**	Raw citations	2410 (0.042)	-36 (0.987)	50.8 (0.089)
		Composite	3.55 (<0.001)	-.0617 (0.718)	.00485 (0.032)
**Chemical Engineering (2.1%)**	**Other fields**	Raw citations	7839 (<0.001)	-1467 (0.491)	-43.2 (0.040)
		Composite	3.46 (<0.001)	-.0154 (0.900)	.000427 (0.724)

© 2022 Ioannidis JPA et al. *JAMA Network Open.*

**General Physics (2%)**	**Other fields**	Raw citations	9983 (<0.001)	2744 (0.359)	-32.1 (0.248)
		Composite	3.5 (<0.001)	.102 (0.351)	.00253 (0.013)
**Optoelectronics & Photonics (2%)**	**Other fields**	Raw citations	3306 (<0.001)	3117 (0.008)	27.7 (0.063)
		Composite	2.99 (<0.001)	.125 (0.108)	.00575 (<0.001)
**Psychoanalysis (2%)**	**Highly related fields**	Raw citations	988 (0.497)	2357 (0.380)	15.2 (0.529)
		Composite	3.41 (<0.001)	.29 (0.276)	-.000634 (0.791)
**Design Practice & Management (2%)**	**Other fields**	Raw citations	3446 (<0.001)	1632 (0.415)	9.81 (0.629)
		Composite	3.26 (<0.001)	.0583 (0.767)	.00209 (0.297)
**Operations Research (2%)**	**Other fields**	Raw citations	758 (0.580)	-544 (0.825)	126 (<0.001)
		Composite	3.44 (<0.001)	.0117 (0.931)	.00566 (0.002)
**Environmental Engineering (2%)**	**Other fields**	Raw citations	5584 (<0.001)	2746 (0.098)	-3.67 (0.859)
		Composite	3.4 (<0.001)	.0912 (0.349)	.00283 (0.020)
**Information Systems (1.9%)**	**Other fields**	Raw citations	5690 (0.004)	2803 (0.455)	65.8 (0.233)
		Composite	3.48 (<0.001)	.0686 (0.625)	.00484 (0.020)
**Literary Studies (1.9%)**	**Other fields**	Raw citations	258 (0.258)	15.9 (0.976)	10.6 (0.150)
		Composite	2.63 (<0.001)	.104 (0.508)	.00514 (0.016)
**Cultural Studies (1.9%)**	**Other fields**	Raw citations	1070 (0.054)	-72.8 (0.949)	2.28 (0.854)
		Composite	3.23 (<0.001)	.11 (0.637)	-.00117 (0.647)
**Languages & Linguistics (1.8%)**	**Other fields**	Raw citations	1367 (0.015)	264 (0.820)	29.1 (0.047)
		Composite	3.36 (<0.001)	.155 (0.341)	.0034 (0.097)
**Fisheries (1.8%)**	**Other fields**	Raw citations	4290 (<0.001)	3379 (0.038)	3.33 (0.882)
		Composite	3.42 (<0.001)	.218 (0.089)	.00115 (0.516)
**Archaeology (1.8%)**	**Other fields**	Raw citations	2083 (0.003)	-452 (0.795)	15.6 (0.373)
		Composite	3.23 (<0.001)	-.0732 (0.673)	.00425 (0.017)
**Computation Theory & Mathematics (1.7%)**	**Other fields**	Raw citations	5046 (<0.001)	-1823 (0.520)	36.2 (0.261)
		Composite	3.53 (<0.001)	-.0894 (0.567)	.00475 (0.008)

© 2022 Ioannidis JPA et al. *JAMA Network Open.*

**Numerical & Computational Mathematics (1.7%)**	**Other fields**	Raw citations	1528 (0.329)	5377 (0.080)	81.3 (0.022)
		Composite	3.56 (<0.001)	.335 (0.062)	.00149 (0.467)
**Urban & Regional Planning (1.6%)**	**Other fields**	Raw citations	3774 (0.005)	-145 (0.961)	-3.4 (0.918)
		Composite	3.57 (<0.001)	-.265 (0.350)	.00131 (0.675)
**Anthropology (1.6%)**	**Other fields**	Raw citations	2335 (0.011)	647 (0.694)	39.1 (0.058)
		Composite	3.49 (<0.001)	-.0296 (0.840)	.00399 (0.030)
**Materials (1.6%)**	**Other fields**	Raw citations	6837 (<0.001)	3504 (0.034)	-25.7 (0.058)
		Composite	3.28 (<0.001)	.245 (0.004)	.00239 (0.001)
**Fluids & Plasmas (1.6%)**	**Other fields**	Raw citations	13116 (<0.001)	6896 (0.170)	-74.3 (0.154)
		Composite	3.73 (<0.001)	.223 (0.058)	.00153 (0.208)
**General Mathematics (1.6%)**	**Other fields**	Raw citations	3092 (<0.001)	-777 (0.478)	-.816 (0.922)
		Composite	3.47 (<0.001)	-.149 (0.084)	.000699 (0.285)
**Building & Construction (1.6%)**	**Other fields**	Raw citations	2339 (0.014)	1702 (0.489)	51.2 (0.064)
		Composite	3.13 (<0.001)	.107 (0.606)	.00737 (0.002)
**Philosophy (1.5%)**	**Other fields**	Raw citations	1638 (<0.001)	3821 (<0.001)	1.48 (0.813)
		Composite	3.42 (<0.001)	.595 (<0.001)	.000139 (0.914)
**Meteorology & Atmospheric Sciences (1.5%)**	**Other fields**	Raw citations	10829 (<0.001)	-1970 (0.409)	17.3 (0.536)
		Composite	3.68 (<0.001)	-.0122 (0.856)	.00292 (<0.001)
**Aerospace & Aeronautics (1.5%)**	**Other fields**	Raw citations	2328 (<0.001)	625 (0.400)	7.86 (0.306)
		Composite	3.08 (<0.001)	-.0421 (0.659)	.00292 (0.003)
**Energy (1.4%)**	**Other fields**	Raw citations	4530 (<0.001)	2329 (0.065)	7.86 (0.468)
		Composite	3.2 (<0.001)	.0433 (0.588)	.00374 (<0.001)
**Geological & Geomatics Engineering (1.4%)**	**Other fields**	Raw citations	5922 (<0.001)	5192 (0.033)	8.83 (0.744)
		Composite	3.31 (<0.001)	.214 (0.112)	.00526 (0.001)
**Ecology (1.3%)**	**Other fields**	Raw citations	6424 (<0.001)	914 (0.777)	116 (0.003)
		Composite	3.55 (<0.001)	.176 (0.048)	.00727 (<0.001)

© 2022 Ioannidis JPA et al. *JAMA Network Open.*

**Oceanography (1.1%)**	**Other fields**	Raw citations	5634 (<0.001)	4612 (0.121)	3 (0.918)
		Composite	3.39 (<0.001)	.163 (0.306)	.00534 (0.001)
**Business & Management (.95%)**	**Other fields**	Raw citations	5565 (<0.001)	216 (0.943)	67.6 (0.011)
		Composite	3.63 (<0.001)	.0771 (0.511)	.0036 (0.001)
**Law (.94%)**	**Other fields**	Raw citations	737 (0.447)	-826 (0.715)	32.7 (0.312)
		Composite	3.11 (<0.001)	-.0805 (0.719)	.00612 (0.057)
**Applied Physics (.94%)**	**Other fields**	Raw citations	10460 (<0.001)	1586 (0.582)	-14.2 (0.436)
		Composite	3.53 (<0.001)	.124 (0.118)	.00196 (<0.001)
**Marine Biology & Hydrobiology (.91%)**	**Other fields**	Raw citations	7907 (<0.001)	4376 (0.190)	-3.06 (0.927)
		Composite	3.66 (<0.001)	.246 (0.073)	.00225 (0.104)
**Physical Chemistry (.88%)**	**Other fields**	Raw citations	11640 (<0.001)	-5354 (0.568)	-38.2 (0.499)
		Composite	3.49 (<0.001)	-.247 (0.517)	.00373 (0.107)
**Nuclear & Particle Physics (.78%)**	**Other fields**	Raw citations	18451 (<0.001)	-417 (0.949)	-99.6 (0.003)
		Composite	3.64 (<0.001)	.038 (0.735)	.00122 (0.034)
**Astronomy & Astrophysics (.76%)**	**Other fields**	Raw citations	18707 (<0.001)	-5138 (0.391)	-87.1 (0.060)
		Composite	3.75 (<0.001)	.149 (0.121)	.00118 (0.110)
**Civil Engineering (.73%)**	**Other fields**	Raw citations	1891 (0.014)	336 (0.898)	36 (0.059)
		Composite	3.08 (<0.001)	.151 (0.519)	.00471 (0.006)
**Geochemistry & Geophysics (.71%)**	**Other fields**	Raw citations	7325 (<0.001)	-1900 (0.374)	15.2 (0.350)
		Composite	3.66 (<0.001)	-.176 (0.081)	.00308 (<0.001)
**Agronomy & Agriculture (.66%)**	**Other fields**	Raw citations	5473 (<0.001)	3108 (0.282)	-.802 (0.966)
		Composite	3.36 (<0.001)	.218 (0.181)	.00275 (0.010)
**Inorganic & Nuclear Chemistry (.63%)**	**Other fields**	Raw citations	5850 (0.003)	-3669 (0.595)	65.5 (0.102)
		Composite	3.38 (<0.001)	-.147 (0.531)	.00501 (<0.001)
**Forestry (.48%)**	**Other fields**	Raw citations	2816 (<0.001)	-2168 (0.376)	16 (0.395)
		Composite	3.15 (<0.001)	-.171 (0.510)	.00368 (0.065)

© 2022 Ioannidis JPA et al. *JAMA Network Open.*

**Zoology (0%)**	**Other fields**	Raw citations			
		Composite			
**Paleontology (0%)**	**Other fields**	Raw citations			
		Composite			
**Music (0%)**	**Other fields**	Raw citations			
		Composite			
**Mathematical Physics (0%)**	**Other fields**	Raw citations			
		Composite			
**Legal & Forensic Medicine (0%)**	**Highly related fields**	Raw citations			
		Composite			
**International Relations (0%)**	**Other fields**	Raw citations			
		Composite			
**Industrial Relations (0%)**	**Other fields**	Raw citations			
		Composite			
**Horticulture (0%)**	**Other fields**	Raw citations			
		Composite			
**History of Social Sciences (0%)**	**Other fields**	Raw citations			
		Composite			
**History of Science, Technology & Medicine (0%)**	**Other fields**	Raw citations			
		Composite			
**History (0%)**	**Other fields**	Raw citations			
		Composite			
**Geology (0%)**	**Other fields**	Raw citations			
		Composite			
**General Psychology & Cognitive Sciences (0%)**	**Highly related fields**	Raw citations			
		Composite			
**Gender Studies (0%)**	**Highly related fields**	Raw citations			
		Composite			
**Folklore (0%)**	**Other fields**	Raw citations			
		Composite			
**Classics (0%)**	**Other fields**	Raw citations			
		Composite			
**Automobile Design & Engineering (0%)**	**Other fields**	Raw citations			
		Composite			

© 2022 Ioannidis JPA et al. *JAMA Network Open.*

**Art Practice, History & Theory (0%)**	**Other fields**	Raw citations			
		Composite			
**Architecture (0%)**	**Other fields**	Raw citations			
		Composite			
**Accounting (0%)**	**Other fields**	Raw citations			
		Composite			

eTable 3.4 : Recent year impact, Funding time any funding Linear Regressions for each subfield (ordered by percentage funded)

Top-cited US-based researchers: Subfield (perc. funded)	Classification	Dependent Variable	Constant (p-val)	Funded (p-val)	Years since first pub (p-val)
**Geriatrics (88%)**	**Highly related fields**	Raw citations	768 (0.390)	1029 (0.122)	9.23 (0.632)
		Composite	2.84 (<0.001)	.25 (0.038)	.00409 (0.240)
**Gerontology (87%)**	**Highly related fields**	Raw citations	392 (0.752)	1370 (0.138)	11.2 (0.680)
		Composite	2.87 (<0.001)	.174 (0.048)	.005 (0.053)
**Substance Abuse (86%)**	**Highly related fields**	Raw citations	719 (0.003)	351 (0.062)	10.1 (0.076)
		Composite	2.98 (<0.001)	.061 (0.251)	.00411 (0.011)
**Developmental Biology (86%)**	**Highly related fields**	Raw citations	4258 (<0.001)	-45.7 (0.886)	-29.7 (0.002)
		Composite	2.99 (<0.001)	.0564 (0.017)	.00616 (<0.001)
**Endocrinology & Metabolism (83%)**	**Highly related fields**	Raw citations	1543 (<0.001)	363 (0.068)	6.25 (0.297)
		Composite	2.98 (<0.001)	.0971 (0.003)	.00481 (<0.001)
**Immunology (83%)**	**Highly related fields**	Raw citations	2095 (<0.001)	10.3 (0.952)	13.7 (0.011)
		Composite	2.96 (<0.001)	.0632 (0.014)	.0053 (<0.001)
**Neurology & Neurosurgery (82%)**	**Highly related fields**	Raw citations	1765 (<0.001)	478 (<0.001)	1.27 (0.709)
		Composite	3 (<0.001)	.0889 (<0.001)	.00399 (<0.001)
**Biochemistry & Molecular Biology (81%)**	**Highly related fields**	Raw citations	1317 (<0.001)	346 (<0.001)	-5.45 (0.032)
		Composite	2.76 (<0.001)	.123 (<0.001)	.00408 (<0.001)
**Virology (81%)**	**Highly related fields**	Raw citations	1723 (<0.001)	120 (0.501)	-1.37 (0.809)
		Composite	2.75 (<0.001)	.0491 (0.094)	.00491 (<0.001)
**Psychiatry (81%)**	**Highly related fields**	Raw citations	1030 (0.002)	785 (<0.001)	19 (0.009)
		Composite	3 (<0.001)	.0841 (0.008)	.00582 (<0.001)
**Genetics & Heredity (79%)**	**Highly related fields**	Raw citations	2348 (<0.001)	414 (0.115)	-16.2 (0.079)
		Composite	2.82 (<0.001)	.0564 (0.107)	.0018 (0.144)

© 2022 Ioannidis JPA et al. *JAMA Network Open.*

**Allergy (75%)**	**Highly related fields**	Raw citations	740 (0.052)	606 (0.015)	19.5 (0.030)
		Composite	2.87 (<0.001)	.145 (0.019)	.00609 (0.007)
**Biophysics (74%)**	**Highly related fields**	Raw citations	689 (0.057)	521 (0.040)	.219 (0.978)
		Composite	2.5 (<0.001)	.198 (<0.001)	.00546 (<0.001)
**Epidemiology (74%)**	**Highly related fields**	Raw citations	1537 (0.105)	217 (0.733)	28.8 (0.207)
		Composite	3.15 (<0.001)	-.0773 (0.286)	.0041 (0.115)
**Biomedical Engineering (74%)**	**Highly related fields**	Raw citations	501 (0.031)	658 (<0.001)	11 (0.078)
		Composite	2.64 (<0.001)	.121 (<0.001)	.00673 (<0.001)
**Arthritis & Rheumatology (73%)**	**Highly related fields**	Raw citations	1440 (0.007)	869 (0.010)	10.2 (0.409)
		Composite	2.98 (<0.001)	.121 (0.021)	.00408 (0.033)
**Oncology & Carcinogenesis (72%)**	**Highly related fields**	Raw citations	3185 (<0.001)	410 (0.014)	-10.9 (0.088)
		Composite	2.91 (<0.001)	.0791 (<0.001)	.00381 (<0.001)
**Developmental & Child Psychology (72%)**	**Highly related fields**	Raw citations	466 (0.005)	572 (<0.001)	14.1 (<0.001)
		Composite	3.02 (<0.001)	.0938 (0.005)	.0053 (<0.001)
**Medical Informatics (71%)**	**Highly related fields**	Raw citations	750 (0.003)	243 (0.217)	.0172 (0.998)
		Composite	2.71 (<0.001)	-.000266 (0.997)	.00417 (0.084)
**Physiology (71%)**	**Highly related fields**	Raw citations	474 (<0.001)	253 (0.001)	4.07 (0.080)
		Composite	2.83 (<0.001)	.0537 (0.171)	.00379 (0.003)
**Public Health (71%)**	**Highly related fields**	Raw citations	962 (0.003)	-94.5 (0.672)	27.4 (0.002)
		Composite	3 (<0.001)	.0397 (0.117)	.00487 (<0.001)
**Gastroenterology & Hepatology (69%)**	**Highly related fields**	Raw citations	1618 (<0.001)	405 (0.012)	.268 (0.965)
		Composite	2.9 (<0.001)	.0949 (0.003)	.00355 (0.004)
**Urology & Nephrology (67%)**	**Highly related fields**	Raw citations	1708 (<0.001)	370 (0.009)	-7.9 (0.150)
		Composite	2.79 (<0.001)	.129 (<0.001)	.0028 (0.004)
**Respiratory System (67%)**	**Highly related fields**	Raw citations	1285 (<0.001)	455 (0.001)	10.8 (0.059)
		Composite	2.82 (<0.001)	.0622 (0.015)	.00506 (<0.001)

© 2022 Ioannidis JPA et al. *JAMA Network Open.*

**Pediatrics (67%)**	**Highly related fields**	Raw citations	699 (<0.001)	257 (<0.001)	2.61 (0.233)
		Composite	2.58 (<0.001)	.0925 (<0.001)	.00346 (<0.001)
**Bioinformatics (67%)**	**Highly related fields**	Raw citations	3520 (<0.001)	765 (0.250)	-19.3 (0.496)
		Composite	3 (<0.001)	.0686 (0.252)	.00289 (0.259)
**Cardiovascular System & Hematology (66%)**	**Highly related fields**	Raw citations	3008 (<0.001)	267 (0.098)	-7.59 (0.199)
		Composite	2.95 (<0.001)	.0794 (<0.001)	.00337 (<0.001)
**Health Policy & Services (64%)**	**Highly related fields**	Raw citations	685 (0.019)	223 (0.223)	17.2 (0.028)
		Composite	2.8 (<0.001)	.0999 (0.026)	.00665 (0.001)
**Experimental Psychology (64%)**	**Highly related fields**	Raw citations	909 (<0.001)	302 (0.001)	2.58 (0.382)
		Composite	3.1 (<0.001)	.0719 (0.012)	.00368 (<0.001)
**Emergency & Critical Care Medicine (63%)**	**Highly related fields**	Raw citations	714 (0.087)	1077 (<0.001)	16.9 (0.157)
		Composite	2.75 (<0.001)	.188 (<0.001)	.00366 (0.048)
**Clinical Psychology (63%)**	**Highly related fields**	Raw citations	541 (0.045)	723 (<0.001)	16.9 (0.006)
		Composite	3.03 (<0.001)	.0951 (0.056)	.00482 (0.005)
**Ophthalmology & Optometry (62%)**	**Highly related fields**	Raw citations	1209 (<0.001)	359 (<0.001)	-5.75 (0.137)
		Composite	2.7 (<0.001)	.132 (<0.001)	.00307 (0.001)
**Family Studies (62%)**	**Other fields**	Raw citations	215 (0.126)	214 (0.022)	6.25 (0.078)
		Composite	2.85 (<0.001)	.0286 (0.626)	.00579 (0.013)
**Speech-Language Pathology & Audiology (61%)**	**Highly related fields**	Raw citations	1132 (<0.001)	-84.7 (0.615)	-9.25 (0.198)
		Composite	2.81 (<0.001)	-.000538 (0.991)	.00419 (0.044)
**Rehabilitation (61%)**	**Highly related fields**	Raw citations	439 (0.003)	159 (0.071)	10 (0.013)
		Composite	2.7 (<0.001)	-.0105 (0.764)	.00662 (<0.001)
**Obstetrics & Reproductive Medicine (60%)**	**Highly related fields**	Raw citations	817 (<0.001)	412 (<0.001)	.233 (0.944)
		Composite	2.72 (<0.001)	.118 (<0.001)	.00254 (0.011)
**Toxicology (60%)**	**Highly related fields**	Raw citations	1148 (<0.001)	536 (0.001)	-5.14 (0.418)
		Composite	2.67 (<0.001)	.133 (<0.001)	.00463 (<0.001)

© 2022 Ioannidis JPA et al. *JAMA Network Open.*

**Environmental & Occupational Health (59%)**	**Highly related fields**	Raw citations	469 (0.208)	-167 (0.446)	12.7 (0.216)
		Composite	2.53 (<0.001)	.0602 (0.230)	.00361 (0.125)
**Nutrition & Dietetics (59%)**	**Highly related fields**	Raw citations	1066 (<0.001)	350 (0.015)	4.67 (0.393)
		Composite	2.95 (<0.001)	.0863 (0.016)	.00441 (0.001)
**Microbiology (59%)**	**Highly related fields**	Raw citations	2613 (<0.001)	161 (0.228)	-24.5 (<0.001)
		Composite	2.92 (<0.001)	.0543 (0.002)	.00369 (<0.001)
**Nursing (59%)**	**Highly related fields**	Raw citations	129 (0.029)	179 (<0.001)	7 (<0.001)
		Composite	2.41 (<0.001)	.0303 (0.183)	.00467 (<0.001)
**Demography (58%)**	**Highly related fields**	Raw citations	58.1 (0.858)	192 (0.369)	13.6 (0.094)
		Composite	2.8 (<0.001)	.0392 (0.691)	.00702 (0.065)
**Analytical Chemistry (58%)**	**Other fields**	Raw citations	810 (<0.001)	606 (<0.001)	-1.48 (0.732)
		Composite	2.71 (<0.001)	.0844 (0.011)	.00318 (0.006)
**Nuclear Medicine & Medical Imaging (57%)**	**Highly related fields**	Raw citations	1004 (<0.001)	324 (<0.001)	-1.93 (0.465)
		Composite	2.66 (<0.001)	.121 (<0.001)	.00171 (0.018)
**Organic Chemistry (56%)**	**Other fields**	Raw citations	1316 (<0.001)	179 (0.095)	-2.69 (0.415)
		Composite	2.82 (<0.001)	.112 (<0.001)	.00348 (<0.001)
**Pharmacology & Pharmacy (53%)**	**Highly related fields**	Raw citations	623 (<0.001)	339 (<0.001)	1.09 (0.641)
		Composite	2.58 (<0.001)	.0988 (<0.001)	.00414 (<0.001)
**Medicinal & Biomolecular Chemistry (51%)**	**Highly related fields**	Raw citations	517 (<0.001)	337 (0.001)	8.9 (0.019)
		Composite	2.44 (<0.001)	.12 (<0.001)	.00677 (<0.001)
**Complementary & Alternative Medicine (50%)**	**Highly related fields**	Raw citations	242 (0.044)	257 (0.001)	1.81 (0.663)
		Composite	2.52 (<0.001)	.0584 (0.483)	.00107 (0.813)
**Behavioral Science & Comparative Psychology (49%)**	**Highly related fields**	Raw citations	692 (<0.001)	236 (0.012)	.129 (0.970)
		Composite	2.93 (<0.001)	.0766 (0.085)	.0045 (0.007)
**Anesthesiology (48%)**	**Highly related fields**	Raw citations	885 (<0.001)	256 (0.023)	-1.98 (0.684)
		Composite	2.6 (<0.001)	.135 (<0.001)	.00423 (0.002)

© 2022 Ioannidis JPA et al. *JAMA Network Open.*

**Dentistry (48%)**	**Highly related fields**	Raw citations	787 (<0.001)	263 (<0.001)	-4.62 (0.061)
		Composite	2.69 (<0.001)	.0963 (0.001)	.00173 (0.107)
**Otorhinolaryngology (47%)**	**Highly related fields**	Raw citations	893 (<0.001)	27.6 (0.734)	-5.1 (0.105)
		Composite	2.6 (<0.001)	.0578 (0.011)	.00161 (0.066)
**Tropical Medicine (46%)**	**Highly related fields**	Raw citations	1264 (0.001)	460 (0.084)	-8.21 (0.381)
		Composite	2.56 (<0.001)	.106 (0.004)	.00463 (<0.001)
**Applied Ethics (46%)**	**Highly related fields**	Raw citations	804 (<0.001)	264 (0.051)	-5.64 (0.300)
		Composite	3.02 (<0.001)	.058 (0.410)	.000272 (0.924)
**General & Internal Medicine (46%)**	**Highly related fields**	Raw citations	3316 (<0.001)	-197 (0.465)	-35.2 (<0.001)
		Composite	2.55 (<0.001)	.209 (<0.001)	.00181 (0.007)
**Statistics & Probability (45%)**	**Other fields**	Raw citations	942 (0.026)	606 (0.039)	12.8 (0.170)
		Composite	3.07 (<0.001)	.0876 (0.068)	.00331 (0.030)
**Surgery (44%)**	**Highly related fields**	Raw citations	943 (<0.001)	395 (<0.001)	2.83 (0.355)
		Composite	2.6 (<0.001)	.104 (<0.001)	.0032 (<0.001)
**Mycology & Parasitology (44%)**	**Highly related fields**	Raw citations	1424 (<0.001)	-270 (0.104)	-8.83 (0.129)
		Composite	2.82 (<0.001)	-.0937 (0.121)	.00172 (0.414)
**Dermatology & Venereal Diseases (42%)**	**Highly related fields**	Raw citations	1145 (<0.001)	683 (<0.001)	-4.48 (0.478)
		Composite	2.73 (<0.001)	.161 (<0.001)	.00273 (0.046)
**History of Social Sciences (41%)**	**Other fields**	Raw citations	-31.4 (0.881)	-172 (0.189)	10.1 (0.041)
		Composite	2.29 (<0.001)	-.0882 (0.519)	.0138 (0.012)
**Biotechnology (41%)**	**Highly related fields**	Raw citations	692 (0.004)	164 (0.359)	20 (0.008)
		Composite	2.64 (<0.001)	.052 (0.260)	.0104 (<0.001)
**General Chemistry (40%)**	**Other fields**	Raw citations	1304 (<0.001)	385 (0.111)	-.0811 (0.986)
		Composite	2.59 (<0.001)	.171 (<0.001)	.00529 (<0.001)
**Sport Sciences (38%)**	**Highly related fields**	Raw citations	784 (0.013)	506 (0.016)	10.4 (0.265)
		Composite	3.02 (<0.001)	.0572 (0.226)	.00204 (0.335)

© 2022 Ioannidis JPA et al. *JAMA Network Open.*

**Social Psychology (38%)**	**Highly related fields**	Raw citations	961 (<0.001)	706 (<0.001)	9.51 (0.047)
		Composite	3.14 (<0.001)	.178 (<0.001)	.00434 (<0.001)
**Acoustics (37%)**	**Other fields**	Raw citations	963 (<0.001)	-5.23 (0.954)	-7.81 (0.005)
		Composite	2.84 (<0.001)	-.0267 (0.508)	.000611 (0.621)
**Optics (32%)**	**Other fields**	Raw citations	1066 (<0.001)	75.7 (0.669)	13.1 (0.052)
		Composite	2.67 (<0.001)	.0451 (0.213)	.00779 (<0.001)
**Orthopedics (31%)**	**Highly related fields**	Raw citations	1110 (<0.001)	365 (<0.001)	-2.37 (0.388)
		Composite	2.79 (<0.001)	.116 (<0.001)	.00336 (<0.001)
**Chemical Physics (30%)**	**Other fields**	Raw citations	1439 (<0.001)	795 (<0.001)	6.04 (0.223)
		Composite	3 (<0.001)	.11 (<0.001)	.00415 (<0.001)
**Evolutionary Biology (30%)**	**Other fields**	Raw citations	960 (0.001)	769 (<0.001)	6.19 (0.419)
		Composite	2.98 (<0.001)	.0813 (0.016)	.00686 (<0.001)
**Nanoscience & Nanotechnology (29%)**	**Other fields**	Raw citations	1728 (<0.001)	-28.7 (0.940)	98.9 (<0.001)
		Composite	2.98 (<0.001)	.071 (0.030)	.0127 (<0.001)
**Sociology (28%)**	**Other fields**	Raw citations	384 (<0.001)	40.1 (0.513)	6.23 (0.002)
		Composite	3.06 (<0.001)	-.0314 (0.427)	.00385 (0.003)
**Pathology (28%)**	**Highly related fields**	Raw citations	1763 (<0.001)	404 (0.056)	-7.32 (0.342)
		Composite	2.83 (<0.001)	.0621 (0.142)	.00202 (0.192)
**Plant Biology & Botany (27%)**	**Other fields**	Raw citations	1194 (<0.001)	418 (<0.001)	-3.61 (0.258)
		Composite	2.87 (<0.001)	.0835 (0.001)	.0041 (<0.001)
**General Clinical Medicine (27%)**	**Highly related fields**	Raw citations	289 (0.034)	465 (<0.001)	1.84 (0.611)
		Composite	2.2 (<0.001)	.175 (0.015)	.00594 (0.006)
**Veterinary Sciences (27%)**	**Highly related fields**	Raw citations	456 (<0.001)	125 (0.001)	.87 (0.563)
		Composite	2.5 (<0.001)	.0669 (0.005)	.0029 (0.002)
**Environmental Sciences (26%)**	**Other fields**	Raw citations	1664 (<0.001)	665 (0.023)	-1.15 (0.909)
		Composite	3.07 (<0.001)	.101 (0.037)	.00295 (0.080)

© 2022 Ioannidis JPA et al. *JAMA Network Open.*

**Social Sciences Methods (25%)**	**Other fields**	Raw citations	487 (0.391)	1982 (<0.001)	11.3 (0.357)
		Composite	3.1 (<0.001)	.322 (0.001)	.00332 (0.113)
**Criminology (25%)**	**Other fields**	Raw citations	379 (<0.001)	311 (<0.001)	8.31 (0.008)
		Composite	2.98 (<0.001)	.0943 (0.033)	.00485 (0.002)
**Microscopy (24%)**	**Highly related fields**	Raw citations	656 (0.236)	235 (0.577)	9.4 (0.521)
		Composite	2.42 (<0.001)	.102 (0.497)	.0104 (0.053)
**Inorganic & Nuclear Chemistry (23%)**	**Other fields**	Raw citations	934 (<0.001)	32.1 (0.892)	4.53 (0.433)
		Composite	2.75 (<0.001)	.111 (0.048)	.00287 (0.036)
**Social Work (23%)**	**Other fields**	Raw citations	190 (0.035)	321 (<0.001)	5.37 (0.092)
		Composite	2.75 (<0.001)	.146 (0.006)	.00126 (0.522)
**Polymers (21%)**	**Other fields**	Raw citations	1200 (<0.001)	543 (0.001)	-1.22 (0.760)
		Composite	2.86 (<0.001)	.074 (0.081)	.00315 (0.004)
**Drama & Theater (20%)**	**Other fields**	Raw citations	89.6 (0.160)	-18 (0.687)	-.776 (0.706)
		Composite	1.99 (<0.001)	.0288 (0.831)	.00431 (0.495)
**Economics (19%)**	**Other fields**	Raw citations	739 (<0.001)	216 (0.033)	4.18 (0.143)
		Composite	3.08 (<0.001)	.0832 (0.028)	.00502 (<0.001)
**Food Science (19%)**	**Other fields**	Raw citations	1061 (0.003)	184 (0.578)	3.59 (0.701)
		Composite	2.86 (<0.001)	.0976 (0.124)	.0054 (0.003)
**Artificial Intelligence & Image Processing (19%)**	**Other fields**	Raw citations	1773 (<0.001)	132 (0.499)	-4.51 (0.470)
		Composite	2.78 (<0.001)	.0109 (0.697)	.00714 (<0.001)
**Distributed Computing (18%)**	**Other fields**	Raw citations	363 (0.266)	663 (0.016)	9.86 (0.374)
		Composite	2.37 (<0.001)	.052 (0.519)	.0042 (0.204)
**Industrial Engineering & Automation (17%)**	**Other fields**	Raw citations	1046 (<0.001)	272 (0.022)	-2.38 (0.489)
		Composite	2.87 (<0.001)	.0638 (0.133)	.00302 (0.015)
**Entomology (17%)**	**Other fields**	Raw citations	962 (<0.001)	392 (<0.001)	-8.84 (<0.001)
		Composite	2.91 (<0.001)	.218 (<0.001)	-.00174 (0.207)

© 2022 Ioannidis JPA et al. *JAMA Network Open.*

**Design Practice & Management (16%)**	**Other fields**	Raw citations	638 (<0.001)	259 (0.137)	-.528 (0.899)
		Composite	2.79 (<0.001)	.0166 (0.876)	.00122 (0.635)
**Human Factors (16%)**	**Highly related fields**	Raw citations	745 (<0.001)	73.4 (0.647)	6.59 (0.204)
		Composite	2.88 (<0.001)	.0226 (0.702)	.00743 (<0.001)
**Information & Library Sciences (16%)**	**Other fields**	Raw citations	216 (0.004)	253 (0.006)	2.05 (0.373)
		Composite	2.6 (<0.001)	.185 (0.026)	.00199 (0.345)
**Fluids & Plasmas (16%)**	**Other fields**	Raw citations	1646 (<0.001)	89.2 (0.718)	-10.5 (0.098)
		Composite	3.12 (<0.001)	.0232 (0.662)	.001 (0.460)
**General Psychology & Cognitive Sciences (16%)**	**Highly related fields**	Raw citations	660 (<0.001)	87.6 (0.610)	.427 (0.927)
		Composite	3 (<0.001)	-.0246 (0.813)	.000894 (0.752)
**Urban & Regional Planning (15%)**	**Other fields**	Raw citations	549 (0.001)	82.1 (0.655)	1.56 (0.748)
		Composite	3.12 (<0.001)	.121 (0.220)	.00247 (0.344)
**Education (15%)**	**Other fields**	Raw citations	340 (<0.001)	216 (<0.001)	6.95 (<0.001)
		Composite	2.89 (<0.001)	.0149 (0.615)	.00417 (<0.001)
**Psychoanalysis (15%)**	**Highly related fields**	Raw citations	82.3 (0.574)	12.2 (0.926)	2.19 (0.413)
		Composite	2.56 (<0.001)	-.0927 (0.607)	.000313 (0.931)
**Development Studies (15%)**	**Other fields**	Raw citations	550 (0.024)	129 (0.515)	2.3 (0.719)
		Composite	3.14 (<0.001)	-.0142 (0.920)	.0018 (0.696)
**Anatomy & Morphology (14%)**	**Highly related fields**	Raw citations	330 (<0.001)	89.4 (0.379)	-1.81 (0.380)
		Composite	2.46 (<0.001)	-.0209 (0.841)	-.000409 (0.847)
**Geography (14%)**	**Other fields**	Raw citations	300 (0.014)	239 (0.066)	7.74 (0.030)
		Composite	2.98 (<0.001)	.185 (0.013)	.00461 (0.023)
**Ornithology (14%)**	**Other fields**	Raw citations	403 (0.003)	-2.79 (0.985)	.55 (0.850)
		Composite	2.57 (<0.001)	.075 (0.480)	.00349 (0.096)
**History of Science, Technology & Medicine (13%)**	**Other fields**	Raw citations	48.4 (0.342)	-3.1 (0.954)	1.18 (0.262)
		Composite	2.29 (<0.001)	-.0496 (0.811)	.00217 (0.587)

© 2022 Ioannidis JPA et al. *JAMA Network Open.*

**Gender Studies (13%)**	**Highly related fields**	Raw citations	417 (0.064)	-153 (0.468)	2.06 (0.723)
		Composite	2.9 (<0.001)	-.0419 (0.769)	.00499 (0.225)
**Dairy & Animal Science (13%)**	**Other fields**	Raw citations	738 (<0.001)	136 (0.066)	-3.79 (0.042)
		Composite	2.63 (<0.001)	.0375 (0.430)	.00236 (0.050)
**Logistics & Transportation (13%)**	**Other fields**	Raw citations	698 (<0.001)	-93.9 (0.615)	6.69 (0.202)
		Composite	2.97 (<0.001)	-.121 (0.142)	.00342 (0.140)
**Economic Theory (13%)**	**Other fields**	Raw citations	220 (0.217)	-137 (0.369)	5.24 (0.228)
		Composite	2.77 (<0.001)	.0322 (0.815)	.00461 (0.246)
**Optoelectronics & Photonics (12%)**	**Other fields**	Raw citations	487 (<0.001)	85.4 (0.264)	1.57 (0.395)
		Composite	2.32 (<0.001)	.0124 (0.746)	.0057 (<0.001)
**Software Engineering (12%)**	**Other fields**	Raw citations	1091 (<0.001)	42.9 (0.695)	-13.3 (<0.001)
		Composite	2.79 (<0.001)	-.0816 (0.153)	.00156 (0.380)
**Marine Biology & Hydrobiology (12%)**	**Other fields**	Raw citations	1526 (<0.001)	54.8 (0.739)	-8.98 (0.088)
		Composite	3.06 (<0.001)	-.0403 (0.429)	.00224 (0.168)
**Anthropology (12%)**	**Other fields**	Raw citations	297 (<0.001)	33 (0.660)	4.7 (0.023)
		Composite	2.97 (<0.001)	-.0705 (0.293)	.00293 (0.109)
**Numerical & Computational Mathematics (12%)**	**Other fields**	Raw citations	525 (0.008)	421 (0.029)	5.09 (0.281)
		Composite	3.1 (<0.001)	.0294 (0.752)	-.0000629 (0.978)
**Geological & Geomatics Engineering (11%)**	**Other fields**	Raw citations	1364 (<0.001)	185 (0.368)	-2.54 (0.640)
		Composite	2.97 (<0.001)	.072 (0.233)	.0049 (0.002)
**Electrical & Electronic Engineering (11%)**	**Other fields**	Raw citations	399 (<0.001)	223 (0.113)	10.2 (0.003)
		Composite	2.45 (<0.001)	.0931 (0.125)	.00632 (<0.001)
**Physical Chemistry (10%)**	**Other fields**	Raw citations	1703 (<0.001)	1296 (0.007)	2.67 (0.754)
		Composite	2.91 (<0.001)	.0543 (0.641)	.00678 (0.002)
**Econometrics (10%)**	**Other fields**	Raw citations	582 (0.488)	111 (0.889)	27.4 (0.217)
		Composite	3.28 (<0.001)	-.171 (0.434)	.00635 (0.298)

© 2022 Ioannidis JPA et al. *JAMA Network Open.*

**Computer Hardware & Architecture (10%)**	**Other fields**	Raw citations	594 (<0.001)	138 (0.248)	2.65 (0.382)
		Composite	2.46 (<0.001)	.0834 (0.168)	.00469 (0.003)
**Communication & Media Studies (10%)**	**Other fields**	Raw citations	113 (0.419)	-127 (0.548)	20.9 (<0.001)
		Composite	3.04 (<0.001)	-.0157 (0.812)	.00428 (0.006)
**Environmental Engineering (10%)**	**Other fields**	Raw citations	1011 (<0.001)	825 (<0.001)	-1.98 (0.631)
		Composite	2.95 (<0.001)	.148 (0.006)	.00205 (0.109)
**Networking & Telecommunications (10%)**	**Other fields**	Raw citations	882 (<0.001)	-92.8 (0.367)	-1.8 (0.371)
		Composite	2.62 (<0.001)	-.0311 (0.404)	.00377 (<0.001)
**Archaeology (9.9%)**	**Other fields**	Raw citations	423 (<0.001)	-37.7 (0.788)	.935 (0.741)
		Composite	2.82 (<0.001)	-.0845 (0.326)	.0015 (0.384)
**Zoology (9.8%)**	**Other fields**	Raw citations	444 (0.001)	-45 (0.729)	.508 (0.891)
		Composite	2.65 (<0.001)	-.0371 (0.664)	-.00103 (0.671)
**Religions & Theology (9.8%)**	**Other fields**	Raw citations	139 (0.002)	174 (0.013)	-.768 (0.593)
		Composite	2.33 (<0.001)	.177 (0.178)	.000108 (0.968)
**Chemical Engineering (9.6%)**	**Other fields**	Raw citations	1492 (<0.001)	508 (0.046)	-8.9 (0.054)
		Composite	2.98 (<0.001)	.12 (0.145)	.00205 (0.170)
**Ecology (9.4%)**	**Other fields**	Raw citations	1294 (<0.001)	14.6 (0.941)	11.6 (0.025)
		Composite	3.07 (<0.001)	.0896 (0.024)	.00531 (<0.001)
**Materials (9.3%)**	**Other fields**	Raw citations	1231 (<0.001)	148 (0.407)	-1.59 (0.606)
		Composite	2.76 (<0.001)	.0408 (0.331)	.00499 (<0.001)
**General Physics (9.1%)**	**Other fields**	Raw citations	1256 (<0.001)	259 (0.178)	-6.79 (0.031)
		Composite	2.78 (<0.001)	.0217 (0.721)	.00334 (0.001)
**Oceanography (8.9%)**	**Other fields**	Raw citations	1002 (<0.001)	141 (0.341)	-5.64 (0.120)
		Composite	2.91 (<0.001)	.0801 (0.269)	.0016 (0.366)
**Building & Construction (8.8%)**	**Other fields**	Raw citations	759 (0.001)	304 (0.295)	12.3 (0.074)
		Composite	2.93 (<0.001)	.0546 (0.601)	.00731 (0.004)

© 2022 Ioannidis JPA et al. *JAMA Network Open.*

**Sport, Leisure & Tourism (8.7%)**	**Other fields**	Raw citations	698 (<0.001)	-248 (0.172)	5.28 (0.303)
		Composite	3.08 (<0.001)	-.0635 (0.481)	.00458 (0.075)
**Applied Mathematics (8.7%)**	**Other fields**	Raw citations	953 (0.016)	1278 (0.009)	6.49 (0.488)
		Composite	3.05 (<0.001)	.262 (0.052)	.0046 (0.078)
**Mechanical Engineering & Transports (8.6%)**	**Other fields**	Raw citations	984 (<0.001)	394 (0.026)	-3.76 (0.259)
		Composite	2.83 (<0.001)	-.0361 (0.566)	.00344 (0.004)
**Strategic, Defence & Security Studies (8.5%)**	**Other fields**	Raw citations	240 (0.113)	137 (0.509)	10.7 (0.017)
		Composite	2.7 (<0.001)	.0471 (0.640)	.00767 (0.001)
**Languages & Linguistics (8.1%)**	**Other fields**	Raw citations	408 (<0.001)	163 (0.115)	.872 (0.713)
		Composite	3.02 (<0.001)	.222 (0.022)	.00292 (0.190)
**Computation Theory & Mathematics (8%)**	**Other fields**	Raw citations	695 (<0.001)	-7.46 (0.970)	-.302 (0.940)
		Composite	2.93 (<0.001)	.0198 (0.814)	.00231 (0.176)
**Marketing (7.9%)**	**Other fields**	Raw citations	191 (0.388)	50.4 (0.840)	27.5 (<0.001)
		Composite	3.01 (<0.001)	.108 (0.133)	.00564 (0.005)
**Law (7.6%)**	**Other fields**	Raw citations	72.5 (0.722)	-104 (0.609)	6.67 (0.369)
		Composite	2.45 (<0.001)	-.0012 (0.992)	.00448 (0.278)
**Meteorology & Atmospheric Sciences (7.4%)**	**Other fields**	Raw citations	1768 (<0.001)	108 (0.512)	1.25 (0.730)
		Composite	3.09 (<0.001)	.0183 (0.595)	.00334 (<0.001)
**Applied Physics (7.3%)**	**Other fields**	Raw citations	1446 (<0.001)	246 (0.160)	-2.46 (0.354)
		Composite	2.84 (<0.001)	.0402 (0.268)	.00261 (<0.001)
**Operations Research (7.2%)**	**Other fields**	Raw citations	553 (0.032)	-250 (0.446)	12.4 (0.062)
		Composite	3.1 (<0.001)	-.0278 (0.771)	.00308 (0.112)
**Finance (7.2%)**	**Other fields**	Raw citations	582 (0.003)	-373 (0.151)	14.3 (0.016)
		Composite	3.01 (<0.001)	-.195 (0.038)	.00732 (0.001)
**Political Science & Public Administration (7%)**	**Other fields**	Raw citations	465 (<0.001)	471 (0.002)	5.7 (0.059)
		Composite	3.05 (<0.001)	.11 (0.088)	.00392 (0.002)

© 2022 Ioannidis JPA et al. *JAMA Network Open.*

**Forestry (6.9%)**	**Other fields**	Raw citations	651 (<0.001)	65.8 (0.626)	-.962 (0.777)
		Composite	2.62 (<0.001)	.0768 (0.368)	.00343 (0.113)
**Business & Management (6.6%)**	**Other fields**	Raw citations	762 (<0.001)	-25.2 (0.885)	12.4 (0.001)
		Composite	3.11 (<0.001)	.00783 (0.872)	.00466 (<0.001)
**Literary Studies (6.5%)**	**Other fields**	Raw citations	33.6 (0.370)	-20.5 (0.688)	1.9 (0.156)
		Composite	1.94 (<0.001)	-.00612 (0.962)	.00787 (0.021)
**General Mathematics (6.1%)**	**Other fields**	Raw citations	442 (<0.001)	190 (0.019)	-.916 (0.398)
		Composite	2.9 (<0.001)	-.00709 (0.899)	.000617 (0.411)
**Aerospace & Aeronautics (6.1%)**	**Other fields**	Raw citations	398 (<0.001)	82.2 (0.179)	-.0663 (0.952)
		Composite	2.44 (<0.001)	-.0296 (0.581)	.00446 (<0.001)
**Geology (6.1%)**	**Other fields**	Raw citations	908 (<0.001)	-69.5 (0.730)	-6.24 (0.131)
		Composite	3.02 (<0.001)	-.0697 (0.599)	-.000583 (0.829)
**History (5.9%)**	**Other fields**	Raw citations	71.9 (0.028)	11.9 (0.837)	.286 (0.738)
		Composite	2.11 (<0.001)	.082 (0.433)	.00162 (0.300)
**Information Systems (5.9%)**	**Other fields**	Raw citations	1214 (<0.001)	-63.9 (0.882)	-1.4 (0.888)
		Composite	3.17 (<0.001)	-.0083 (0.942)	.000141 (0.957)
**Energy (5.9%)**	**Other fields**	Raw citations	1018 (<0.001)	138 (0.367)	.526 (0.830)
		Composite	2.85 (<0.001)	.0144 (0.758)	.00394 (<0.001)
**Science Studies (5.7%)**	**Other fields**	Raw citations	244 (0.310)	-101 (0.767)	12.1 (0.066)
		Composite	3.03 (<0.001)	-.185 (0.456)	.00637 (0.174)
**Legal & Forensic Medicine (5.7%)**	**Highly related fields**	Raw citations	272 (0.004)	-22.3 (0.883)	.685 (0.828)
		Composite	2.22 (<0.001)	.0124 (0.934)	.00552 (0.082)
**Cultural Studies (5.6%)**	**Other fields**	Raw citations	78.7 (0.289)	-88.9 (0.450)	2.51 (0.253)
		Composite	2.44 (<0.001)	-.193 (0.338)	.00556 (0.141)
**Astronomy & Astrophysics (5.5%)**	**Other fields**	Raw citations	3131 (<0.001)	-293 (0.433)	-23.5 (0.001)
		Composite	3.17 (<0.001)	-.00394 (0.929)	.000616 (0.441)

© 2022 Ioannidis JPA et al. *JAMA Network Open.*

**Accounting (5.5%)**	**Other fields**	Raw citations	427 (0.023)	176 (0.473)	13 (0.024)
		Composite	2.92 (<0.001)	.042 (0.766)	.00809 (0.016)
**Music (5.4%)**	**Other fields**	Raw citations	54.8 (0.008)	-18.7 (0.557)	.573 (0.492)
		Composite	2.17 (<0.001)	.0175 (0.906)	-.00137 (0.724)
**Geochemistry & Geophysics (5.4%)**	**Other fields**	Raw citations	1303 (<0.001)	-52 (0.678)	-4.8 (0.042)
		Composite	3.15 (<0.001)	-.0317 (0.500)	.00175 (0.049)
**Agronomy & Agriculture (5.3%)**	**Other fields**	Raw citations	1178 (<0.001)	106 (0.670)	-2.93 (0.475)
		Composite	2.88 (<0.001)	.0747 (0.325)	.00287 (0.022)
**Nuclear & Particle Physics (4.9%)**	**Other fields**	Raw citations	3823 (<0.001)	-419 (0.433)	-40.4 (<0.001)
		Composite	2.89 (<0.001)	.0439 (0.390)	.00253 (<0.001)
**Fisheries (4.8%)**	**Other fields**	Raw citations	710 (<0.001)	-75.4 (0.658)	1.11 (0.751)
		Composite	2.77 (<0.001)	-.0803 (0.408)	.00303 (0.131)
**Classics (4.8%)**	**Other fields**	Raw citations	40.1 (0.022)	17 (0.536)	.248 (0.593)
		Composite	2.04 (<0.001)	.216 (0.279)	.00159 (0.633)
**Mining & Metallurgy (4.7%)**	**Other fields**	Raw citations	223 (0.141)	-133 (0.605)	4.36 (0.253)
		Composite	2.3 (<0.001)	.0316 (0.895)	.006 (0.094)
**Paleontology (4.4%)**	**Other fields**	Raw citations	1174 (<0.001)	-209 (0.609)	-4.07 (0.558)
		Composite	3.13 (<0.001)	-.0634 (0.488)	.000257 (0.869)
**Agricultural Economics & Policy (4.3%)**	**Other fields**	Raw citations	976 (<0.001)	200 (0.586)	-2.55 (0.713)
		Composite	3.24 (<0.001)	-.00151 (0.993)	-.00163 (0.634)
**Philosophy (3.9%)**	**Other fields**	Raw citations	232 (<0.001)	177 (0.026)	.676 (0.568)
		Composite	2.89 (<0.001)	.288 (0.012)	.00113 (0.504)
**International Relations (3.6%)**	**Other fields**	Raw citations	118 (0.154)	-153 (0.350)	9.87 (<0.001)
		Composite	2.82 (<0.001)	-.276 (0.112)	.00882 (0.002)
**Civil Engineering (2.9%)**	**Other fields**	Raw citations	536 (<0.001)	-9.04 (0.975)	6.69 (0.071)
		Composite	2.77 (<0.001)	-.0232 (0.872)	.00494 (0.008)

© 2022 Ioannidis JPA et al. *JAMA Network Open.*

**Mathematical Physics (0%)**	**Other fields**	Raw citations			
		Composite			
**Industrial Relations (0%)**	**Other fields**	Raw citations			
		Composite			
**Horticulture (0%)**	**Other fields**	Raw citations			
		Composite			
**Folklore (0%)**	**Other fields**	Raw citations			
		Composite			
**Automobile Design & Engineering (0%)**	**Other fields**	Raw citations			
		Composite			
**Art Practice, History & Theory (0%)**	**Other fields**	Raw citations			
		Composite			
**Architecture (0%)**	**Other fields**	Raw citations			
		Composite			

© 2022 Ioannidis JPA et al. *JAMA Network Open.*

eTable 3.5 : Recent year impact, Funding time recent funding Linear Regressions for each subfield (ordered by percentage funded)

Top-cited US-based researchers: Subfield (perc. funded)	Classification	Dependent Variable	Constant (p-val)	Funded (p-val)	Years since first pub (p-val)
**Geriatrics (55%)**	**Highly related fields**	Raw citations	1138 (0.217)	421 (0.390)	18.2 (0.404)
		Composite	2.91 (<0.001)	.125 (0.160)	.00672 (0.091)
**Substance Abuse (50%)**	**Highly related fields**	Raw citations	758 (0.001)	270 (0.045)	14 (0.017)
		Composite	3.03 (<0.001)	-.00696 (0.855)	.00419 (0.012)
**Developmental Biology (49%)**	**Highly related fields**	Raw citations	4063 (<0.001)	213 (0.346)	-28.2 (0.004)
		Composite	3 (<0.001)	.0355 (0.034)	.00674 (<0.001)
**Medical Informatics (48%)**	**Highly related fields**	Raw citations	749 (0.003)	220 (0.226)	2.61 (0.728)
		Composite	2.69 (<0.001)	.0314 (0.594)	.00444 (0.071)
**Bioinformatics (46%)**	**Highly related fields**	Raw citations	3212 (0.001)	1178 (0.063)	-9.15 (0.748)
		Composite	2.99 (<0.001)	.0779 (0.173)	.00361 (0.163)
**Virology (45%)**	**Highly related fields**	Raw citations	1694 (<0.001)	160 (0.264)	.291 (0.960)
		Composite	2.76 (<0.001)	.0343 (0.146)	.00534 (<0.001)
**Immunology (45%)**	**Highly related fields**	Raw citations	2053 (<0.001)	69.6 (0.586)	14.3 (0.008)
		Composite	2.97 (<0.001)	.0445 (0.021)	.00604 (<0.001)
**Gerontology (44%)**	**Highly related fields**	Raw citations	153 (0.889)	1644 (0.012)	31.9 (0.250)
		Composite	2.96 (<0.001)	.0662 (0.295)	.00593 (0.030)
**Biomedical Engineering (44%)**	**Highly related fields**	Raw citations	579 (0.011)	531 (0.001)	17 (0.008)
		Composite	2.66 (<0.001)	.0817 (0.005)	.00769 (<0.001)
**Neurology & Neurosurgery (42%)**	**Highly related fields**	Raw citations	1995 (<0.001)	198 (0.027)	3.48 (0.325)
		Composite	3.06 (<0.001)	.0074 (0.581)	.00411 (<0.001)
**Oncology & Carcinogenesis (40%)**	**Highly related fields**	Raw citations	3170 (<0.001)	335 (0.030)	-5.15 (0.425)
		Composite	2.92 (<0.001)	.044 (0.001)	.00473 (<0.001)

© 2022 Ioannidis JPA et al. *JAMA Network Open.*

**Public Health (38%)**	**Highly related fields**	Raw citations	984 (0.002)	-124 (0.562)	26 (0.004)
		Composite	3.02 (<0.001)	.0108 (0.658)	.00504 (<0.001)
**Genetics & Heredity (38%)**	**Highly related fields**	Raw citations	2182 (<0.001)	636 (0.005)	-9.39 (0.318)
		Composite	2.81 (<0.001)	.076 (0.012)	.00263 (0.037)
**Emergency & Critical Care Medicine (38%)**	**Highly related fields**	Raw citations	721 (0.088)	1059 (<0.001)	26.2 (0.035)
		Composite	2.78 (<0.001)	.147 (0.001)	.00482 (0.014)
**Epidemiology (36%)**	**Highly related fields**	Raw citations	1546 (0.090)	237 (0.686)	30.6 (0.184)
		Composite	3.09 (<0.001)	.00261 (0.969)	.00395 (0.134)
**Psychiatry (36%)**	**Highly related fields**	Raw citations	1237 (<0.001)	610 (0.002)	24.9 (0.001)
		Composite	3.03 (<0.001)	.0527 (0.054)	.0063 (<0.001)
**Gastroenterology & Hepatology (35%)**	**Highly related fields**	Raw citations	1700 (<0.001)	259 (0.104)	3.67 (0.560)
		Composite	2.91 (<0.001)	.0798 (0.012)	.00449 (<0.001)
**Analytical Chemistry (35%)**	**Other fields**	Raw citations	901 (<0.001)	476 (0.001)	1.37 (0.765)
		Composite	2.72 (<0.001)	.0665 (0.064)	.00358 (0.003)
**Arthritis & Rheumatology (34%)**	**Highly related fields**	Raw citations	2017 (<0.001)	176 (0.610)	10.2 (0.451)
		Composite	3.06 (<0.001)	.0323 (0.541)	.0042 (0.044)
**Allergy (34%)**	**Highly related fields**	Raw citations	803 (0.028)	600 (0.013)	25.2 (0.008)
		Composite	2.93 (<0.001)	.0852 (0.163)	.00665 (0.006)
**Developmental & Child Psychology (33%)**	**Highly related fields**	Raw citations	561 (<0.001)	546 (<0.001)	18 (<0.001)
		Composite	3.04 (<0.001)	.0724 (0.025)	.00582 (<0.001)
**Endocrinology & Metabolism (33%)**	**Highly related fields**	Raw citations	1785 (<0.001)	49.4 (0.764)	7.44 (0.234)
		Composite	3.06 (<0.001)	-.00906 (0.738)	.00488 (<0.001)
**Respiratory System (33%)**	**Highly related fields**	Raw citations	1545 (<0.001)	110 (0.443)	11.1 (0.063)
		Composite	2.86 (<0.001)	.00952 (0.720)	.00504 (<0.001)
**Nuclear Medicine & Medical Imaging (32%)**	**Highly related fields**	Raw citations	968 (<0.001)	332 (<0.001)	1.75 (0.520)
		Composite	2.65 (<0.001)	.118 (<0.001)	.00304 (<0.001)

© 2022 Ioannidis JPA et al. *JAMA Network Open.*

**Cardiovascular System & Hematology (32%)**	**Highly related fields**	Raw citations	2767 (<0.001)	642 (<0.001)	-1.31 (0.830)
		Composite	2.95 (<0.001)	.0824 (<0.001)	.00418 (<0.001)
**Health Policy & Services (31%)**	**Highly related fields**	Raw citations	749 (0.008)	177 (0.354)	17.9 (0.023)
		Composite	2.82 (<0.001)	.1 (0.032)	.00711 (<0.001)
**Microbiology (31%)**	**Highly related fields**	Raw citations	2694 (<0.001)	8.58 (0.952)	-24.2 (<0.001)
		Composite	2.94 (<0.001)	.0275 (0.150)	.00397 (<0.001)
**Toxicology (31%)**	**Highly related fields**	Raw citations	1040 (<0.001)	729 (<0.001)	.942 (0.885)
		Composite	2.69 (<0.001)	.121 (0.001)	.00558 (<0.001)
**Pediatrics (31%)**	**Highly related fields**	Raw citations	704 (<0.001)	294 (<0.001)	4.8 (0.033)
		Composite	2.6 (<0.001)	.0875 (<0.001)	.00409 (<0.001)
**Biophysics (31%)**	**Highly related fields**	Raw citations	1228 (0.001)	-204 (0.443)	-2.28 (0.796)
		Composite	2.57 (<0.001)	.0794 (0.128)	.00663 (<0.001)
**Rehabilitation (30%)**	**Highly related fields**	Raw citations	428 (0.004)	197 (0.038)	11.5 (0.005)
		Composite	2.7 (<0.001)	-.0139 (0.714)	.00651 (<0.001)
**Urology & Nephrology (30%)**	**Highly related fields**	Raw citations	1878 (<0.001)	99.8 (0.505)	-6.43 (0.257)
		Composite	2.81 (<0.001)	.106 (<0.001)	.00398 (<0.001)
**Ophthalmology & Optometry (30%)**	**Highly related fields**	Raw citations	1186 (<0.001)	409 (<0.001)	-2.45 (0.537)
		Composite	2.69 (<0.001)	.154 (<0.001)	.00432 (<0.001)
**Physiology (29%)**	**Highly related fields**	Raw citations	603 (<0.001)	136 (0.071)	4.35 (0.072)
		Composite	2.86 (<0.001)	.0303 (0.448)	.00386 (0.003)
**Biochemistry & Molecular Biology (29%)**	**Highly related fields**	Raw citations	1535 (<0.001)	113 (0.149)	-4.76 (0.071)
		Composite	2.85 (<0.001)	.0271 (0.140)	.00422 (<0.001)
**Clinical Psychology (28%)**	**Highly related fields**	Raw citations	890 (<0.001)	471 (0.018)	16.1 (0.011)
		Composite	3.08 (<0.001)	.0664 (0.219)	.00474 (0.006)
**Medicinal & Biomolecular Chemistry (28%)**	**Highly related fields**	Raw citations	588 (<0.001)	190 (0.093)	10.4 (0.007)
		Composite	2.47 (<0.001)	.0491 (0.174)	.0072 (<0.001)

© 2022 Ioannidis JPA et al. *JAMA Network Open.*

**Obstetrics & Reproductive Medicine (28%)**	**Highly related fields**	Raw citations	917 (<0.001)	202 (0.035)	2.97 (0.395)
		Composite	2.75 (<0.001)	.0575 (0.045)	.00332 (0.002)
**Anesthesiology (27%)**	**Highly related fields**	Raw citations	891 (<0.001)	244 (0.061)	-.264 (0.958)
		Composite	2.61 (<0.001)	.11 (0.003)	.00495 (<0.001)
**Tropical Medicine (27%)**	**Highly related fields**	Raw citations	1428 (<0.001)	145 (0.639)	-7.89 (0.416)
		Composite	2.59 (<0.001)	.0525 (0.224)	.00484 (<0.001)
**Pharmacology & Pharmacy (25%)**	**Highly related fields**	Raw citations	605 (<0.001)	358 (<0.001)	4.33 (0.064)
		Composite	2.58 (<0.001)	.106 (<0.001)	.0051 (<0.001)
**Nutrition & Dietetics (25%)**	**Highly related fields**	Raw citations	995 (<0.001)	502 (0.003)	9 (0.107)
		Composite	2.95 (<0.001)	.0784 (0.060)	.00515 (<0.001)
**Nursing (25%)**	**Highly related fields**	Raw citations	140 (0.015)	226 (<0.001)	8.23 (<0.001)
		Composite	2.39 (<0.001)	.0803 (0.002)	.00504 (<0.001)
**Demography (25%)**	**Highly related fields**	Raw citations	-165 (0.590)	609 (0.009)	18.5 (0.016)
		Composite	2.74 (<0.001)	.148 (0.184)	.00817 (0.030)
**Speech-Language Pathology & Audiology (25%)**	**Highly related fields**	Raw citations	1059 (<0.001)	28 (0.886)	-8.84 (0.232)
		Composite	2.81 (<0.001)	.00573 (0.919)	.00424 (0.047)
**Statistics & Probability (24%)**	**Other fields**	Raw citations	942 (0.021)	843 (0.015)	14.7 (0.120)
		Composite	3.09 (<0.001)	.0833 (0.141)	.0033 (0.034)
**Family Studies (24%)**	**Other fields**	Raw citations	245 (0.052)	325 (0.002)	7 (0.042)
		Composite	2.81 (<0.001)	.161 (0.014)	.00634 (0.005)
**Environmental & Occupational Health (23%)**	**Highly related fields**	Raw citations	462 (0.218)	-153 (0.547)	11.1 (0.280)
		Composite	2.56 (<0.001)	-.0146 (0.802)	.00391 (0.098)
**Experimental Psychology (22%)**	**Highly related fields**	Raw citations	980 (<0.001)	330 (0.003)	3.88 (0.197)
		Composite	3.12 (<0.001)	.0657 (0.051)	.00392 (<0.001)
**Applied Ethics (22%)**	**Highly related fields**	Raw citations	798 (<0.001)	292 (0.069)	-3.71 (0.489)
		Composite	3.02 (<0.001)	.0736 (0.375)	.000696 (0.804)

© 2022 Ioannidis JPA et al. *JAMA Network Open.*

**Organic Chemistry (22%)**	**Other fields**	Raw citations	1298 (<0.001)	324 (0.012)	-1.26 (0.702)
		Composite	2.85 (<0.001)	.0937 (0.006)	.00407 (<0.001)
**Optics (22%)**	**Other fields**	Raw citations	1038 (<0.001)	181 (0.367)	13.6 (0.045)
		Composite	2.67 (<0.001)	.0303 (0.461)	.0078 (<0.001)
**Microscopy (21%)**	**Highly related fields**	Raw citations	636 (0.253)	260 (0.554)	10 (0.491)
		Composite	2.42 (<0.001)	.0882 (0.574)	.0107 (0.047)
**General & Internal Medicine (21%)**	**Highly related fields**	Raw citations	3261 (<0.001)	-114 (0.736)	-35.6 (<0.001)
		Composite	2.58 (<0.001)	.176 (<0.001)	.00249 (<0.001)
**Biotechnology (21%)**	**Highly related fields**	Raw citations	715 (0.003)	60 (0.782)	21.1 (0.005)
		Composite	2.63 (<0.001)	.0562 (0.316)	.0109 (<0.001)
**Otorhinolaryngology (20%)**	**Highly related fields**	Raw citations	901 (<0.001)	.501 (0.996)	-4.95 (0.116)
		Composite	2.6 (<0.001)	.0495 (0.080)	.00214 (0.015)
**Surgery (20%)**	**Highly related fields**	Raw citations	928 (<0.001)	478 (<0.001)	5.87 (0.058)
		Composite	2.6 (<0.001)	.0936 (<0.001)	.00384 (<0.001)
**General Chemistry (19%)**	**Other fields**	Raw citations	1459 (<0.001)	-43.1 (0.889)	.159 (0.973)
		Composite	2.62 (<0.001)	.126 (0.029)	.00576 (<0.001)
**Mycology & Parasitology (18%)**	**Highly related fields**	Raw citations	1377 (<0.001)	-247 (0.255)	-9.55 (0.107)
		Composite	2.78 (<0.001)	-.00229 (0.977)	.00178 (0.408)
**Nanoscience & Nanotechnology (18%)**	**Other fields**	Raw citations	1719 (<0.001)	29.8 (0.947)	98.7 (<0.001)
		Composite	2.98 (<0.001)	.0545 (0.157)	.0131 (<0.001)
**Behavioral Science & Comparative Psychology (17%)**	**Highly related fields**	Raw citations	715 (<0.001)	204 (0.109)	1.51 (0.671)
		Composite	2.94 (<0.001)	.0557 (0.353)	.0049 (0.004)
**Dermatology & Venereal Diseases (16%)**	**Highly related fields**	Raw citations	1168 (<0.001)	708 (0.002)	.0314 (0.996)
		Composite	2.76 (<0.001)	.0719 (0.146)	.00366 (0.009)
**Acoustics (16%)**	**Other fields**	Raw citations	958 (<0.001)	5.66 (0.963)	-7.77 (0.007)
		Composite	2.85 (<0.001)	-.0544 (0.321)	.000337 (0.791)

© 2022 Ioannidis JPA et al. *JAMA Network Open.*

**Orthopedics (15%)**	**Highly related fields**	Raw citations	1128 (<0.001)	313 (0.002)	-.821 (0.768)
		Composite	2.79 (<0.001)	.114 (<0.001)	.0039 (<0.001)
**Environmental Sciences (15%)**	**Other fields**	Raw citations	1593 (<0.001)	923 (0.010)	2.43 (0.809)
		Composite	3.06 (<0.001)	.113 (0.057)	.00346 (0.040)
**Sociology (13%)**	**Other fields**	Raw citations	385 (<0.001)	57 (0.483)	6.31 (0.002)
		Composite	3.05 (<0.001)	.00144 (0.978)	.00394 (0.003)
**Complementary & Alternative Medicine (12%)**	**Highly related fields**	Raw citations	298 (0.019)	284 (0.024)	3.2 (0.461)
		Composite	2.54 (<0.001)	.195 (0.120)	.000627 (0.887)
**Dentistry (12%)**	**Highly related fields**	Raw citations	796 (<0.001)	391 (<0.001)	-2.76 (0.271)
		Composite	2.69 (<0.001)	.15 (0.001)	.00244 (0.025)
**Distributed Computing (12%)**	**Other fields**	Raw citations	314 (0.343)	627 (0.056)	13.4 (0.228)
		Composite	2.38 (<0.001)	-.0301 (0.754)	.00461 (0.160)
**Pathology (11%)**	**Highly related fields**	Raw citations	1747 (<0.001)	418 (0.169)	-4.81 (0.539)
		Composite	2.82 (<0.001)	.0974 (0.108)	.00255 (0.104)
**Evolutionary Biology (11%)**	**Other fields**	Raw citations	1049 (<0.001)	465 (0.145)	8.82 (0.257)
		Composite	2.99 (<0.001)	.0804 (0.101)	.00719 (<0.001)
**Artificial Intelligence & Image Processing (11%)**	**Other fields**	Raw citations	1797 (<0.001)	-95.5 (0.694)	-4.07 (0.512)
		Composite	2.78 (<0.001)	-.0148 (0.670)	.00718 (<0.001)
**Veterinary Sciences (11%)**	**Highly related fields**	Raw citations	464 (<0.001)	158 (0.004)	1.12 (0.462)
		Composite	2.5 (<0.001)	.0864 (0.013)	.00304 (0.001)
**Plant Biology & Botany (10%)**	**Other fields**	Raw citations	1183 (<0.001)	578 (<0.001)	-1.7 (0.593)
		Composite	2.87 (<0.001)	.114 (0.003)	.00448 (<0.001)
**Chemical Physics (10%)**	**Other fields**	Raw citations	1676 (<0.001)	315 (0.274)	5.26 (0.297)
		Composite	3.02 (<0.001)	.112 (0.014)	.0042 (<0.001)
**Industrial Engineering & Automation (10%)**	**Other fields**	Raw citations	1099 (<0.001)	55.1 (0.709)	-2.81 (0.420)
		Composite	2.89 (<0.001)	-.019 (0.720)	.00283 (0.024)

© 2022 Ioannidis JPA et al. *JAMA Network Open.*

**Sport Sciences (10%)**	**Highly related fields**	Raw citations	895 (0.005)	388 (0.261)	11.8 (0.218)
		Composite	3.04 (<0.001)	.0182 (0.814)	.00208 (0.333)
**Drama & Theater (10%)**	**Other fields**	Raw citations	82.1 (0.212)	3.11 (0.960)	-.644 (0.765)
		Composite	1.95 (<0.001)	.146 (0.424)	.00562 (0.376)
**Social Work (9.6%)**	**Other fields**	Raw citations	143 (0.105)	551 (<0.001)	8.07 (0.010)
		Composite	2.74 (<0.001)	.198 (0.010)	.00226 (0.262)
**Criminology (9.6%)**	**Other fields**	Raw citations	395 (<0.001)	295 (0.023)	9.47 (0.003)
		Composite	2.98 (<0.001)	.121 (0.063)	.00524 (0.001)
**Design Practice & Management (9%)**	**Other fields**	Raw citations	606 (<0.001)	437 (0.055)	.541 (0.897)
		Composite	2.79 (<0.001)	-.0125 (0.929)	.00115 (0.657)
**Polymers (8.5%)**	**Other fields**	Raw citations	1292 (<0.001)	556 (0.016)	-1.92 (0.633)
		Composite	2.88 (<0.001)	.0689 (0.266)	.00304 (0.005)
**General Clinical Medicine (7.8%)**	**Highly related fields**	Raw citations	282 (0.050)	404 (0.051)	4.72 (0.204)
		Composite	2.2 (<0.001)	.0323 (0.786)	.00712 (0.001)
**Geography (7.5%)**	**Other fields**	Raw citations	283 (0.020)	356 (0.037)	8.5 (0.016)
		Composite	2.98 (<0.001)	.169 (0.085)	.00521 (0.011)
**Social Psychology (7.5%)**	**Highly related fields**	Raw citations	1188 (<0.001)	424 (0.146)	9.77 (0.045)
		Composite	3.19 (<0.001)	.139 (0.042)	.00442 (<0.001)
**Development Studies (7.4%)**	**Other fields**	Raw citations	588 (0.014)	-109 (0.685)	1.96 (0.759)
		Composite	3.13 (<0.001)	-.199 (0.293)	.00232 (0.604)
**Economics (7.2%)**	**Other fields**	Raw citations	743 (<0.001)	387 (0.012)	4.46 (0.119)
		Composite	3.08 (<0.001)	.161 (0.005)	.00515 (<0.001)
**Fluids & Plasmas (7.1%)**	**Other fields**	Raw citations	1573 (<0.001)	505 (0.153)	-9.19 (0.148)
		Composite	3.1 (<0.001)	.158 (0.037)	.00142 (0.297)
**Social Sciences Methods (7%)**	**Other fields**	Raw citations	964 (0.103)	1194 (0.212)	9.64 (0.459)
		Composite	3.14 (<0.001)	.485 (0.002)	.00348 (0.103)

© 2022 Ioannidis JPA et al. *JAMA Network Open.*

**Entomology (6.9%)**	**Other fields**	Raw citations	984 (<0.001)	440 (<0.001)	-8.51 (0.001)
		Composite	2.92 (<0.001)	.27 (<0.001)	-.00155 (0.266)
**Numerical & Computational Mathematics (6.9%)**	**Other fields**	Raw citations	538 (0.006)	556 (0.024)	5.04 (0.285)
		Composite	3.1 (<0.001)	.0774 (0.514)	-.0000828 (0.971)
**Electrical & Electronic Engineering (6.9%)**	**Other fields**	Raw citations	416 (<0.001)	170 (0.330)	10 (0.003)
		Composite	2.46 (<0.001)	.0702 (0.350)	.00625 (<0.001)
**Gender Studies (6.7%)**	**Highly related fields**	Raw citations	395 (0.084)	-186 (0.531)	2.48 (0.681)
		Composite	2.9 (<0.001)	.0222 (0.912)	.00468 (0.268)
**Computer Hardware & Architecture (6.6%)**	**Other fields**	Raw citations	597 (<0.001)	119 (0.413)	2.76 (0.363)
		Composite	2.46 (<0.001)	.0401 (0.587)	.00471 (0.003)
**Logistics & Transportation (6.6%)**	**Other fields**	Raw citations	667 (<0.001)	102 (0.683)	7.13 (0.174)
		Composite	2.96 (<0.001)	-.0995 (0.366)	.00357 (0.124)
**Food Science (6.5%)**	**Other fields**	Raw citations	1030 (0.003)	528 (0.317)	4.53 (0.630)
		Composite	2.87 (<0.001)	.0813 (0.424)	.00537 (0.003)
**Information & Library Sciences (6.3%)**	**Other fields**	Raw citations	253 (0.001)	172 (0.218)	1.77 (0.457)
		Composite	2.62 (<0.001)	.235 (0.061)	.00172 (0.417)
**Psychoanalysis (6.1%)**	**Highly related fields**	Raw citations	51 (0.729)	182 (0.366)	2.62 (0.329)
		Composite	2.5 (<0.001)	.236 (0.389)	.000928 (0.799)
**Education (6%)**	**Other fields**	Raw citations	346 (<0.001)	233 (0.004)	7.41 (<0.001)
		Composite	2.89 (<0.001)	.0232 (0.606)	.0042 (<0.001)
**Archaeology (5.9%)**	**Other fields**	Raw citations	417 (<0.001)	3.67 (0.984)	.996 (0.725)
		Composite	2.81 (<0.001)	-.0678 (0.535)	.00151 (0.386)
**Optoelectronics & Photonics (5.9%)**	**Other fields**	Raw citations	477 (<0.001)	222 (0.038)	1.78 (0.333)
		Composite	2.31 (<0.001)	.0967 (0.072)	.00576 (<0.001)
**History of Social Sciences (5.9%)**	**Other fields**	Raw citations	-114 (0.589)	-126 (0.655)	10.6 (0.043)
		Composite	2.26 (<0.001)	-.237 (0.403)	.014 (0.011)

© 2022 Ioannidis JPA et al. *JAMA Network Open.*

**Science Studies (5.7%)**	**Other fields**	Raw citations	244 (0.310)	-101 (0.767)	12.1 (0.066)
		Composite	3.03 (<0.001)	-.185 (0.456)	.00637 (0.174)
**Human Factors (5.6%)**	**Highly related fields**	Raw citations	739 (<0.001)	45.7 (0.854)	7.17 (0.152)
		Composite	2.88 (<0.001)	.00908 (0.921)	.00762 (<0.001)
**Urban & Regional Planning (5.6%)**	**Other fields**	Raw citations	561 (0.001)	332 (0.249)	.96 (0.842)
		Composite	3.13 (<0.001)	.195 (0.210)	.00245 (0.346)
**Networking & Telecommunications (5.2%)**	**Other fields**	Raw citations	886 (<0.001)	-177 (0.208)	-1.95 (0.335)
		Composite	2.63 (<0.001)	-.0825 (0.105)	.0037 (<0.001)
**Dairy & Animal Science (5.1%)**	**Other fields**	Raw citations	730 (<0.001)	233 (0.040)	-3.4 (0.070)
		Composite	2.63 (<0.001)	.0804 (0.271)	.0025 (0.039)
**Languages & Linguistics (5.1%)**	**Other fields**	Raw citations	427 (<0.001)	-57.6 (0.656)	.771 (0.749)
		Composite	3.05 (<0.001)	-.00505 (0.967)	.00283 (0.216)
**Building & Construction (4.9%)**	**Other fields**	Raw citations	752 (0.001)	449 (0.239)	12.7 (0.065)
		Composite	2.94 (<0.001)	.0109 (0.937)	.00728 (0.004)
**Religions & Theology (4.9%)**	**Other fields**	Raw citations	143 (0.002)	12.9 (0.895)	-.325 (0.827)
		Composite	2.33 (<0.001)	.0544 (0.763)	.000519 (0.850)
**Inorganic & Nuclear Chemistry (4.9%)**	**Other fields**	Raw citations	909 (0.001)	196 (0.677)	5.08 (0.390)
		Composite	2.77 (<0.001)	-.000647 (0.995)	.00296 (0.037)
**Applied Mathematics (4.8%)**	**Other fields**	Raw citations	839 (0.030)	2290 (<0.001)	9.42 (0.301)
		Composite	3.03 (<0.001)	.355 (0.045)	.00509 (0.052)
**Mechanical Engineering & Transports (4.7%)**	**Other fields**	Raw citations	971 (<0.001)	653 (0.005)	-3.37 (0.311)
		Composite	2.83 (<0.001)	-.045 (0.589)	.00343 (0.004)
**General Psychology & Cognitive Sciences (4.7%)**	**Highly related fields**	Raw citations	664 (<0.001)	185 (0.530)	.446 (0.924)
		Composite	3 (<0.001)	-.184 (0.299)	.000869 (0.757)
**Mining & Metallurgy (4.7%)**	**Other fields**	Raw citations	154 (0.286)	528 (0.036)	5.41 (0.141)
		Composite	2.25 (<0.001)	.438 (0.062)	.0068 (0.051)

© 2022 Ioannidis JPA et al. *JAMA Network Open.*

**Software Engineering (4.5%)**	**Other fields**	Raw citations	1084 (<0.001)	293 (0.084)	-13.3 (<0.001)
		Composite	2.8 (<0.001)	-.0675 (0.447)	.00108 (0.535)
**Anthropology (4.3%)**	**Other fields**	Raw citations	291 (<0.001)	113 (0.344)	4.83 (0.018)
		Composite	2.97 (<0.001)	-.0875 (0.415)	.00273 (0.135)
**Materials (4.3%)**	**Other fields**	Raw citations	1235 (<0.001)	154 (0.550)	-1.51 (0.626)
		Composite	2.75 (<0.001)	.103 (0.087)	.00508 (<0.001)
**Chemical Engineering (4.3%)**	**Other fields**	Raw citations	1517 (<0.001)	355 (0.340)	-8.67 (0.063)
		Composite	2.98 (<0.001)	.0989 (0.410)	.00212 (0.159)
**Accounting (4.1%)**	**Other fields**	Raw citations	457 (0.017)	-35.1 (0.902)	12.4 (0.033)
		Composite	2.94 (<0.001)	-.125 (0.443)	.00754 (0.024)
**Econometrics (4.1%)**	**Other fields**	Raw citations	591 (0.479)	-366 (0.763)	27.9 (0.209)
		Composite	3.27 (<0.001)	-.44 (0.187)	.00673 (0.265)
**Ecology (3.9%)**	**Other fields**	Raw citations	1298 (<0.001)	-72.3 (0.806)	11.6 (0.025)
		Composite	3.07 (<0.001)	.0478 (0.422)	.00549 (<0.001)
**Sport, Leisure & Tourism (3.8%)**	**Other fields**	Raw citations	701 (<0.001)	-147 (0.576)	4.48 (0.382)
		Composite	3.09 (<0.001)	-.0594 (0.650)	.00439 (0.086)
**Legal & Forensic Medicine (3.8%)**	**Highly related fields**	Raw citations	277 (0.003)	-118 (0.515)	.588 (0.850)
		Composite	2.22 (<0.001)	-.037 (0.837)	.00542 (0.084)
**Communication & Media Studies (3.7%)**	**Other fields**	Raw citations	108 (0.441)	6.53 (0.984)	20.6 (<0.001)
		Composite	3.04 (<0.001)	-.0162 (0.877)	.00426 (0.006)
**Physical Chemistry (3.7%)**	**Other fields**	Raw citations	1889 (<0.001)	-162 (0.837)	1.19 (0.893)
		Composite	2.92 (<0.001)	-.0478 (0.798)	.00667 (0.002)
**Operations Research (3.6%)**	**Other fields**	Raw citations	523 (0.040)	-82 (0.857)	12.8 (0.054)
		Composite	3.09 (<0.001)	.0494 (0.709)	.00316 (0.102)
**Anatomy & Morphology (3.6%)**	**Highly related fields**	Raw citations	344 (<0.001)	60.3 (0.756)	-1.92 (0.357)
		Composite	2.46 (<0.001)	-.0653 (0.743)	-.000483 (0.821)

© 2022 Ioannidis JPA et al. *JAMA Network Open.*

**Information Systems (3.6%)**	**Other fields**	Raw citations	1212 (<0.001)	-39.3 (0.943)	-1.41 (0.887)
		Composite	3.16 (<0.001)	.0141 (0.923)	.00017 (0.948)
**Marketing (3.5%)**	**Other fields**	Raw citations	195 (0.377)	-72.8 (0.841)	27.6 (<0.001)
		Composite	3.01 (<0.001)	.00894 (0.932)	.00571 (0.005)
**Ornithology (3.4%)**	**Other fields**	Raw citations	404 (0.002)	-16.7 (0.952)	.54 (0.850)
		Composite	2.57 (<0.001)	.287 (0.139)	.00354 (0.077)
**General Physics (3.3%)**	**Other fields**	Raw citations	1266 (<0.001)	344 (0.265)	-6.75 (0.032)
		Composite	2.78 (<0.001)	.0854 (0.379)	.00337 (0.001)
**Finance (3.3%)**	**Other fields**	Raw citations	571 (0.004)	-336 (0.372)	14.1 (0.017)
		Composite	3 (<0.001)	-.145 (0.286)	.00721 (0.001)
**Zoology (3.3%)**	**Other fields**	Raw citations	428 (0.001)	257 (0.234)	.604 (0.869)
		Composite	2.65 (<0.001)	.0414 (0.772)	-.0011 (0.653)
**Oceanography (3%)**	**Other fields**	Raw citations	1007 (<0.001)	299 (0.229)	-5.66 (0.119)
		Composite	2.92 (<0.001)	.211 (0.082)	.00157 (0.373)
**Meteorology & Atmospheric Sciences (3%)**	**Other fields**	Raw citations	1771 (<0.001)	138 (0.587)	1.29 (0.723)
		Composite	3.09 (<0.001)	.0817 (0.124)	.00339 (<0.001)
**Applied Physics (2.8%)**	**Other fields**	Raw citations	1450 (<0.001)	299 (0.275)	-2.3 (0.387)
		Composite	2.84 (<0.001)	.0262 (0.645)	.00263 (<0.001)
**Geological & Geomatics Engineering (2.8%)**	**Other fields**	Raw citations	1323 (<0.001)	912 (0.019)	-1.47 (0.784)
		Composite	2.96 (<0.001)	.271 (0.017)	.00517 (0.001)
**Aerospace & Aeronautics (2.8%)**	**Other fields**	Raw citations	401 (<0.001)	93.9 (0.289)	-.0888 (0.936)
		Composite	2.44 (<0.001)	-.0482 (0.534)	.00448 (<0.001)
**Marine Biology & Hydrobiology (2.8%)**	**Other fields**	Raw citations	1529 (<0.001)	61.8 (0.850)	-8.91 (0.090)
		Composite	3.06 (<0.001)	-.0715 (0.480)	.00219 (0.179)
**Cultural Studies (2.8%)**	**Other fields**	Raw citations	74.7 (0.319)	-92.6 (0.578)	2.56 (0.252)
		Composite	2.44 (<0.001)	-.17 (0.551)	.0056 (0.147)

© 2022 Ioannidis JPA et al. *JAMA Network Open.*

**Energy (2.6%)**	**Other fields**	Raw citations	1012 (<0.001)	304 (0.182)	.717 (0.770)
		Composite	2.85 (<0.001)	.0828 (0.233)	.00398 (<0.001)
**Environmental Engineering (2.5%)**	**Other fields**	Raw citations	1064 (<0.001)	1045 (0.002)	-1.87 (0.658)
		Composite	2.97 (<0.001)	.111 (0.282)	.002 (0.123)
**Geology (2.4%)**	**Other fields**	Raw citations	926 (<0.001)	-295 (0.341)	-6.59 (0.108)
		Composite	3.04 (<0.001)	-.168 (0.410)	-.000855 (0.750)
**Business & Management (2.3%)**	**Other fields**	Raw citations	761 (<0.001)	-18.9 (0.948)	12.4 (0.001)
		Composite	3.11 (<0.001)	.0511 (0.525)	.00466 (<0.001)
**Strategic, Defence & Security Studies (2.3%)**	**Other fields**	Raw citations	246 (0.105)	-146 (0.704)	11 (0.014)
		Composite	2.7 (<0.001)	.128 (0.490)	.00777 (<0.001)
**Computation Theory & Mathematics (2.3%)**	**Other fields**	Raw citations	686 (<0.001)	344 (0.340)	-.292 (0.942)
		Composite	2.93 (<0.001)	.203 (0.184)	.00233 (0.169)
**Law (2.2%)**	**Other fields**	Raw citations	81.5 (0.691)	-4.51 (0.990)	6.04 (0.411)
		Composite	2.45 (<0.001)	-.00751 (0.970)	.00446 (0.274)
**Political Science & Public Administration (2%)**	**Other fields**	Raw citations	463 (<0.001)	1092 (<0.001)	6.15 (0.040)
		Composite	3.05 (<0.001)	.312 (0.007)	.00406 (0.001)
**History (2%)**	**Other fields**	Raw citations	72.9 (0.026)	-33.6 (0.729)	.299 (0.726)
		Composite	2.12 (<0.001)	-.0867 (0.623)	.00174 (0.262)
**International Relations (1.8%)**	**Other fields**	Raw citations	120 (0.151)	-104 (0.655)	9.67 (<0.001)
		Composite	2.82 (<0.001)	-.296 (0.231)	.00875 (0.002)
**Astronomy & Astrophysics (1.7%)**	**Other fields**	Raw citations	3149 (<0.001)	-813 (0.213)	-24.2 (<0.001)
		Composite	3.17 (<0.001)	.0159 (0.837)	.000621 (0.438)
**General Mathematics (1.7%)**	**Other fields**	Raw citations	452 (<0.001)	82.7 (0.590)	-.899 (0.410)
		Composite	2.9 (<0.001)	-.0362 (0.732)	.000608 (0.418)
**Fisheries (1.6%)**	**Other fields**	Raw citations	713 (<0.001)	394 (0.172)	.749 (0.830)
		Composite	2.77 (<0.001)	.158 (0.337)	.00279 (0.162)

© 2022 Ioannidis JPA et al. *JAMA Network Open.*

**Philosophy (1.6%)**	**Other fields**	Raw citations	242 (<0.001)	476 (<0.001)	.346 (0.761)
		Composite	2.91 (<0.001)	.712 (<0.001)	.000684 (0.676)
**Literary Studies (1.4%)**	**Other fields**	Raw citations	30.4 (0.415)	24 (0.820)	1.96 (0.143)
		Composite	1.93 (<0.001)	.34 (0.201)	.00816 (0.016)
**Agricultural Economics & Policy (1.4%)**	**Other fields**	Raw citations	990 (<0.001)	-8.71 (0.989)	-2.71 (0.698)
		Composite	3.24 (<0.001)	.033 (0.915)	-.0016 (0.641)
**Civil Engineering (1.4%)**	**Other fields**	Raw citations	533 (<0.001)	218 (0.591)	6.66 (0.072)
		Composite	2.77 (<0.001)	-.00115 (0.995)	.00495 (0.007)
**Geochemistry & Geophysics (1.3%)**	**Other fields**	Raw citations	1303 (<0.001)	-225 (0.359)	-4.81 (0.042)
		Composite	3.15 (<0.001)	-.101 (0.271)	.00174 (0.050)
**Forestry (1.1%)**	**Other fields**	Raw citations	652 (<0.001)	-132 (0.681)	-.812 (0.812)
		Composite	2.62 (<0.001)	-.0327 (0.873)	.00351 (0.106)
**Nuclear & Particle Physics (1.1%)**	**Other fields**	Raw citations	3809 (<0.001)	-755 (0.501)	-40.4 (<0.001)
		Composite	2.89 (<0.001)	.0162 (0.880)	.00251 (<0.001)
**Agronomy & Agriculture (.83%)**	**Other fields**	Raw citations	1177 (<0.001)	432 (0.482)	-2.86 (0.486)
		Composite	2.88 (<0.001)	.226 (0.227)	.00291 (0.020)
**Paleontology (.74%)**	**Other fields**	Raw citations	1198 (<0.001)	-586 (0.551)	-4.82 (0.489)
		Composite	3.14 (<0.001)	-.258 (0.241)	-.0000303 (0.984)
**Music (0%)**	**Other fields**	Raw citations			
		Composite			
**Mathematical Physics (0%)**	**Other fields**	Raw citations			
		Composite			
**Industrial Relations (0%)**	**Other fields**	Raw citations			
		Composite			
**Horticulture (0%)**	**Other fields**	Raw citations			
		Composite			
**History of Science, Technology & Medicine (0%)**	**Other fields**	Raw citations			
		Composite			

© 2022 Ioannidis JPA et al. *JAMA Network Open.*

**Folklore (0%)**	**Other fields**	Raw citations			
		Composite			
**Economic Theory (0%)**	**Other fields**	Raw citations			
		Composite			
**Classics (0%)**	**Other fields**	Raw citations			
		Composite			
**Automobile Design & Engineering (0%)**	**Other fields**	Raw citations			
		Composite			
**Art Practice, History & Theory (0%)**	**Other fields**	Raw citations			
		Composite			
**Architecture (0%)**	**Other fields**	Raw citations			
		Composite			

© 2022 Ioannidis JPA et al. *JAMA Network Open.*

eTable 3.6 : Recent year impact, Funding time current funding Linear Regressions for each subfield (ordered by percentage funded)

Top-cited US-based researchers: Subfield (perc. funded)	Classification	Dependent Variable	Constant (p-val)	Funded (p-val)	Years since first pub (p-val)
**Geriatrics (43%)**	**Highly related fields**	Raw citations	1293 (0.144)	330 (0.501)	16.3 (0.454)
		Composite	2.97 (<0.001)	.083 (0.352)	.00586 (0.141)
**Developmental Biology (36%)**	**Highly related fields**	Raw citations	4064 (<0.001)	253 (0.282)	-27.8 (0.004)
		Composite	3.02 (<0.001)	.0166 (0.342)	.00658 (<0.001)
**Medical Informatics (34%)**	**Highly related fields**	Raw citations	790 (0.001)	204 (0.286)	2.4 (0.749)
		Composite	2.66 (<0.001)	.0811 (0.190)	.00485 (0.048)
**Bioinformatics (34%)**	**Highly related fields**	Raw citations	3646 (<0.001)	633 (0.345)	-13 (0.650)
		Composite	3 (<0.001)	.0606 (0.315)	.00348 (0.180)
**Substance Abuse (31%)**	**Highly related fields**	Raw citations	804 (<0.001)	306 (0.033)	13.8 (0.018)
		Composite	3.03 (<0.001)	-.00341 (0.933)	.00423 (0.010)
**Gerontology (31%)**	**Highly related fields**	Raw citations	1530 (0.155)	18.8 (0.979)	12.4 (0.657)
		Composite	3.02 (<0.001)	-.00842 (0.900)	.00507 (0.060)
**Biomedical Engineering (30%)**	**Highly related fields**	Raw citations	684 (0.002)	526 (0.003)	15.9 (0.013)
		Composite	2.69 (<0.001)	.0568 (0.071)	.00733 (<0.001)
**Virology (30%)**	**Highly related fields**	Raw citations	1648 (<0.001)	289 (0.064)	1.28 (0.823)
		Composite	2.75 (<0.001)	.0665 (0.010)	.00559 (<0.001)
**Immunology (30%)**	**Highly related fields**	Raw citations	2113 (<0.001)	-20.8 (0.881)	13.6 (0.011)
		Composite	2.98 (<0.001)	.0336 (0.110)	.00596 (<0.001)
**Neurology & Neurosurgery (29%)**	**Highly related fields**	Raw citations	2058 (<0.001)	147 (0.130)	2.82 (0.424)
		Composite	3.06 (<0.001)	.0234 (0.109)	.00424 (<0.001)
**Allergy (27%)**	**Highly related fields**	Raw citations	1238 (0.001)	56.5 (0.830)	17.6 (0.072)
		Composite	2.97 (<0.001)	.0356 (0.587)	.00594 (0.016)

© 2022 Ioannidis JPA et al. *JAMA Network Open.*

**Emergency & Critical Care Medicine (27%)**	**Highly related fields**	Raw citations	1029 (0.014)	782 (0.011)	22.2 (0.078)
		Composite	2.82 (<0.001)	.102 (0.033)	.0042 (0.034)
**Oncology & Carcinogenesis (26%)**	**Highly related fields**	Raw citations	3201 (<0.001)	381 (0.026)	-5.11 (0.429)
		Composite	2.93 (<0.001)	.0316 (0.031)	.00459 (<0.001)
**Public Health (26%)**	**Highly related fields**	Raw citations	881 (0.005)	38.7 (0.870)	27.5 (0.002)
		Composite	3.02 (<0.001)	.0222 (0.412)	.00512 (<0.001)
**Gastroenterology & Hepatology (24%)**	**Highly related fields**	Raw citations	1731 (<0.001)	269 (0.128)	3.51 (0.577)
		Composite	2.92 (<0.001)	.0809 (0.021)	.00443 (<0.001)
**Psychiatry (23%)**	**Highly related fields**	Raw citations	1246 (<0.001)	783 (<0.001)	25.6 (0.001)
		Composite	3.05 (<0.001)	.0432 (0.161)	.0061 (<0.001)
**Arthritis & Rheumatology (23%)**	**Highly related fields**	Raw citations	1826 (0.001)	496 (0.197)	14.2 (0.293)
		Composite	3.06 (<0.001)	.0397 (0.502)	.00425 (0.042)
**Epidemiology (22%)**	**Highly related fields**	Raw citations	1526 (0.099)	289 (0.677)	31.8 (0.178)
		Composite	3.1 (<0.001)	-.011 (0.889)	.00384 (0.155)
**Genetics & Heredity (22%)**	**Highly related fields**	Raw citations	2360 (<0.001)	521 (0.052)	-10.8 (0.255)
		Composite	2.86 (<0.001)	.0139 (0.698)	.00201 (0.114)
**Analytical Chemistry (22%)**	**Other fields**	Raw citations	1.0e+03 (<0.001)	425 (0.008)	.584 (0.900)
		Composite	2.75 (<0.001)	.0411 (0.323)	.00331 (0.006)
**Ophthalmology & Optometry (22%)**	**Highly related fields**	Raw citations	1306 (<0.001)	245 (0.041)	-3.83 (0.340)
		Composite	2.73 (<0.001)	.0999 (0.001)	.00386 (<0.001)
**Developmental & Child Psychology (22%)**	**Highly related fields**	Raw citations	712 (<0.001)	430 (0.001)	16.2 (<0.001)
		Composite	3.07 (<0.001)	.0305 (0.404)	.00545 (<0.001)
**Nuclear Medicine & Medical Imaging (22%)**	**Highly related fields**	Raw citations	1014 (<0.001)	333 (<0.001)	1.32 (0.627)
		Composite	2.68 (<0.001)	.0934 (<0.001)	.00269 (<0.001)
**Endocrinology & Metabolism (21%)**	**Highly related fields**	Raw citations	1804 (<0.001)	33.3 (0.858)	7.17 (0.246)
		Composite	3.04 (<0.001)	.0171 (0.577)	.00512 (<0.001)

© 2022 Ioannidis JPA et al. *JAMA Network Open.*

**Biophysics (21%)**	**Highly related fields**	Raw citations	1169 (0.002)	-156 (0.605)	-1.46 (0.869)
		Composite	2.59 (<0.001)	.0786 (0.184)	.00653 (<0.001)
**Toxicology (21%)**	**Highly related fields**	Raw citations	1144 (<0.001)	671 (0.001)	.418 (0.950)
		Composite	2.71 (<0.001)	.105 (0.012)	.00544 (<0.001)
**Cardiovascular System & Hematology (21%)**	**Highly related fields**	Raw citations	2934 (<0.001)	511 (0.009)	-3.39 (0.578)
		Composite	2.97 (<0.001)	.0645 (0.003)	.00391 (<0.001)
**Respiratory System (21%)**	**Highly related fields**	Raw citations	1614 (<0.001)	5.34 (0.974)	10 (0.091)
		Composite	2.87 (<0.001)	-.00743 (0.807)	.00489 (<0.001)
**Physiology (21%)**	**Highly related fields**	Raw citations	662 (<0.001)	39.5 (0.642)	3.65 (0.134)
		Composite	2.86 (<0.001)	.036 (0.424)	.00388 (0.003)
**Rehabilitation (21%)**	**Highly related fields**	Raw citations	398 (0.005)	316 (0.003)	12.3 (0.003)
		Composite	2.7 (<0.001)	-.0147 (0.732)	.00652 (<0.001)
**Microbiology (20%)**	**Highly related fields**	Raw citations	2630 (<0.001)	173 (0.297)	-23.2 (<0.001)
		Composite	2.93 (<0.001)	.0481 (0.028)	.00407 (<0.001)
**Urology & Nephrology (20%)**	**Highly related fields**	Raw citations	1917 (<0.001)	47.8 (0.780)	-7 (0.216)
		Composite	2.83 (<0.001)	.0954 (0.002)	.00371 (<0.001)
**Medicinal & Biomolecular Chemistry (20%)**	**Highly related fields**	Raw citations	564 (<0.001)	295 (0.021)	11 (0.005)
		Composite	2.47 (<0.001)	.0665 (0.102)	.00731 (<0.001)
**Health Policy & Services (20%)**	**Highly related fields**	Raw citations	806 (0.004)	80.9 (0.717)	17.4 (0.029)
		Composite	2.84 (<0.001)	.061 (0.266)	.00691 (<0.001)
**Pediatrics (19%)**	**Highly related fields**	Raw citations	834 (<0.001)	122 (0.109)	3 (0.185)
		Composite	2.63 (<0.001)	.0545 (0.051)	.00367 (<0.001)
**Obstetrics & Reproductive Medicine (19%)**	**Highly related fields**	Raw citations	1012 (<0.001)	46.9 (0.670)	1.57 (0.654)
		Composite	2.77 (<0.001)	.0234 (0.478)	.003 (0.004)
**Anesthesiology (18%)**	**Highly related fields**	Raw citations	949 (<0.001)	205 (0.172)	-1.18 (0.813)
		Composite	2.64 (<0.001)	.0743 (0.079)	.00441 (0.002)

© 2022 Ioannidis JPA et al. *JAMA Network Open.*

**Statistics & Probability (17%)**	**Other fields**	Raw citations	1055 (0.008)	811 (0.036)	13.3 (0.157)
		Composite	3.1 (<0.001)	.093 (0.143)	.00324 (0.036)
**Biochemistry & Molecular Biology (17%)**	**Highly related fields**	Raw citations	1481 (<0.001)	282 (0.003)	-3.82 (0.146)
		Composite	2.84 (<0.001)	.0613 (0.006)	.0044 (<0.001)
**Speech-Language Pathology & Audiology (17%)**	**Highly related fields**	Raw citations	1188 (<0.001)	-254 (0.262)	-11.2 (0.130)
		Composite	2.82 (<0.001)	-.0227 (0.728)	.004 (0.062)
**Demography (17%)**	**Highly related fields**	Raw citations	-70.6 (0.826)	504 (0.072)	17.8 (0.028)
		Composite	2.79 (<0.001)	.0537 (0.684)	.00757 (0.050)
**Experimental Psychology (16%)**	**Highly related fields**	Raw citations	969 (<0.001)	476 (<0.001)	4.14 (0.166)
		Composite	3.11 (<0.001)	.12 (0.002)	.00407 (<0.001)
**Pharmacology & Pharmacy (15%)**	**Highly related fields**	Raw citations	692 (<0.001)	195 (0.034)	3.56 (0.134)
		Composite	2.6 (<0.001)	.0519 (0.145)	.00485 (<0.001)
**Clinical Psychology (15%)**	**Highly related fields**	Raw citations	1058 (<0.001)	217 (0.386)	14.2 (0.025)
		Composite	3.11 (<0.001)	.0157 (0.817)	.00442 (0.010)
**Applied Ethics (15%)**	**Highly related fields**	Raw citations	816 (<0.001)	328 (0.081)	-3.75 (0.486)
		Composite	3.02 (<0.001)	.0772 (0.427)	.000686 (0.807)
**Organic Chemistry (15%)**	**Other fields**	Raw citations	1337 (<0.001)	263 (0.080)	-1.46 (0.659)
		Composite	2.86 (<0.001)	.0742 (0.062)	.004 (<0.001)
**Nutrition & Dietetics (14%)**	**Highly related fields**	Raw citations	1129 (<0.001)	397 (0.052)	7.14 (0.199)
		Composite	2.98 (<0.001)	.0591 (0.247)	.00485 (0.001)
**Mycology & Parasitology (14%)**	**Highly related fields**	Raw citations	1379 (<0.001)	-244 (0.312)	-9.84 (0.101)
		Composite	2.8 (<0.001)	-.062 (0.480)	.00148 (0.494)
**Nursing (14%)**	**Highly related fields**	Raw citations	158 (0.005)	271 (<0.001)	8.23 (<0.001)
		Composite	2.4 (<0.001)	.0782 (0.015)	.00498 (<0.001)
**Optics (14%)**	**Other fields**	Raw citations	1011 (<0.001)	388 (0.103)	14 (0.038)
		Composite	2.68 (<0.001)	.0331 (0.498)	.00778 (<0.001)

© 2022 Ioannidis JPA et al. *JAMA Network Open.*

**General Chemistry (13%)**	**Other fields**	Raw citations	1421 (<0.001)	127 (0.717)	.507 (0.915)
		Composite	2.62 (<0.001)	.171 (0.009)	.00576 (<0.001)
**General & Internal Medicine (13%)**	**Highly related fields**	Raw citations	3268 (<0.001)	-184 (0.648)	-35.8 (<0.001)
		Composite	2.61 (<0.001)	.159 (<0.001)	.00227 (0.001)
**Surgery (13%)**	**Highly related fields**	Raw citations	1002 (<0.001)	380 (0.002)	4.97 (0.110)
		Composite	2.62 (<0.001)	.0801 (0.002)	.00369 (<0.001)
**Nanoscience & Nanotechnology (13%)**	**Other fields**	Raw citations	1746 (<0.001)	-224 (0.664)	99 (<0.001)
		Composite	2.99 (<0.001)	.0258 (0.561)	.0132 (<0.001)
**Otorhinolaryngology (12%)**	**Highly related fields**	Raw citations	880 (<0.001)	76.9 (0.537)	-4.58 (0.148)
		Composite	2.6 (<0.001)	.0574 (0.101)	.00219 (0.014)
**Environmental & Occupational Health (12%)**	**Highly related fields**	Raw citations	429 (0.240)	-174 (0.594)	11.6 (0.256)
		Composite	2.56 (<0.001)	-.007 (0.926)	.00397 (0.092)
**Tropical Medicine (12%)**	**Highly related fields**	Raw citations	1405 (<0.001)	391 (0.343)	-7.44 (0.436)
		Composite	2.6 (<0.001)	.0677 (0.239)	.00472 (<0.001)
**Microscopy (12%)**	**Highly related fields**	Raw citations	684 (0.229)	16 (0.977)	10.2 (0.490)
		Composite	2.42 (<0.001)	.0482 (0.808)	.0108 (0.046)
**Biotechnology (12%)**	**Highly related fields**	Raw citations	720 (0.003)	56.2 (0.836)	21.1 (0.005)
		Composite	2.64 (<0.001)	.0649 (0.355)	.0109 (<0.001)
**Drama & Theater (10%)**	**Other fields**	Raw citations	82.1 (0.212)	3.11 (0.960)	-.644 (0.765)
		Composite	1.95 (<0.001)	.146 (0.424)	.00562 (0.376)
**Complementary & Alternative Medicine (10%)**	**Highly related fields**	Raw citations	266 (0.035)	297 (0.027)	4.52 (0.295)
		Composite	2.52 (<0.001)	.272 (0.041)	.00146 (0.732)
**Acoustics (9.9%)**	**Other fields**	Raw citations	962 (<0.001)	-7.41 (0.960)	-7.83 (0.006)
		Composite	2.84 (<0.001)	-.0526 (0.428)	.000464 (0.711)
**Dermatology & Venereal Diseases (9.7%)**	**Highly related fields**	Raw citations	1159 (<0.001)	841 (0.003)	1.27 (0.843)
		Composite	2.76 (<0.001)	.0544 (0.380)	.0037 (0.009)

© 2022 Ioannidis JPA et al. *JAMA Network Open.*

**Orthopedics (9.5%)**	**Highly related fields**	Raw citations	1159 (<0.001)	275 (0.023)	-1.15 (0.681)
		Composite	2.8 (<0.001)	.111 (0.001)	.00381 (<0.001)
**Family Studies (9.1%)**	**Other fields**	Raw citations	294 (0.025)	352 (0.027)	6.89 (0.056)
		Composite	2.87 (<0.001)	.0212 (0.833)	.00579 (0.014)
**Environmental Sciences (9%)**	**Other fields**	Raw citations	1628 (<0.001)	1024 (0.023)	2.79 (0.783)
		Composite	3.06 (<0.001)	.147 (0.047)	.00353 (0.036)
**Behavioral Science & Comparative Psychology (8.7%)**	**Highly related fields**	Raw citations	758 (<0.001)	180 (0.292)	.93 (0.793)
		Composite	2.95 (<0.001)	.0981 (0.219)	.00487 (0.004)
**Sociology (7.8%)**	**Other fields**	Raw citations	408 (<0.001)	-53.7 (0.606)	5.98 (0.003)
		Composite	3.06 (<0.001)	-.0488 (0.467)	.00379 (0.004)
**Pathology (7.7%)**	**Highly related fields**	Raw citations	1786 (<0.001)	363 (0.314)	-5.4 (0.489)
		Composite	2.84 (<0.001)	.0321 (0.657)	.00223 (0.154)
**Design Practice & Management (7.5%)**	**Other fields**	Raw citations	619 (<0.001)	510 (0.037)	.181 (0.965)
		Composite	2.79 (<0.001)	.0271 (0.858)	.00125 (0.628)
**Dentistry (7.3%)**	**Highly related fields**	Raw citations	837 (<0.001)	335 (0.013)	-3.29 (0.192)
		Composite	2.72 (<0.001)	.0978 (0.094)	.00214 (0.051)
**Plant Biology & Botany (7.2%)**	**Other fields**	Raw citations	1188 (<0.001)	588 (<0.001)	-1.3 (0.684)
		Composite	2.87 (<0.001)	.0966 (0.031)	.00453 (<0.001)
**Evolutionary Biology (7.1%)**	**Other fields**	Raw citations	1068 (<0.001)	519 (0.185)	8.71 (0.263)
		Composite	2.99 (<0.001)	.115 (0.056)	.00721 (<0.001)
**Artificial Intelligence & Image Processing (6.7%)**	**Other fields**	Raw citations	1792 (<0.001)	-76.2 (0.800)	-4.08 (0.511)
		Composite	2.78 (<0.001)	-.0237 (0.583)	.00717 (<0.001)
**Chemical Physics (6.7%)**	**Other fields**	Raw citations	1697 (<0.001)	264 (0.449)	5.11 (0.312)
		Composite	3.03 (<0.001)	.12 (0.029)	.0042 (<0.001)
**Industrial Engineering & Automation (6.7%)**	**Other fields**	Raw citations	1097 (<0.001)	108 (0.543)	-2.79 (0.421)
		Composite	2.89 (<0.001)	-.0181 (0.777)	.00285 (0.022)

© 2022 Ioannidis JPA et al. *JAMA Network Open.*

**Criminology (6.4%)**	**Other fields**	Raw citations	416 (<0.001)	170 (0.282)	9.35 (0.004)
		Composite	2.99 (<0.001)	.0653 (0.408)	.00519 (0.001)
**Sport Sciences (6.3%)**	**Highly related fields**	Raw citations	912 (0.003)	742 (0.079)	11 (0.242)
		Composite	3.04 (<0.001)	.0609 (0.522)	.00208 (0.328)
**Veterinary Sciences (6.2%)**	**Highly related fields**	Raw citations	479 (<0.001)	126 (0.076)	.954 (0.533)
		Composite	2.51 (<0.001)	.0793 (0.074)	.00298 (0.002)
**Psychoanalysis (6.1%)**	**Highly related fields**	Raw citations	51 (0.729)	182 (0.366)	2.62 (0.329)
		Composite	2.5 (<0.001)	.236 (0.389)	.000928 (0.799)
**Social Work (6%)**	**Other fields**	Raw citations	167 (0.045)	764 (<0.001)	7.4 (0.013)
		Composite	2.75 (<0.001)	.233 (0.013)	.00193 (0.336)
**Polymers (5.9%)**	**Other fields**	Raw citations	1339 (<0.001)	384 (0.160)	-2.5 (0.536)
		Composite	2.89 (<0.001)	-.0114 (0.877)	.00287 (0.009)
**General Clinical Medicine (5.8%)**	**Highly related fields**	Raw citations	285 (0.045)	578 (0.014)	4.57 (0.214)
		Composite	2.21 (<0.001)	.0189 (0.889)	.00713 (0.001)
**Entomology (5.8%)**	**Other fields**	Raw citations	989 (<0.001)	461 (<0.001)	-8.53 (0.001)
		Composite	2.92 (<0.001)	.316 (<0.001)	-.00156 (0.262)
**Religions & Theology (4.9%)**	**Other fields**	Raw citations	143 (0.002)	12.9 (0.895)	-.325 (0.827)
		Composite	2.33 (<0.001)	.0544 (0.763)	.000519 (0.850)
**Distributed Computing (4.9%)**	**Other fields**	Raw citations	352 (0.296)	142 (0.774)	14.3 (0.203)
		Composite	2.37 (<0.001)	.159 (0.267)	.0045 (0.167)
**Computer Hardware & Architecture (4.4%)**	**Other fields**	Raw citations	605 (<0.001)	80.3 (0.647)	2.63 (0.387)
		Composite	2.46 (<0.001)	.0563 (0.528)	.00468 (0.003)
**Information & Library Sciences (4.2%)**	**Other fields**	Raw citations	258 (0.001)	69.6 (0.682)	1.9 (0.429)
		Composite	2.62 (<0.001)	.174 (0.254)	.00193 (0.368)
**Education (4%)**	**Other fields**	Raw citations	350 (<0.001)	263 (0.008)	7.37 (<0.001)
		Composite	2.89 (<0.001)	-.00199 (0.971)	.0042 (<0.001)

© 2022 Ioannidis JPA et al. *JAMA Network Open.*

**Logistics & Transportation (4%)**	**Other fields**	Raw citations	614 (<0.001)	497 (0.121)	8.55 (0.107)
		Composite	2.95 (<0.001)	-.0441 (0.757)	.00361 (0.128)
**Social Psychology (3.9%)**	**Highly related fields**	Raw citations	1214 (<0.001)	269 (0.497)	9.64 (0.048)
		Composite	3.2 (<0.001)	.0938 (0.311)	.00439 (<0.001)
**Applied Mathematics (3.8%)**	**Other fields**	Raw citations	908 (0.026)	1266 (0.082)	9.22 (0.337)
		Composite	3.05 (<0.001)	.156 (0.436)	.005 (0.061)
**Legal & Forensic Medicine (3.8%)**	**Highly related fields**	Raw citations	277 (0.003)	-118 (0.515)	.588 (0.850)
		Composite	2.22 (<0.001)	-.037 (0.837)	.00542 (0.084)
**Physical Chemistry (3.7%)**	**Other fields**	Raw citations	1889 (<0.001)	-162 (0.837)	1.19 (0.893)
		Composite	2.92 (<0.001)	-.0478 (0.798)	.00667 (0.002)
**Development Studies (3.7%)**	**Other fields**	Raw citations	598 (0.013)	194 (0.603)	1.21 (0.849)
		Composite	3.13 (<0.001)	-.137 (0.606)	.00221 (0.630)
**Economics (3.6%)**	**Other fields**	Raw citations	739 (<0.001)	767 (<0.001)	4.58 (0.106)
		Composite	3.08 (<0.001)	.289 (<0.001)	.00517 (<0.001)
**Anatomy & Morphology (3.6%)**	**Highly related fields**	Raw citations	344 (<0.001)	60.3 (0.756)	-1.92 (0.357)
		Composite	2.46 (<0.001)	-.0653 (0.743)	-.000483 (0.821)
**Food Science (3.6%)**	**Other fields**	Raw citations	1047 (0.002)	882 (0.206)	4.11 (0.659)
		Composite	2.88 (<0.001)	.0576 (0.669)	.00521 (0.004)
**Ornithology (3.4%)**	**Other fields**	Raw citations	404 (0.002)	-16.7 (0.952)	.54 (0.850)
		Composite	2.57 (<0.001)	.287 (0.139)	.00354 (0.077)
**Geography (3.3%)**	**Other fields**	Raw citations	318 (0.010)	-236 (0.348)	8.48 (0.018)
		Composite	3 (<0.001)	-.149 (0.302)	.00519 (0.012)
**Electrical & Electronic Engineering (3.3%)**	**Other fields**	Raw citations	426 (<0.001)	130 (0.603)	9.93 (0.004)
		Composite	2.47 (<0.001)	.0131 (0.903)	.00613 (<0.001)
**Networking & Telecommunications (3.3%)**	**Other fields**	Raw citations	880 (<0.001)	-151 (0.387)	-1.87 (0.354)
		Composite	2.62 (<0.001)	-.0672 (0.289)	.00373 (<0.001)

© 2022 Ioannidis JPA et al. *JAMA Network Open.*

**Dairy & Animal Science (3.3%)**	**Other fields**	Raw citations	735 (<0.001)	248 (0.076)	-3.44 (0.067)
		Composite	2.64 (<0.001)	-.0237 (0.792)	.0023 (0.058)
**General Psychology & Cognitive Sciences (3.1%)**	**Highly related fields**	Raw citations	699 (<0.001)	-340 (0.344)	-.0267 (0.995)
		Composite	3.02 (<0.001)	-.286 (0.185)	.000522 (0.852)
**Fluids & Plasmas (3.1%)**	**Other fields**	Raw citations	1638 (<0.001)	291 (0.579)	-10.1 (0.111)
		Composite	3.11 (<0.001)	.157 (0.163)	.00125 (0.360)
**Social Sciences Methods (3%)**	**Other fields**	Raw citations	1036 (0.073)	2227 (0.117)	8.28 (0.520)
		Composite	3.18 (<0.001)	.701 (0.003)	.00289 (0.173)
**Numerical & Computational Mathematics (3%)**	**Other fields**	Raw citations	490 (0.014)	829 (0.025)	6.65 (0.163)
		Composite	3.07 (<0.001)	.364 (0.040)	.000555 (0.807)
**Building & Construction (2.9%)**	**Other fields**	Raw citations	780 (<0.001)	363 (0.457)	12.2 (0.078)
		Composite	2.94 (<0.001)	-.0224 (0.898)	.00725 (0.004)
**Mechanical Engineering & Transports (2.9%)**	**Other fields**	Raw citations	1048 (<0.001)	-.278 (0.999)	-4.51 (0.178)
		Composite	2.83 (<0.001)	-.092 (0.384)	.0034 (0.004)
**Science Studies (2.9%)**	**Other fields**	Raw citations	217 (0.353)	62.4 (0.894)	12.7 (0.051)
		Composite	3 (<0.001)	-.136 (0.688)	.00692 (0.136)
**Cultural Studies (2.8%)**	**Other fields**	Raw citations	74.7 (0.319)	-92.6 (0.578)	2.56 (0.252)
		Composite	2.44 (<0.001)	-.17 (0.551)	.0056 (0.147)
**Marketing (2.8%)**	**Other fields**	Raw citations	194 (0.380)	-19.8 (0.962)	27.6 (<0.001)
		Composite	3.01 (<0.001)	.04 (0.735)	.00568 (0.005)
**Anthropology (2.6%)**	**Other fields**	Raw citations	288 (<0.001)	184 (0.229)	4.94 (0.016)
		Composite	2.97 (<0.001)	-.129 (0.348)	.00266 (0.146)
**Materials (2.4%)**	**Other fields**	Raw citations	1235 (<0.001)	275 (0.415)	-1.51 (0.626)
		Composite	2.76 (<0.001)	.131 (0.098)	.00504 (<0.001)
**Operations Research (2.4%)**	**Other fields**	Raw citations	518 (0.042)	10.5 (0.985)	12.9 (0.053)
		Composite	3.09 (<0.001)	.0935 (0.562)	.00317 (0.101)

© 2022 Ioannidis JPA et al. *JAMA Network Open.*

**Mining & Metallurgy (2.3%)**	**Other fields**	Raw citations	225 (0.148)	-103 (0.778)	4.22 (0.278)
		Composite	2.31 (<0.001)	-.105 (0.755)	.00577 (0.113)
**Optoelectronics & Photonics (2.3%)**	**Other fields**	Raw citations	486 (<0.001)	327 (0.052)	1.69 (0.360)
		Composite	2.31 (<0.001)	.166 (0.050)	.00571 (<0.001)
**Human Factors (2.3%)**	**Highly related fields**	Raw citations	731 (<0.001)	266 (0.487)	7.31 (0.141)
		Composite	2.88 (<0.001)	.218 (0.121)	.00767 (<0.001)
**Software Engineering (2.2%)**	**Other fields**	Raw citations	1082 (<0.001)	240 (0.313)	-12.9 (<0.001)
		Composite	2.8 (<0.001)	-.162 (0.189)	.000988 (0.568)
**Finance (2.2%)**	**Other fields**	Raw citations	568 (0.004)	-345 (0.451)	14.1 (0.018)
		Composite	3 (<0.001)	-.0545 (0.743)	.00713 (0.001)
**Meteorology & Atmospheric Sciences (2.2%)**	**Other fields**	Raw citations	1773 (<0.001)	157 (0.592)	1.23 (0.733)
		Composite	3.09 (<0.001)	.0653 (0.287)	.00335 (<0.001)
**Communication & Media Studies (2.1%)**	**Other fields**	Raw citations	107 (0.444)	129 (0.770)	20.5 (<0.001)
		Composite	3.04 (<0.001)	.00959 (0.945)	.00424 (0.006)
**Econometrics (2%)**	**Other fields**	Raw citations	624 (0.460)	505 (0.769)	26.3 (0.243)
		Composite	3.25 (<0.001)	-.296 (0.534)	.00695 (0.264)
**Political Science & Public Administration (2%)**	**Other fields**	Raw citations	463 (<0.001)	1092 (<0.001)	6.15 (0.040)
		Composite	3.05 (<0.001)	.312 (0.007)	.00406 (0.001)
**Languages & Linguistics (2%)**	**Other fields**	Raw citations	422 (<0.001)	35.6 (0.860)	.804 (0.738)
		Composite	3.04 (<0.001)	.182 (0.340)	.00281 (0.218)
**General Physics (2%)**	**Other fields**	Raw citations	1270 (<0.001)	381 (0.335)	-6.75 (0.032)
		Composite	2.78 (<0.001)	.101 (0.419)	.00337 (0.001)
**Archaeology (2%)**	**Other fields**	Raw citations	418 (<0.001)	-1.75 (0.995)	.99 (0.726)
		Composite	2.81 (<0.001)	.0493 (0.789)	.00164 (0.344)
**Sport, Leisure & Tourism (1.9%)**	**Other fields**	Raw citations	696 (<0.001)	-46.8 (0.900)	4.47 (0.387)
		Composite	3.08 (<0.001)	.0348 (0.850)	.00429 (0.096)

© 2022 Ioannidis JPA et al. *JAMA Network Open.*

**Environmental Engineering (1.9%)**	**Other fields**	Raw citations	1071 (<0.001)	1489 (<0.001)	-2.14 (0.608)
		Composite	2.97 (<0.001)	.156 (0.188)	.00197 (0.127)
**Energy (1.9%)**	**Other fields**	Raw citations	1012 (<0.001)	454 (0.087)	.673 (0.784)
		Composite	2.85 (<0.001)	.0496 (0.540)	.00395 (<0.001)
**Marine Biology & Hydrobiology (1.7%)**	**Other fields**	Raw citations	1519 (<0.001)	379 (0.355)	-8.78 (0.094)
		Composite	3.05 (<0.001)	.0472 (0.711)	.00222 (0.172)
**Ecology (1.6%)**	**Other fields**	Raw citations	1288 (<0.001)	263 (0.563)	11.7 (0.024)
		Composite	3.07 (<0.001)	.154 (0.095)	.00552 (<0.001)
**Chemical Engineering (1.6%)**	**Other fields**	Raw citations	1543 (<0.001)	-113 (0.850)	-8.93 (0.056)
		Composite	2.98 (<0.001)	.215 (0.265)	.00205 (0.170)
**Philosophy (1.6%)**	**Other fields**	Raw citations	242 (<0.001)	476 (<0.001)	.346 (0.761)
		Composite	2.91 (<0.001)	.712 (<0.001)	.000684 (0.676)
**Aerospace & Aeronautics (1.6%)**	**Other fields**	Raw citations	403 (<0.001)	142 (0.229)	-.121 (0.913)
		Composite	2.44 (<0.001)	-.0368 (0.723)	.00447 (<0.001)
**Inorganic & Nuclear Chemistry (1.5%)**	**Other fields**	Raw citations	951 (<0.001)	-182 (0.828)	4.34 (0.458)
		Composite	2.77 (<0.001)	-.0123 (0.951)	.00294 (0.036)
**Agricultural Economics & Policy (1.4%)**	**Other fields**	Raw citations	990 (<0.001)	-8.71 (0.989)	-2.71 (0.698)
		Composite	3.24 (<0.001)	.033 (0.915)	-.0016 (0.641)
**Geological & Geomatics Engineering (1.4%)**	**Other fields**	Raw citations	1385 (<0.001)	399 (0.462)	-2.71 (0.616)
		Composite	2.98 (<0.001)	.19 (0.233)	.00485 (0.002)
**Accounting (1.4%)**	**Other fields**	Raw citations	465 (0.014)	-178 (0.713)	12.2 (0.036)
		Composite	2.92 (<0.001)	.0437 (0.875)	.00806 (0.017)
**Applied Physics (1.2%)**	**Other fields**	Raw citations	1454 (<0.001)	257 (0.535)	-2.28 (0.392)
		Composite	2.84 (<0.001)	.0137 (0.874)	.00263 (<0.001)
**Business & Management (1.2%)**	**Other fields**	Raw citations	761 (<0.001)	14.3 (0.971)	12.4 (0.001)
		Composite	3.11 (<0.001)	.0147 (0.894)	.00466 (<0.001)

© 2022 Ioannidis JPA et al. *JAMA Network Open.*

**Information Systems (1.2%)**	**Other fields**	Raw citations	1196 (<0.001)	629 (0.504)	-1.17 (0.906)
		Composite	3.16 (<0.001)	.321 (0.195)	.000241 (0.926)
**Computation Theory & Mathematics (1.1%)**	**Other fields**	Raw citations	696 (<0.001)	-77.8 (0.878)	-.305 (0.939)
		Composite	2.93 (<0.001)	.0902 (0.675)	.00232 (0.173)
**Astronomy & Astrophysics (.94%)**	**Other fields**	Raw citations	3121 (<0.001)	113 (0.898)	-23.8 (<0.001)
		Composite	3.17 (<0.001)	.101 (0.332)	.000634 (0.428)
**Geochemistry & Geophysics (.81%)**	**Other fields**	Raw citations	1302 (<0.001)	-173 (0.583)	-4.81 (0.042)
		Composite	3.15 (<0.001)	-.0982 (0.408)	.00175 (0.049)
**Strategic, Defence & Security Studies (.77%)**	**Other fields**	Raw citations	244 (0.106)	-462 (0.483)	11.1 (0.013)
		Composite	2.7 (<0.001)	-.125 (0.695)	.00778 (<0.001)
**Paleontology (.74%)**	**Other fields**	Raw citations	1198 (<0.001)	-586 (0.551)	-4.82 (0.489)
		Composite	3.14 (<0.001)	-.258 (0.241)	-.0000303 (0.984)
**Literary Studies (.72%)**	**Other fields**	Raw citations	30.3 (0.414)	67.7 (0.648)	1.96 (0.143)
		Composite	1.93 (<0.001)	.635 (0.089)	.00804 (0.017)
**General Mathematics (.71%)**	**Other fields**	Raw citations	447 (<0.001)	391 (0.094)	-.82 (0.452)
		Composite	2.9 (<0.001)	.0651 (0.685)	.000633 (0.399)
**Nuclear & Particle Physics (.63%)**	**Other fields**	Raw citations	3770 (<0.001)	2194 (0.129)	-39.9 (<0.001)
		Composite	2.89 (<0.001)	.224 (0.106)	.00254 (<0.001)
**Oceanography (.6%)**	**Other fields**	Raw citations	1004 (<0.001)	262 (0.634)	-5.42 (0.136)
		Composite	2.92 (<0.001)	-.267 (0.320)	.00165 (0.352)
**Agronomy & Agriculture (.55%)**	**Other fields**	Raw citations	1183 (<0.001)	839 (0.265)	-3.06 (0.455)
		Composite	2.88 (<0.001)	.343 (0.133)	.00282 (0.024)
**Fisheries (.53%)**	**Other fields**	Raw citations	710 (<0.001)	425 (0.393)	.938 (0.788)
		Composite	2.77 (<0.001)	.109 (0.701)	.00287 (0.151)
**Zoology (0%)**	**Other fields**	Raw citations			
		Composite			

© 2022 Ioannidis JPA et al. *JAMA Network Open.*

**Urban & Regional Planning (0%)**	**Other fields**	Raw citations			
		Composite			
**Music (0%)**	**Other fields**	Raw citations			
		Composite			
**Mathematical Physics (0%)**	**Other fields**	Raw citations			
		Composite			
**Law (0%)**	**Other fields**	Raw citations			
		Composite			
**International Relations (0%)**	**Other fields**	Raw citations			
		Composite			
**Industrial Relations (0%)**	**Other fields**	Raw citations			
		Composite			
**Horticulture (0%)**	**Other fields**	Raw citations			
		Composite			
**History of Social Sciences (0%)**	**Other fields**	Raw citations			
		Composite			
**History of Science, Technology & Medicine (0%)**	**Other fields**	Raw citations			
		Composite			
**History (0%)**	**Other fields**	Raw citations			
		Composite			
**Geology (0%)**	**Other fields**	Raw citations			
		Composite			
**Gender Studies (0%)**	**Highly related fields**	Raw citations			
		Composite			
**Forestry (0%)**	**Other fields**	Raw citations			
		Composite			
**Folklore (0%)**	**Other fields**	Raw citations			
		Composite			
**Economic Theory (0%)**	**Other fields**	Raw citations			
		Composite			
**Classics (0%)**	**Other fields**	Raw citations			
		Composite			
**Civil Engineering (0%)**	**Other fields**	Raw citations			
		Composite			

© 2022 Ioannidis JPA et al. *JAMA Network Open.*

**Automobile Design & Engineering (0%)**	**Other fields**	Raw citations			
		Composite			
**Art Practice, History & Theory (0%)**	**Other fields**	Raw citations			
		Composite			
**Architecture (0%)**	**Other fields**	Raw citations			
		Composite			

© 2022 Ioannidis JPA et al. *JAMA Network Open.*

eTable 4. Mixed-Effects Linear Regressions

eTable 4.1: Mixed-effects linear regressions grouped by subfields

Top-cited US-based researchers	Funding time	Dependent Variable	Constant (p-val)	Funded (p-val)	Years since first pub (p-val)
**Career-long impact**	**Any funding**	Raw citations	5763 (<0.001)	3370 (<0.001)	13 (<0.001)
		Composite	3.34 (<0.001)	.115 (<0.001)	.00372 (<0.001)
Highly related fields		Raw citations	6799 (<0.001)	3724 (<0.001)	19.8 (0.001)
		Composite	3.36 (<0.001)	.126 (<0.001)	.00412 (<0.001)
Other fields		Raw citations	5086 (<0.001)	2515 (<0.001)	6.34 (0.095)
		Composite	3.32 (<0.001)	.0914 (<0.001)	.00334 (<0.001)
**Career-long impact**	**Recent funding**	Raw citations	6502 (<0.001)	2386 (<0.001)	13.1 (<0.001)
		Composite	3.37 (<0.001)	.0636 (<0.001)	.00363 (<0.001)
Highly related fields		Raw citations	8346 (<0.001)	2404 (<0.001)	20.3 (0.001)
		Composite	3.43 (<0.001)	.0609 (<0.001)	.00396 (<0.001)
Other fields		Raw citations	5321 (<0.001)	2393 (<0.001)	5.88 (0.123)
		Composite	3.33 (<0.001)	.0825 (<0.001)	.00331 (<0.001)
**Career-long impact**	**Current funding**	Raw citations	6786 (<0.001)	2130 (<0.001)	8.93 (0.011)
		Composite	3.38 (<0.001)	.0546 (<0.001)	.00352 (<0.001)
Highly related fields		Raw citations	8861 (<0.001)	2069 (<0.001)	13.1 (0.024)
		Composite	3.44 (<0.001)	.05 (<0.001)	.00377 (<0.001)
Other fields		Raw citations	5422 (<0.001)	2457 (<0.001)	4.53 (0.235)
		Composite	3.34 (<0.001)	.084 (<0.001)	.00327 (<0.001)
**Recent year impact**	**Any funding**	Raw citations	1087 (<0.001)	305 (<0.001)	-2.13 (<0.001)
		Composite	2.78 (<0.001)	.0829 (<0.001)	.00383 (<0.001)

© 2022 Ioannidis JPA et al. *JAMA Network Open.*

Highly related fields		Raw citations	1343 (<0.001)	323 (<0.001)	-3.34 (0.001)
		Composite	2.78 (<0.001)	.0952 (<0.001)	.00386 (<0.001)
Other fields		Raw citations	895 (<0.001)	250 (<0.001)	-.979 (0.130)
		Composite	2.78 (<0.001)	.056 (<0.001)	.00378 (<0.001)
**Recent year impact**	**Recent funding**	Raw citations	1096 (<0.001)	273 (<0.001)	-.754 (0.213)
		Composite	2.79 (<0.001)	.0581 (<0.001)	.00414 (<0.001)
Highly related fields		Raw citations	1369 (<0.001)	275 (<0.001)	-.849 (0.399)
		Composite	2.79 (<0.001)	.0594 (<0.001)	.00444 (<0.001)
Other fields		Raw citations	905 (<0.001)	239 (<0.001)	-.691 (0.287)
		Composite	2.78 (<0.001)	.0592 (<0.001)	.00385 (<0.001)
**Recent year impact**	**Current funding**	Raw citations	1117 (<0.001)	267 (<0.001)	-.945 (0.119)
		Composite	2.79 (<0.001)	.0514 (<0.001)	.00407 (<0.001)
Highly related fields		Raw citations	1409 (<0.001)	259 (<0.001)	-1.22 (0.224)
		Composite	2.81 (<0.001)	.0505 (<0.001)	.00432 (<0.001)
Other fields		Raw citations	911 (<0.001)	277 (<0.001)	-.723 (0.265)
		Composite	2.79 (<0.001)	.0635 (<0.001)	.00384 (<0.001)

Funding time is defined as 'any': any grant entry in Reporter; 'recent': any entry in the Reporter that covers any year in the period 2015 until 2022; 'current': any entry in the Reporter that covers 2021 and/or 2022.

Highly related fields (according to the Science-Metrix classification) are 69 subfields, i.e. the 60 subfields within the larger fields of Biomedical Research, Clinical Medicine, Public Health and Health Services, and Psychology and Cognitive Sciences as well as 9 prespecified subfields from other large fields, i.e. Applied Ethics, Bioinformatics, Biomedical Engineering, Biotechnology, Demography, Gender Studies, Medical Informatics, Medicinal and Biomolecular Chemistry, and Veterinary Sciences.

© 2022 Ioannidis JPA et al. *JAMA Network Open.*

eTable 4.2: Mixed-effects linear regressions grouped by subfields (the number of citations in these regressions was transformed by logarithm, so the regression coefficients for citations are multiplicative).

Top-cited US-based researchers	Funding time	Dependent Variable	Constant (p-val)	Funded (p-val)	Years since first pub (p-val)	Number (% funded)	Prob > chi2	Wald chi2
**Career-long impact**	**Any funding**	Raw citations (mult.)	4230 (<0.001)	1.39 (<0.001)	.997 (<0.001)	75316 (40%)	<0.001	2643
		Composite (add.)	3.34 (<0.001)	.115 (<0.001)	.00372 (<0.001)		<0.001	3001
Highly related fields		Raw citations (mult.)	5541 (<0.001)	1.42 (<0.001)	.998 (<0.001)	40887 (63%)	<0.001	2076
		Composite (add.)	3.36 (<0.001)	.126 (<0.001)	.00412 (<0.001)		<0.001	2152
Other fields		Raw citations (mult.)	3533 (<0.001)	1.32 (<0.001)	.997 (<0.001)	34429 (14%)	<0.001	628
		Composite (add.)	3.32 (<0.001)	.0914 (<0.001)	.00334 (<0.001)		<0.001	917
**Career-long impact**	**Recent funding**	Raw citations (mult.)	4583 (<0.001)	1.23 (<0.001)	.997 (<0.001)	75316 (15%)	<0.001	954
		Composite (add.)	3.37 (<0.001)	.0636 (<0.001)	.00363 (<0.001)		<0.001	1674
Highly related fields		Raw citations (mult.)	6503 (<0.001)	1.22 (<0.001)	.997 (<0.001)	40887 (23%)	<0.001	624
		Composite (add.)	3.43 (<0.001)	.0609 (<0.001)	.00396 (<0.001)		<0.001	984
Other fields		Raw citations (mult.)	3641 (<0.001)	1.32 (<0.001)	.997 (<0.001)	34429 (5.3%)	<0.001	351
		Composite (add.)	3.33 (<0.001)	.0825 (<0.001)	.00331 (<0.001)		<0.001	711
**Career-long impact**	**Current funding**	Raw citations (mult.)	4675 (<0.001)	1.2 (<0.001)	.997 (<0.001)	75316 (9.1%)	<0.001	659
		Composite (add.)	3.38 (<0.001)	.0546 (<0.001)	.00352 (<0.001)		<0.001	1497
Highly related fields		Raw citations (mult.)	6768 (<0.001)	1.18 (<0.001)	.997 (<0.001)	40887 (14%)	<0.001	409
		Composite (add.)	3.44 (<0.001)	.05 (<0.001)	.00377 (<0.001)		<0.001	846
Other fields		Raw citations (mult.)	3678 (<0.001)	1.31 (<0.001)	.997 (<0.001)	34429 (3.1%)	<0.001	269
		Composite (add.)	3.34 (<0.001)	.084 (<0.001)	.00327 (<0.001)		<0.001	670
**Recent year impact**	**Any funding**	Raw citations (mult.)	659 (<0.001)	1.28 (<0.001)	.996 (<0.001)	65560 (42%)	<0.001	1365
		Composite (add.)	2.78 (<0.001)	.0829 (<0.001)	.00383 (<0.001)		<0.001	2356

© 2022 Ioannidis JPA et al. *JAMA Network Open.*

Highly related fields		Raw citations (mult.)	854 (<0.001)	1.3 (<0.001)	.996 (<0.001)	35787 (65%)	<0.001	929
		Composite (add.)	2.78 (<0.001)	.0952 (<0.001)	.00386 (<0.001)		<0.001	1524
Other fields		Raw citations (mult.)	550 (<0.001)	1.23 (<0.001)	.996 (<0.001)	29773 (15%)	<0.001	439
		Composite (add.)	2.78 (<0.001)	.056 (<0.001)	.00378 (<0.001)		<0.001	877
**Recent year impact**	**Recent funding**	Raw citations (mult.)	665 (<0.001)	1.21 (<0.001)	.997 (<0.001)	65560 (20%)	<0.001	812
		Composite (add.)	2.79 (<0.001)	.0581 (<0.001)	.00414 (<0.001)		<0.001	1943
Highly related fields		Raw citations (mult.)	889 (<0.001)	1.2 (<0.001)	.998 (<0.001)	35787 (31%)	<0.001	487
		Composite (add.)	2.79 (<0.001)	.0594 (<0.001)	.00444 (<0.001)		<0.001	1120
Other fields		Raw citations (mult.)	556 (<0.001)	1.27 (<0.001)	.996 (<0.001)	29773 (6.5%)	<0.001	352
		Composite (add.)	2.78 (<0.001)	.0592 (<0.001)	.00385 (<0.001)		<0.001	835
**Recent year impact**	**Current funding**	Raw citations (mult.)	679 (<0.001)	1.19 (<0.001)	.997 (<0.001)	65560 (13%)	<0.001	611
		Composite (add.)	2.79 (<0.001)	.0514 (<0.001)	.00407 (<0.001)		<0.001	1825
Highly related fields		Raw citations (mult.)	916 (<0.001)	1.18 (<0.001)	.998 (<0.001)	35787 (21%)	<0.001	341
		Composite (add.)	2.81 (<0.001)	.0505 (<0.001)	.00432 (<0.001)		<0.001	1014
Other fields		Raw citations (mult.)	561 (<0.001)	1.27 (<0.001)	.996 (<0.001)	29773 (4%)	<0.001	296
		Composite (add.)	2.79 (<0.001)	.0635 (<0.001)	.00384 (<0.001)		<0.001	820

© 2022 Ioannidis JPA et al. *JAMA Network Open.*
