# Supplementary material for: Federal Funding and Citation Metrics of US Biomedical Researchers, 1996 to 2022
Source: JAMA Netw Open. 2022 Dec 7;5(12):e2245590. doi: 10.1001/jamanetworkopen.2022.45590 (PMC9856501; doi:10.1001/jamanetworkopen.2022.45590)
Supplement: Supplement 2. — Data Sharing Statement [file jamanetwopen-e2245590-s002.pdf]

Data Sharing Statement

Ioannidis. Federal Funding and Citation Metrics of US Biomedical Researchers, 1996 to 2022. *JAMA Netw Open*. Published December 07, 2022. doi:10.1001/jamanetworkopen.2022.45590

Data
Data available: Yes
Data types: Other (please specify)
Additional Information: Both the citation and funding databases are publicly available.
How to access data: https://dx.doi.org/10.17632/btchxktzyw
https://data.mendeley.com/datasets/btchxktzyw/3 https://reporter.nih.gov
When available: With publication

Supporting Documents
Document types: None

Additional Information
Who can access the data: Anyone (already publicly available)
Types of analyses: Anything (already publicly available)
Mechanisms of data availability: Already available in public repositories
